# *Ctenosciara alexanderkoenigi* sp. n. (Diptera: Sciaridae), an exotic invader in Germany?

**DOI:** 10.3897/BDJ.4.e6460

**Published:** 2016-04-01

**Authors:** Kai Heller, Björn Rulik

**Affiliations:** ‡ Unaffiliated, Quickborn, Germany; § Zoologisches Forschungsmuseum Alexander Koenig, Bonn, Germany

**Keywords:** New species, faunistics, invasive species, DNA barcoding, identification key

## Abstract

A new species of the genus *Ctenosciara* Tuomikoski, 1960 is here described based upon a single specimen, obtained from collectings in the garden at Museum Alexander Koenig in Bonn. *Ctenosciara alexanderkoenigi* **sp. n.** differs from all other congeneric European species by its striking coloration and distinct male genitalia. However, DNA barcoding reveals associations with two specimens from New Zealand. Therefore a recent migration of *Ctenosciara* species from the Australasian Region, the likely center of origin of the genus, is discussed. A key to the European species of *Ctenosciara* is provided. Barcoding results reveale that *Ctenosciara exigua* is not clearly distinguished from *Ctenosciara hyalipennis* by its COI sequence (both share the same BIN BOLD:AAH3983) and that its species status may be questionable.

## Introduction

*Ctenosciara* was erected by Tuomikoski (1960) and at the time was a monotypic genus including a common European species *Ctenosciara hyalipennis* (Meigen, 1804). In Europe this genus is very species poor with three species currently known. Two species, *Ctenosciara hyalipennis* and *Ctenosciara lutea* (Meigen, 1804) the latter combined by Menzel et al. (1990) are very abundant while the third species, *Ctenosciara exigua* (Salmela & Vilkamaa, 2005), described from Finland (Salmela and Vilkamaa 2005), is very rare. While no *Ctenosciara* species are known from North America (Mohrig et al. 2013), the number of species rises towards the eastern Palaearctic Region with 6 species in China (Wu et al. 2010) and 7 in Japan (Sutou and Ito 2003). The Oriental Region has not been studied sufficiently enough to make estimations of species richness of the genus there, but the Australasian Region appears to be the center of diversification for *Ctenosciara*: 10 species are known from Papua New Guinea (Mohrig 2013), 8 species from New Caledonia (Vilkamaa et al. 2012) and 7 species from New Zealand (Mohrig and Jaschhof 1999). A preliminary examination of Southern Australian material, mostly from pitfall traps (Heller and Mohrig pers. obs.), indicates that *Ctenosciara* is by far the most dominant genus of Sciaridae on the Australian continent with approximately several hundred different species. Therefore, it is surprising that Malaise trap sampling in the garden of the Koenig Museum in the city of Bonn (Germany), revealed a male specimen of another *Ctenosciara* species which is strongly different from the other European species. Fortunately, it was possible to analyze the DNA of the specimen in conjunction with GBOL, thus facilitating not only a morphological but also a genetic comparison of the new species.

## Materials and methods

**Figure 1.**
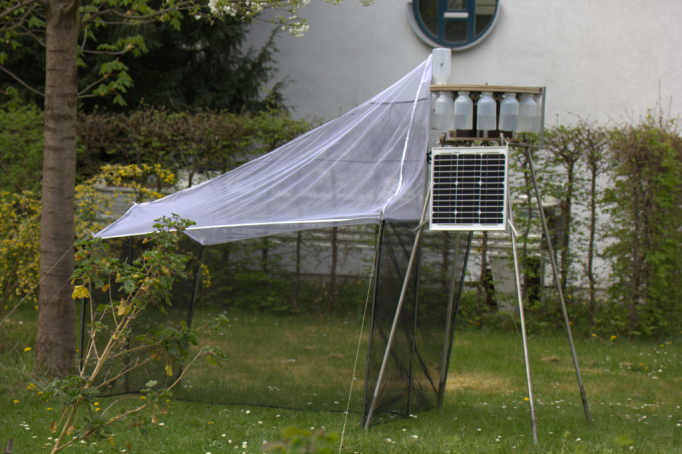
Locus typicus of *Ctenosciara alexanderkoenigi* **sp. n**. in the garden of Museum Koenig, late spring, 2014. Malaise trap is equipped with the prototype of the AMTC.

The new species was extracted from the catch of a standard malaise trap equipped with a prototype of the Automatic Malaise Trap Changer (AMTC; Rulik et al. 2014) in Bonn, Germany. From June 5th to June 7th 2014 the malaise trap with AMTC was placed in the garden of Museum Koenig for testing purposes (Fig. 1). Collection bottles were filled with 96% ethanol as a preservative. The garden is embedded in a 7000m^2^ park containing more than 80 species of predominantly non-native trees and shrubs. The park was established under the leadership of Alexander Koenig presumbably in the early 1890's. What was initially a relatively species poor grassy lawn, the area was morphed into natural meadows and hedgerows while being managed to further promote biodiversity (Hutterer et al. 2012).

### Description and specimen deposition

Habitus photos were captured with the aid of a Canon D60 camera fitted with a MP-E 65mm macro photo lens. More detailed close-up photos of specimens were created using a MCA-510 USB microscope camera by TUCSEN (Xintu Photonics Co., Ltd.). Between 15 and 40 images taken at different focal lengths were merged with the aid of the Public Domain Software CombineZP using “Weighted Average” method. All images were retouched using the freely available software GIMP, version 2.8.0. Species descriptions were prepared using DELTA (DEscription Language for TAxonomy) (Dallwitz et al. 1999). Measurements were taken from the photos, whereby a standard range of variability as known from other Sciaridae species was assumed. The specimens are deposited in Zoologisches Forschungsmuseum Alexander Koenig, Bonn, Germany (ZFMK).

### Molecular Analysis

G enomic DNA was extracted at ZFMK from the entire specimen using the BioSprint96 magnetic bead extractor by Qiagen (Hilden, Germany). Polymerase chain reaction (PCR) was carried out in total reaction mixes of 20 μl, including 2 μl of undiluted DNA template, 0,8 μl of each primer (10 pmol/μl), 2 μl of ‘Q-Solution’ and 10 μl of ‘Multiplex PCR Master Mix’, containing hot start Taq DNA polymerase and buffers. The latter components are available in the Multiplex PCR kit from Qiagen (Hilden, Germany). PCR reactions were run individually and not multiplexed.

Thermal cycling was performed on GeneAmp PCR System 2700 (Applied Biosystems, Foster City, CA, USA) as follows: hot start Taq activation: 15 min at 95°C; first cycle set (15 repeats): 35-s denaturation at 94°C, 90-s annealing at 55°C (−1 °C/cycle) and 90-s extension at 72°C. Second cycle set (25 repeats): 35-s denaturation at 94°C, 90-s annealing at 40°C and 90-s extension at 72°C; final elongation 10 min at 72°C using the primers LCO1490: 5´-GGTCAACAAATCATAAAGATATTGG- 3´ and C1-N-2191 (aka Nancy): 5´-CCCGGTAAAATTAAAATATAAACTTC- 3´ (Folmer et al. 1994, Simon et al. 1994) or combining LCO1490-JJ: 5´-CHACWAAYCATAAAGATATYGG- 3´ with HCO2198-JJ: 5´-AWACTTCVGGRTGVCCAAARAATCA- 3´ respectively (Astrin and Stüben 2008).

Sequencing of the unpurified PCR products in both directions was conducted at Beijing Genomics Institute (Hongkong, CN). Sequence analysis was done using the Geneious® software version 7.1.7 (http://www.geneious.com). All sequences were deposited in BOLD (http://dx.doi.org/10.5883/DS-CTENSCIA) and GenBank under accession numbers KT601633-KT601635 .

### Data analysis

Public BOLD API was queried for distribution pattern of Nearctic Sciaridae in order to test for sampling bias (Suppl. material 1). All records with coordinates (N=72611) were used to plot occurences of Sciaridae with the aid of the Diversity GIS Editor 2.2.4.1 as standalone module of the Diversity Workbench software suite (http://diversityworkbench.net/Portal/Software). Publicly available sequences (N=4047) of BIN AAH3983 (with applied filter: longer than 500bp, without contaminants and without stop codons) were downloaded from BOLD. An alignment was build using the MUSCLE algorithm (Edgar 2004) checked manually and trimmed to the 658bp long barcode region before continue processing analysis (Suppl. material 2). DNASP version 5.10 (Librado and Rozas 2009) was employed for single nucleotide polymorphism for calculating variable sites and quantifying haplotypes. Calculation of nucleotid statistics and pairwise distances using the Kimura 2-parameter (K2P) model were performed with MEGA6 (Tamura et al. 2013). A neighbor joining tree was generated using the buildin BOLD TaxonID tool for visualisation of genetic distances.

## Taxon treatments

### Ctenosciara alexanderkoenigi

Heller & Rulik 2016 sp. n.

HRCTE001-15

urn:lsid:zoobank.org:act:1E97DF91-7C19-4C18-9A1C-E4DFC9CB3E5C

#### Materials

- **Type status:** Holotype. **Occurrence:** catalogNumber: ZFMK-TIS-2527968; recordedBy: Björn Rulik; individualCount: 1; sex: male; lifeStage: adult; preparations: slide; otherCatalogNumbers: ZFMK-DIP-00011896; **Taxon:** scientificName: Ctenosciara alexanderkoenigi; genus: Ctenosciara; specificEpithet: alexanderkoenigi; scientificNameAuthorship: Heller & Rulik, 2016; **Location:** country: Germany; countryCode: DE; stateProvince: North-Rhine-Westphalia; county: Cologne; municipality: Bonn; locality: Museum Koenig; verbatimElevation: 67 m; decimalLatitude: 50.721944; decimalLongitude: 7.113611; **Event:** samplingProtocol: Malaise trap; eventDate: 07/06/2014; startDayOfYear: 155; endDayOfYear: 159; year: 2014; month: 6; day: 7; habitat: museum´s garden; **Record Level:** institutionCode: ZFMK

#### Description

**Head**. Eye bridge 2–3 rows of facets. Antenna with scape and pedicel brightened. LW-index of 4^th^ antennal flagellar segment 2.65; neck 0.35 × the segment width (Fig. 2d); some pale sensillae present. Transition of basal part to neck pronounced. Neck unicolour. Antennal setae shorter than segment width; of normal strength; sparse; salient. Palpus bright; with three palpomeres. First palpomere of normal shape; with 2 bristles; with only some sparse sensillae, or with delimited sensillary field. Second palpomere short oval. Third palpomere as long as first segment. **Thorax**. Colour reddish, bicolour. Notum partially brightened. Thoracic setae long and strong, or normal; black. Mesonotum with some weaker central bristles. Posterior pronotum bare. Mesothoracic sclerites bare. *Legs*. Colour yellow-white. Hind coxa of same colour as femur. Hairs on fore coxa black. Front tibia apically with a distinct, delimited comb (Fig. 2e). Tibial comb undivided, with 7–8 bristles. Setae of front tibial organ bright. Front tibial organ distinctly bordered. Tibial setae on hind legs weak, inconspicuous. Tibial spurs of equal length. Claws untoothed. *Wing* (Fig. 2f). Slightly darkened; of normal shape. Wing membrane without macrotrichia. Wing venation weak, with faint stM. M-fork of normal shape. R_1_ ending clearly before base of m-fork; posterior veins with macrotrichia; stM bare; CuA_1_ with and CuA_2_ without macrotrichia; bM bare; r-m with a few setae; bM:r-M 1.1; st-Cu:bM 0.7; R_1_:R 0.47; c:w 0.7. Halter dark; of normal length. **Abdomen**. Abdominal setae strong and dense; tergal setae black; sternal setae black. Hypopygium (Fig. 2a) brighter than abdomen; 0.62 (0.55–0.70) × longer than wide. Base of gonocoxite with normal, weak hairs; gonocoxites fused; inner margin of gonocoxite narrowly U-shaped; inner membrane of hypopygium bare; elongated setae on valves of hypopygium absent. Gonostylus (Fig. 2b) elongate; 3.6 × longer than wide; Inner margin straight, or convex; apex tapered. Apical tooth present; as long or longer than subapical megasetae; ca. 5.6 × longer than wide; strong. Megasetae present subapically; number of megasetae 5; thick; curved; in one group; Posososition of basalmost megaseta 36 (32–40) % from top. Tegmen (Fig. 2c) nearly as long as broad; equally rounded; central process absent. Length of ejaculatory apodeme about 15 % of hypopygium; Aeadeagal apical structure absent. Field with aedeagal teeth present. **Measurements**. Body size ca. 2.2mm. Hind tibia length 1.05 mm. Wing length 2.0 mm.

#### Diagnosis

**Figure 3.**
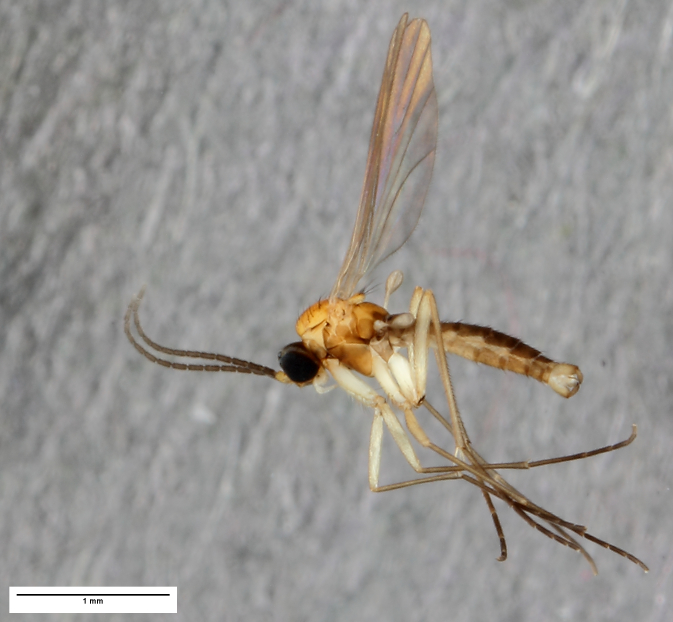
*Ctenosciara alexanderkoenigi* **sp. n.**, habitus photograph, scale 1 mm.

This beautiful species is conspicuous among the European species of *Ctenosciara* by its eye-catching and contrasting coloration (Fig. 3). *Ctenosciara lutea* is also colorful, but more or less completely orange, whereas *Ctenosciara hyalipennis* and *Ctenosciara exigua* are bright brownish just like many other Sciaridae. The male hypopygium conforms to the simple structure of the other European species, although the megasetae are more prominent. The new species also differs from all other European species in having a completely bare stM and CuA_2_ and a continuous, undivided tibial comb. The similar New Zealand species *Ctenosciara nigrostyla* (Mohrig, 1999) differs in having a less colourful hypopygium and a straighter gonostylus with a shorter apical tooth.

#### Etymology

The new species is named in honour of the founder of the Koenig museum in Bonn, Alexander Koenig (1885-1940).

#### Distribution

Besides the holotype, the species also appears to be present in New Zealand as confirmed by matching COI sequences on BOLD. We have not studied that material as of yet which is deposited in the Biodiversity Institute of Ontario, Canada.

#### Taxon discussion

After having seen the conspicuously looking *Ctenosciara* specimen from the museum's garden in Bonn for the first time, we were convinced of having discovered a new species native to Europe. The yielded COI sequence showed a 7% distance on BOLD to the nearest neighbour from Australia and convinced us furthermore of having an unknown species. After submitting the sequence to BOLD, it was shown to be identical to two other also newly submitted sequences from New Zealand, sharing the same BIN BOLD:ACP7364. Initially having consulted the key to the New Zealand species (Mohrig and Jaschhof 1999), we were inclined to identify our specimen as *Ctenosciara nigrostyla* Mohrig but a comparison with the type material in the collection of Werner Mohrig (Poseritz, Germany) revealed that the two species are in fact different. A worldwide DNA database like BOLD does not only help to distinguish new and cryptic species, but may also show distribution patterns. Usually more competitive continental species disperse on islands like New Zealand, but apparently the inverse case is also possible. It is a rare occurrence, that a species from the opposite end of the world is represented by a single specimen only and it is not yet clear, whether *Ctenosciara alexanderkoenigi* has a permanent population in Germany or if it was only introduced casually with plants or soil. Probably the species was recently introduced from the Australasian Region. If it was a permanent member of the European fauna, a striking species like this would likely have been found earlier.

### Ctenosciara exigua

Salmela & Vilkamaa 2005

HRCTE002-15

HRCTE003-15

#### Materials

- **Type status:** Other material. **Occurrence:** catalogNumber: ZFMK-TIS-2544881; recordedBy: Jukka Salmela; individualCount: 1; sex: male; lifeStage: adult; preparations: slide; otherCatalogNumbers: ZFMK-TIS-2544881; **Taxon:** scientificName: Ctenosciara exigua; genus: Ctenosciara; specificEpithet: exigua; scientificNameAuthorship: Salmela & Vilkamaa, 2005; **Location:** country: Finland; countryCode: FI; stateProvince: Lapland; municipality: Enontekiö; locality: Pikkuvaarat SW; verbatimElevation: 493; verbatimLatitude: 68°07'49.7'' N; verbatimLongitude: 24°02'39.8'' E; **Event:** samplingProtocol: Malaise trap; eventDate: 12/09/2014; endDayOfYear: 164; year: 2014; month: 9; day: 12; habitat: Poor sedge fen; **Record Level:** institutionCode: ZFMK
- **Type status:** Other material. **Occurrence:** catalogNumber: ZFMK-TIS-2544914; recordedBy: Jukka Salmela; individualCount: 1; sex: male; lifeStage: adult; preparations: slide; otherCatalogNumbers: ZFMK-TIS-2544914; **Taxon:** scientificName: Ctenosciara exigua; genus: Ctenosciara; specificEpithet: exigua; scientificNameAuthorship: Salmela & Vilkamaa, 2005; **Location:** country: Finland; countryCode: FI; stateProvince: Lapland; municipality: Savukoski; locality: Tyyroja; verbatimElevation: 251; verbatimLatitude: 68°09'00" N; verbatimLongitude: 28°33'00" E; **Event:** samplingProtocol: Malaise trap; eventDate: 05/08/2014; endDayOfYear: 217; year: 2014; month: 8; day: 5; habitat: alpine brook, stony; **Record Level:** institutionCode: ZFMK

#### Description

See Salmela and Vilkamaa (2005).

#### Diagnosis

*Ctenosciara exigua* was described based on several specimens from mires in Central Finland. It was differentiated from *Ctenosciara hyalipennis* by the evenly broad gonostyli with lacking megasetae at the dorsal side of the apical tooth, the smaller size and less setose CuA_2_. However, the most distinctive character, the shorter and roundish tegmen, was not mentioned. In *Ct. hyalipennis* the tegmen is much longer than wide, nearly triangular. In our material, the number of macrotrichia on CuA_2_ varies from 0 to 18 and the tibial comb was also found to be undivided in some specimens. There were usually differences to the original description in every specimen studied. As seen in Fig. 4, showing one of the barcoded individuals, the gonostylus is more tapered and has apical megasetae. One might argue, that our specimens do not exactly match with *Ct. exigua*. But as they are in the same manner clearly and more different from *Ct. hyalipennis*, we confidently identify them as *Ct. exigua*.

##### DNA barcoding result

BIN algorithm of BOLD indicates that the COI sequence of *Ctenosciara exigua* is not significantly different from that of *Ctenosciara hyalipennis* and belongs to the same BIN BOLD:AAH3983. More than 3000 specimens belonging to that same BIN are recorded from the South West and South East of Canada opposed by only roughly 1000 central-European records. Comparisons based on K2P distances within and between regions show closer affinities of *Ctenosciara exigua* to the Nearctic population than to the European (Suppl. material 4). Nonetheless, *Ct. exigua* is genetically identical with over 500 specimens from Canada, Germany and Norway (Suppl. material 3 & Suppl. material 6). The species complex of *Ct. hyalipennis* and *Ct. exigua* was first recorded for North America by Telfer et al. (2015).

#### Distribution

Since the original description from Finland, the species was mentioned again by Heller et al. (2009) from Sweden and therefore it appears to have a Northern European distribution. As the identification of this species is only possible by careful microsopic analysis of the male genitalia and because the most similar species, *Ctenosciara hyalipennis*, is one of the most common European Sciaridae, it may have been overlooked.

#### Taxon discussion

**Figure 6.**
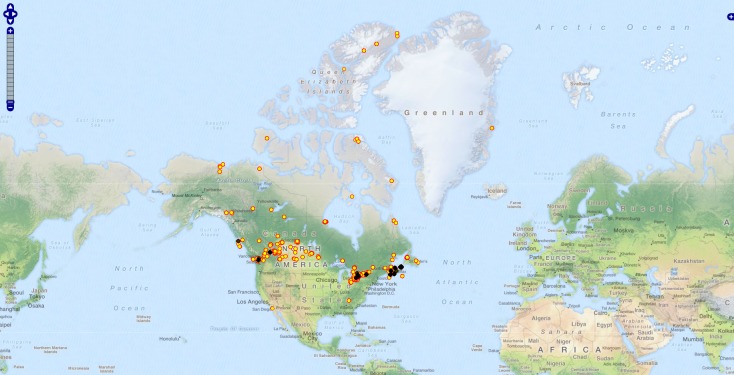
Occurence of Nearctic Sciaridae based on public availaible records on BOLD Suppl. material 1. Localities with Sciaridae (yellow dots) are even distributed, but BIN AAH3983 (black diamond) is restricted to West and East coast only.

The barcoding results coupled with the fact that *Ctenosciara hyalipennis* and *Ct. exigua* (in our understanding) are quite polymorphic raise the question *"* Is *Ctenosciara exigua* really a distinct species?" or "Is it only one variant of the former?". In Central Europe, *Ct. hyalipennis* shows two distinct morphs. The early spring form is larger and has clearly clavate gonostyles, whereas the summer variant is smaller, brighter and the shape of the gonostyles is just as parallel as in *Ctenosciara exigua*. The summer variant was treated as *Ctenosciara thiedei* in Thiede (1977), a *nomen nudum*, which was never officially published. The analysis of the COI did not show any significant differences between both seasonal morphs. The same situation could be present for the *Ctenosciara exigua/hyalipennis* complex. Recently Kurina et al. (2015) described a species of Mycetophilidae, which is not distinguishable genetically but only differs in the structure of male genitalia. Similarly is imaginable, that *Ct. hyalipennis* is a species that has only recently invaded from some other part of the world, then successfully occupied different ecological nices, but speciation has not yet progressed to a point where clear genetic differences have taken place. The bifid East-West distribution pattern in Canada (Fig. 6) might be be a reminiscence of two recent, parallel immigrations, which independently started from the eastern and western coasts. All those localities of *Ct. hyalipennis* are in the vicinity of typical entry points like harbors, airports and bigger cities with massive human activity or spreading already upstream. Geographic distribution of haplotypes underpin this assumption as Neartic population is gentically less diverse than European (Table 1). Keeping in mind, that most of the sciarid sequences (96%) discussed here originated from the Global Malaise Trap Program and thus sequences are only single strand generated, so some of the singletons may reflect in fact sequencing artefacts. Also earlier faunistic studies from North America (Johannsen 1912, Pettey 1918) do not mention this species and it was found neither in historical collections nor in younger material until 2000 (Mohrig pers. comm.). Further morphological, ecological and genetic analyses are needed to shed light on species concepts of *Ctenosciara hyalipennis* *sensu latu.* For the moment we propose to continue treating *Ctenosciara exigua* as a distinct species.

## Identification Keys

### Key to the European species of *Ctenosciara*

**Table:** 

1	Bright, orange-coloured species. Scape and pedicellus yellow.	2
–	Unicolorous, brownish species. Scape and pedicellus not brightened.	3
2	Body nearly unicolored, orange. CuA_2_ and stM with macrotrichia. Tibial comb on fore tibia divided.	*Ctenosciara lutea* (Meigen, 1804)
–	Body bicolored, thorax mainly orange, abdomen mainly brown. CuA_2_ and stM without macrotrichia. Tibial comb on fore tibia undivided.	*Ctenosciara alexanderkoenigi* **sp. n.**
3	Larger species, wing length > 2 mm. CuA_2_ with more than 10 macrotrichia. Front tibial comb strictly divided. Tegmen conical, longer than broad.	*Ctenosciara hyalipennis* (Meigen, 1804)
–	Smaller species, wing length ≤ 2 mm. CuA_2_ bare or with less than 8 macrotrichia. Apical comb on fore tibia undivided or unclearly divided. Tegmen roundish, not longer than broad.	*Ctenosciara exigua* Salmela & Vilkamaa, 2005

All currently known European species of *Ctenosciara* (Fig. 5) share the following characters:
Posterior wing veins (at least M_1_, M_2_, stM and CuA_1_) with macrotrichia Apical part of fore tibia with a bordered tibial comb, which is mostly divided Gonostylus simple, elongate, with apical tooth and a group of subapical megasetae.

## Discussion

The identification of small Diptera, especially of Sciaridae is based primarally on differences in the male genitalia. In this regard, high quality hand drawings of the hypopygium and the gonostylus at least have become the standard in the description of species. However there are two issues, which led us to depart from this tradition. The first is the sheer number of undescribed species that exist even in a well studied region such as Europe. Thorough drawings tend to be extremely time consuming and thus high quality stacked photos offer a quicker alternative. Secondly, very often, genitalia do not show clear differences at a first glance. DNA barcoding offers a means in which to unravel cryptic diversity and resolve species complexes that might go ignored and/or unnoticed. Interestingly coloration is proving more and more to be an effective tool for taxonomic differentiation in the Sciaridae despite being previously deemed unimportant. Conspecific variation in colouration appears to be minimal in the Sciaridae of the same DNA makeup. When species concepts have to be reconsidered, high quality photo documentation, as is standard in the BOLD project will be a useful method for evaluation. Furthermore a barcode assigned to a BIN will become indispensable to overcome the challenge of future biodiversity issues. We believe, that the rapid description of *Ctenosciara alexanderkoenigi* coupled with the BDJ reviewing system might be a robust and ground-breaking way to accelerate and stabilize taxonomy in the future.

## Supplementary Material

Supplementary material 1 Nearctic Sciaridae from BOLD Data type: data spread sheet localities
File: oo_73947.xlsx Björn Rulik & Kai Heller

Supplementary material 2 BOLD AAH3983 Alignment Data type: FASTA
File: oo_73950.fas Björn Rulik & Kai Heller

Supplementary material 3 K2P pairwise distances BIN AAH3983 Data type: distance matrix
File: oo_74309.xlsx Björn Rulik & Kai Heller

Supplementary material 4 K2P comparison BIN AAH3983 Data type: distance data
File: oo_74311.xlsx Björn Rulik & Kai Heller

Supplementary material 5 Haplotype information Data type: genomic
File: oo_73960.xlsx Björn Rulik & Kai Heller

Supplementary material 6 BOLD Taxon ID tree Data type: tree
File: oo_73957.pdf Björn Rulik & Kai Heller

XML Treatment for Ctenosciara alexanderkoenigi

XML Treatment for Ctenosciara exigua

## Figures and Tables

**Figure 2a.**
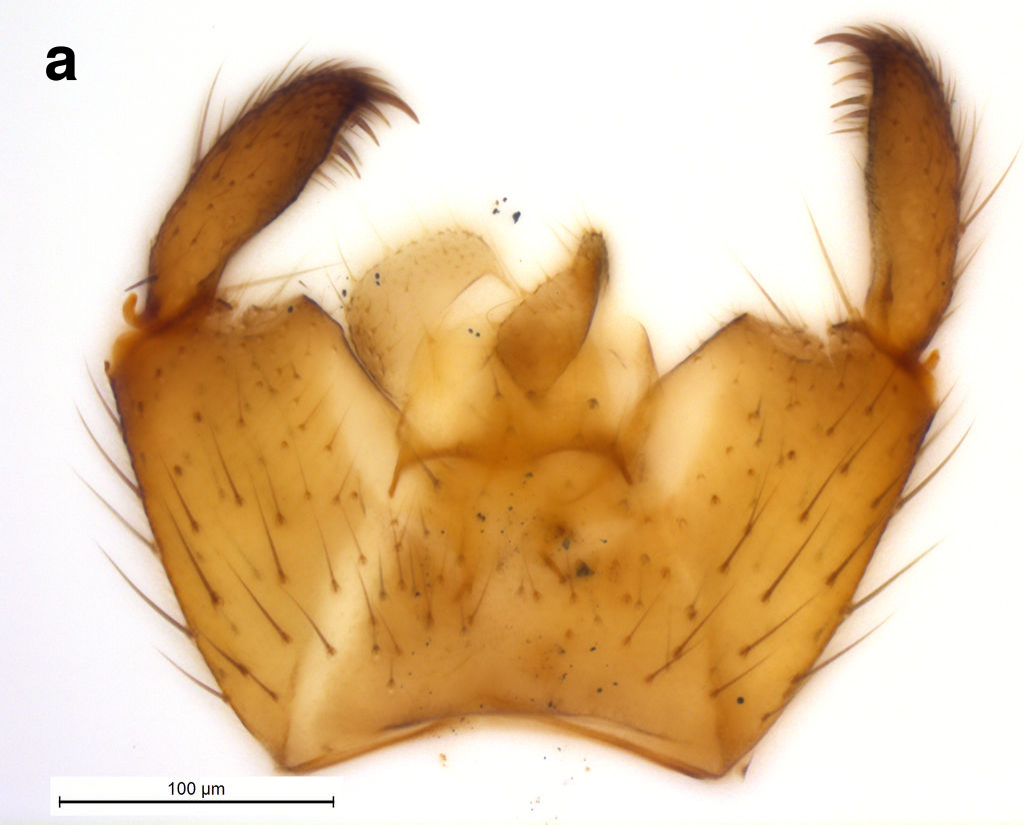
Hypopygium, scale 0.1 mm

**Figure 2b.**
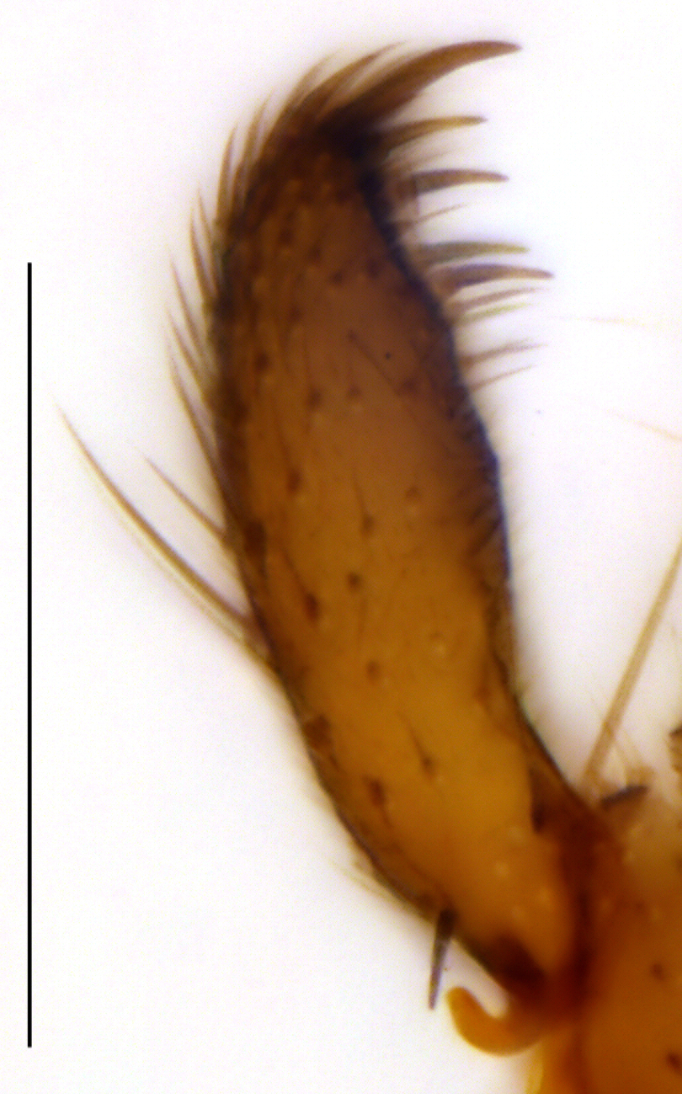
Gonostylus, scale 0.1 mm

**Figure 2c.**
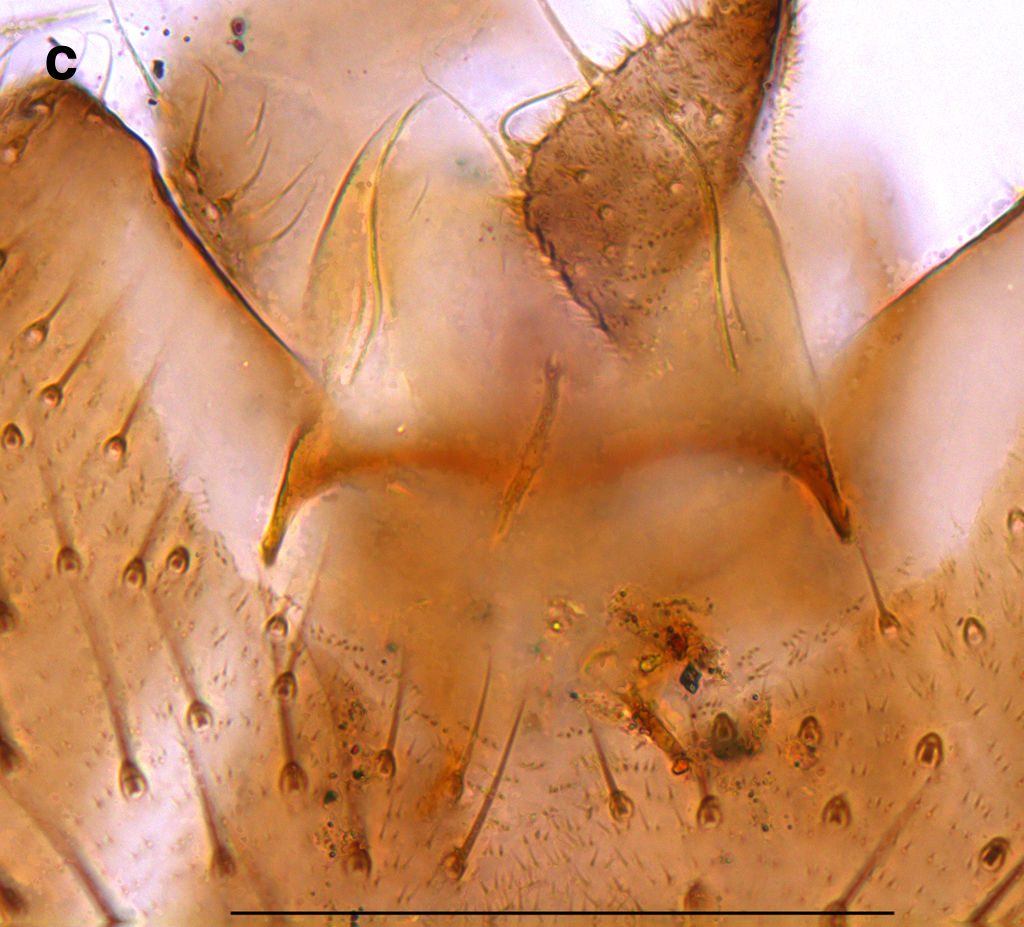
Tegmen, scale 0.1 mm

**Figure 2d.**
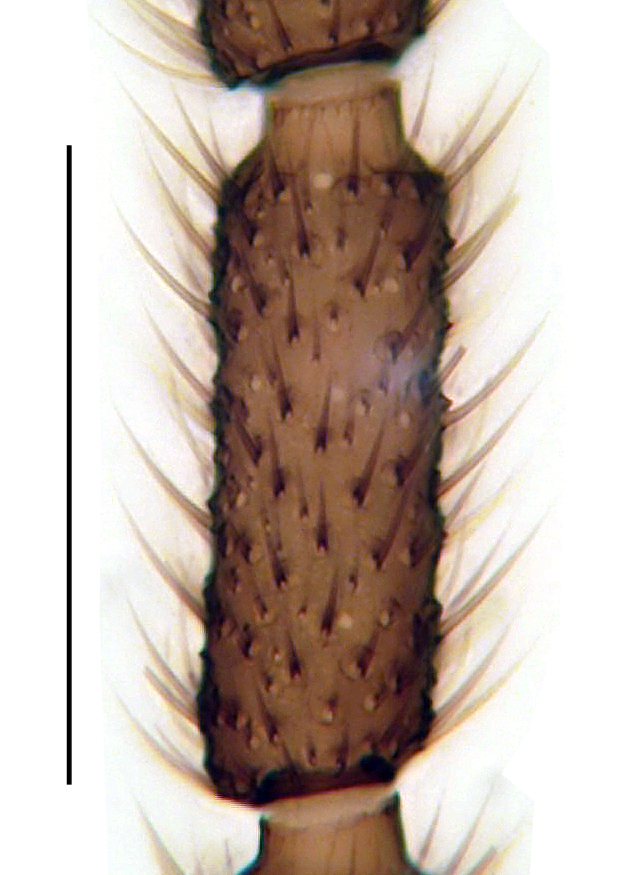
4^th^ antennal flagellomere, scale 0.1 mm

**Figure 2e.**
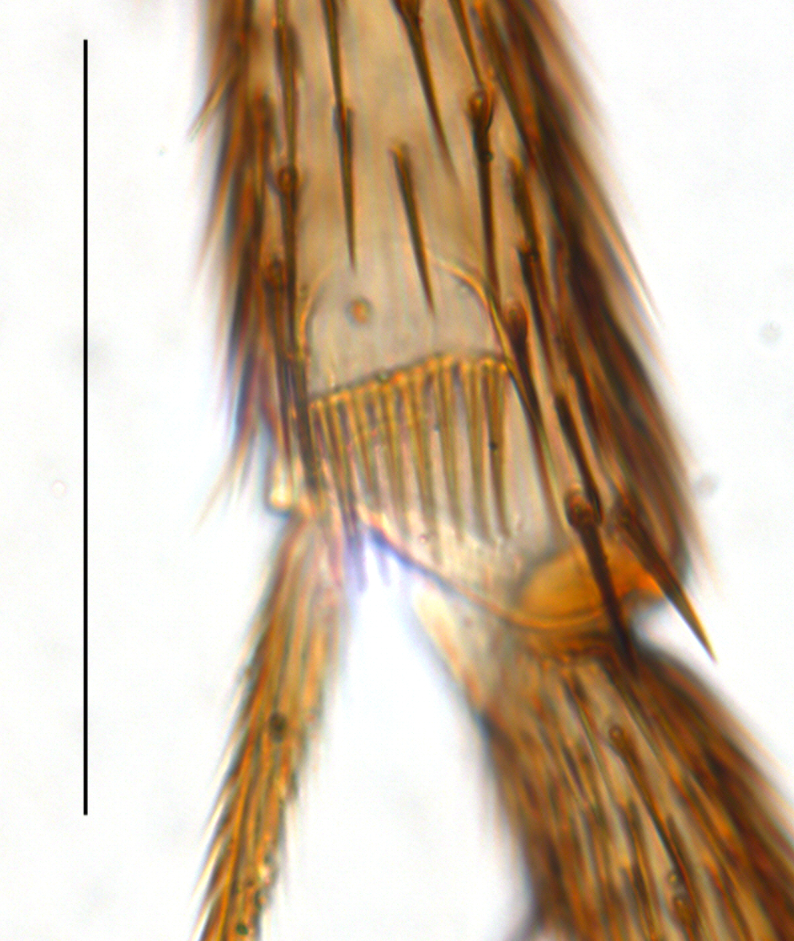
Tibial organ, scale 0.1 mm

**Figure 2f.**
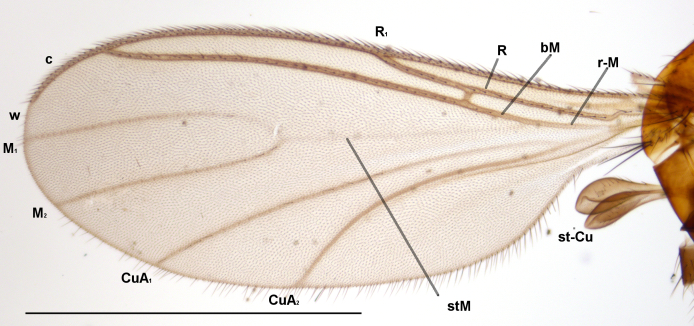
Wing, scale 1 mm

**Figure 4a.**
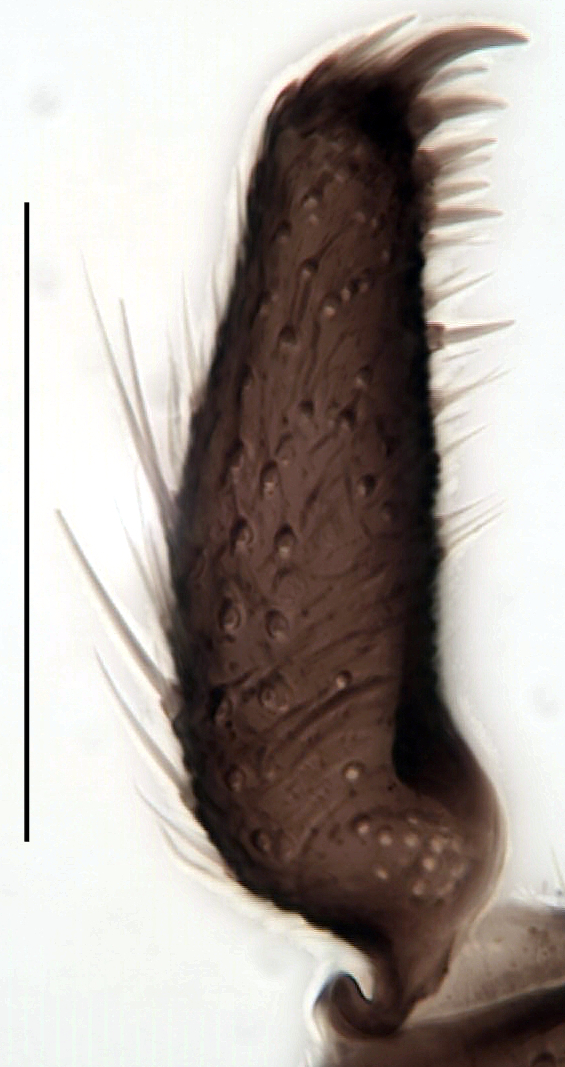
Gonostylus, scale 0.1 mm

**Figure 4b.**
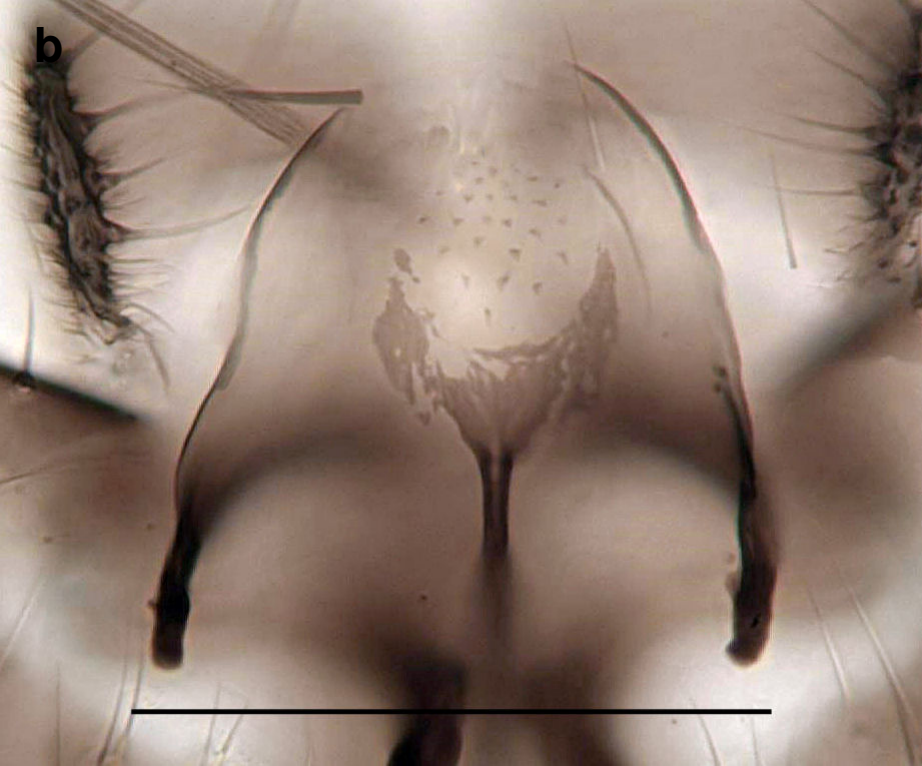
Tegmen, scale 0.1 mm

**Figure 4c.**
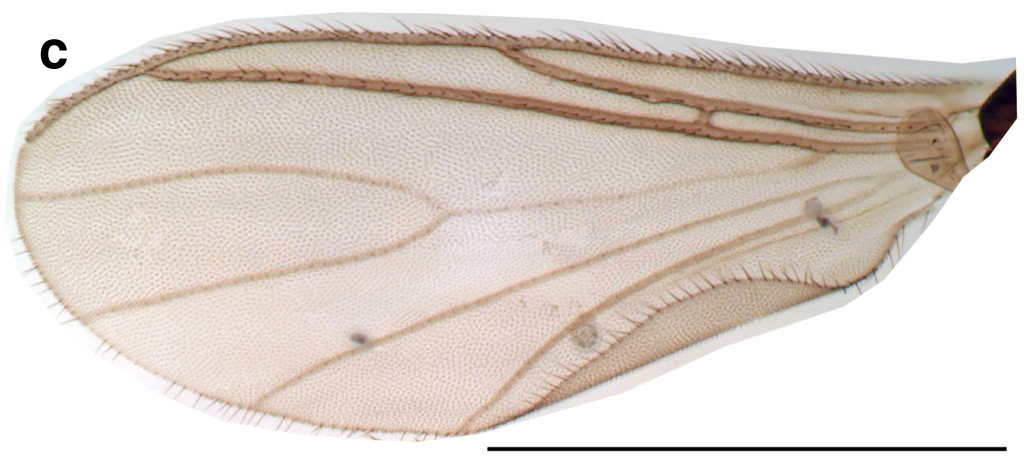
Wing, gonostylus, scale 1 mm

**Figure 4d.**
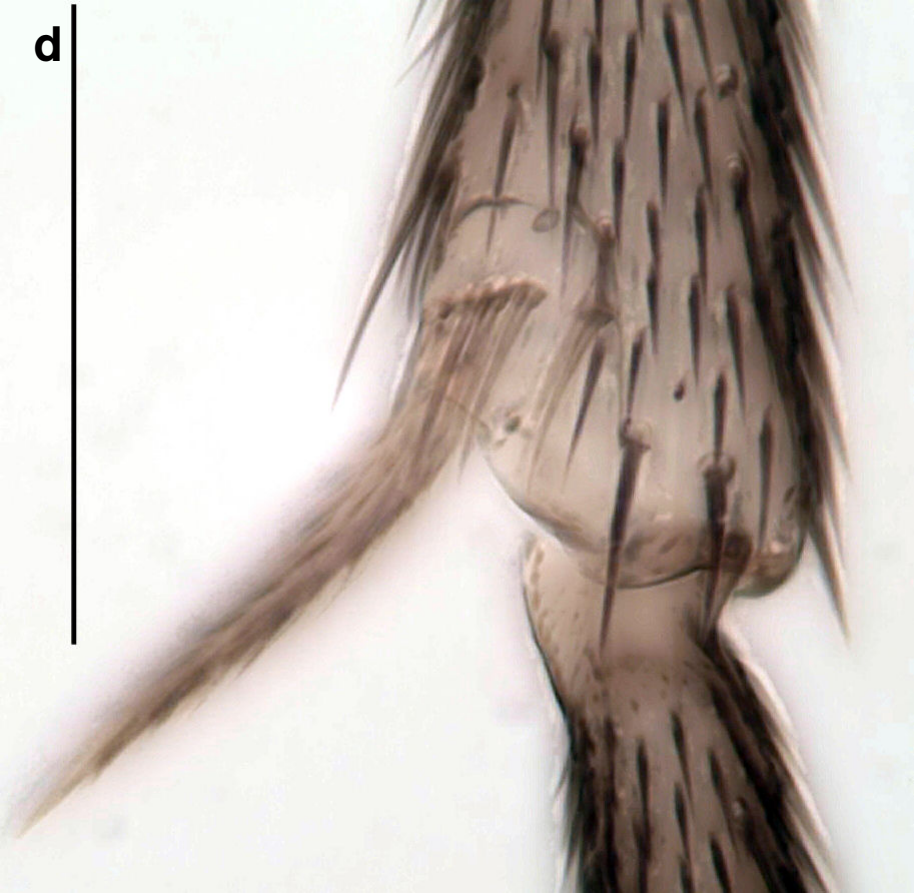
Tibial comb, scale 0.1 mm

**Figure 5a.**
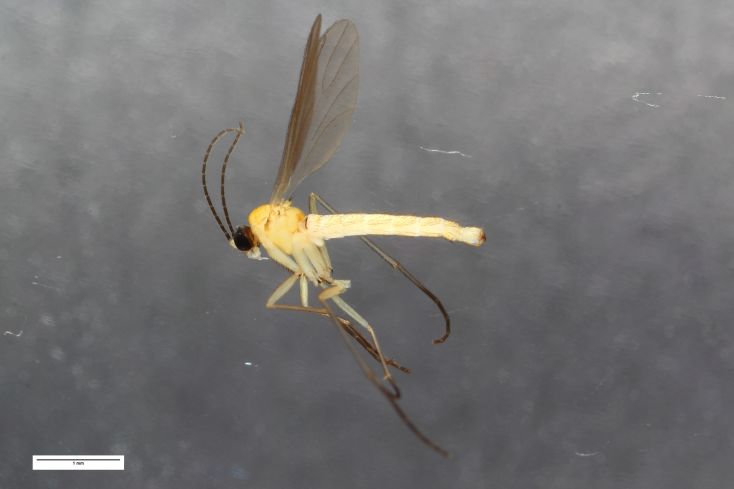
*Ctenosciara lutea*, scale 1 mm.

**Figure 5b.**
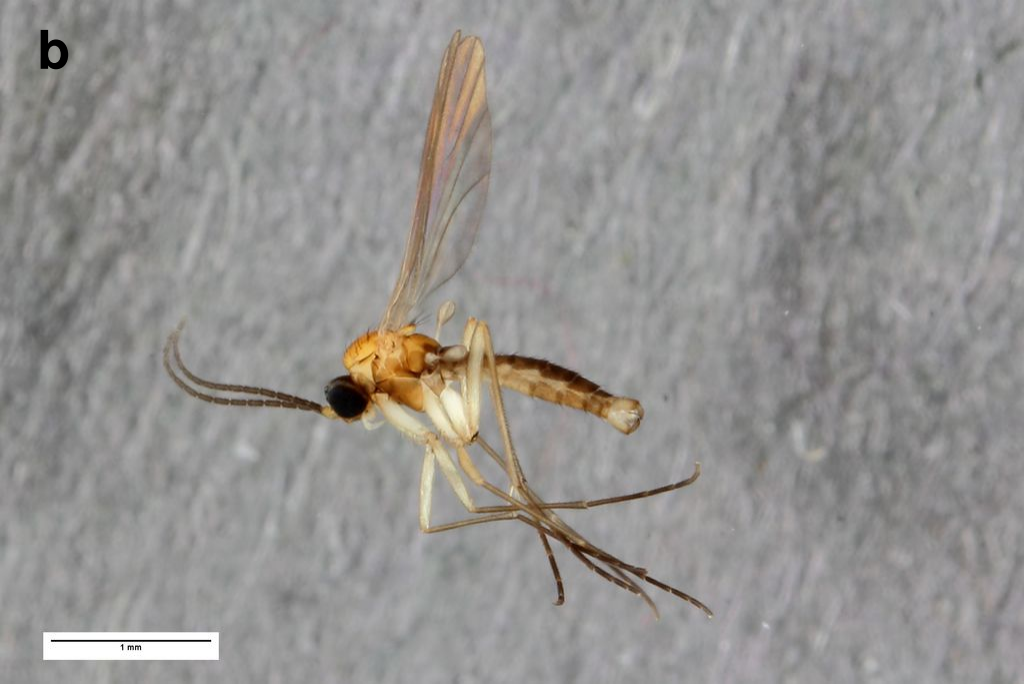
*Ctenosciara alexanderkoenigi* sp. nov., scale 1 mm.

**Figure 5c.**
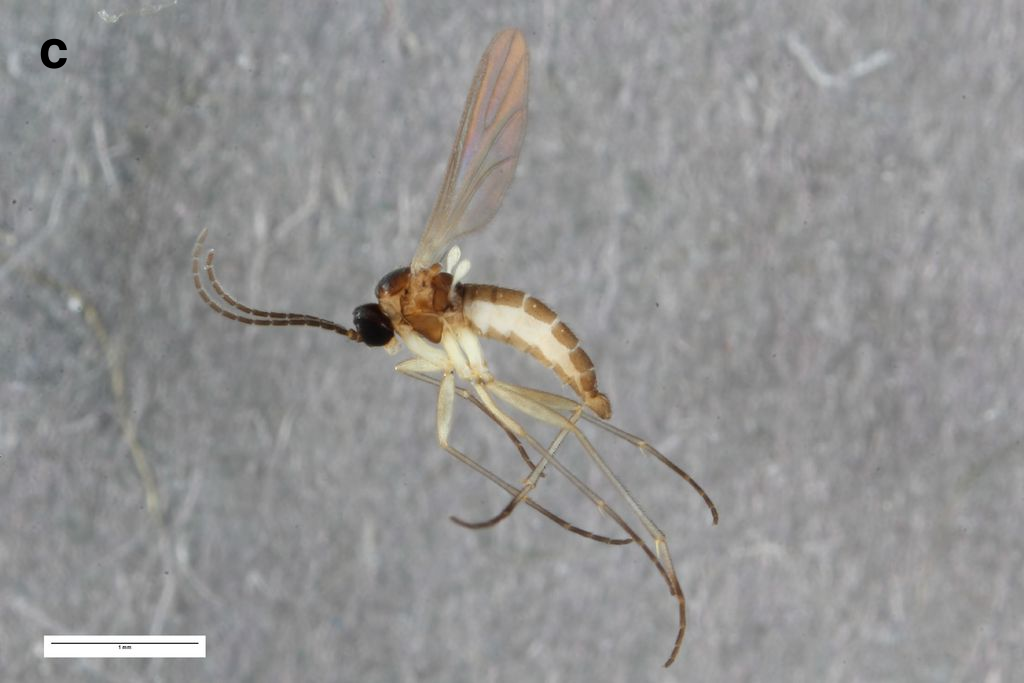
*Ctenosciara hyalipennis*, scale 1 mm.

**Figure 5d.**
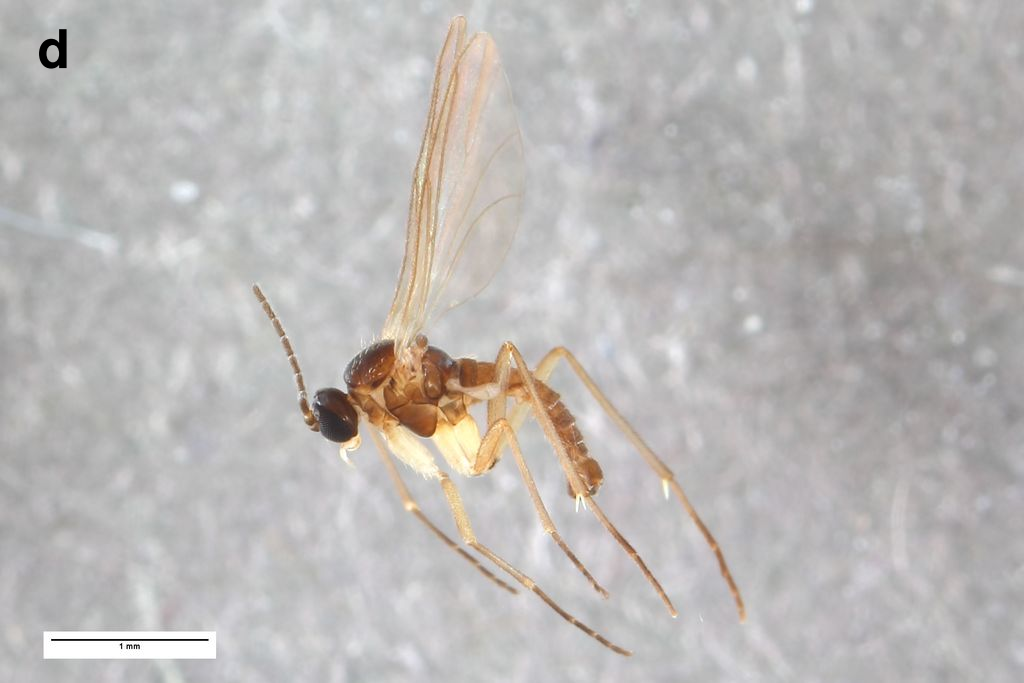
*Ctenosciara exigua*, scale 1mm

**Table 1.** Geographic haplotype distribution. ENEA = East Nearctic, FIN = Finland, * = *Ct. exigua*, GER = Germany, NOR = Norway, WNEA = West Nearctic, see also Suppl. material 5

**hap**	**1**	**2**	**3**	**4**	**5**	**6**	**7**	**8**	**9**	**10**	**11**	**12**	**13**	**14**	**15**	**16**	**17**	**18**	**19**	**20**
**ENEA**	2711	213	1	1																1
FIN*	2																			
**GER**	132	2			14	853	1	1	3	3	5	2	4	2	1	2	1	1	1	
**NOR**	1				2															
**WNEA**	8	79																		
**total**	2854	294	1	1	16	853	1	1	3	3	5	2	4	2	1	2	1	1	1	1
