# Supplementary material for: Ctenosciara alexanderkoenigi sp. n. (Diptera: Sciaridae), an exotic invader in Germany?
Source: Biodivers Data J. 2016 Apr 1;(4):e6460. doi: 10.3897/BDJ.4.e6460 (PMC4822059; doi:10.3897/BDJ.4.e6460)
Supplement: Supplementary material 6 — BOLD Taxon ID tree [file biodiversity_data_journal-4-e6460-s006.pdf]

# BOLD TaxonID Tree

| | | |
|---|---|---|
| Title | : | Tree Result - Search (4047 records) |
| Date | : | 20-January-2016 |
| Data Type | : | Nucleotide |
| Distance Model | : | Kimura 2 Parameter |
| Marker | : | COI-5P |
| Colourization | : | |
| Label | : | Process ID |
| Label | : | Country |
| Label | : | Barcode Cluster (BIN) |
| Filter | : | exclude records with stop codons |
| Filter | : | exclude contaminants |
| Sequence Count | : | 4047 |
| Species count | : | 2 |
| Genus count | : | 1 |
| Family count | : | 1 |
| Unidentified | : | 0 |
| BIN Count | : | 1 |

0.5 %

Ctenosciara hyalipennis|GMGRG1496-13|Germany|BOLD_AAH3983
Ctenosciara hyalipennis|GMGRF3850-13|Germany|BOLD_AAH3983
Ctenosciara hyalipennis|GMGRH1773-13|Germany|BOLD_AAH3983
Ctenosciara hyalipennis|GMGRG862-13|Germany|BOLD_AAH3983
Ctenosciara hyalipennis|GMGRF2980-13|Germany|BOLD_AAH3983
Ctenosciara hyalipennis|GMGRF1719-13|Germany|BOLD_AAH3983
Ctenosciara hyalipennis|GMGRF3162-13|Germany|BOLD_AAH3983
Ctenosciara hyalipennis|GMGRF805-13|Germany|BOLD_AAH3983
Ctenosciara hyalipennis|GMGRF804-13|Germany|BOLD_AAH3983
Ctenosciara hyalipennis|GMGRF720-13|Germany|BOLD_AAH3983
Ctenosciara hyalipennis|GMGRF530-13|Germany|BOLD_AAH3983
Ctenosciara hyalipennis|GMGRG2211-13|Germany|BOLD_AAH3983
Ctenosciara hyalipennis|GMGRF4806-13|Germany|BOLD_AAH3983
Ctenosciara hyalipennis|GMGRF3536-13|Germany|BOLD_AAH3983
Ctenosciara hyalipennis|GMGRF4633-13|Germany|BOLD_AAH3983
Ctenosciara hyalipennis|GMGRF302-13|Germany|BOLD_AAH3983
Ctenosciara hyalipennis|GMGRG880-13|Germany|BOLD_AAH3983
Ctenosciara hyalipennis|GMGRG1352-13|Germany|BOLD_AAH3983
Ctenosciara hyalipennis|GMGRF350-13|Germany|BOLD_AAH3983
Ctenosciara hyalipennis|GMGRF3464-13|Germany|BOLD_AAH3983
Ctenosciara hyalipennis|GMGRG2190-13|Germany|BOLD_AAH3983
Ctenosciara hyalipennis|GMGRF1135-13|Germany|BOLD_AAH3983
Ctenosciara hyalipennis|GMGRG742-13|Germany|BOLD_AAH3983
Ctenosciara hyalipennis|GMGRG2282-13|Germany|BOLD_AAH3983
Ctenosciara hyalipennis|GMGRA858-13|Germany|BOLD_AAH3983
Ctenosciara hyalipennis|GMGRA837-13|Germany|BOLD_AAH3983
Ctenosciara hyalipennis|GMGRA795-13|Germany|BOLD_AAH3983
Ctenosciara hyalipennis|GMGRB541-13|Germany|BOLD_AAH3983
Ctenosciara hyalipennis|GMGRB644-13|Germany|BOLD_AAH3983
Ctenosciara hyalipennis|GMGRB162-13|Germany|BOLD_AAH3983
Ctenosciara hyalipennis|GMGRB322-13|Germany|BOLD_AAH3983
Ctenosciara hyalipennis|GMGRA1229-13|Germany|BOLD_AAH3983
Ctenosciara hyalipennis|GMGRA1230-13|Germany|BOLD_AAH3983
Ctenosciara hyalipennis|GMGRA1232-13|Germany|BOLD_AAH3983
Ctenosciara hyalipennis|GMGRA1267-13|Germany|BOLD_AAH3983
Ctenosciara hyalipennis|GMGRA1190-13|Germany|BOLD_AAH3983
Ctenosciara hyalipennis|GMGRA1225-13|Germany|BOLD_AAH3983
Ctenosciara hyalipennis|GMGRA972-13|Germany|BOLD_AAH3983
Ctenosciara hyalipennis|GMGRA1014-13|Germany|BOLD_AAH3983
Ctenosciara hyalipennis|GMGRA449-13|Germany|BOLD_AAH3983
Ctenosciara hyalipennis|GMGRA883-13|Germany|BOLD_AAH3983
Ctenosciara hyalipennis|GMGRA447-13|Germany|BOLD_AAH3983
Ctenosciara hyalipennis|GMGRA448-13|Germany|BOLD_AAH3983
Ctenosciara hyalipennis|GMGRA391-13|Germany|BOLD_AAH3983
Ctenosciara hyalipennis|GMGRA396-13|Germany|BOLD_AAH3983
Ctenosciara hyalipennis|GMGRC366-13|Germany|BOLD_AAH3983
Ctenosciara hyalipennis|GMGRA373-13|Germany|BOLD_AAH3983
Ctenosciara hyalipennis|GMGRA189-13|Germany|BOLD_AAH3983
Ctenosciara hyalipennis|GMGRA081-13|Germany|BOLD_AAH3983
Ctenosciara hyalipennis|GMGRA202-13|Germany|BOLD_AAH3983
Ctenosciara hyalipennis|GMGRA219-13|Germany|BOLD_AAH3983
Ctenosciara hyalipennis|GMGRA224-13|Germany|BOLD_AAH3983
Ctenosciara hyalipennis|GMGRA232-13|Germany|BOLD_AAH3983
Ctenosciara hyalipennis|GMGRA234-13|Germany|BOLD_AAH3983
Ctenosciara hyalipennis|GMGRA238-13|Germany|BOLD_AAH3983
Ctenosciara hyalipennis|GMGRA251-13|Germany|BOLD_AAH3983
Ctenosciara hyalipennis|GMGRA254-13|Germany|BOLD_AAH3983
Ctenosciara hyalipennis|GMGRA273-13|Germany|BOLD_AAH3983
Ctenosciara hyalipennis|GMGRA293-13|Germany|BOLD_AAH3983
Ctenosciara hyalipennis|GMGRA296-13|Germany|BOLD_AAH3983
Ctenosciara hyalipennis|GMGRA298-13|Germany|BOLD_AAH3983
Ctenosciara hyalipennis|GMGRA465-13|Germany|BOLD_AAH3983
Ctenosciara hyalipennis|GMGRA496-13|Germany|BOLD_AAH3983
Ctenosciara hyalipennis|GMGRA526-13|Germany|BOLD_AAH3983
Ctenosciara hyalipennis|GMGRA547-13|Germany|BOLD_AAH3983
Ctenosciara hyalipennis|GMGRA568-13|Germany|BOLD_AAH3983
Ctenosciara hyalipennis|GMGRA569-13|Germany|BOLD_AAH3983
Ctenosciara hyalipennis|GMGRA574-13|Germany|BOLD_AAH3983
Ctenosciara hyalipennis|GMGRA666-13|Germany|BOLD_AAH3983
Ctenosciara hyalipennis|GMGRA670-13|Germany|BOLD_AAH3983
Ctenosciara hyalipennis|GMGRA671-13|Germany|BOLD_AAH3983
Ctenosciara hyalipennis|GMGRA675-13|Germany|BOLD_AAH3983
Ctenosciara hyalipennis|GMGRA691-13|Germany|BOLD_AAH3983
Ctenosciara hyalipennis|GMGRA693-13|Germany|BOLD_AAH3983
Ctenosciara hyalipennis|GMGRA715-13|Germany|BOLD_AAH3983
Ctenosciara hyalipennis|GMGRA717-13|Germany|BOLD_AAH3983
Ctenosciara hyalipennis|GMGRA742-13|Germany|BOLD_AAH3983
Ctenosciara hyalipennis|GMGRA870-13|Germany|BOLD_AAH3983
Ctenosciara hyalipennis|GMGRA876-13|Germany|BOLD_AAH3983
Ctenosciara hyalipennis|GMGRA848-13|Germany|BOLD_AAH3983
Ctenosciara hyalipennis|GMGRA879-13|Germany|BOLD_AAH3983
Ctenosciara hyalipennis|GMGRA878-13|Germany|BOLD_AAH3983
Ctenosciara hyalipennis|GMGRA903-13|Germany|BOLD_AAH3983
Ctenosciara hyalipennis|GMGRA960-13|Germany|BOLD_AAH3983
Ctenosciara hyalipennis|GMGRA1250-13|Germany|BOLD_AAH3983
Ctenosciara hyalipennis|GMGRA1038-13|Germany|BOLD_AAH3983
Ctenosciara hyalipennis|GMGRA1299-13|Germany|BOLD_AAH3983
Ctenosciara hyalipennis|GMGRA793-13|Germany|BOLD_AAH3983
Ctenosciara hyalipennis|GMGRA1332-13|Germany|BOLD_AAH3983
Ctenosciara hyalipennis|GMGRB119-13|Germany|BOLD_AAH3983
Ctenosciara hyalipennis|GMGRB1166-13|Germany|BOLD_AAH3983
Ctenosciara hyalipennis|GMGRA184-13|Germany|BOLD_AAH3983
Ctenosciara hyalipennis|GMGRA185-13|Germany|BOLD_AAH3983
Ctenosciara hyalipennis|GMGRA150-13|Germany|BOLD_AAH3983
Ctenosciara hyalipennis|GMGRA160-13|Germany|BOLD_AAH3983
Ctenosciara hyalipennis|GMGRA142-13|Germany|BOLD_AAH3983
Ctenosciara hyalipennis|GMGRA149-13|Germany|BOLD_AAH3983
Ctenosciara hyalipennis|GMGRA117-13|Germany|BOLD_AAH3983
Ctenosciara hyalipennis|GMGRA128-13|Germany|BOLD_AAH3983

Ctenosciara hyalipennis|GMGRA149-13|Germany|BOLD_AAH3983
Ctenosciara hyalipennis|GMGRA117-13|Germany|BOLD_AAH3983
Ctenosciara hyalipennis|GMGRA128-13|Germany|BOLD_AAH3983
Ctenosciara hyalipennis|GMGRA094-13|Germany|BOLD_AAH3983
Ctenosciara hyalipennis|GMGRA108-13|Germany|BOLD_AAH3983
Ctenosciara hyalipennis|GMGRA055-13|Germany|BOLD_AAH3983
Ctenosciara hyalipennis|GMGRA083-13|Germany|BOLD_AAH3983
Ctenosciara hyalipennis|GMGRA040-13|Germany|BOLD_AAH3983
Ctenosciara hyalipennis|GMGRA052-13|Germany|BOLD_AAH3983
Ctenosciara hyalipennis|GMGRB595-13|Germany|BOLD_AAH3983
Ctenosciara hyalipennis|GMGRB661-13|Germany|BOLD_AAH3983
Ctenosciara hyalipennis|GMGRA962-13|Germany|BOLD_AAH3983
Ctenosciara hyalipennis|GMGRA1013-13|Germany|BOLD_AAH3983
Ctenosciara hyalipennis|GMGRA875-13|Germany|BOLD_AAH3983
Ctenosciara hyalipennis|GMGRA937-13|Germany|BOLD_AAH3983
Ctenosciara hyalipennis|GMGRA287-13|Germany|BOLD_AAH3983
Ctenosciara hyalipennis|GMGRA808-13|Germany|BOLD_AAH3983
Ctenosciara hyalipennis|GMGRA212-13|Germany|BOLD_AAH3983
Ctenosciara hyalipennis|GMGRA216-13|Germany|BOLD_AAH3983
Ctenosciara hyalipennis|GMGRB539-13|Germany|BOLD_AAH3983
Ctenosciara hyalipennis|GMGRB590-13|Germany|BOLD_AAH3983
Ctenosciara hyalipennis|GMGRA1308-13|Germany|BOLD_AAH3983
Ctenosciara hyalipennis|GMGRB355-13|Germany|BOLD_AAH3983
Ctenosciara hyalipennis|GMGRA200-13|Germany|BOLD_AAH3983
Ctenosciara hyalipennis|GMGRA286-13|Germany|BOLD_AAH3983
Ctenosciara hyalipennis|GMGRA532-13|Germany|BOLD_AAH3983
Ctenosciara hyalipennis|GMGRA792-13|Germany|BOLD_AAH3983
Ctenosciara hyalipennis|GMGRA806-13|Germany|BOLD_AAH3983
Ctenosciara hyalipennis|GMGRA867-13|Germany|BOLD_AAH3983
Ctenosciara hyalipennis|GMGRA884-13|Germany|BOLD_AAH3983
Ctenosciara hyalipennis|GMGRA1324-13|Germany|BOLD_AAH3983
Ctenosciara hyalipennis|GMGRA190-13|Germany|BOLD_AAH3983
Ctenosciara hyalipennis|GMGRB208-13|Germany|BOLD_AAH3983
Ctenosciara hyalipennis|GMGRB377-13|Germany|BOLD_AAH3983
Ctenosciara hyalipennis|GMGRB1172-13|Germany|BOLD_AAH3983
Ctenosciara hyalipennis|GMGRC335-13|Germany|BOLD_AAH3983
Ctenosciara hyalipennis|GMGRA1285-13|Germany|BOLD_AAH3983
Ctenosciara hyalipennis|GMGRA1249-13|Germany|BOLD_AAH3983
Ctenosciara hyalipennis|GMGRA1237-13|Germany|BOLD_AAH3983
Ctenosciara hyalipennis|GMGRA1180-13|Germany|BOLD_AAH3983
Ctenosciara hyalipennis|GMGRA424-13|Germany|BOLD_AAH3983
Ctenosciara hyalipennis|GMGRB812-13|Germany|BOLD_AAH3983
Ctenosciara hyalipennis|GMGRH241-13|Germany|BOLD_AAH3983
Ctenosciara hyalipennis|GMGRB626-13|Germany|BOLD_AAH3983
Ctenosciara hyalipennis|GMGRB650-13|Germany|BOLD_AAH3983
Ctenosciara hyalipennis|GMGRA1284-13|Germany|BOLD_AAH3983
Ctenosciara hyalipennis|GMGRA1348-13|Germany|BOLD_AAH3983
Ctenosciara hyalipennis|GMGRA1037-13|Germany|BOLD_AAH3983
Ctenosciara hyalipennis|GMGRA1273-13|Germany|BOLD_AAH3983
Ctenosciara hyalipennis|GMGRB577-13|Germany|BOLD_AAH3983
Ctenosciara hyalipennis|GMGRB620-13|Germany|BOLD_AAH3983
Ctenosciara hyalipennis|GMGRB152-13|Germany|BOLD_AAH3983
Ctenosciara hyalipennis|GMGRB244-13|Germany|BOLD_AAH3983
Ctenosciara hyalipennis|GMGRA852-13|Germany|BOLD_AAH3983
Ctenosciara hyalipennis|GMGRA963-13|Germany|BOLD_AAH3983
Ctenosciara hyalipennis|GMGRA633-13|Germany|BOLD_AAH3983
Ctenosciara hyalipennis|GMGRA794-13|Germany|BOLD_AAH3983
Ctenosciara hyalipennis|GMGRA1337-13|Germany|BOLD_AAH3983
Ctenosciara hyalipennis|GMGRB315-13|Germany|BOLD_AAH3983
Ctenosciara hyalipennis|GMGRA636-13|Germany|BOLD_AAH3983
Ctenosciara hyalipennis|GMGRA680-13|Germany|BOLD_AAH3983
Ctenosciara hyalipennis|GMGRA846-13|Germany|BOLD_AAH3983
Ctenosciara hyalipennis|GMGRA1290-13|Germany|BOLD_AAH3983
Ctenosciara hyalipennis|GMGRB510-13|Germany|BOLD_AAH3983
Ctenosciara hyalipennis|GMGRB1189-13|Germany|BOLD_AAH3983
Ctenosciara hyalipennis|GMGRA027-13|Germany|BOLD_AAH3983
Ctenosciara hyalipennis|GMGRB760-13|Germany|BOLD_AAH3983
Ctenosciara hyalipennis|GMGRB761-13|Germany|BOLD_AAH3983
Ctenosciara hyalipennis|GMGRB755-13|Germany|BOLD_AAH3983
Ctenosciara hyalipennis|GMGRB758-13|Germany|BOLD_AAH3983
Ctenosciara hyalipennis|GMGRB744-13|Germany|BOLD_AAH3983
Ctenosciara hyalipennis|GMGRB747-13|Germany|BOLD_AAH3983
Ctenosciara hyalipennis|GMGRB706-13|Germany|BOLD_AAH3983
Ctenosciara hyalipennis|GMGRB708-13|Germany|BOLD_AAH3983
Ctenosciara hyalipennis|GMGRB678-13|Germany|BOLD_AAH3983
Ctenosciara hyalipennis|GMGRB682-13|Germany|BOLD_AAH3983
Ctenosciara hyalipennis|GMGRB813-13|Germany|BOLD_AAH3983
Ctenosciara hyalipennis|GMGRG886-13|Germany|BOLD_AAH3983
Ctenosciara hyalipennis|GMGRG4752-13|Germany|BOLD_AAH3983
Ctenosciara hyalipennis|GMGRB675-13|Germany|BOLD_AAH3983
Ctenosciara hyalipennis|GMGRB674-13|Germany|BOLD_AAH3983
Ctenosciara hyalipennis|GMGRB627-13|Germany|BOLD_AAH3983
Ctenosciara hyalipennis|GMGRA600-13|Germany|BOLD_AAH3983
Ctenosciara hyalipennis|GMGRA188-13|Germany|BOLD_AAH3983
Ctenosciara hyalipennis|GMGRB641-13|Germany|BOLD_AAH3983
Ctenosciara hyalipennis|GMGRB342-13|Germany|BOLD_AAH3983
Ctenosciara hyalipennis|GMGRA1068-13|Germany|BOLD_AAH3983
Ctenosciara hyalipennis|GMGRA1029-13|Germany|BOLD_AAH3983
Ctenosciara hyalipennis|GMGRA260-13|Germany|BOLD_AAH3983
Ctenosciara hyalipennis|GMGRB591-13|Germany|BOLD_AAH3983
Ctenosciara hyalipennis|GMGRF198-13|Germany|BOLD_AAH3983
Ctenosciara hyalipennis|GMGRB521-13|Germany|BOLD_AAH3983
Ctenosciara hyalipennis|GMGRB567-13|Germany|BOLD_AAH3983
Ctenosciara hyalipennis|GMGRB274-13|Germany|BOLD_AAH3983
Ctenosciara hyalipennis|GMGRB512-13|Germany|BOLD_AAH3983
Ctenosciara hyalipennis|GMGRA1344-13|Germany|BOLD_AAH3983
Ctenosciara hyalipennis|GMGRA1354-13|Germany|BOLD_AAH3983
Ctenosciara hyalipennis|GMGRA1218-13|Germany|BOLD_AAH3983
Ctenosciara hyalipennis|GMGRA1235-13|Germany|BOLD_AAH3983
Ctenosciara hyalipennis|GMGRA1053-13|Germany|BOLD_AAH3983
Ctenosciara hyalipennis|GMGRA1057-13|Germany|BOLD_AAH3983
Ctenosciara hyalipennis|GMGRA1063-13|Germany|BOLD_AAH3983

[illegible]
Ctenosciara hyalipennis|GMGRA1053-13|Germany|BOLD_AAH3983
Ctenosciara hyalipennis|GMGRA1057-13|Germany|BOLD_AAH3983
Ctenosciara hyalipennis|GMGRA1063-13|Germany|BOLD_AAH3983
Ctenosciara hyalipennis|GMGRA1187-13|Germany|BOLD_AAH3983
Ctenosciara hyalipennis|GMGRA1019-13|Germany|BOLD_AAH3983
Ctenosciara hyalipennis|GMGRA729-13|Germany|BOLD_AAH3983
Ctenosciara hyalipennis|GMGRA604-13|Germany|BOLD_AAH3983
Ctenosciara hyalipennis|GMGRA710-13|Germany|BOLD_AAH3983
Ctenosciara hyalipennis|GMGRA1343-13|Germany|BOLD_AAH3983
Ctenosciara hyalipennis|GMGRB455-13|Germany|BOLD_AAH3983
Ctenosciara hyalipennis|GMGRB476-13|Germany|BOLD_AAH3983
Ctenosciara hyalipennis|GMGRB446-13|Germany|BOLD_AAH3983
Ctenosciara hyalipennis|GMGRB447-13|Germany|BOLD_AAH3983
Ctenosciara hyalipennis|GMGRB432-13|Germany|BOLD_AAH3983
Ctenosciara hyalipennis|GMGRB434-13|Germany|BOLD_AAH3983
Ctenosciara hyalipennis|GMGRB431-13|Germany|BOLD_AAH3983
Ctenosciara hyalipennis|GMGRB430-13|Germany|BOLD_AAH3983
Ctenosciara hyalipennis|GMGRB422-13|Germany|BOLD_AAH3983
Ctenosciara hyalipennis|GMGRB413-13|Germany|BOLD_AAH3983
Ctenosciara hyalipennis|GMGRB392-13|Germany|BOLD_AAH3983
Ctenosciara hyalipennis|GMGRB388-13|Germany|BOLD_AAH3983
Ctenosciara hyalipennis|GMGRB1181-13|Germany|BOLD_AAH3983
Ctenosciara hyalipennis|GMGRC995-13|Germany|BOLD_AAH3983
Ctenosciara hyalipennis|GMGRA1247-13|Germany|BOLD_AAH3983
Ctenosciara hyalipennis|GMGRB666-13|Germany|BOLD_AAH3983
Ctenosciara hyalipennis|GMGRA1245-13|Germany|BOLD_AAH3983
Ctenosciara hyalipennis|GMGRA1240-13|Germany|BOLD_AAH3983
Ctenosciara hyalipennis|GMGRA1223-13|Germany|BOLD_AAH3983
Ctenosciara hyalipennis|GMGRA1203-13|Germany|BOLD_AAH3983
Ctenosciara hyalipennis|GMGRF044-13|Germany|BOLD_AAH3983
Ctenosciara hyalipennis|GMGRF008-13|Germany|BOLD_AAH3983
Ctenosciara hyalipennis|GMGRB719-13|Germany|BOLD_AAH3983
Ctenosciara hyalipennis|GMGRG4734-13|Germany|BOLD_AAH3983
Ctenosciara hyalipennis|GMGRB494-13|Germany|BOLD_AAH3983
Ctenosciara hyalipennis|GMGRB429-13|Germany|BOLD_AAH3983
Ctenosciara hyalipennis|GMGRA583-13|Germany|BOLD_AAH3983
Ctenosciara hyalipennis|GMGRA585-13|Germany|BOLD_AAH3983
Ctenosciara hyalipennis|GMGRA1266-13|Germany|BOLD_AAH3983
Ctenosciara hyalipennis|GMGRF084-13|Germany|BOLD_AAH3983
Ctenosciara hyalipennis|GMGRF100-13|Germany|BOLD_AAH3983
Ctenosciara hyalipennis|GMGRF1776-13|Germany|BOLD_AAH3983
Ctenosciara hyalipennis|GMGRF214-13|Germany|BOLD_AAH3983
Ctenosciara hyalipennis|GMGRF207-13|Germany|BOLD_AAH3983
Ctenosciara hyalipennis|GMGRF208-13|Germany|BOLD_AAH3983
Ctenosciara hyalipennis|GMGRG1529-13|Germany|BOLD_AAH3983
Ctenosciara hyalipennis|GMGRG1624-13|Germany|BOLD_AAH3983
Ctenosciara hyalipennis|GMGRG1722-13|Germany|BOLD_AAH3983
Ctenosciara hyalipennis|GMGRG803-13|Germany|BOLD_AAH3983
Ctenosciara hyalipennis|GMGRG720-13|Germany|BOLD_AAH3983
Ctenosciara hyalipennis|GMGRG718-13|Germany|BOLD_AAH3983
Ctenosciara hyalipennis|GMGRF3067-13|Germany|BOLD_AAH3983
Ctenosciara hyalipennis|GMGRF829-13|Germany|BOLD_AAH3983
Ctenosciara hyalipennis|GMGRG559-13|Germany|BOLD_AAH3983
Ctenosciara hyalipennis|GMGRG286-13|Germany|BOLD_AAH3983
Ctenosciara hyalipennis|GMGRG404-13|Germany|BOLD_AAH3983
Ctenosciara hyalipennis|GMGRG543-13|Germany|BOLD_AAH3983
Ctenosciara hyalipennis|GMGRG431-13|Germany|BOLD_AAH3983
Ctenosciara hyalipennis|GMGRG323-13|Germany|BOLD_AAH3983
Ctenosciara hyalipennis|GMGRG311-13|Germany|BOLD_AAH3983
Ctenosciara hyalipennis|GMGRG183-13|Germany|BOLD_AAH3983
Ctenosciara hyalipennis|GMGRF4885-13|Germany|BOLD_AAH3983
Ctenosciara hyalipennis|GMGRF3803-13|Germany|BOLD_AAH3983
Ctenosciara hyalipennis|GMGRF2595-13|Germany|BOLD_AAH3983
Ctenosciara hyalipennis|GMGRF2568-13|Germany|BOLD_AAH3983
Ctenosciara hyalipennis|GMGRF1822-13|Germany|BOLD_AAH3983
Ctenosciara hyalipennis|GMGRF3040-13|Germany|BOLD_AAH3983
Ctenosciara hyalipennis|GMGRG3501-13|Germany|BOLD_AAH3983
Ctenosciara hyalipennis|GMGRF4889-13|Germany|BOLD_AAH3983
Ctenosciara hyalipennis|GMGRG322-13|Germany|BOLD_AAH3983
Ctenosciara hyalipennis|GMGRG324-13|Germany|BOLD_AAH3983
Ctenosciara hyalipennis|GMGRG327-13|Germany|BOLD_AAH3983
Ctenosciara hyalipennis|GMGRG336-13|Germany|BOLD_AAH3983
Ctenosciara hyalipennis|GMGRG186-13|Germany|BOLD_AAH3983
Ctenosciara hyalipennis|GMGRG316-13|Germany|BOLD_AAH3983
Ctenosciara hyalipennis|GMGRG360-13|Germany|BOLD_AAH3983
Ctenosciara hyalipennis|GMGRG372-13|Germany|BOLD_AAH3983
Ctenosciara hyalipennis|GMGRG500-13|Germany|BOLD_AAH3983
Ctenosciara hyalipennis|GMGRG501-13|Germany|BOLD_AAH3983
Ctenosciara hyalipennis|GMGRG511-13|Germany|BOLD_AAH3983
Ctenosciara hyalipennis|GMGRG556-13|Germany|BOLD_AAH3983
Ctenosciara hyalipennis|GMGRG293-13|Germany|BOLD_AAH3983
Ctenosciara hyalipennis|GMGRG174-13|Germany|BOLD_AAH3983
Ctenosciara hyalipennis|GMGRG088-13|Germany|BOLD_AAH3983
Ctenosciara hyalipennis|GMGRG060-13|Germany|BOLD_AAH3983
Ctenosciara hyalipennis|GMGRF393-13|Germany|BOLD_AAH3983
Ctenosciara hyalipennis|GMGRF3886-13|Germany|BOLD_AAH3983
Ctenosciara hyalipennis|GMGRG4829-13|Germany|BOLD_AAH3983
Ctenosciara hyalipennis|GMGRF3873-13|Germany|BOLD_AAH3983
Ctenosciara hyalipennis|GMGRF3885-13|Germany|BOLD_AAH3983
Ctenosciara hyalipennis|GMGRF2585-13|Germany|BOLD_AAH3983
Ctenosciara hyalipennis|GMGRF3741-13|Germany|BOLD_AAH3983
Ctenosciara hyalipennis|GMGRF2146-13|Germany|BOLD_AAH3983
Ctenosciara hyalipennis|GMGRF2174-13|Germany|BOLD_AAH3983
Ctenosciara hyalipennis|GMGRF2138-13|Germany|BOLD_AAH3983
Ctenosciara hyalipennis|GMGRF1781-13|Germany|BOLD_AAH3983
Ctenosciara hyalipennis|GMGRF1451-13|Germany|BOLD_AAH3983
Ctenosciara hyalipennis|GMGRF1433-13|Germany|BOLD_AAH3983
Ctenosciara hyalipennis|GMGRF1148-13|Germany|BOLD_AAH3983
Ctenosciara hyalipennis|GMGRG3346-13|Germany|BOLD_AAH3983
Ctenosciara hyalipennis|GMGRG3360-13|Germany|BOLD_AAH3983
Ctenosciara hyalipennis|GMGRG1265-13|Germany|BOLD_AAH3983
[illegible]

Ctenosciara hyalipennis|GMGRG3346-13|Germany|BOLD_AAH3983
Ctenosciara hyalipennis|GMGRG3360-13|Germany|BOLD_AAH3983
Ctenosciara hyalipennis|GMGRG1265-13|Germany|BOLD_AAH3983
Ctenosciara hyalipennis|GMGRG1569-13|Germany|BOLD_AAH3983
Ctenosciara hyalipennis|GMGRF3765-13|Germany|BOLD_AAH3983
Ctenosciara hyalipennis|GMGRF5699-13|Germany|BOLD_AAH3983
Ctenosciara hyalipennis|GMGRF3183-13|Germany|BOLD_AAH3983
Ctenosciara hyalipennis|GMGRF3751-13|Germany|BOLD_AAH3983
Ctenosciara hyalipennis|GMGRG1629-13|Germany|BOLD_AAH3983
Ctenosciara hyalipennis|GMGRG1878-13|Germany|BOLD_AAH3983
Ctenosciara hyalipennis|GMGRG3592-13|Germany|BOLD_AAH3983
Ctenosciara hyalipennis|GMGRF3156-13|Germany|BOLD_AAH3983
Ctenosciara hyalipennis|GMGRF3096-13|Germany|BOLD_AAH3983
Ctenosciara hyalipennis|GMGRF3061-13|Germany|BOLD_AAH3983
Ctenosciara hyalipennis|GMGRF2069-13|Germany|BOLD_AAH3983
Ctenosciara hyalipennis|GMGRG2223-13|Germany|BOLD_AAH3983
Ctenosciara hyalipennis|GMGRG2304-13|Germany|BOLD_AAH3983
Ctenosciara hyalipennis|GMGRG2750-13|Germany|BOLD_AAH3983
Ctenosciara hyalipennis|GMGRG2909-13|Germany|BOLD_AAH3983
Ctenosciara hyalipennis|GMGRG2199-13|Germany|BOLD_AAH3983
Ctenosciara hyalipennis|GMGRG2204-13|Germany|BOLD_AAH3983
Ctenosciara hyalipennis|GMGRG2166-13|Germany|BOLD_AAH3983
Ctenosciara hyalipennis|GMGRG2198-13|Germany|BOLD_AAH3983
Ctenosciara hyalipennis|GMGRG1904-13|Germany|BOLD_AAH3983
Ctenosciara hyalipennis|GMGRG1915-13|Germany|BOLD_AAH3983
Ctenosciara hyalipennis|GMGRG1935-13|Germany|BOLD_AAH3983
Ctenosciara hyalipennis|GMGRG1964-13|Germany|BOLD_AAH3983
Ctenosciara hyalipennis|GMGRG1852-13|Germany|BOLD_AAH3983
Ctenosciara hyalipennis|GMGRG1867-13|Germany|BOLD_AAH3983
Ctenosciara hyalipennis|GMGRG1880-13|Germany|BOLD_AAH3983
Ctenosciara hyalipennis|GMGRG1898-13|Germany|BOLD_AAH3983
Ctenosciara hyalipennis|GMGRG738-13|Germany|BOLD_AAH3983
Ctenosciara hyalipennis|GMGRG741-13|Germany|BOLD_AAH3983
Ctenosciara hyalipennis|GMGRG773-13|Germany|BOLD_AAH3983
Ctenosciara hyalipennis|GMGRG795-13|Germany|BOLD_AAH3983
Ctenosciara hyalipennis|GMGRG1469-13|Germany|BOLD_AAH3983
Ctenosciara hyalipennis|GMGRG1474-13|Germany|BOLD_AAH3983
Ctenosciara hyalipennis|GMGRG798-13|Germany|BOLD_AAH3983
Ctenosciara hyalipennis|GMGRG819-13|Germany|BOLD_AAH3983
Ctenosciara hyalipennis|GMGRG859-13|Germany|BOLD_AAH3983
Ctenosciara hyalipennis|GMGRG871-13|Germany|BOLD_AAH3983
Ctenosciara hyalipennis|GMGRG873-13|Germany|BOLD_AAH3983
Ctenosciara hyalipennis|GMGRG874-13|Germany|BOLD_AAH3983
Ctenosciara hyalipennis|GMGRG938-13|Germany|BOLD_AAH3983
Ctenosciara hyalipennis|GMGRG1058-13|Germany|BOLD_AAH3983
Ctenosciara hyalipennis|GMGRG1602-13|Germany|BOLD_AAH3983
Ctenosciara hyalipennis|GMGRG1609-13|Germany|BOLD_AAH3983
Ctenosciara hyalipennis|GMGRG1260-13|Germany|BOLD_AAH3983
Ctenosciara hyalipennis|GMGRG1281-13|Germany|BOLD_AAH3983
Ctenosciara hyalipennis|GMGRG1334-13|Germany|BOLD_AAH3983
Ctenosciara hyalipennis|GMGRG1371-13|Germany|BOLD_AAH3983
Ctenosciara hyalipennis|GMGRF3184-13|Germany|BOLD_AAH3983
Ctenosciara hyalipennis|GMGRF3191-13|Germany|BOLD_AAH3983
Ctenosciara hyalipennis|GMGRF5921-13|Germany|BOLD_AAH3983
Ctenosciara hyalipennis|GMGRG713-13|Germany|BOLD_AAH3983
Ctenosciara hyalipennis|GMGRG721-13|Germany|BOLD_AAH3983
Ctenosciara hyalipennis|GMGRG727-13|Germany|BOLD_AAH3983
Ctenosciara hyalipennis|GMGRG1223-13|Germany|BOLD_AAH3983
Ctenosciara hyalipennis|GMGRG1246-13|Germany|BOLD_AAH3983
Ctenosciara hyalipennis|GMGRG1634-13|Germany|BOLD_AAH3983
Ctenosciara hyalipennis|GMGRG1635-13|Germany|BOLD_AAH3983
Ctenosciara hyalipennis|GMGRG1639-13|Germany|BOLD_AAH3983
Ctenosciara hyalipennis|GMGRG1732-13|Germany|BOLD_AAH3983
Ctenosciara hyalipennis|GMGRG1756-13|Germany|BOLD_AAH3983
Ctenosciara hyalipennis|GMGRG1851-13|Germany|BOLD_AAH3983
Ctenosciara hyalipennis|GMGRG3049-13|Germany|BOLD_AAH3983
Ctenosciara hyalipennis|GMGRG3107-13|Germany|BOLD_AAH3983
Ctenosciara hyalipennis|GMGRG3168-13|Germany|BOLD_AAH3983
Ctenosciara hyalipennis|GMGRG3182-13|Germany|BOLD_AAH3983
Ctenosciara hyalipennis|GMGRG3393-13|Germany|BOLD_AAH3983
Ctenosciara hyalipennis|GMGRG3478-13|Germany|BOLD_AAH3983
Ctenosciara hyalipennis|GMGRG3499-13|Germany|BOLD_AAH3983
Ctenosciara hyalipennis|GMGRG3558-13|Germany|BOLD_AAH3983
Ctenosciara hyalipennis|GMGRG1487-13|Germany|BOLD_AAH3983
Ctenosciara hyalipennis|GMGRG1586-13|Germany|BOLD_AAH3983
Ctenosciara hyalipennis|GMGRF3095-13|Germany|BOLD_AAH3983
Ctenosciara hyalipennis|GMGRF3102-13|Germany|BOLD_AAH3983
Ctenosciara hyalipennis|GMGRF2993-13|Germany|BOLD_AAH3983
Ctenosciara hyalipennis|GMGRF3087-13|Germany|BOLD_AAH3983
Ctenosciara hyalipennis|GMGRG3691-13|Germany|BOLD_AAH3983
Ctenosciara hyalipennis|GMGRF2046-13|Germany|BOLD_AAH3983
Ctenosciara hyalipennis|GMGRF1145-13|Germany|BOLD_AAH3983
Ctenosciara hyalipennis|GMGRF807-13|Germany|BOLD_AAH3983
Ctenosciara hyalipennis|GMGRF551-13|Germany|BOLD_AAH3983
Ctenosciara hyalipennis|GMGRF521-13|Germany|BOLD_AAH3983
Ctenosciara hyalipennis|GMGRB1732-13|Germany|BOLD_AAH3983
Ctenosciara hyalipennis|GMGRB1724-13|Germany|BOLD_AAH3983
Ctenosciara hyalipennis|GMGRB1670-13|Germany|BOLD_AAH3983
Ctenosciara hyalipennis|GMGRG422-13|Germany|BOLD_AAH3983
Ctenosciara hyalipennis|GMGRF2386-13|Germany|BOLD_AAH3983
Ctenosciara hyalipennis|GMGRG523-13|Germany|BOLD_AAH3983
Ctenosciara hyalipennis|GMGRG201-13|Germany|BOLD_AAH3983
Ctenosciara hyalipennis|GMGRF2171-13|Germany|BOLD_AAH3983
Ctenosciara hyalipennis|GMGRG545-13|Germany|BOLD_AAH3983
Ctenosciara hyalipennis|GMGRA853-13|Germany|BOLD_AAH3983
Ctenosciara hyalipennis|GMGRA1291-13|Germany|BOLD_AAH3983
Ctenosciara hyalipennis|GMGRG1014-13|Germany|BOLD_AAH3983
Ctenosciara hyalipennis|GMGRF745-13|Germany|BOLD_AAH3983
Ctenosciara hyalipennis|GMGRH653-13|Germany|BOLD_AAH3983
Ctenosciara hyalipennis|GMGRF6053-13|Germany|BOLD_AAH3983
Ctenosciara hyalipennis|GMGRG837-13|Germany|BOLD_AAH3983
Ctenosciara hyalipennis|GMGRB1612-13|Germany|BOLD_AAH3983

Ctenosciara hyalipennis|GMGRF6053-13|Germany|BOLD_AAH3983
Ctenosciara hyalipennis|GMGRG837-13|Germany|BOLD_AAH3983
Ctenosciara hyalipennis|GMGRB1612-13|Germany|BOLD_AAH3983
Ctenosciara hyalipennis|GMGRF1726-13|Germany|BOLD_AAH3983
Ctenosciara hyalipennis|GMGRG594-13|Germany|BOLD_AAH3983
Ctenosciara hyalipennis|GMGRH545-13|Germany|BOLD_AAH3983
Ctenosciara hyalipennis|GMGRG1936-13|Germany|BOLD_AAH3983
Ctenosciara hyalipennis|GMGRF1199-13|Germany|BOLD_AAH3983
Ctenosciara hyalipennis|GMGRB1717-13|Germany|BOLD_AAH3983
Ctenosciara hyalipennis|GMGRG652-13|Germany|BOLD_AAH3983
Ctenosciara hyalipennis|GMGRG463-13|Germany|BOLD_AAH3983
Ctenosciara hyalipennis|GMGRG460-13|Germany|BOLD_AAH3983
Ctenosciara hyalipennis|GMGRG171-13|Germany|BOLD_AAH3983
Ctenosciara hyalipennis|GMGRG168-13|Germany|BOLD_AAH3983
Ctenosciara hyalipennis|GMGRG165-13|Germany|BOLD_AAH3983
Ctenosciara hyalipennis|GMGRG070-13|Germany|BOLD_AAH3983
Ctenosciara hyalipennis|GMGRG002-13|Germany|BOLD_AAH3983
Ctenosciara hyalipennis|GMGRF2217-13|Germany|BOLD_AAH3983
Ctenosciara hyalipennis|GMGRF2175-13|Germany|BOLD_AAH3983
Ctenosciara hyalipennis|GMGRF2161-13|Germany|BOLD_AAH3983
Ctenosciara hyalipennis|GMGRF1497-13|Germany|BOLD_AAH3983
Ctenosciara hyalipennis|GMGRF4935-13|Germany|BOLD_AAH3983
Ctenosciara hyalipennis|GMGRF3164-13|Germany|BOLD_AAH3983
Ctenosciara hyalipennis|GMGRG555-13|Germany|BOLD_AAH3983
Ctenosciara hyalipennis|GMGRG519-13|Germany|BOLD_AAH3983
Ctenosciara hyalipennis|GMGRG477-13|Germany|BOLD_AAH3983
Ctenosciara hyalipennis|GMGRG451-13|Germany|BOLD_AAH3983
Ctenosciara hyalipennis|GMGRG234-13|Germany|BOLD_AAH3983
Ctenosciara hyalipennis|GMGRG223-13|Germany|BOLD_AAH3983
Ctenosciara hyalipennis|GMGRG100-13|Germany|BOLD_AAH3983
Ctenosciara hyalipennis|GMGRG072-13|Germany|BOLD_AAH3983
Ctenosciara hyalipennis|GMGRG053-13|Germany|BOLD_AAH3983
Ctenosciara hyalipennis|GMGRG051-13|Germany|BOLD_AAH3983
Ctenosciara hyalipennis|GMGRG029-13|Germany|BOLD_AAH3983
Ctenosciara hyalipennis|GMGRG010-13|Germany|BOLD_AAH3983
Ctenosciara hyalipennis|GMGRF4894-13|Germany|BOLD_AAH3983
Ctenosciara hyalipennis|GMGRG641-13|Germany|BOLD_AAH3983
Ctenosciara hyalipennis|GMGRG621-13|Germany|BOLD_AAH3983
Ctenosciara hyalipennis|GMGRG604-13|Germany|BOLD_AAH3983
Ctenosciara hyalipennis|GMGRG573-13|Germany|BOLD_AAH3983
Ctenosciara hyalipennis|GMGRG2161-13|Germany|BOLD_AAH3983
Ctenosciara hyalipennis|GMGRF2512-13|Germany|BOLD_AAH3983
Ctenosciara hyalipennis|GMGRG3608-13|Germany|BOLD_AAH3983
Ctenosciara hyalipennis|GMGRG3507-13|Germany|BOLD_AAH3983
Ctenosciara hyalipennis|GMGRG3382-13|Germany|BOLD_AAH3983
Ctenosciara hyalipennis|GMGRG2990-13|Germany|BOLD_AAH3983
Ctenosciara hyalipennis|GMGRG2839-13|Germany|BOLD_AAH3983
Ctenosciara hyalipennis|GMGRG2225-13|Germany|BOLD_AAH3983
Ctenosciara hyalipennis|GMGRG1922-13|Germany|BOLD_AAH3983
Ctenosciara hyalipennis|GMGRG1914-13|Germany|BOLD_AAH3983
Ctenosciara hyalipennis|GMGRG1905-13|Germany|BOLD_AAH3983
Ctenosciara hyalipennis|GMGRG1882-13|Germany|BOLD_AAH3983
Ctenosciara hyalipennis|GMGRG1757-13|Germany|BOLD_AAH3983
Ctenosciara hyalipennis|GMGRG1625-13|Germany|BOLD_AAH3983
Ctenosciara hyalipennis|GMGRG1581-13|Germany|BOLD_AAH3983
Ctenosciara hyalipennis|GMGRG1579-13|Germany|BOLD_AAH3983
Ctenosciara hyalipennis|GMGRG1264-13|Germany|BOLD_AAH3983
Ctenosciara hyalipennis|GMGRG1210-13|Germany|BOLD_AAH3983
Ctenosciara hyalipennis|GMGRG1208-13|Germany|BOLD_AAH3983
Ctenosciara hyalipennis|GMGRG960-13|Germany|BOLD_AAH3983
Ctenosciara hyalipennis|GMGRG950-13|Germany|BOLD_AAH3983
Ctenosciara hyalipennis|GMGRG854-13|Germany|BOLD_AAH3983
Ctenosciara hyalipennis|GMGRG796-13|Germany|BOLD_AAH3983
Ctenosciara hyalipennis|GMGRG747-13|Germany|BOLD_AAH3983
Ctenosciara hyalipennis|GMGRG731-13|Germany|BOLD_AAH3983
Ctenosciara hyalipennis|GMGRG728-13|Germany|BOLD_AAH3983
Ctenosciara hyalipennis|GMGRF3166-13|Germany|BOLD_AAH3983
Ctenosciara hyalipennis|GMGRF3118-13|Germany|BOLD_AAH3983
Ctenosciara hyalipennis|GMGRF3054-13|Germany|BOLD_AAH3983
Ctenosciara hyalipennis|GMGRF2030-13|Germany|BOLD_AAH3983
Ctenosciara hyalipennis|GMGRG3548-13|Germany|BOLD_AAH3983
Ctenosciara hyalipennis|GMGRG3514-13|Germany|BOLD_AAH3983
Ctenosciara hyalipennis|GMGRG3505-13|Germany|BOLD_AAH3983
Ctenosciara hyalipennis|GMGRG1865-13|Germany|BOLD_AAH3983
Ctenosciara hyalipennis|GMGRG1655-13|Germany|BOLD_AAH3983
Ctenosciara hyalipennis|GMGRC611-13|Germany|BOLD_AAH3983
Ctenosciara hyalipennis|GMGRG299-13|Germany|BOLD_AAH3983
Ctenosciara hyalipennis|GMGRG475-13|Germany|BOLD_AAH3983
Ctenosciara hyalipennis|GMGRF2105-13|Germany|BOLD_AAH3983
Ctenosciara hyalipennis|GMGRG256-13|Germany|BOLD_AAH3983
Ctenosciara hyalipennis|GMGRG187-13|Germany|BOLD_AAH3983
Ctenosciara hyalipennis|GMGRG2645-13|Germany|BOLD_AAH3983
Ctenosciara hyalipennis|GMGRG566-13|Germany|BOLD_AAH3983
Ctenosciara hyalipennis|GMGRG1614-13|Germany|BOLD_AAH3983
Ctenosciara hyalipennis|GMGRG1881-13|Germany|BOLD_AAH3983
Ctenosciara hyalipennis|GMGRG1933-13|Germany|BOLD_AAH3983
Ctenosciara hyalipennis|GMGRG2510-13|Germany|BOLD_AAH3983
Ctenosciara hyalipennis|GMGRG909-13|Germany|BOLD_AAH3983
Ctenosciara hyalipennis|GMGRG951-13|Germany|BOLD_AAH3983
Ctenosciara hyalipennis|GMGRG844-13|Germany|BOLD_AAH3983
Ctenosciara hyalipennis|GMGRG825-13|Germany|BOLD_AAH3983
Ctenosciara hyalipennis|GMGRG776-13|Germany|BOLD_AAH3983
Ctenosciara hyalipennis|GMGRG772-13|Germany|BOLD_AAH3983
Ctenosciara hyalipennis|GMGRG591-13|Germany|BOLD_AAH3983
Ctenosciara hyalipennis|GMGRF3777-13|Germany|BOLD_AAH3983
Ctenosciara hyalipennis|GMGRF3075-13|Germany|BOLD_AAH3983
Ctenosciara hyalipennis|GMGRF3064-13|Germany|BOLD_AAH3983
Ctenosciara hyalipennis|GMGRF2940-13|Germany|BOLD_AAH3983
Ctenosciara hyalipennis|GMGRG1883-13|Germany|BOLD_AAH3983
Ctenosciara hyalipennis|GMGRG008-13|Germany|BOLD_AAH3983
Ctenosciara hyalipennis|GMGRG488-13|Germany|BOLD_AAH3983
Ctenosciara hyalipennis|GMGRG534-13|Germany|BOLD_AAH3983

Ctenosciara hyalipennis|GMGRG008-13|Germany|BOLD_AAH3983
Ctenosciara hyalipennis|GMGRG488-13|Germany|BOLD_AAH3983
Ctenosciara hyalipennis|GMGRG534-13|Germany|BOLD_AAH3983
Ctenosciara hyalipennis|GMGRG236-13|Germany|BOLD_AAH3983
Ctenosciara hyalipennis|GMGRG301-13|Germany|BOLD_AAH3983
Ctenosciara hyalipennis|GMGRG209-13|Germany|BOLD_AAH3983
Ctenosciara hyalipennis|GMGRG229-13|Germany|BOLD_AAH3983
Ctenosciara hyalipennis|GMGRG160-13|Germany|BOLD_AAH3983
Ctenosciara hyalipennis|GMGRG196-13|Germany|BOLD_AAH3983
Ctenosciara hyalipennis|GMGRG330-13|Germany|BOLD_AAH3983
Ctenosciara hyalipennis|GMGRG332-13|Germany|BOLD_AAH3983
Ctenosciara hyalipennis|GMGRG349-13|Germany|BOLD_AAH3983
Ctenosciara hyalipennis|GMGRG440-13|Germany|BOLD_AAH3983
Ctenosciara hyalipennis|GMGRG474-13|Germany|BOLD_AAH3983
Ctenosciara hyalipennis|GMGRG502-13|Germany|BOLD_AAH3983
Ctenosciara hyalipennis|GMGRG537-13|Germany|BOLD_AAH3983
Ctenosciara hyalipennis|GMGRG539-13|Germany|BOLD_AAH3983
Ctenosciara hyalipennis|GMGRG540-13|Germany|BOLD_AAH3983
Ctenosciara hyalipennis|GMGRG547-13|Germany|BOLD_AAH3983
Ctenosciara hyalipennis|GMGRG497-13|Germany|BOLD_AAH3983
Ctenosciara hyalipennis|GMGRG550-13|Germany|BOLD_AAH3983
Ctenosciara hyalipennis|GMGRG137-13|Germany|BOLD_AAH3983
Ctenosciara hyalipennis|GMGRG111-13|Germany|BOLD_AAH3983
Ctenosciara hyalipennis|GMGRG083-13|Germany|BOLD_AAH3983
Ctenosciara hyalipennis|GMGRG038-13|Germany|BOLD_AAH3983
Ctenosciara hyalipennis|GMGRG021-13|Germany|BOLD_AAH3983
Ctenosciara hyalipennis|GMGRG007-13|Germany|BOLD_AAH3983
Ctenosciara hyalipennis|GMGRF4893-13|Germany|BOLD_AAH3983
Ctenosciara hyalipennis|GMGRG644-13|Germany|BOLD_AAH3983
Ctenosciara hyalipennis|GMGRG646-13|Germany|BOLD_AAH3983
Ctenosciara hyalipennis|GMGRG300-13|Germany|BOLD_AAH3983
Ctenosciara hyalipennis|GMGRG415-13|Germany|BOLD_AAH3983
Ctenosciara hyalipennis|GMGRG583-13|Germany|BOLD_AAH3983
Ctenosciara hyalipennis|GMGRG610-13|Germany|BOLD_AAH3983
Ctenosciara hyalipennis|GMGRH224-13|Germany|BOLD_AAH3983
Ctenosciara hyalipennis|GMGRF2103-13|Germany|BOLD_AAH3983
Ctenosciara hyalipennis|GMGRF1624-13|Germany|BOLD_AAH3983
Ctenosciara hyalipennis|GMGRF1621-13|Germany|BOLD_AAH3983
Ctenosciara hyalipennis|GMGRF1440-13|Germany|BOLD_AAH3983
Ctenosciara hyalipennis|GMGRF1386-13|Germany|BOLD_AAH3983
Ctenosciara hyalipennis|GMGRF1277-13|Germany|BOLD_AAH3983
Ctenosciara hyalipennis|GMGRF1141-13|Germany|BOLD_AAH3983
Ctenosciara hyalipennis|GMGRG1308-13|Germany|BOLD_AAH3983
Ctenosciara hyalipennis|GMGRF2629-13|Germany|BOLD_AAH3983
Ctenosciara hyalipennis|GMGRB1664-13|Germany|BOLD_AAH3983
Ctenosciara hyalipennis|GMGRH1445-13|Germany|BOLD_AAH3983
Ctenosciara hyalipennis|GMGRH1261-13|Germany|BOLD_AAH3983
Ctenosciara hyalipennis|GMGRG640-13|Germany|BOLD_AAH3983
Ctenosciara hyalipennis|GMGRG260-13|Germany|BOLD_AAH3983
Ctenosciara hyalipennis|GMGRG118-13|Germany|BOLD_AAH3983
Ctenosciara hyalipennis|GMGRF2173-13|Germany|BOLD_AAH3983
Ctenosciara hyalipennis|GMGRF1412-13|Germany|BOLD_AAH3983
Ctenosciara hyalipennis|GMGRG2595-13|Germany|BOLD_AAH3983
Ctenosciara hyalipennis|GMGRG1368-13|Germany|BOLD_AAH3983
Ctenosciara hyalipennis|GMGRG2254-13|Germany|BOLD_AAH3983
Ctenosciara hyalipennis|GMGRF4868-13|Germany|BOLD_AAH3983
Ctenosciara hyalipennis|GMGRG046-13|Germany|BOLD_AAH3983
Ctenosciara hyalipennis|GMGRH672-13|Germany|BOLD_AAH3983
Ctenosciara hyalipennis|GMGRH480-13|Germany|BOLD_AAH3983
Ctenosciara hyalipennis|GMGRH263-13|Germany|BOLD_AAH3983
Ctenosciara hyalipennis|GMGRG605-13|Germany|BOLD_AAH3983
Ctenosciara hyalipennis|GMGRG538-13|Germany|BOLD_AAH3983
Ctenosciara hyalipennis|GMGRG319-13|Germany|BOLD_AAH3983
Ctenosciara hyalipennis|GMGRG258-13|Germany|BOLD_AAH3983
Ctenosciara hyalipennis|GMGRG193-13|Germany|BOLD_AAH3983
Ctenosciara hyalipennis|GMGRG169-13|Germany|BOLD_AAH3983
Ctenosciara hyalipennis|GMGRG138-13|Germany|BOLD_AAH3983
Ctenosciara hyalipennis|GMGRG133-13|Germany|BOLD_AAH3983
Ctenosciara hyalipennis|GMGRG077-13|Germany|BOLD_AAH3983
Ctenosciara hyalipennis|GMGRF1549-13|Germany|BOLD_AAH3983
Ctenosciara hyalipennis|GMGRF1484-13|Germany|BOLD_AAH3983
Ctenosciara hyalipennis|GMGRF1207-13|Germany|BOLD_AAH3983
Ctenosciara hyalipennis|GMGRF1096-13|Germany|BOLD_AAH3983
Ctenosciara hyalipennis|GMGRG3157-13|Germany|BOLD_AAH3983
Ctenosciara hyalipennis|GMGRG2780-13|Germany|BOLD_AAH3983
Ctenosciara hyalipennis|GMGRG2754-13|Germany|BOLD_AAH3983
Ctenosciara hyalipennis|GMGRG2214-13|Germany|BOLD_AAH3983
Ctenosciara hyalipennis|GMGRG1929-13|Germany|BOLD_AAH3983
Ctenosciara hyalipennis|GMGRG913-13|Germany|BOLD_AAH3983
Ctenosciara hyalipennis|GMGRF1270-13|Germany|BOLD_AAH3983
Ctenosciara hyalipennis|GMGRF1535-13|Germany|BOLD_AAH3983
Ctenosciara hyalipennis|GMGRF2179-13|Germany|BOLD_AAH3983
Ctenosciara hyalipennis|GMGRF3151-13|Germany|BOLD_AAH3983
Ctenosciara hyalipennis|GMGRG988-13|Germany|BOLD_AAH3983
Ctenosciara hyalipennis|GMGRG990-13|Germany|BOLD_AAH3983
Ctenosciara hyalipennis|GMGRG1021-13|Germany|BOLD_AAH3983
Ctenosciara hyalipennis|GMGRG1031-13|Germany|BOLD_AAH3983
Ctenosciara hyalipennis|GMGRG1045-13|Germany|BOLD_AAH3983
Ctenosciara hyalipennis|GMGRG1203-13|Germany|BOLD_AAH3983
Ctenosciara hyalipennis|GMGRG1267-13|Germany|BOLD_AAH3983
Ctenosciara hyalipennis|GMGRG1269-13|Germany|BOLD_AAH3983
Ctenosciara hyalipennis|GMGRG1330-13|Germany|BOLD_AAH3983
Ctenosciara hyalipennis|GMGRG1366-13|Germany|BOLD_AAH3983
Ctenosciara hyalipennis|GMGRG1573-13|Germany|BOLD_AAH3983
Ctenosciara hyalipennis|GMGRG1580-13|Germany|BOLD_AAH3983
Ctenosciara hyalipennis|GMGRG1617-13|Germany|BOLD_AAH3983
Ctenosciara hyalipennis|GMGRG1648-13|Germany|BOLD_AAH3983
Ctenosciara hyalipennis|GMGRG1861-13|Germany|BOLD_AAH3983
Ctenosciara hyalipennis|GMGRG1879-13|Germany|BOLD_AAH3983
Ctenosciara hyalipennis|GMGRG2140-13|Germany|BOLD_AAH3983
Ctenosciara hyalipennis|GMGRG2164-13|Germany|BOLD_AAH3983
Ctenosciara hyalipennis|GMGRG2244-13|Germany|BOLD_AAH3983

[illegible]
Ctenosciara hyalipennis|GMGRG2140-13|Germany|BOLD_AAH3983
Ctenosciara hyalipennis|GMGRG2164-13|Germany|BOLD_AAH3983
Ctenosciara hyalipennis|GMGRG2244-13|Germany|BOLD_AAH3983
Ctenosciara hyalipennis|GMGRG2289-13|Germany|BOLD_AAH3983
Ctenosciara hyalipennis|GMGRG2317-13|Germany|BOLD_AAH3983
Ctenosciara hyalipennis|GMGRG2847-13|Germany|BOLD_AAH3983
Ctenosciara hyalipennis|GMGRG2955-13|Germany|BOLD_AAH3983
Ctenosciara hyalipennis|GMGRG2970-13|Germany|BOLD_AAH3983
Ctenosciara hyalipennis|GMGRG3508-13|Germany|BOLD_AAH3983
Ctenosciara hyalipennis|GMGRG3819-13|Germany|BOLD_AAH3983
Ctenosciara hyalipennis|GMGRG4535-13|Germany|BOLD_AAH3983
Ctenosciara hyalipennis|GMGRG4545-13|Germany|BOLD_AAH3983
Ctenosciara hyalipennis|GMGRG1015-13|Germany|BOLD_AAH3983
Ctenosciara hyalipennis|GMGRG1020-13|Germany|BOLD_AAH3983
Ctenosciara hyalipennis|GMGRG935-13|Germany|BOLD_AAH3983
Ctenosciara hyalipennis|GMGRG956-13|Germany|BOLD_AAH3983
Ctenosciara hyalipennis|GMGRG1000-13|Germany|BOLD_AAH3983
Ctenosciara hyalipennis|GMGRG1006-13|Germany|BOLD_AAH3983
Ctenosciara hyalipennis|GMGRG975-13|Germany|BOLD_AAH3983
Ctenosciara hyalipennis|GMGRG978-13|Germany|BOLD_AAH3983
Ctenosciara hyalipennis|GMGRG915-13|Germany|BOLD_AAH3983
Ctenosciara hyalipennis|GMGRG928-13|Germany|BOLD_AAH3983
Ctenosciara hyalipennis|GMGRG4586-13|Germany|BOLD_AAH3983
Ctenosciara hyalipennis|GMGRG911-13|Germany|BOLD_AAH3983
Ctenosciara hyalipennis|GMGRG902-13|Germany|BOLD_AAH3983
Ctenosciara hyalipennis|GMGRG901-13|Germany|BOLD_AAH3983
Ctenosciara hyalipennis|GMGRG894-13|Germany|BOLD_AAH3983
Ctenosciara hyalipennis|GMGRG826-13|Germany|BOLD_AAH3983
Ctenosciara hyalipennis|GMGRG802-13|Germany|BOLD_AAH3983
Ctenosciara hyalipennis|GMGRG702-13|Germany|BOLD_AAH3983
Ctenosciara hyalipennis|GMGRF2062-13|Germany|BOLD_AAH3983
Ctenosciara hyalipennis|GMGRB1725-13|Germany|BOLD_AAH3983
Ctenosciara hyalipennis|GMGRF878-13|Germany|BOLD_AAH3983
Ctenosciara hyalipennis|GMGRF3042-13|Germany|BOLD_AAH3983
Ctenosciara hyalipennis|GMGRG016-13|Germany|BOLD_AAH3983
Ctenosciara hyalipennis|GMGRF3922-13|Germany|BOLD_AAH3983
Ctenosciara hyalipennis|GMGRG2322-13|Germany|BOLD_AAH3983
Ctenosciara hyalipennis|GMGRG993-13|Germany|BOLD_AAH3983
Ctenosciara hyalipennis|GMGRB1623-13|Germany|BOLD_AAH3983
Ctenosciara hyalipennis|GMGRF4913-13|Germany|BOLD_AAH3983
Ctenosciara hyalipennis|GMGRG525-13|Germany|BOLD_AAH3983
Ctenosciara hyalipennis|GMGRG980-13|Germany|BOLD_AAH3983
Ctenosciara hyalipennis|GMGRG917-13|Germany|BOLD_AAH3983
Ctenosciara hyalipennis|GMGRI338-13|Germany|BOLD_AAH3983
Ctenosciara hyalipennis|GMGRI181-13|Germany|BOLD_AAH3983
Ctenosciara hyalipennis|GMGRG4599-13|Germany|BOLD_AAH3983
Ctenosciara hyalipennis|GMGRG2315-13|Germany|BOLD_AAH3983
Ctenosciara hyalipennis|GMGRG1486-13|Germany|BOLD_AAH3983
Ctenosciara hyalipennis|GMGRG1349-13|Germany|BOLD_AAH3983
Ctenosciara hyalipennis|GMGRG1335-13|Germany|BOLD_AAH3983
Ctenosciara hyalipennis|GMGRG1276-13|Germany|BOLD_AAH3983
Ctenosciara hyalipennis|GMGRG1268-13|Germany|BOLD_AAH3983
Ctenosciara hyalipennis|GMGRG1218-13|Germany|BOLD_AAH3983
Ctenosciara hyalipennis|GMGRG1202-13|Germany|BOLD_AAH3983
Ctenosciara hyalipennis|GMGRG1198-13|Germany|BOLD_AAH3983
Ctenosciara hyalipennis|GMGRG1038-13|Germany|BOLD_AAH3983
Ctenosciara hyalipennis|GMGRG1030-13|Germany|BOLD_AAH3983
Ctenosciara hyalipennis|GMGRG1001-13|Germany|BOLD_AAH3983
Ctenosciara hyalipennis|GMGRG923-13|Germany|BOLD_AAH3983
Ctenosciara hyalipennis|GMGRG920-13|Germany|BOLD_AAH3983
Ctenosciara hyalipennis|GMGRG903-13|Germany|BOLD_AAH3983
Ctenosciara hyalipennis|GMGRG816-13|Germany|BOLD_AAH3983
Ctenosciara hyalipennis|GMGRF3203-13|Germany|BOLD_AAH3983
Ctenosciara hyalipennis|GMGRF3186-13|Germany|BOLD_AAH3983
Ctenosciara hyalipennis|GMGRG1928-13|Germany|BOLD_AAH3983
Ctenosciara hyalipennis|GMGRG596-13|Germany|BOLD_AAH3983
Ctenosciara hyalipennis|GMGRG586-13|Germany|BOLD_AAH3983
Ctenosciara hyalipennis|GMGRG362-13|Germany|BOLD_AAH3983
Ctenosciara hyalipennis|GMGRG203-13|Germany|BOLD_AAH3983
Ctenosciara hyalipennis|GMGRG142-13|Germany|BOLD_AAH3983
Ctenosciara hyalipennis|GMGRF2626-13|Germany|BOLD_AAH3983
Ctenosciara hyalipennis|GMGRG3732-13|Germany|BOLD_AAH3983
Ctenosciara hyalipennis|GMGRG2220-13|Germany|BOLD_AAH3983
Ctenosciara hyalipennis|GMGRG2169-13|Germany|BOLD_AAH3983
Ctenosciara hyalipennis|GMGRG1279-13|Germany|BOLD_AAH3983
Ctenosciara hyalipennis|GMGRG969-13|Germany|BOLD_AAH3983
Ctenosciara hyalipennis|GMGRG719-13|Germany|BOLD_AAH3983
Ctenosciara hyalipennis|GMGRG226-13|Germany|BOLD_AAH3983
Ctenosciara hyalipennis|GMGRH842-13|Germany|BOLD_AAH3983
Ctenosciara hyalipennis|GMGRG615-13|Germany|BOLD_AAH3983
Ctenosciara hyalipennis|GMGRF1846-13|Germany|BOLD_AAH3983
Ctenosciara hyalipennis|GMGRF1805-13|Germany|BOLD_AAH3983
Ctenosciara hyalipennis|GMGRF1732-13|Germany|BOLD_AAH3983
Ctenosciara hyalipennis|GMGRG408-13|Germany|BOLD_AAH3983
Ctenosciara hyalipennis|GMGRG097-13|Germany|BOLD_AAH3983
Ctenosciara hyalipennis|GMGRG2230-13|Germany|BOLD_AAH3983
Ctenosciara hyalipennis|GMGRG2203-13|Germany|BOLD_AAH3983
Ctenosciara hyalipennis|GMGRG1200-13|Germany|BOLD_AAH3983
Ctenosciara hyalipennis|GMGRG897-13|Germany|BOLD_AAH3983
Ctenosciara hyalipennis|GMGRG836-13|Germany|BOLD_AAH3983
Ctenosciara hyalipennis|GMGRG2263-13|Germany|BOLD_AAH3983
Ctenosciara hyalipennis|GMGRG1044-13|Germany|BOLD_AAH3983
Ctenosciara hyalipennis|GMGRG085-13|Germany|BOLD_AAH3983
Ctenosciara hyalipennis|GMGRG3146-13|Germany|BOLD_AAH3983
Ctenosciara hyalipennis|GMGRG163-13|Germany|BOLD_AAH3983
Ctenosciara hyalipennis|GMGRI310-13|Germany|BOLD_AAH3983
Ctenosciara hyalipennis|GMGRG4765-13|Germany|BOLD_AAH3983
Ctenosciara hyalipennis|GMGRG1817-13|Germany|BOLD_AAH3983
Ctenosciara hyalipennis|GMGRG1498-13|Germany|BOLD_AAH3983
Ctenosciara hyalipennis|GMGRF3858-13|Germany|BOLD_AAH3983
Ctenosciara hyalipennis|GMGRB1613-13|Germany|BOLD_AAH3983
[illegible]

Ctenosciara hyalipennis|GMGRG1498-13|Germany|BOLD_AAH3983
Ctenosciara hyalipennis|GMGRF3858-13|Germany|BOLD_AAH3983
Ctenosciara hyalipennis|GMGRB1613-13|Germany|BOLD_AAH3983
Ctenosciara hyalipennis|GMGRG098-13|Germany|BOLD_AAH3983
Ctenosciara hyalipennis|GMGRG620-13|Germany|BOLD_AAH3983
Ctenosciara hyalipennis|GMGRF1656-13|Germany|BOLD_AAH3983
Ctenosciara hyalipennis|GMGRF1459-13|Germany|BOLD_AAH3983
Ctenosciara hyalipennis|GMGRG4741-13|Germany|BOLD_AAH3983
Ctenosciara hyalipennis|GMGRG957-13|Germany|BOLD_AAH3983
Ctenosciara hyalipennis|GMGRF1543-13|Germany|BOLD_AAH3983
Ctenosciara hyalipennis|GMGRF1449-13|Germany|BOLD_AAH3983
Ctenosciara hyalipennis|GMGRF1413-13|Germany|BOLD_AAH3983
Ctenosciara hyalipennis|GMGRF1350-13|Germany|BOLD_AAH3983
Ctenosciara hyalipennis|GMGRF1348-13|Germany|BOLD_AAH3983
Ctenosciara hyalipennis|GMGRF1275-13|Germany|BOLD_AAH3983
Ctenosciara hyalipennis|GMGRG2252-13|Germany|BOLD_AAH3983
Ctenosciara hyalipennis|GMGRG1590-13|Germany|BOLD_AAH3983
Ctenosciara hyalipennis|GMGRG973-13|Germany|BOLD_AAH3983
Ctenosciara hyalipennis|GMGRG971-13|Germany|BOLD_AAH3983
Ctenosciara hyalipennis|GMGRG2184-13|Germany|BOLD_AAH3983
Ctenosciara hyalipennis|GMGRG1370-13|Germany|BOLD_AAH3983
Ctenosciara hyalipennis|GMGRG1043-13|Germany|BOLD_AAH3983
Ctenosciara hyalipennis|GMGRG1013-13|Germany|BOLD_AAH3983
Ctenosciara hyalipennis|GMGRG462-13|Germany|BOLD_AAH3983
Ctenosciara hyalipennis|GMGRG313-13|Germany|BOLD_AAH3983
Ctenosciara hyalipennis|GMGRF1795-13|Germany|BOLD_AAH3983
Ctenosciara hyalipennis|GMGRF1564-13|Germany|BOLD_AAH3983
Ctenosciara hyalipennis|GMGRF1558-13|Germany|BOLD_AAH3983
Ctenosciara hyalipennis|GMGRF1185-13|Germany|BOLD_AAH3983
Ctenosciara hyalipennis|GMGRG194-13|Germany|BOLD_AAH3983
Ctenosciara hyalipennis|GMGRF1355-13|Germany|BOLD_AAH3983
Ctenosciara hyalipennis|GMGRF1347-13|Germany|BOLD_AAH3983
Ctenosciara hyalipennis|GMGRF1190-13|Germany|BOLD_AAH3983
Ctenosciara hyalipennis|GMGRG329-13|Germany|BOLD_AAH3983
Ctenosciara hyalipennis|GMGRG173-13|Germany|BOLD_AAH3983
Ctenosciara hyalipennis|GMGRG105-13|Germany|BOLD_AAH3983
Ctenosciara hyalipennis|GMGRF2169-13|Germany|BOLD_AAH3983
Ctenosciara hyalipennis|GMGRF1814-13|Germany|BOLD_AAH3983
Ctenosciara hyalipennis|GMGRG524-13|Germany|BOLD_AAH3983
Ctenosciara hyalipennis|GMGRG2135-13|Germany|BOLD_AAH3983
Ctenosciara hyalipennis|GMGRF5713-13|Germany|BOLD_AAH3983
Ctenosciara hyalipennis|GMGRF3528-13|Germany|BOLD_AAH3983
Ctenosciara hyalipennis|GMGRF3544-13|Germany|BOLD_AAH3983
Ctenosciara hyalipennis|GMGRF4993-13|Germany|BOLD_AAH3983
Ctenosciara hyalipennis|GMGRF4656-13|Germany|BOLD_AAH3983
Ctenosciara hyalipennis|GMGRF4603-13|Germany|BOLD_AAH3983
Ctenosciara hyalipennis|GMGRF420-13|Germany|BOLD_AAH3983
Ctenosciara hyalipennis|GMGRF2856-13|Germany|BOLD_AAH3983
Ctenosciara hyalipennis|GMGRF1060-13|Germany|BOLD_AAH3983
Ctenosciara hyalipennis|GMGRF4612-13|Germany|BOLD_AAH3983
Ctenosciara hyalipennis|GMGRF1690-13|Germany|BOLD_AAH3983
Ctenosciara hyalipennis|GMGRG4529-13|Germany|BOLD_AAH3983
Ctenosciara hyalipennis|GMGRG1601-13|Germany|BOLD_AAH3983
Ctenosciara hyalipennis|GMGRG1364-13|Germany|BOLD_AAH3983
Ctenosciara hyalipennis|GMGRF3212-13|Germany|BOLD_AAH3983
Ctenosciara hyalipennis|GMGRF2942-13|Germany|BOLD_AAH3983
Ctenosciara hyalipennis|GMGRF1598-13|Germany|BOLD_AAH3983
Ctenosciara hyalipennis|GMGRF1194-13|Germany|BOLD_AAH3983
Ctenosciara hyalipennis|GMGRF4792-13|Germany|BOLD_AAH3983
Ctenosciara hyalipennis|GMGRG1039-13|Germany|BOLD_AAH3983
Ctenosciara hyalipennis|GMGRG722-13|Germany|BOLD_AAH3983
Ctenosciara hyalipennis|GMGRG744-13|Germany|BOLD_AAH3983
Ctenosciara hyalipennis|GMGRG195-13|Germany|BOLD_AAH3983
Ctenosciara hyalipennis|GMGRF1706-13|Germany|BOLD_AAH3983
Ctenosciara hyalipennis|GMGRF1684-13|Germany|BOLD_AAH3983
Ctenosciara hyalipennis|GMGRF1590-13|Germany|BOLD_AAH3983
Ctenosciara hyalipennis|GMGRF877-13|Germany|BOLD_AAH3983
Ctenosciara hyalipennis|GMGRG600-13|Germany|BOLD_AAH3983
Ctenosciara hyalipennis|GMGRF2159-13|Germany|BOLD_AAH3983
Ctenosciara hyalipennis|GMGRF1680-13|Germany|BOLD_AAH3983
Ctenosciara hyalipennis|GMGRF154-13|Germany|BOLD_AAH3983
Ctenosciara hyalipennis|GMGRF030-13|Germany|BOLD_AAH3983
Ctenosciara hyalipennis|GMGRF1110-13|Germany|BOLD_AAH3983
Ctenosciara hyalipennis|GMGRF2318-13|Germany|BOLD_AAH3983
Ctenosciara hyalipennis|GMGRG509-13|Germany|BOLD_AAH3983
Ctenosciara hyalipennis|GMGRF232-13|Germany|BOLD_AAH3983
Ctenosciara hyalipennis|GMGRF053-13|Germany|BOLD_AAH3983
Ctenosciara hyalipennis|GMGRF025-13|Germany|BOLD_AAH3983
Ctenosciara hyalipennis|GMGRF1136-13|Germany|BOLD_AAH3983
Ctenosciara hyalipennis|GMGRF4926-13|Germany|BOLD_AAH3983
Ctenosciara hyalipennis|GMGRF4632-13|Germany|BOLD_AAH3983
Ctenosciara hyalipennis|GMGRF4624-13|Germany|BOLD_AAH3983
Ctenosciara hyalipennis|GMGRF2916-13|Germany|BOLD_AAH3983
Ctenosciara hyalipennis|GMGRF337-13|Germany|BOLD_AAH3983
Ctenosciara hyalipennis|GMGRB1594-13|Germany|BOLD_AAH3983
Ctenosciara hyalipennis|GMGRF4640-13|Germany|BOLD_AAH3983
Ctenosciara hyalipennis|GMGRF3462-13|Germany|BOLD_AAH3983
Ctenosciara hyalipennis|GMGRF2922-13|Germany|BOLD_AAH3983
Ctenosciara hyalipennis|GMGRF3525-13|Germany|BOLD_AAH3983
Ctenosciara hyalipennis|GMGRF2156-13|Germany|BOLD_AAH3983
Ctenosciara hyalipennis|GMGRF4941-13|Germany|BOLD_AAH3983
Ctenosciara hyalipennis|GMGRF1632-13|Germany|BOLD_AAH3983
Ctenosciara hyalipennis|GMGRF4595-13|Germany|BOLD_AAH3983
Ctenosciara hyalipennis|GMGRF2917-13|Germany|BOLD_AAH3983
Ctenosciara hyalipennis|GMGRF183-13|Germany|BOLD_AAH3983
Ctenosciara hyalipennis|GMGRF079-13|Germany|BOLD_AAH3983
Ctenosciara hyalipennis|GMGRF058-13|Germany|BOLD_AAH3983
Ctenosciara hyalipennis|GMGRF020-13|Germany|BOLD_AAH3983
Ctenosciara hyalipennis|GMGRF1483-13|Germany|BOLD_AAH3983
Ctenosciara hyalipennis|GMGRF1121-13|Germany|BOLD_AAH3983
Ctenosciara hyalipennis|GMGRF310-13|Germany|BOLD_AAH3983
Ctenosciara hyalipennis|GMGRF215-13|Germany|BOLD_AAH3983

Ctenosciara hyalipennis|GMGRF1121-13|Germany|BOLD_AAH3983
Ctenosciara hyalipennis|GMGRF310-13|Germany|BOLD_AAH3983
Ctenosciara hyalipennis|GMGRF215-13|Germany|BOLD_AAH3983
Ctenosciara hyalipennis|GMGRF161-13|Germany|BOLD_AAH3983
Ctenosciara hyalipennis|GMGRF151-13|Germany|BOLD_AAH3983
Ctenosciara hyalipennis|GMGRF126-13|Germany|BOLD_AAH3983
Ctenosciara hyalipennis|GMGRF1177-13|Germany|BOLD_AAH3983
Ctenosciara hyalipennis|GMGRF1676-13|Germany|BOLD_AAH3983
Ctenosciara hyalipennis|GMGRF3481-13|Germany|BOLD_AAH3983
Ctenosciara hyalipennis|GMGRF115-13|Germany|BOLD_AAH3983
Ctenosciara hyalipennis|GMGRF075-13|Germany|BOLD_AAH3983
Ctenosciara hyalipennis|GMGRF249-13|Germany|BOLD_AAH3983
Ctenosciara hyalipennis|GMGRG1205-13|Germany|BOLD_AAH3983
Ctenosciara hyalipennis|GMGRF2855-13|Germany|BOLD_AAH3983
Ctenosciara hyalipennis|GMGRG3534-13|Germany|BOLD_AAH3983
Ctenosciara hyalipennis|GMGRG365-13|Germany|BOLD_AAH3983
Ctenosciara hyalipennis|GMGRG520-13|Germany|BOLD_AAH3983
Ctenosciara hyalipennis|GMGRF2143-13|Germany|BOLD_AAH3983
Ctenosciara hyalipennis|GMGRF2853-13|Germany|BOLD_AAH3983
Ctenosciara hyalipennis|GMGRF1705-13|Germany|BOLD_AAH3983
Ctenosciara hyalipennis|GMGRF2454-13|Germany|BOLD_AAH3983
Ctenosciara hyalipennis|GMGRF1242-13|Germany|BOLD_AAH3983
Ctenosciara hyalipennis|GMGRF3478-13|Germany|BOLD_AAH3983
Ctenosciara hyalipennis|GMGRF034-13|Germany|BOLD_AAH3983
Ctenosciara hyalipennis|GMGRB1716-13|Germany|BOLD_AAH3983
Ctenosciara hyalipennis|GMGRB227-13|Germany|BOLD_AAH3983
Ctenosciara hyalipennis|GMGRF252-13|Germany|BOLD_AAH3983
Ctenosciara hyalipennis|GMGRG4062-13|Germany|BOLD_AAH3983
Ctenosciara hyalipennis|GMGRG4543-13|Germany|BOLD_AAH3983
Ctenosciara hyalipennis|GMGRH2475-13|Germany|BOLD_AAH3983
Ctenosciara hyalipennis|GMGRI154-13|Germany|BOLD_AAH3983
Ctenosciara hyalipennis|GMGRI401-13|Germany|BOLD_AAH3983
Ctenosciara hyalipennis|GMGRG1348-13|Germany|BOLD_AAH3983
Ctenosciara hyalipennis|GMGRG1504-13|Germany|BOLD_AAH3983
Ctenosciara hyalipennis|GMGRG1555-13|Germany|BOLD_AAH3983
Ctenosciara hyalipennis|GMGRG1560-13|Germany|BOLD_AAH3983
Ctenosciara hyalipennis|GMGRG1564-13|Germany|BOLD_AAH3983
Ctenosciara hyalipennis|GMGRG1597-13|Germany|BOLD_AAH3983
Ctenosciara hyalipennis|GMGRG3102-13|Germany|BOLD_AAH3983
Ctenosciara hyalipennis|GMGRI634-13|Germany|BOLD_AAH3983
Ctenosciara hyalipennis|GMGRF5228-13|Germany|BOLD_AAH3983
Ctenosciara hyalipennis|GMGRI620-13|Germany|BOLD_AAH3983
Ctenosciara hyalipennis|GMGRB1813-13|Germany|BOLD_AAH3983
Ctenosciara hyalipennis|GMGRG315-13|Germany|BOLD_AAH3983
Ctenosciara hyalipennis|GMGRG910-13|Germany|BOLD_AAH3983
Ctenosciara hyalipennis|GMGRG1588-13|Germany|BOLD_AAH3983
Ctenosciara hyalipennis|GMGRG2200-13|Germany|BOLD_AAH3983
Ctenosciara hyalipennis|GMGMK1417-14|Germany|BOLD_AAH3983
Ctenosciara hyalipennis|GBMWN393-15|Germany|BOLD_AAH3983
Ctenosciara hyalipennis|GMGRG112-13|Germany|BOLD_AAH3983
Ctenosciara hyalipennis|GMGRG1626-13|Germany|BOLD_AAH3983
Ctenosciara hyalipennis|GMGRG762-13|Germany|BOLD_AAH3983
Ctenosciara hyalipennis|GMGML1308-14|Germany|BOLD_AAH3983
Ctenosciara hyalipennis|GMGMG1589-14|Germany|BOLD_AAH3983
Ctenosciara hyalipennis|GMGML781-14|Germany|BOLD_AAH3983
Ctenosciara hyalipennis|GMGRF133-13|Germany|BOLD_AAH3983
Ctenosciara hyalipennis|GMGRA1330-13|Germany|BOLD_AAH3983
Ctenosciara hyalipennis|GMGRA609-13|Germany|BOLD_AAH3983
Ctenosciara hyalipennis|GMGRF2953-13|Germany|BOLD_AAH3983
Ctenosciara hyalipennis|GMGRG947-13|Germany|BOLD_AAH3983
Ctenosciara hyalipennis|GMGMG934-14|Germany|BOLD_AAH3983
Ctenosciara hyalipennis|GBMWN868-15|Germany|BOLD_AAH3983
Ctenosciara hyalipennis|FBSCI130-13|Germany|BOLD_AAH3983
Ctenosciara hyalipennis|GMGRA831-13|Germany|BOLD_AAH3983
Ctenosciara hyalipennis|GMGMG940-14|Germany|BOLD_AAH3983
Ctenosciara hyalipennis|GBMWN424-15|Germany|BOLD_AAH3983
Ctenosciara hyalipennis|GMGRG1778-13|Germany|BOLD_AAH3983
Ctenosciara hyalipennis|GMGRG1271-13|Germany|BOLD_AAH3983
Ctenosciara hyalipennis|GMGRA155-13|Germany|BOLD_AAH3983
Ctenosciara hyalipennis|GMGRB927-13|Germany|BOLD_AAH3983
Ctenosciara hyalipennis|GMGRF1851-13|Germany|BOLD_AAH3983
Ctenosciara hyalipennis|GMGRG1059-13|Germany|BOLD_AAH3983
Ctenosciara hyalipennis|GMGRG2185-13|Germany|BOLD_AAH3983
Ctenosciara hyalipennis|GMGRG4784-13|Germany|BOLD_AAH3983
Ctenosciara hyalipennis|GBMWN380-15|Germany|BOLD_AAH3983
Ctenosciara hyalipennis|GMGRH287-13|Germany|BOLD_AAH3983
Ctenosciara hyalipennis|GMGRG411-13|Germany|BOLD_AAH3983
Ctenosciara hyalipennis|GMGRG1346-13|Germany|BOLD_AAH3983
Ctenosciara hyalipennis|GMGRF2129-13|Germany|BOLD_AAH3983
Ctenosciara hyalipennis|GMGMG1153-14|Germany|BOLD_AAH3983
Ctenosciara hyalipennis|GMGRA120-13|Germany|BOLD_AAH3983
Ctenosciara hyalipennis|GMGRB218-13|Germany|BOLD_AAH3983
Ctenosciara hyalipennis|GMGRA1222-13|Germany|BOLD_AAH3983
Ctenosciara hyalipennis|GMGRG549-13|Germany|BOLD_AAH3983
Ctenosciara hyalipennis|GMGRG2261-13|Germany|BOLD_AAH3983
Ctenosciara hyalipennis|GBMWN1438-15|Germany|BOLD_AAH3983
Ctenosciara hyalipennis|GMGRF1889-13|Germany|BOLD_AAH3983
Ctenosciara hyalipennis|GMGRB1707-13|Germany|BOLD_AAH3983
Ctenosciara hyalipennis|GMGRF1766-13|Germany|BOLD_AAH3983
Ctenosciara hyalipennis|GMGRB540-13|Germany|BOLD_AAH3983
Ctenosciara hyalipennis|GMGRF1998-13|Germany|BOLD_AAH3983
Ctenosciara hyalipennis|GBMWN340-15|Germany|BOLD_AAH3983
Ctenosciara hyalipennis|GMGRG810-13|Germany|BOLD_AAH3983
Ctenosciara hyalipennis|FBSCI007-13|Germany|BOLD_AAH3983
Ctenosciara hyalipennis|GMGMG818-14|Germany|BOLD_AAH3983
Ctenosciara hyalipennis|SCINO217-15|Norway|BOLD_AAH3983
Ctenosciara hyalipennis|SCINO520-15|Norway|BOLD_AAH3983
Ctenosciara hyalipennis|GBMWN342-15|Germany|BOLD_AAH3983
Ctenosciara hyalipennis|GBMWN1011-15|Germany|BOLD_AAH3983
Ctenosciara hyalipennis|GBMWN1027-15|Germany|BOLD_AAH3983
Ctenosciara hyalipennis|GBMWN1640-15|Germany|BOLD_AAH3983
Ctenosciara hyalipennis|CNBRB290-14|Canada|BOLD_AAH3983

Ctenosciara hyalipennis|GBMWN1027-15|Germany|BOLD_AAH3983
Ctenosciara hyalipennis|GBMWN1640-15|Germany|BOLD_AAH3983
Ctenosciara hyalipennis|CNBRB290-14|Canada|BOLD_AAH3983
Ctenosciara hyalipennis|SSBRC756-14|Canada|BOLD_AAH3983
Ctenosciara hyalipennis|CNRGJ027-15|Canada|BOLD_AAH3983
Ctenosciara hyalipennis|SSBRC1153-14|Canada|BOLD_AAH3983
Ctenosciara hyalipennis|CNBRN210-14|Canada|BOLD_AAH3983
Ctenosciara hyalipennis|SSBRC1010-14|Canada|BOLD_AAH3983
Ctenosciara hyalipennis|CNTIF1605-15|Canada|BOLD_AAH3983
Ctenosciara hyalipennis|PHSEP1428-11|Canada|BOLD_AAH3983
Ctenosciara hyalipennis|CNSLA027-12|Canada|BOLD_AAH3983
Ctenosciara hyalipennis|CNSLA075-12|Canada|BOLD_AAH3983
Ctenosciara hyalipennis|CNSLP560-13|Canada|BOLD_AAH3983
Ctenosciara hyalipennis|SMTPJ5687-14|Canada|BOLD_AAH3983
Ctenosciara hyalipennis|SSBRC1040-14|Canada|BOLD_AAH3983
Ctenosciara hyalipennis|CNBRN218-14|Canada|BOLD_AAH3983
Ctenosciara hyalipennis|SSBRC935-14|Canada|BOLD_AAH3983
Ctenosciara hyalipennis|SSBRC1347-14|Canada|BOLD_AAH3983
Ctenosciara hyalipennis|CNSLA096-12|Canada|BOLD_AAH3983
Ctenosciara hyalipennis|SSBRC1314-14|Canada|BOLD_AAH3983
Ctenosciara hyalipennis|SSBRC1044-14|Canada|BOLD_AAH3983
Ctenosciara hyalipennis|CNBRP1009-14|Canada|BOLD_AAH3983
Ctenosciara hyalipennis|CNBRL510-14|Canada|BOLD_AAH3983
Ctenosciara hyalipennis|CNBRL485-14|Canada|BOLD_AAH3983
Ctenosciara hyalipennis|CNBRA022-14|Canada|BOLD_AAH3983
Ctenosciara hyalipennis|CNBRK332-14|Canada|BOLD_AAH3983
Ctenosciara hyalipennis|CNBRQ561-14|Canada|BOLD_AAH3983
Ctenosciara hyalipennis|CNBRK360-14|Canada|BOLD_AAH3983
Ctenosciara hyalipennis|SSBRC1389-14|Canada|BOLD_AAH3983
Ctenosciara hyalipennis|SSBRC696-14|Canada|BOLD_AAH3983
Ctenosciara hyalipennis|CNGBE460-14|Canada|BOLD_AAH3983
Ctenosciara hyalipennis|SSBRC1271-14|Canada|BOLD_AAH3983
Ctenosciara hyalipennis|SSBRC1232-14|Canada|BOLD_AAH3983
Ctenosciara hyalipennis|SSBRC1092-14|Canada|BOLD_AAH3983
Ctenosciara hyalipennis|SSBRC822-14|Canada|BOLD_AAH3983
Ctenosciara hyalipennis|SSBRC786-14|Canada|BOLD_AAH3983
Ctenosciara hyalipennis|SSBRC784-14|Canada|BOLD_AAH3983
Ctenosciara hyalipennis|SSBRC774-14|Canada|BOLD_AAH3983
Ctenosciara hyalipennis|SSBRC698-14|Canada|BOLD_AAH3983
Ctenosciara hyalipennis|SSBRC691-14|Canada|BOLD_AAH3983
Ctenosciara hyalipennis|SSBRC684-14|Canada|BOLD_AAH3983
Ctenosciara hyalipennis|SSBRC078-14|Canada|BOLD_AAH3983
Ctenosciara hyalipennis|SSFDC4748-14|Canada|BOLD_AAH3983
Ctenosciara hyalipennis|SSFDC3955-14|Canada|BOLD_AAH3983
Ctenosciara hyalipennis|SSFDB633-14|Canada|BOLD_AAH3983
Ctenosciara hyalipennis|SSBRC2567-14|Canada|BOLD_AAH3983
Ctenosciara hyalipennis|SSBRC2216-14|Canada|BOLD_AAH3983
Ctenosciara hyalipennis|SSBRC1937-14|Canada|BOLD_AAH3983
Ctenosciara hyalipennis|SSBRC1750-14|Canada|BOLD_AAH3983
Ctenosciara hyalipennis|SSBRC1707-14|Canada|BOLD_AAH3983
Ctenosciara hyalipennis|SSBRC1700-14|Canada|BOLD_AAH3983
Ctenosciara hyalipennis|SSBRC1397-14|Canada|BOLD_AAH3983
Ctenosciara hyalipennis|SSBRC1394-14|Canada|BOLD_AAH3983
Ctenosciara hyalipennis|SSBRC1392-14|Canada|BOLD_AAH3983
Ctenosciara hyalipennis|SSBRC1391-14|Canada|BOLD_AAH3983
Ctenosciara hyalipennis|SSBRC1387-14|Canada|BOLD_AAH3983
Ctenosciara hyalipennis|SSBRC1385-14|Canada|BOLD_AAH3983
Ctenosciara hyalipennis|SSBRC1381-14|Canada|BOLD_AAH3983
Ctenosciara hyalipennis|SSBRC1377-14|Canada|BOLD_AAH3983
Ctenosciara hyalipennis|SSBRC1373-14|Canada|BOLD_AAH3983
Ctenosciara hyalipennis|SSBRC1372-14|Canada|BOLD_AAH3983
Ctenosciara hyalipennis|SSBRC1370-14|Canada|BOLD_AAH3983
Ctenosciara hyalipennis|SSBRC1363-14|Canada|BOLD_AAH3983
Ctenosciara hyalipennis|SSBRC1352-14|Canada|BOLD_AAH3983
Ctenosciara hyalipennis|SSBRC1349-14|Canada|BOLD_AAH3983
Ctenosciara hyalipennis|SSBRC1348-14|Canada|BOLD_AAH3983
Ctenosciara hyalipennis|SSBRC1275-14|Canada|BOLD_AAH3983
Ctenosciara hyalipennis|SSBRC1263-14|Canada|BOLD_AAH3983
Ctenosciara hyalipennis|SSBRC1242-14|Canada|BOLD_AAH3983
Ctenosciara hyalipennis|SSBRC1240-14|Canada|BOLD_AAH3983
Ctenosciara hyalipennis|SSBRC1222-14|Canada|BOLD_AAH3983
Ctenosciara hyalipennis|SSBRC1219-14|Canada|BOLD_AAH3983
Ctenosciara hyalipennis|SSBRC1193-14|Canada|BOLD_AAH3983
Ctenosciara hyalipennis|SSBRC1174-14|Canada|BOLD_AAH3983
Ctenosciara hyalipennis|SSBRC1167-14|Canada|BOLD_AAH3983
Ctenosciara hyalipennis|SSBRC1134-14|Canada|BOLD_AAH3983
Ctenosciara hyalipennis|SSBRC1133-14|Canada|BOLD_AAH3983
Ctenosciara hyalipennis|SSBRC1127-14|Canada|BOLD_AAH3983
Ctenosciara hyalipennis|SSBRC1119-14|Canada|BOLD_AAH3983
Ctenosciara hyalipennis|SSBRC1101-14|Canada|BOLD_AAH3983
Ctenosciara hyalipennis|SSBRC1098-14|Canada|BOLD_AAH3983
Ctenosciara hyalipennis|SSBRC1078-14|Canada|BOLD_AAH3983
Ctenosciara hyalipennis|SSBRC1076-14|Canada|BOLD_AAH3983
Ctenosciara hyalipennis|SSBRC1067-14|Canada|BOLD_AAH3983
Ctenosciara hyalipennis|SSBRC1062-14|Canada|BOLD_AAH3983
Ctenosciara hyalipennis|SSBRC1061-14|Canada|BOLD_AAH3983
Ctenosciara hyalipennis|SSBRC1046-14|Canada|BOLD_AAH3983
Ctenosciara hyalipennis|SSBRC1043-14|Canada|BOLD_AAH3983
Ctenosciara hyalipennis|SSBRC1035-14|Canada|BOLD_AAH3983
Ctenosciara hyalipennis|SSBRC1027-14|Canada|BOLD_AAH3983
Ctenosciara hyalipennis|SSBRC997-14|Canada|BOLD_AAH3983
Ctenosciara hyalipennis|SSBRC985-14|Canada|BOLD_AAH3983
Ctenosciara hyalipennis|SSBRC950-14|Canada|BOLD_AAH3983
Ctenosciara hyalipennis|SSBRC936-14|Canada|BOLD_AAH3983
Ctenosciara hyalipennis|SSBRC844-14|Canada|BOLD_AAH3983
Ctenosciara hyalipennis|SSBRC815-14|Canada|BOLD_AAH3983
Ctenosciara hyalipennis|SSBRC733-14|Canada|BOLD_AAH3983
Ctenosciara hyalipennis|SSBRC718-14|Canada|BOLD_AAH3983
Ctenosciara hyalipennis|SSBRC715-14|Canada|BOLD_AAH3983
Ctenosciara hyalipennis|SSBRC707-14|Canada|BOLD_AAH3983
Ctenosciara hyalipennis|SSBRC693-14|Canada|BOLD_AAH3983
Ctenosciara hyalipennis|SSBRC690-14|Canada|BOLD_AAH3983

[illegible]
Ctenosciara hyalipennis|SSBRC707-14|Canada|BOLD_AAH3983
Ctenosciara hyalipennis|SSBRC693-14|Canada|BOLD_AAH3983
Ctenosciara hyalipennis|SSBRC690-14|Canada|BOLD_AAH3983
Ctenosciara hyalipennis|SSBRC685-14|Canada|BOLD_AAH3983
Ctenosciara hyalipennis|SSBRC682-14|Canada|BOLD_AAH3983
Ctenosciara hyalipennis|SSBRC658-14|Canada|BOLD_AAH3983
Ctenosciara hyalipennis|SSBRC063-14|Canada|BOLD_AAH3983
Ctenosciara hyalipennis|CNBRK107-14|Canada|BOLD_AAH3983
Ctenosciara hyalipennis|CNBRQ557-14|Canada|BOLD_AAH3983
Ctenosciara hyalipennis|SSBRC755-14|Canada|BOLD_AAH3983
Ctenosciara hyalipennis|CNBRN182-14|Canada|BOLD_AAH3983
Ctenosciara hyalipennis|CNBRK340-14|Canada|BOLD_AAH3983
Ctenosciara hyalipennis|CNPEB117-14|Canada|BOLD_AAH3983
Ctenosciara hyalipennis|CNBRA039-14|Canada|BOLD_AAH3983
Ctenosciara hyalipennis|CNBRB272-14|Canada|BOLD_AAH3983
Ctenosciara hyalipennis|CNBRA072-14|Canada|BOLD_AAH3983
Ctenosciara hyalipennis|SSBRC773-14|Canada|BOLD_AAH3983
Ctenosciara hyalipennis|SSBRC796-14|Canada|BOLD_AAH3983
Ctenosciara hyalipennis|SSBRC988-14|Canada|BOLD_AAH3983
Ctenosciara hyalipennis|SSBRC1100-14|Canada|BOLD_AAH3983
Ctenosciara hyalipennis|SSBRC1343-14|Canada|BOLD_AAH3983
Ctenosciara hyalipennis|SSBRC1364-14|Canada|BOLD_AAH3983
Ctenosciara hyalipennis|SSBRC1931-14|Canada|BOLD_AAH3983
Ctenosciara hyalipennis|SSBRC735-14|Canada|BOLD_AAH3983
Ctenosciara hyalipennis|SSBRC726-14|Canada|BOLD_AAH3983
Ctenosciara hyalipennis|SSBRC663-14|Canada|BOLD_AAH3983
Ctenosciara hyalipennis|CNBRR181-14|Canada|BOLD_AAH3983
Ctenosciara hyalipennis|CNTIB501-15|Canada|BOLD_AAH3983
Ctenosciara hyalipennis|SSBRC1396-14|Canada|BOLD_AAH3983
Ctenosciara hyalipennis|SSBRC1365-14|Canada|BOLD_AAH3983
Ctenosciara hyalipennis|SSBRC1207-14|Canada|BOLD_AAH3983
Ctenosciara hyalipennis|SSBRC1097-14|Canada|BOLD_AAH3983
Ctenosciara hyalipennis|SSBRC993-14|Canada|BOLD_AAH3983
Ctenosciara hyalipennis|SSBRC986-14|Canada|BOLD_AAH3983
Ctenosciara hyalipennis|CNBRK195-14|Canada|BOLD_AAH3983
Ctenosciara hyalipennis|CNBRK157-14|Canada|BOLD_AAH3983
Ctenosciara hyalipennis|CNBRK154-14|Canada|BOLD_AAH3983
Ctenosciara hyalipennis|SSBRC1208-14|Canada|BOLD_AAH3983
Ctenosciara hyalipennis|CNBRA141-14|Canada|BOLD_AAH3983
Ctenosciara hyalipennis|SMTPJ5631-14|Canada|BOLD_AAH3983
Ctenosciara hyalipennis|SSBRC2845-14|Canada|BOLD_AAH3983
Ctenosciara hyalipennis|SSBRC2085-14|Canada|BOLD_AAH3983
Ctenosciara hyalipennis|SSBRC1325-14|Canada|BOLD_AAH3983
Ctenosciara hyalipennis|SSBRC1316-14|Canada|BOLD_AAH3983
Ctenosciara hyalipennis|SSBRC976-14|Canada|BOLD_AAH3983
Ctenosciara hyalipennis|SSBRC932-14|Canada|BOLD_AAH3983
Ctenosciara hyalipennis|CNBRB165-14|Canada|BOLD_AAH3983
Ctenosciara hyalipennis|SSBRC1946-14|Canada|BOLD_AAH3983
Ctenosciara hyalipennis|SSBRC1393-14|Canada|BOLD_AAH3983
Ctenosciara hyalipennis|SSBRC1110-14|Canada|BOLD_AAH3983
Ctenosciara hyalipennis|SSBRC660-14|Canada|BOLD_AAH3983
Ctenosciara hyalipennis|SMTPJ5719-14|Canada|BOLD_AAH3983
Ctenosciara hyalipennis|SSBRC1112-14|Canada|BOLD_AAH3983
Ctenosciara hyalipennis|CNBRK401-14|Canada|BOLD_AAH3983
Ctenosciara hyalipennis|SSBRC3248-14|Canada|BOLD_AAH3983
Ctenosciara hyalipennis|SSBRC1380-14|Canada|BOLD_AAH3983
Ctenosciara hyalipennis|SSBRC1354-14|Canada|BOLD_AAH3983
Ctenosciara hyalipennis|SSBRC1229-14|Canada|BOLD_AAH3983
Ctenosciara hyalipennis|SSBRC1187-14|Canada|BOLD_AAH3983
Ctenosciara hyalipennis|SSBRC939-14|Canada|BOLD_AAH3983
Ctenosciara hyalipennis|SSBRC938-14|Canada|BOLD_AAH3983
Ctenosciara hyalipennis|SSBRC930-14|Canada|BOLD_AAH3983
Ctenosciara hyalipennis|CNBRF261-14|Canada|BOLD_AAH3983
Ctenosciara hyalipennis|SSBRC1918-14|Canada|BOLD_AAH3983
Ctenosciara hyalipennis|SSBRC1149-14|Canada|BOLD_AAH3983
Ctenosciara hyalipennis|SSBRC963-14|Canada|BOLD_AAH3983
Ctenosciara hyalipennis|CNBRA117-14|Canada|BOLD_AAH3983
Ctenosciara hyalipennis|SSBRC1889-14|Canada|BOLD_AAH3983
Ctenosciara hyalipennis|SSBRC1204-14|Canada|BOLD_AAH3983
Ctenosciara hyalipennis|SSBRC657-14|Canada|BOLD_AAH3983
Ctenosciara hyalipennis|CNBRN180-14|Canada|BOLD_AAH3983
Ctenosciara hyalipennis|SSBRC931-14|Canada|BOLD_AAH3983
Ctenosciara hyalipennis|PANOV082-12|Canada|BOLD_AAH3983
Ctenosciara hyalipennis|SSBRC1164-14|Canada|BOLD_AAH3983
Ctenosciara hyalipennis|CNTIE1221-15|Canada|BOLD_AAH3983
Ctenosciara hyalipennis|CNTIG1006-15|Canada|BOLD_AAH3983
Ctenosciara hyalipennis|RRSSA2547-15|Canada|BOLD_AAH3983
Ctenosciara hyalipennis|RRSSA3297-15|Canada|BOLD_AAH3983
Ctenosciara hyalipennis|GMGRA398-13|Germany|BOLD_AAH3983
Ctenosciara hyalipennis|GMGRF3705-13|Germany|BOLD_AAH3983
Ctenosciara hyalipennis|GMGRG1899-13|Germany|BOLD_AAH3983
Ctenosciara hyalipennis|SSPRA496-15|Canada|BOLD_AAH3983
Ctenosciara hyalipennis|CNGAC624-15|Canada|BOLD_AAH3983
Ctenosciara hyalipennis|HEOCT1099-12|Canada|BOLD_AAH3983
Ctenosciara hyalipennis|RRSSA3465-15|Canada|BOLD_AAH3983
Ctenosciara hyalipennis|RRSSA2768-15|Canada|BOLD_AAH3983
Ctenosciara hyalipennis|SMTPM6037-15|Canada|BOLD_AAH3983
Ctenosciara hyalipennis|CNRVG767-15|Canada|BOLD_AAH3983
Ctenosciara hyalipennis|CNGUH358-15|Canada|BOLD_AAH3983
Ctenosciara hyalipennis|CNROC259-13|Canada|BOLD_AAH3983
Ctenosciara hyalipennis|CNRVG1116-15|Canada|BOLD_AAH3983
Ctenosciara hyalipennis|GMGMG975-14|Germany|BOLD_AAH3983
Ctenosciara hyalipennis|RRSSA1147-15|Canada|BOLD_AAH3983
Ctenosciara hyalipennis|RRSSA939-15|Canada|BOLD_AAH3983
Ctenosciara hyalipennis|RRMFD1282-15|Canada|BOLD_AAH3983
Ctenosciara hyalipennis|CNRVF331-15|Canada|BOLD_AAH3983
Ctenosciara hyalipennis|CNRVF158-15|Canada|BOLD_AAH3983
Ctenosciara hyalipennis|CNGAC308-15|Canada|BOLD_AAH3983
Ctenosciara hyalipennis|CNRVA1327-15|Canada|BOLD_AAH3983
Ctenosciara hyalipennis|CNRVA669-15|Canada|BOLD_AAH3983
Ctenosciara hyalipennis|CNGAB684-15|Canada|BOLD_AAH3983
[illegible]

Ctenosciara hyalipennis|CNRVA1327-15|Canada|BOLD_AAH3983
Ctenosciara hyalipennis|CNRVA669-15|Canada|BOLD_AAH3983
Ctenosciara hyalipennis|CNGAB684-15|Canada|BOLD_AAH3983
Ctenosciara hyalipennis|CNGAB671-15|Canada|BOLD_AAH3983
Ctenosciara hyalipennis|CNGAB660-15|Canada|BOLD_AAH3983
Ctenosciara hyalipennis|CNGAB392-15|Canada|BOLD_AAH3983
Ctenosciara hyalipennis|CNGUC983-15|Canada|BOLD_AAH3983
Ctenosciara hyalipennis|RRSSA2577-15|Canada|BOLD_AAH3983
Ctenosciara hyalipennis|RRSSA2566-15|Canada|BOLD_AAH3983
Ctenosciara hyalipennis|RRSSA2219-15|Canada|BOLD_AAH3983
Ctenosciara hyalipennis|RRSSA1139-15|Canada|BOLD_AAH3983
Ctenosciara hyalipennis|RRMFE278-15|Canada|BOLD_AAH3983
Ctenosciara hyalipennis|CNRVG1565-15|Canada|BOLD_AAH3983
Ctenosciara hyalipennis|CNRVG1137-15|Canada|BOLD_AAH3983
Ctenosciara hyalipennis|CNRVG931-15|Canada|BOLD_AAH3983
Ctenosciara hyalipennis|CNRVG874-15|Canada|BOLD_AAH3983
Ctenosciara hyalipennis|CNRVG815-15|Canada|BOLD_AAH3983
Ctenosciara hyalipennis|CNRVG730-15|Canada|BOLD_AAH3983
Ctenosciara hyalipennis|CNRVF445-15|Canada|BOLD_AAH3983
Ctenosciara hyalipennis|CNRVF392-15|Canada|BOLD_AAH3983
Ctenosciara hyalipennis|CNRVF383-15|Canada|BOLD_AAH3983
Ctenosciara hyalipennis|CNRVF335-15|Canada|BOLD_AAH3983
Ctenosciara hyalipennis|CNRVF325-15|Canada|BOLD_AAH3983
Ctenosciara hyalipennis|CNRVF189-15|Canada|BOLD_AAH3983
Ctenosciara hyalipennis|CNRVE2667-15|Canada|BOLD_AAH3983
Ctenosciara hyalipennis|CNRVE2550-15|Canada|BOLD_AAH3983
Ctenosciara hyalipennis|CNRVE1666-15|Canada|BOLD_AAH3983
Ctenosciara hyalipennis|CNGAH199-15|Canada|BOLD_AAH3983
Ctenosciara hyalipennis|CNGAC623-15|Canada|BOLD_AAH3983
Ctenosciara hyalipennis|CNGAC528-15|Canada|BOLD_AAH3983
Ctenosciara hyalipennis|CNRVA1344-15|Canada|BOLD_AAH3983
Ctenosciara hyalipennis|CNRVA1119-15|Canada|BOLD_AAH3983
Ctenosciara hyalipennis|CNRVA594-15|Canada|BOLD_AAH3983
Ctenosciara hyalipennis|CNGAB665-15|Canada|BOLD_AAH3983
Ctenosciara hyalipennis|CNGAB498-15|Canada|BOLD_AAH3983
Ctenosciara hyalipennis|CNGAB450-15|Canada|BOLD_AAH3983
Ctenosciara hyalipennis|CNGAB366-15|Canada|BOLD_AAH3983
Ctenosciara hyalipennis|CNGAA236-15|Canada|BOLD_AAH3983
Ctenosciara hyalipennis|CNGUH403-15|Canada|BOLD_AAH3983
Ctenosciara hyalipennis|CNGUD214-15|Canada|BOLD_AAH3983
Ctenosciara hyalipennis|CNGUC635-15|Canada|BOLD_AAH3983
Ctenosciara hyalipennis|CNGUC617-15|Canada|BOLD_AAH3983
Ctenosciara hyalipennis|CNGUB396-14|Canada|BOLD_AAH3983
Ctenosciara hyalipennis|CNGUB358-14|Canada|BOLD_AAH3983
Ctenosciara hyalipennis|CNGUB356-14|Canada|BOLD_AAH3983
Ctenosciara hyalipennis|CNGUB354-14|Canada|BOLD_AAH3983
Ctenosciara hyalipennis|CNGUB314-14|Canada|BOLD_AAH3983
Ctenosciara hyalipennis|CNGUB302-14|Canada|BOLD_AAH3983
Ctenosciara hyalipennis|CNGUB274-14|Canada|BOLD_AAH3983
Ctenosciara hyalipennis|CNGUB061-14|Canada|BOLD_AAH3983
Ctenosciara hyalipennis|CNGUB030-14|Canada|BOLD_AAH3983
Ctenosciara hyalipennis|CNGUA106-14|Canada|BOLD_AAH3983
Ctenosciara hyalipennis|GMGMG944-14|Germany|BOLD_AAH3983
Ctenosciara hyalipennis|PHOCT533-11|Canada|BOLD_AAH3983
Ctenosciara hyalipennis|HEAUG818-12|Canada|BOLD_AAH3983
Ctenosciara hyalipennis|RRSSA022-15|Canada|BOLD_AAH3983
Ctenosciara hyalipennis|CNGAB492-15|Canada|BOLD_AAH3983
Ctenosciara hyalipennis|SMTPJ4758-14|Canada|BOLD_AAH3983
Ctenosciara hyalipennis|RRSSA2779-15|Canada|BOLD_AAH3983
Ctenosciara hyalipennis|CNRVG818-15|Canada|BOLD_AAH3983
Ctenosciara hyalipennis|RRSSA2220-15|Canada|BOLD_AAH3983
Ctenosciara hyalipennis|CNRVG1437-15|Canada|BOLD_AAH3983
Ctenosciara hyalipennis|CNGAB500-15|Canada|BOLD_AAH3983
Ctenosciara hyalipennis|CNGUC588-15|Canada|BOLD_AAH3983
Ctenosciara hyalipennis|RRSSA2937-15|Canada|BOLD_AAH3983
Ctenosciara hyalipennis|RRSSA2825-15|Canada|BOLD_AAH3983
Ctenosciara hyalipennis|RRSSA2816-15|Canada|BOLD_AAH3983
Ctenosciara hyalipennis|RRSSA2795-15|Canada|BOLD_AAH3983
Ctenosciara hyalipennis|RRSSA2775-15|Canada|BOLD_AAH3983
Ctenosciara hyalipennis|RRSSA2218-15|Canada|BOLD_AAH3983
Ctenosciara hyalipennis|RRSSA2202-15|Canada|BOLD_AAH3983
Ctenosciara hyalipennis|CNTIF701-15|Canada|BOLD_AAH3983
Ctenosciara hyalipennis|CNRVG1906-15|Canada|BOLD_AAH3983
Ctenosciara hyalipennis|CNRVG1588-15|Canada|BOLD_AAH3983
Ctenosciara hyalipennis|CNGUC620-15|Canada|BOLD_AAH3983
Ctenosciara hyalipennis|CNGUC597-15|Canada|BOLD_AAH3983
Ctenosciara hyalipennis|CNGUC332-15|Canada|BOLD_AAH3983
Ctenosciara hyalipennis|RRSSA3321-15|Canada|BOLD_AAH3983
Ctenosciara hyalipennis|RRSSA2558-15|Canada|BOLD_AAH3983
Ctenosciara hyalipennis|RRSSA2552-15|Canada|BOLD_AAH3983
Ctenosciara hyalipennis|CNRVG1820-15|Canada|BOLD_AAH3983
Ctenosciara hyalipennis|CNGAB461-15|Canada|BOLD_AAH3983
Ctenosciara hyalipennis|CNGAB435-15|Canada|BOLD_AAH3983
Ctenosciara hyalipennis|CNRVG1492-15|Canada|BOLD_AAH3983
Ctenosciara hyalipennis|CNGAB102-15|Canada|BOLD_AAH3983
Ctenosciara hyalipennis|CNRVG1809-15|Canada|BOLD_AAH3983
Ctenosciara hyalipennis|RRSSA166-15|Canada|BOLD_AAH3983
Ctenosciara hyalipennis|RRSSA2763-15|Canada|BOLD_AAH3983
Ctenosciara hyalipennis|CNRVG791-15|Canada|BOLD_AAH3983
Ctenosciara hyalipennis|CNRVG817-15|Canada|BOLD_AAH3983
Ctenosciara hyalipennis|CNRVF183-15|Canada|BOLD_AAH3983
Ctenosciara hyalipennis|RRSSA2559-15|Canada|BOLD_AAH3983
Ctenosciara hyalipennis|RRSSA2554-15|Canada|BOLD_AAH3983
Ctenosciara hyalipennis|CNRVF374-15|Canada|BOLD_AAH3983
Ctenosciara hyalipennis|CNGAB337-15|Canada|BOLD_AAH3983
Ctenosciara hyalipennis|RRSSA2747-15|Canada|BOLD_AAH3983
Ctenosciara hyalipennis|CNGUC156-15|Canada|BOLD_AAH3983
Ctenosciara hyalipennis|RRSSA2556-15|Canada|BOLD_AAH3983
Ctenosciara hyalipennis|RRSSA2776-15|Canada|BOLD_AAH3983
Ctenosciara hyalipennis|RRSSA3410-15|Canada|BOLD_AAH3983
Ctenosciara hyalipennis|RRSSA3482-15|Canada|BOLD_AAH3983
Ctenosciara hyalipennis|RRSSA3487-15|Canada|BOLD_AAH3983

Ctenosciara hyalipennis|RRSSA3410-15|Canada|BOLD_AAH3983
Ctenosciara hyalipennis|RRSSA3482-15|Canada|BOLD_AAH3983
Ctenosciara hyalipennis|RRSSA3487-15|Canada|BOLD_AAH3983
Ctenosciara hyalipennis|GMGRG2732-13|Germany|BOLD_AAH3983
Ctenosciara hyalipennis|GMGRA819-13|Germany|BOLD_AAH3983
Ctenosciara hyalipennis|GMGRB380-13|Germany|BOLD_AAH3983
Ctenosciara hyalipennis|GMGRB633-13|Germany|BOLD_AAH3983
Ctenosciara hyalipennis|GMGRG128-13|Germany|BOLD_AAH3983
Ctenosciara hyalipennis|GMGRG576-13|Germany|BOLD_AAH3983
Ctenosciara hyalipennis|GMGRB1316-13|Germany|BOLD_AAH3983
Ctenosciara hyalipennis|GMGRG659-13|Germany|BOLD_AAH3983
Ctenosciara hyalipennis|GBMWN351-15|Germany|BOLD_AAH3983
Ctenosciara hyalipennis|CNPEB958-14|Canada|BOLD_AAH3983
Ctenosciara hyalipennis|CNBRB203-14|Canada|BOLD_AAH3983
Ctenosciara hyalipennis|CNPEU168-14|Canada|BOLD_AAH3983
Ctenosciara hyalipennis|CNBRB210-14|Canada|BOLD_AAH3983
Ctenosciara hyalipennis|CNBRQ422-14|Canada|BOLD_AAH3983
Ctenosciara hyalipennis|CNBRQ415-14|Canada|BOLD_AAH3983
Ctenosciara hyalipennis|CNBRQ175-14|Canada|BOLD_AAH3983
Ctenosciara hyalipennis|CNPEQ143-14|Canada|BOLD_AAH3983
Ctenosciara hyalipennis|CNPEN770-14|Canada|BOLD_AAH3983
Ctenosciara hyalipennis|CNBRL531-14|Canada|BOLD_AAH3983
Ctenosciara hyalipennis|CNBRK436-14|Canada|BOLD_AAH3983
Ctenosciara hyalipennis|CNBRK435-14|Canada|BOLD_AAH3983
Ctenosciara hyalipennis|CNBRK434-14|Canada|BOLD_AAH3983
Ctenosciara hyalipennis|CNBRK429-14|Canada|BOLD_AAH3983
Ctenosciara hyalipennis|CNBRK416-14|Canada|BOLD_AAH3983
Ctenosciara hyalipennis|CNBRK411-14|Canada|BOLD_AAH3983
Ctenosciara hyalipennis|CNBRK409-14|Canada|BOLD_AAH3983
Ctenosciara hyalipennis|CNBRK407-14|Canada|BOLD_AAH3983
Ctenosciara hyalipennis|CNBRK405-14|Canada|BOLD_AAH3983
Ctenosciara hyalipennis|CNBRK396-14|Canada|BOLD_AAH3983
Ctenosciara hyalipennis|CNBRK392-14|Canada|BOLD_AAH3983
Ctenosciara hyalipennis|CNBRK389-14|Canada|BOLD_AAH3983
Ctenosciara hyalipennis|CNBRK385-14|Canada|BOLD_AAH3983
Ctenosciara hyalipennis|CNBRK384-14|Canada|BOLD_AAH3983
Ctenosciara hyalipennis|CNBRK383-14|Canada|BOLD_AAH3983
Ctenosciara hyalipennis|CNBRK358-14|Canada|BOLD_AAH3983
Ctenosciara hyalipennis|CNBRK345-14|Canada|BOLD_AAH3983
Ctenosciara hyalipennis|CNBRK295-14|Canada|BOLD_AAH3983
Ctenosciara hyalipennis|CNBRK292-14|Canada|BOLD_AAH3983
Ctenosciara hyalipennis|CNBRG066-14|Canada|BOLD_AAH3983
Ctenosciara hyalipennis|CNPEB1519-14|Canada|BOLD_AAH3983
Ctenosciara hyalipennis|CNPEB1364-14|Canada|BOLD_AAH3983
Ctenosciara hyalipennis|CNPEB1327-14|Canada|BOLD_AAH3983
Ctenosciara hyalipennis|CNPEB1319-14|Canada|BOLD_AAH3983
Ctenosciara hyalipennis|CNPEB567-14|Canada|BOLD_AAH3983
Ctenosciara hyalipennis|CNPEB532-14|Canada|BOLD_AAH3983
Ctenosciara hyalipennis|CNPEB518-14|Canada|BOLD_AAH3983
Ctenosciara hyalipennis|CNPEB422-14|Canada|BOLD_AAH3983
Ctenosciara hyalipennis|CNPEB349-14|Canada|BOLD_AAH3983
Ctenosciara hyalipennis|CNPEB252-14|Canada|BOLD_AAH3983
Ctenosciara hyalipennis|CNBRB351-14|Canada|BOLD_AAH3983
Ctenosciara hyalipennis|CNBRB328-14|Canada|BOLD_AAH3983
Ctenosciara hyalipennis|CNBRB117-14|Canada|BOLD_AAH3983
Ctenosciara hyalipennis|CNBRN166-14|Canada|BOLD_AAH3983
Ctenosciara hyalipennis|CNBRN162-14|Canada|BOLD_AAH3983
Ctenosciara hyalipennis|CNBRN125-14|Canada|BOLD_AAH3983
Ctenosciara hyalipennis|CNPEM3098-14|Canada|BOLD_AAH3983
Ctenosciara hyalipennis|CNBRK403-14|Canada|BOLD_AAH3983
Ctenosciara hyalipennis|CNBRK289-14|Canada|BOLD_AAH3983
Ctenosciara hyalipennis|CNBRB192-14|Canada|BOLD_AAH3983
Ctenosciara hyalipennis|HPPPK407-13|Canada|BOLD_AAH3983
Ctenosciara hyalipennis|HPPPK332-13|Canada|BOLD_AAH3983
Ctenosciara hyalipennis|HPPPK209-13|Canada|BOLD_AAH3983
Ctenosciara hyalipennis|HPPPJ640-13|Canada|BOLD_AAH3983
Ctenosciara hyalipennis|HPPPJ593-13|Canada|BOLD_AAH3983
Ctenosciara hyalipennis|HPPPJ321-13|Canada|BOLD_AAH3983
Ctenosciara hyalipennis|HPPPJ289-13|Canada|BOLD_AAH3983
Ctenosciara hyalipennis|HPPPJ187-13|Canada|BOLD_AAH3983
Ctenosciara hyalipennis|HPPPJ166-13|Canada|BOLD_AAH3983
Ctenosciara hyalipennis|HPPPJ152-13|Canada|BOLD_AAH3983
Ctenosciara hyalipennis|HPPPJ115-13|Canada|BOLD_AAH3983
Ctenosciara hyalipennis|HPPPE735-13|Canada|BOLD_AAH3983
Ctenosciara hyalipennis|HPPPA457-13|Canada|BOLD_AAH3983
Ctenosciara hyalipennis|HPPPA385-13|Canada|BOLD_AAH3983
Ctenosciara hyalipennis|HPPPA209-13|Canada|BOLD_AAH3983
Ctenosciara hyalipennis|HPPPF103-13|Canada|BOLD_AAH3983
Ctenosciara hyalipennis|HPPPE1071-13|Canada|BOLD_AAH3983
Ctenosciara hyalipennis|HPPPE759-13|Canada|BOLD_AAH3983
Ctenosciara hyalipennis|HPPPE698-13|Canada|BOLD_AAH3983
Ctenosciara hyalipennis|HPPPE668-13|Canada|BOLD_AAH3983
Ctenosciara hyalipennis|CNBRG168-14|Canada|BOLD_AAH3983
Ctenosciara hyalipennis|CNPEB143-14|Canada|BOLD_AAH3983
Ctenosciara hyalipennis|CNPEN632-14|Canada|BOLD_AAH3983
Ctenosciara hyalipennis|CNPEL745-14|Canada|BOLD_AAH3983
Ctenosciara hyalipennis|CNPER128-14|Canada|BOLD_AAH3983
Ctenosciara hyalipennis|CNPER095-14|Canada|BOLD_AAH3983
Ctenosciara hyalipennis|SSBRB4744-14|Canada|BOLD_AAH3983
Ctenosciara hyalipennis|CNPEL471-14|Canada|BOLD_AAH3983
Ctenosciara hyalipennis|CNBRI029-14|Canada|BOLD_AAH3983
Ctenosciara hyalipennis|CNBRK338-14|Canada|BOLD_AAH3983
Ctenosciara hyalipennis|CNBRF056-14|Canada|BOLD_AAH3983
Ctenosciara hyalipennis|CNPEB210-14|Canada|BOLD_AAH3983
Ctenosciara hyalipennis|CNBRA109-14|Canada|BOLD_AAH3983
Ctenosciara hyalipennis|HPPPA416-13|Canada|BOLD_AAH3983
Ctenosciara hyalipennis|HPPPF535-13|Canada|BOLD_AAH3983
Ctenosciara hyalipennis|CNPEL627-14|Canada|BOLD_AAH3983
Ctenosciara hyalipennis|HPPPA142-13|Canada|BOLD_AAH3983
Ctenosciara hyalipennis|HPPPA094-13|Canada|BOLD_AAH3983
Ctenosciara hyalipennis|HPPPA086-13|Canada|BOLD_AAH3983
Ctenosciara hyalipennis|HPPPA048-13|Canada|BOLD_AAH3983

Ctenosciara hyalipennis|HPPPA094-13|Canada|BOLD_AAH3983
Ctenosciara hyalipennis|HPPPA086-13|Canada|BOLD_AAH3983
Ctenosciara hyalipennis|HPPPA048-13|Canada|BOLD_AAH3983
Ctenosciara hyalipennis|HPPPA029-13|Canada|BOLD_AAH3983
Ctenosciara hyalipennis|CNBRQ627-14|Canada|BOLD_AAH3983
Ctenosciara hyalipennis|CNBRQ625-14|Canada|BOLD_AAH3983
Ctenosciara hyalipennis|CNBRQ610-14|Canada|BOLD_AAH3983
Ctenosciara hyalipennis|CNBRQ602-14|Canada|BOLD_AAH3983
Ctenosciara hyalipennis|CNBRQ601-14|Canada|BOLD_AAH3983
Ctenosciara hyalipennis|CNBRQ599-14|Canada|BOLD_AAH3983
Ctenosciara hyalipennis|CNBRQ585-14|Canada|BOLD_AAH3983
Ctenosciara hyalipennis|CNBRQ567-14|Canada|BOLD_AAH3983
Ctenosciara hyalipennis|CNBRQ511-14|Canada|BOLD_AAH3983
Ctenosciara hyalipennis|CNBRQ474-14|Canada|BOLD_AAH3983
Ctenosciara hyalipennis|CNBRQ464-14|Canada|BOLD_AAH3983
Ctenosciara hyalipennis|CNBRQ462-14|Canada|BOLD_AAH3983
Ctenosciara hyalipennis|CNBRQ211-14|Canada|BOLD_AAH3983
Ctenosciara hyalipennis|CNBRQ190-14|Canada|BOLD_AAH3983
Ctenosciara hyalipennis|CNBRQ189-14|Canada|BOLD_AAH3983
Ctenosciara hyalipennis|CNBRP997-14|Canada|BOLD_AAH3983
Ctenosciara hyalipennis|CNBRP969-14|Canada|BOLD_AAH3983
Ctenosciara hyalipennis|CNBRP930-14|Canada|BOLD_AAH3983
Ctenosciara hyalipennis|CNBRP746-14|Canada|BOLD_AAH3983
Ctenosciara hyalipennis|CNBRP635-14|Canada|BOLD_AAH3983
Ctenosciara hyalipennis|CNBRP585-14|Canada|BOLD_AAH3983
Ctenosciara hyalipennis|CNBRP560-14|Canada|BOLD_AAH3983
Ctenosciara hyalipennis|CNBRP507-14|Canada|BOLD_AAH3983
Ctenosciara hyalipennis|CNBRP470-14|Canada|BOLD_AAH3983
Ctenosciara hyalipennis|CNBRP460-14|Canada|BOLD_AAH3983
Ctenosciara hyalipennis|CNBRP452-14|Canada|BOLD_AAH3983
Ctenosciara hyalipennis|CNBRP360-14|Canada|BOLD_AAH3983
Ctenosciara hyalipennis|CNBRP345-14|Canada|BOLD_AAH3983
Ctenosciara hyalipennis|CNBRP229-14|Canada|BOLD_AAH3983
Ctenosciara hyalipennis|CNBRP228-14|Canada|BOLD_AAH3983
Ctenosciara hyalipennis|CNBRP041-14|Canada|BOLD_AAH3983
Ctenosciara hyalipennis|CNPEU107-14|Canada|BOLD_AAH3983
Ctenosciara hyalipennis|CNPEU086-14|Canada|BOLD_AAH3983
Ctenosciara hyalipennis|CNPES156-14|Canada|BOLD_AAH3983
Ctenosciara hyalipennis|CNPEQ466-14|Canada|BOLD_AAH3983
Ctenosciara hyalipennis|CNPEQ210-14|Canada|BOLD_AAH3983
Ctenosciara hyalipennis|CNPEQ201-14|Canada|BOLD_AAH3983
Ctenosciara hyalipennis|CNPEN732-14|Canada|BOLD_AAH3983
Ctenosciara hyalipennis|CNPEN623-14|Canada|BOLD_AAH3983
Ctenosciara hyalipennis|CNPEN597-14|Canada|BOLD_AAH3983
Ctenosciara hyalipennis|CNBRN170-14|Canada|BOLD_AAH3983
Ctenosciara hyalipennis|CNPEM1783-14|Canada|BOLD_AAH3983
Ctenosciara hyalipennis|CNPEM1750-14|Canada|BOLD_AAH3983
Ctenosciara hyalipennis|CNPEM1717-14|Canada|BOLD_AAH3983
Ctenosciara hyalipennis|CNBRL636-14|Canada|BOLD_AAH3983
Ctenosciara hyalipennis|CNBRL620-14|Canada|BOLD_AAH3983
Ctenosciara hyalipennis|CNBRL609-14|Canada|BOLD_AAH3983
Ctenosciara hyalipennis|CNBRL603-14|Canada|BOLD_AAH3983
Ctenosciara hyalipennis|CNBRL587-14|Canada|BOLD_AAH3983
Ctenosciara hyalipennis|CNBRL583-14|Canada|BOLD_AAH3983
Ctenosciara hyalipennis|CNBRL551-14|Canada|BOLD_AAH3983
Ctenosciara hyalipennis|CNBRL549-14|Canada|BOLD_AAH3983
Ctenosciara hyalipennis|CNBRL548-14|Canada|BOLD_AAH3983
Ctenosciara hyalipennis|CNBRL542-14|Canada|BOLD_AAH3983
Ctenosciara hyalipennis|CNBRL512-14|Canada|BOLD_AAH3983
Ctenosciara hyalipennis|CNBRL481-14|Canada|BOLD_AAH3983
Ctenosciara hyalipennis|CNPEL771-14|Canada|BOLD_AAH3983
Ctenosciara hyalipennis|CNPEL764-14|Canada|BOLD_AAH3983
Ctenosciara hyalipennis|CNPEL756-14|Canada|BOLD_AAH3983
Ctenosciara hyalipennis|CNPEL729-14|Canada|BOLD_AAH3983
Ctenosciara hyalipennis|CNPEL721-14|Canada|BOLD_AAH3983
Ctenosciara hyalipennis|CNPEL716-14|Canada|BOLD_AAH3983
Ctenosciara hyalipennis|CNPEL701-14|Canada|BOLD_AAH3983
Ctenosciara hyalipennis|CNBRK442-14|Canada|BOLD_AAH3983
Ctenosciara hyalipennis|CNBRK421-14|Canada|BOLD_AAH3983
Ctenosciara hyalipennis|CNBRK412-14|Canada|BOLD_AAH3983
Ctenosciara hyalipennis|CNBRK395-14|Canada|BOLD_AAH3983
Ctenosciara hyalipennis|CNBRK347-14|Canada|BOLD_AAH3983
Ctenosciara hyalipennis|CNBRK346-14|Canada|BOLD_AAH3983
Ctenosciara hyalipennis|CNBRK333-14|Canada|BOLD_AAH3983
Ctenosciara hyalipennis|CNBRK328-14|Canada|BOLD_AAH3983
Ctenosciara hyalipennis|CNBRK324-14|Canada|BOLD_AAH3983
Ctenosciara hyalipennis|CNBRK320-14|Canada|BOLD_AAH3983
Ctenosciara hyalipennis|CNBRK315-14|Canada|BOLD_AAH3983
Ctenosciara hyalipennis|CNBRK312-14|Canada|BOLD_AAH3983
Ctenosciara hyalipennis|CNBRK305-14|Canada|BOLD_AAH3983
Ctenosciara hyalipennis|CNBRK299-14|Canada|BOLD_AAH3983
Ctenosciara hyalipennis|CNBRK297-14|Canada|BOLD_AAH3983
Ctenosciara hyalipennis|CNBRK291-14|Canada|BOLD_AAH3983
Ctenosciara hyalipennis|CNBRK287-14|Canada|BOLD_AAH3983
Ctenosciara hyalipennis|CNBRK286-14|Canada|BOLD_AAH3983
Ctenosciara hyalipennis|CNBRK283-14|Canada|BOLD_AAH3983
Ctenosciara hyalipennis|CNBRK282-14|Canada|BOLD_AAH3983
Ctenosciara hyalipennis|CNBRK278-14|Canada|BOLD_AAH3983
Ctenosciara hyalipennis|CNBRF067-14|Canada|BOLD_AAH3983
Ctenosciara hyalipennis|CNPEF544-14|Canada|BOLD_AAH3983
Ctenosciara hyalipennis|CNPEF499-14|Canada|BOLD_AAH3983
Ctenosciara hyalipennis|CNPEF376-14|Canada|BOLD_AAH3983
Ctenosciara hyalipennis|CNPEF148-14|Canada|BOLD_AAH3983
Ctenosciara hyalipennis|CNBRG250-14|Canada|BOLD_AAH3983
Ctenosciara hyalipennis|CNBRG159-14|Canada|BOLD_AAH3983
Ctenosciara hyalipennis|CNBRG114-14|Canada|BOLD_AAH3983
Ctenosciara hyalipennis|CNPED378-14|Canada|BOLD_AAH3983
Ctenosciara hyalipennis|CNPEC1562-14|Canada|BOLD_AAH3983
Ctenosciara hyalipennis|CNPEC470-14|Canada|BOLD_AAH3983
Ctenosciara hyalipennis|CNPEB1746-14|Canada|BOLD_AAH3983
Ctenosciara hyalipennis|CNPEB1695-14|Canada|BOLD_AAH3983
Ctenosciara hyalipennis|CNPEB1692-14|Canada|BOLD_AAH3983

Ctenosciara hyalipennis|[illegible]|Canada|BOLD_AAH3983
Ctenosciara hyalipennis|CNPEB1746-14|Canada|BOLD_AAH3983
Ctenosciara hyalipennis|CNPEB1695-14|Canada|BOLD_AAH3983
Ctenosciara hyalipennis|CNPEB1692-14|Canada|BOLD_AAH3983
Ctenosciara hyalipennis|CNPEB1648-14|Canada|BOLD_AAH3983
Ctenosciara hyalipennis|CNPEB1564-14|Canada|BOLD_AAH3983
Ctenosciara hyalipennis|CNPEB1541-14|Canada|BOLD_AAH3983
Ctenosciara hyalipennis|CNPEB1366-14|Canada|BOLD_AAH3983
Ctenosciara hyalipennis|CNPEB1339-14|Canada|BOLD_AAH3983
Ctenosciara hyalipennis|CNPEB1243-14|Canada|BOLD_AAH3983
Ctenosciara hyalipennis|CNPEB1144-14|Canada|BOLD_AAH3983
Ctenosciara hyalipennis|CNPEB1089-14|Canada|BOLD_AAH3983
Ctenosciara hyalipennis|CNPEB1084-14|Canada|BOLD_AAH3983
Ctenosciara hyalipennis|CNPEB1082-14|Canada|BOLD_AAH3983
Ctenosciara hyalipennis|CNPEB1017-14|Canada|BOLD_AAH3983
Ctenosciara hyalipennis|CNPEB1007-14|Canada|BOLD_AAH3983
Ctenosciara hyalipennis|CNPEB977-14|Canada|BOLD_AAH3983
Ctenosciara hyalipennis|CNPEB974-14|Canada|BOLD_AAH3983
Ctenosciara hyalipennis|CNPEB905-14|Canada|BOLD_AAH3983
Ctenosciara hyalipennis|CNPEB818-14|Canada|BOLD_AAH3983
Ctenosciara hyalipennis|CNPEB794-14|Canada|BOLD_AAH3983
Ctenosciara hyalipennis|CNPEB758-14|Canada|BOLD_AAH3983
Ctenosciara hyalipennis|CNPEB757-14|Canada|BOLD_AAH3983
Ctenosciara hyalipennis|CNPEB700-14|Canada|BOLD_AAH3983
Ctenosciara hyalipennis|CNPEB699-14|Canada|BOLD_AAH3983
Ctenosciara hyalipennis|CNPEB648-14|Canada|BOLD_AAH3983
Ctenosciara hyalipennis|CNPEB625-14|Canada|BOLD_AAH3983
Ctenosciara hyalipennis|CNPEB624-14|Canada|BOLD_AAH3983
Ctenosciara hyalipennis|CNPEB620-14|Canada|BOLD_AAH3983
Ctenosciara hyalipennis|CNPEB614-14|Canada|BOLD_AAH3983
Ctenosciara hyalipennis|CNPEB600-14|Canada|BOLD_AAH3983
Ctenosciara hyalipennis|CNPEB580-14|Canada|BOLD_AAH3983
Ctenosciara hyalipennis|CNPEB533-14|Canada|BOLD_AAH3983
Ctenosciara hyalipennis|CNPEB521-14|Canada|BOLD_AAH3983
Ctenosciara hyalipennis|CNPEB333-14|Canada|BOLD_AAH3983
Ctenosciara hyalipennis|CNPEB313-14|Canada|BOLD_AAH3983
Ctenosciara hyalipennis|CNPEB245-14|Canada|BOLD_AAH3983
Ctenosciara hyalipennis|CNPEB241-14|Canada|BOLD_AAH3983
Ctenosciara hyalipennis|CNPEB193-14|Canada|BOLD_AAH3983
Ctenosciara hyalipennis|CNPEB173-14|Canada|BOLD_AAH3983
Ctenosciara hyalipennis|CNPEB146-14|Canada|BOLD_AAH3983
Ctenosciara hyalipennis|CNPEB138-14|Canada|BOLD_AAH3983
Ctenosciara hyalipennis|CNPEB134-14|Canada|BOLD_AAH3983
Ctenosciara hyalipennis|CNPEB129-14|Canada|BOLD_AAH3983
Ctenosciara hyalipennis|CNPEB118-14|Canada|BOLD_AAH3983
Ctenosciara hyalipennis|CNPEA1083-14|Canada|BOLD_AAH3983
Ctenosciara hyalipennis|CNPEA288-14|Canada|BOLD_AAH3983
Ctenosciara hyalipennis|CNBRD299-14|Canada|BOLD_AAH3983
Ctenosciara hyalipennis|CNBRC201-14|Canada|BOLD_AAH3983
Ctenosciara hyalipennis|CNBRC164-14|Canada|BOLD_AAH3983
Ctenosciara hyalipennis|CNBRC086-14|Canada|BOLD_AAH3983
Ctenosciara hyalipennis|CNBRC061-14|Canada|BOLD_AAH3983
Ctenosciara hyalipennis|CNBRC054-14|Canada|BOLD_AAH3983
Ctenosciara hyalipennis|CNBRC045-14|Canada|BOLD_AAH3983
Ctenosciara hyalipennis|CNBRB371-14|Canada|BOLD_AAH3983
Ctenosciara hyalipennis|CNBRB368-14|Canada|BOLD_AAH3983
Ctenosciara hyalipennis|CNBRB365-14|Canada|BOLD_AAH3983
Ctenosciara hyalipennis|CNBRB353-14|Canada|BOLD_AAH3983
Ctenosciara hyalipennis|CNBRB342-14|Canada|BOLD_AAH3983
Ctenosciara hyalipennis|CNBRB331-14|Canada|BOLD_AAH3983
Ctenosciara hyalipennis|CNBRB327-14|Canada|BOLD_AAH3983
Ctenosciara hyalipennis|CNBRB318-14|Canada|BOLD_AAH3983
Ctenosciara hyalipennis|CNBRB306-14|Canada|BOLD_AAH3983
Ctenosciara hyalipennis|CNBRB305-14|Canada|BOLD_AAH3983
Ctenosciara hyalipennis|CNBRB300-14|Canada|BOLD_AAH3983
Ctenosciara hyalipennis|CNBRB299-14|Canada|BOLD_AAH3983
Ctenosciara hyalipennis|CNBRB288-14|Canada|BOLD_AAH3983
Ctenosciara hyalipennis|CNBRB282-14|Canada|BOLD_AAH3983
Ctenosciara hyalipennis|CNBRB279-14|Canada|BOLD_AAH3983
Ctenosciara hyalipennis|CNBRB277-14|Canada|BOLD_AAH3983
Ctenosciara hyalipennis|CNBRB274-14|Canada|BOLD_AAH3983
Ctenosciara hyalipennis|CNBRB273-14|Canada|BOLD_AAH3983
Ctenosciara hyalipennis|CNBRB271-14|Canada|BOLD_AAH3983
Ctenosciara hyalipennis|CNBRB264-14|Canada|BOLD_AAH3983
Ctenosciara hyalipennis|CNBRB262-14|Canada|BOLD_AAH3983
Ctenosciara hyalipennis|CNBRB250-14|Canada|BOLD_AAH3983
Ctenosciara hyalipennis|CNBRB240-14|Canada|BOLD_AAH3983
Ctenosciara hyalipennis|CNBRB225-14|Canada|BOLD_AAH3983
Ctenosciara hyalipennis|CNBRB221-14|Canada|BOLD_AAH3983
Ctenosciara hyalipennis|CNBRB219-14|Canada|BOLD_AAH3983
Ctenosciara hyalipennis|CNBRB215-14|Canada|BOLD_AAH3983
Ctenosciara hyalipennis|CNBRB213-14|Canada|BOLD_AAH3983
Ctenosciara hyalipennis|CNBRB212-14|Canada|BOLD_AAH3983
Ctenosciara hyalipennis|CNBRB196-14|Canada|BOLD_AAH3983
Ctenosciara hyalipennis|CNBRB182-14|Canada|BOLD_AAH3983
Ctenosciara hyalipennis|CNBRB142-14|Canada|BOLD_AAH3983
Ctenosciara hyalipennis|CNBRB108-14|Canada|BOLD_AAH3983
Ctenosciara hyalipennis|CNBRA157-14|Canada|BOLD_AAH3983
Ctenosciara hyalipennis|CNBRA126-14|Canada|BOLD_AAH3983
Ctenosciara hyalipennis|CNBRA113-14|Canada|BOLD_AAH3983
Ctenosciara hyalipennis|CNBRA104-14|Canada|BOLD_AAH3983
Ctenosciara hyalipennis|CNBRA076-14|Canada|BOLD_AAH3983
Ctenosciara hyalipennis|CNBRA074-14|Canada|BOLD_AAH3983
Ctenosciara hyalipennis|CNBRA058-14|Canada|BOLD_AAH3983
Ctenosciara hyalipennis|CNBRA050-14|Canada|BOLD_AAH3983
Ctenosciara hyalipennis|CNBRA033-14|Canada|BOLD_AAH3983
Ctenosciara hyalipennis|CNBRA004-14|Canada|BOLD_AAH3983
Ctenosciara hyalipennis|CNPEF407-14|Canada|BOLD_AAH3983
Ctenosciara hyalipennis|CNBRA112-14|Canada|BOLD_AAH3983
Ctenosciara hyalipennis|CNBRI053-14|Canada|BOLD_AAH3983
Ctenosciara hyalipennis|HPPPS014-13|Canada|BOLD_AAH3983
Ctenosciara hyalipennis|GMGRG3513-13|Germany|BOLD_AAH3983
Ctenosciara hyalipennis|GMGRF3025-13|Germany|BOLD_AAH3983

Ctenosciara hyalipennis|CNBR[illegible]-14|Canada|BOLD_AAH3983
Ctenosciara hyalipennis|HPPPS014-13|Canada|BOLD_AAH3983
Ctenosciara hyalipennis|GMGRG3513-13|Germany|BOLD_AAH3983
Ctenosciara hyalipennis|GMGRF3025-13|Germany|BOLD_AAH3983
Ctenosciara hyalipennis|HPPPA306-13|Canada|BOLD_AAH3983
Ctenosciara hyalipennis|HPPPA200-13|Canada|BOLD_AAH3983
Ctenosciara hyalipennis|CNPEB1451-14|Canada|BOLD_AAH3983
Ctenosciara hyalipennis|CNPEB1396-14|Canada|BOLD_AAH3983
Ctenosciara hyalipennis|CNPEB1178-14|Canada|BOLD_AAH3983
Ctenosciara hyalipennis|CNPEB543-14|Canada|BOLD_AAH3983
Ctenosciara hyalipennis|HPPPJ966-13|Canada|BOLD_AAH3983
Ctenosciara hyalipennis|HPPPF778-13|Canada|BOLD_AAH3983
Ctenosciara hyalipennis|HPPPF468-13|Canada|BOLD_AAH3983
Ctenosciara hyalipennis|HPPPF433-13|Canada|BOLD_AAH3983
Ctenosciara hyalipennis|HPPPF159-13|Canada|BOLD_AAH3983
Ctenosciara hyalipennis|HPPPF154-13|Canada|BOLD_AAH3983
Ctenosciara hyalipennis|HPPPF089-13|Canada|BOLD_AAH3983
Ctenosciara hyalipennis|HPPPF046-13|Canada|BOLD_AAH3983
Ctenosciara hyalipennis|HPPPE690-13|Canada|BOLD_AAH3983
Ctenosciara hyalipennis|HPPPE628-13|Canada|BOLD_AAH3983
Ctenosciara hyalipennis|HPPPE560-13|Canada|BOLD_AAH3983
Ctenosciara hyalipennis|HPPPE524-13|Canada|BOLD_AAH3983
Ctenosciara hyalipennis|HPPPA459-13|Canada|BOLD_AAH3983
Ctenosciara hyalipennis|HPPPA444-13|Canada|BOLD_AAH3983
Ctenosciara hyalipennis|HPPPA438-13|Canada|BOLD_AAH3983
Ctenosciara hyalipennis|HPPPA420-13|Canada|BOLD_AAH3983
Ctenosciara hyalipennis|HPPPA401-13|Canada|BOLD_AAH3983
Ctenosciara hyalipennis|HPPPA396-13|Canada|BOLD_AAH3983
Ctenosciara hyalipennis|CNBRQ458-14|Canada|BOLD_AAH3983
Ctenosciara hyalipennis|SSBRB2763-14|Canada|BOLD_AAH3983
Ctenosciara hyalipennis|CNPEB1292-14|Canada|BOLD_AAH3983
Ctenosciara hyalipennis|CNPEB389-14|Canada|BOLD_AAH3983
Ctenosciara hyalipennis|HPPPS124-13|Canada|BOLD_AAH3983
Ctenosciara hyalipennis|HPPPS123-13|Canada|BOLD_AAH3983
Ctenosciara hyalipennis|HPPPS122-13|Canada|BOLD_AAH3983
Ctenosciara hyalipennis|HPPPS060-13|Canada|BOLD_AAH3983
Ctenosciara hyalipennis|HPPPS058-13|Canada|BOLD_AAH3983
Ctenosciara hyalipennis|HPPPK686-13|Canada|BOLD_AAH3983
Ctenosciara hyalipennis|HPPPK349-13|Canada|BOLD_AAH3983
Ctenosciara hyalipennis|HPPPK249-13|Canada|BOLD_AAH3983
Ctenosciara hyalipennis|HPPPJ957-13|Canada|BOLD_AAH3983
Ctenosciara hyalipennis|HPPPJ642-13|Canada|BOLD_AAH3983
Ctenosciara hyalipennis|HPPPJ391-13|Canada|BOLD_AAH3983
Ctenosciara hyalipennis|HPPPJ385-13|Canada|BOLD_AAH3983
Ctenosciara hyalipennis|HPPPJ375-13|Canada|BOLD_AAH3983
Ctenosciara hyalipennis|HPPPJ350-13|Canada|BOLD_AAH3983
Ctenosciara hyalipennis|HPPPJ337-13|Canada|BOLD_AAH3983
Ctenosciara hyalipennis|HPPPJ231-13|Canada|BOLD_AAH3983
Ctenosciara hyalipennis|HPPPJ136-13|Canada|BOLD_AAH3983
Ctenosciara hyalipennis|HPPPJ122-13|Canada|BOLD_AAH3983
Ctenosciara hyalipennis|HPPPJ114-13|Canada|BOLD_AAH3983
Ctenosciara hyalipennis|HPPPI284-13|Canada|BOLD_AAH3983
Ctenosciara hyalipennis|HPPPE1193-13|Canada|BOLD_AAH3983
Ctenosciara hyalipennis|HPPPE790-13|Canada|BOLD_AAH3983
Ctenosciara hyalipennis|HPPPE764-13|Canada|BOLD_AAH3983
Ctenosciara hyalipennis|HPPPE746-13|Canada|BOLD_AAH3983
Ctenosciara hyalipennis|HPPPE702-13|Canada|BOLD_AAH3983
Ctenosciara hyalipennis|HPPPE679-13|Canada|BOLD_AAH3983
Ctenosciara hyalipennis|HPPPE675-13|Canada|BOLD_AAH3983
Ctenosciara hyalipennis|HPPPE672-13|Canada|BOLD_AAH3983
Ctenosciara hyalipennis|HPPPE607-13|Canada|BOLD_AAH3983
Ctenosciara hyalipennis|HPPPE597-13|Canada|BOLD_AAH3983
Ctenosciara hyalipennis|HPPPE594-13|Canada|BOLD_AAH3983
Ctenosciara hyalipennis|HPPPE577-13|Canada|BOLD_AAH3983
Ctenosciara hyalipennis|HPPPA554-13|Canada|BOLD_AAH3983
Ctenosciara hyalipennis|HPPPA452-13|Canada|BOLD_AAH3983
Ctenosciara hyalipennis|HPPPA127-13|Canada|BOLD_AAH3983
Ctenosciara hyalipennis|CNPEL1433-14|Canada|BOLD_AAH3983
Ctenosciara hyalipennis|CNPEL1399-14|Canada|BOLD_AAH3983
Ctenosciara hyalipennis|CNPEL631-14|Canada|BOLD_AAH3983
Ctenosciara hyalipennis|CNPEL537-14|Canada|BOLD_AAH3983
Ctenosciara hyalipennis|CNPEL475-14|Canada|BOLD_AAH3983
Ctenosciara hyalipennis|CNPEL437-14|Canada|BOLD_AAH3983
Ctenosciara hyalipennis|CNPEL432-14|Canada|BOLD_AAH3983
Ctenosciara hyalipennis|HPPPJ386-13|Canada|BOLD_AAH3983
Ctenosciara hyalipennis|CNBRL479-14|Canada|BOLD_AAH3983
Ctenosciara hyalipennis|HPPPF666-13|Canada|BOLD_AAH3983
Ctenosciara hyalipennis|CNPEB1504-14|Canada|BOLD_AAH3983
Ctenosciara hyalipennis|CNPEB1472-14|Canada|BOLD_AAH3983
Ctenosciara hyalipennis|SSPEA326-15|Canada|BOLD_AAH3983
Ctenosciara hyalipennis|SSBRB3670-14|Canada|BOLD_AAH3983
Ctenosciara hyalipennis|SSBRB313-14|Canada|BOLD_AAH3983
Ctenosciara hyalipennis|SSBRB104-14|Canada|BOLD_AAH3983
Ctenosciara hyalipennis|SSBRB031-14|Canada|BOLD_AAH3983
Ctenosciara hyalipennis|SSBRA389-14|Canada|BOLD_AAH3983
Ctenosciara hyalipennis|CNPEL1846-14|Canada|BOLD_AAH3983
Ctenosciara hyalipennis|CNPEL789-14|Canada|BOLD_AAH3983
Ctenosciara hyalipennis|CNPEL368-14|Canada|BOLD_AAH3983
Ctenosciara hyalipennis|CNPEL267-14|Canada|BOLD_AAH3983
Ctenosciara hyalipennis|CNBRK258-14|Canada|BOLD_AAH3983
Ctenosciara hyalipennis|GMGRG908-13|Germany|BOLD_AAH3983
Ctenosciara hyalipennis|CNBRK265-14|Canada|BOLD_AAH3983
Ctenosciara hyalipennis|CNBRP393-14|Canada|BOLD_AAH3983
Ctenosciara hyalipennis|HPPPS062-13|Canada|BOLD_AAH3983
Ctenosciara hyalipennis|HPPPS057-13|Canada|BOLD_AAH3983
Ctenosciara hyalipennis|HPPPS054-13|Canada|BOLD_AAH3983
Ctenosciara hyalipennis|HPPPK659-13|Canada|BOLD_AAH3983
Ctenosciara hyalipennis|HPPPJ180-13|Canada|BOLD_AAH3983
Ctenosciara hyalipennis|HPPPJ154-13|Canada|BOLD_AAH3983
Ctenosciara hyalipennis|GMGRF1237-13|Germany|BOLD_AAH3983
Ctenosciara hyalipennis|HPPPE654-13|Canada|BOLD_AAH3983
Ctenosciara hyalipennis|CNBRB281-14|Canada|BOLD_AAH3983

Ctenosciara hyalipennis|GMGRF1237-13|Germany|BOLD_AAH3983
Ctenosciara hyalipennis|HPPPE654-13|Canada|BOLD_AAH3983
Ctenosciara hyalipennis|CNBRB281-14|Canada|BOLD_AAH3983
Ctenosciara hyalipennis|CNPEB1544-14|Canada|BOLD_AAH3983
Ctenosciara hyalipennis|HPPPA222-13|Canada|BOLD_AAH3983
Ctenosciara hyalipennis|CNPEL401-14|Canada|BOLD_AAH3983
Ctenosciara hyalipennis|HPPPF177-13|Canada|BOLD_AAH3983
Ctenosciara hyalipennis|SSBRB2775-14|Canada|BOLD_AAH3983
Ctenosciara hyalipennis|SSBRB2640-14|Canada|BOLD_AAH3983
Ctenosciara hyalipennis|CNBRS076-14|Canada|BOLD_AAH3983
Ctenosciara hyalipennis|CNBRK217-14|Canada|BOLD_AAH3983
Ctenosciara hyalipennis|HPPPA369-13|Canada|BOLD_AAH3983
Ctenosciara hyalipennis|CNBRQ644-14|Canada|BOLD_AAH3983
Ctenosciara hyalipennis|CNBRQ638-14|Canada|BOLD_AAH3983
Ctenosciara hyalipennis|CNBRP915-14|Canada|BOLD_AAH3983
Ctenosciara hyalipennis|CNBRP661-14|Canada|BOLD_AAH3983
Ctenosciara hyalipennis|CNBRP111-14|Canada|BOLD_AAH3983
Ctenosciara hyalipennis|CNBRO147-14|Canada|BOLD_AAH3983
Ctenosciara hyalipennis|CNPER180-14|Canada|BOLD_AAH3983
Ctenosciara hyalipennis|CNPEQ332-14|Canada|BOLD_AAH3983
Ctenosciara hyalipennis|CNPEQ328-14|Canada|BOLD_AAH3983
Ctenosciara hyalipennis|CNBRM478-14|Canada|BOLD_AAH3983
Ctenosciara hyalipennis|CNBRL655-14|Canada|BOLD_AAH3983
Ctenosciara hyalipennis|CNBRL605-14|Canada|BOLD_AAH3983
Ctenosciara hyalipennis|CNBRL598-14|Canada|BOLD_AAH3983
Ctenosciara hyalipennis|CNBRL588-14|Canada|BOLD_AAH3983
Ctenosciara hyalipennis|CNBRL576-14|Canada|BOLD_AAH3983
Ctenosciara hyalipennis|CNBRL573-14|Canada|BOLD_AAH3983
Ctenosciara hyalipennis|CNBRL511-14|Canada|BOLD_AAH3983
Ctenosciara hyalipennis|CNPEL752-14|Canada|BOLD_AAH3983
Ctenosciara hyalipennis|CNPEL751-14|Canada|BOLD_AAH3983
Ctenosciara hyalipennis|CNPEL706-14|Canada|BOLD_AAH3983
Ctenosciara hyalipennis|CNBRK348-14|Canada|BOLD_AAH3983
Ctenosciara hyalipennis|CNBRK318-14|Canada|BOLD_AAH3983
Ctenosciara hyalipennis|CNBRK304-14|Canada|BOLD_AAH3983
Ctenosciara hyalipennis|CNBRK303-14|Canada|BOLD_AAH3983
Ctenosciara hyalipennis|CNBRK302-14|Canada|BOLD_AAH3983
Ctenosciara hyalipennis|CNBRK301-14|Canada|BOLD_AAH3983
Ctenosciara hyalipennis|CNPEF481-14|Canada|BOLD_AAH3983
Ctenosciara hyalipennis|CNBRG032-14|Canada|BOLD_AAH3983
Ctenosciara hyalipennis|CNBRG013-14|Canada|BOLD_AAH3983
Ctenosciara hyalipennis|CNPED425-14|Canada|BOLD_AAH3983
Ctenosciara hyalipennis|CNPED099-14|Canada|BOLD_AAH3983
Ctenosciara hyalipennis|CNPEC1548-14|Canada|BOLD_AAH3983
Ctenosciara hyalipennis|CNPEB1718-14|Canada|BOLD_AAH3983
Ctenosciara hyalipennis|CNPEB1715-14|Canada|BOLD_AAH3983
Ctenosciara hyalipennis|CNPEB1686-14|Canada|BOLD_AAH3983
Ctenosciara hyalipennis|CNPEB1681-14|Canada|BOLD_AAH3983
Ctenosciara hyalipennis|CNPEB1659-14|Canada|BOLD_AAH3983
Ctenosciara hyalipennis|CNPEB1634-14|Canada|BOLD_AAH3983
Ctenosciara hyalipennis|CNPEB1609-14|Canada|BOLD_AAH3983
Ctenosciara hyalipennis|CNPEB1593-14|Canada|BOLD_AAH3983
Ctenosciara hyalipennis|CNPEB1590-14|Canada|BOLD_AAH3983
Ctenosciara hyalipennis|CNPEB1030-14|Canada|BOLD_AAH3983
Ctenosciara hyalipennis|CNPEB874-14|Canada|BOLD_AAH3983
Ctenosciara hyalipennis|CNPEB803-14|Canada|BOLD_AAH3983
Ctenosciara hyalipennis|CNPEB747-14|Canada|BOLD_AAH3983
Ctenosciara hyalipennis|CNPEB702-14|Canada|BOLD_AAH3983
Ctenosciara hyalipennis|CNPEB186-14|Canada|BOLD_AAH3983
Ctenosciara hyalipennis|CNPEB164-14|Canada|BOLD_AAH3983
Ctenosciara hyalipennis|CNPEB066-14|Canada|BOLD_AAH3983
Ctenosciara hyalipennis|CNPEB029-14|Canada|BOLD_AAH3983
Ctenosciara hyalipennis|CNBRC189-14|Canada|BOLD_AAH3983
Ctenosciara hyalipennis|CNBRB347-14|Canada|BOLD_AAH3983
Ctenosciara hyalipennis|CNBRB343-14|Canada|BOLD_AAH3983
Ctenosciara hyalipennis|CNBRB329-14|Canada|BOLD_AAH3983
Ctenosciara hyalipennis|CNBRB321-14|Canada|BOLD_AAH3983
Ctenosciara hyalipennis|CNBRB315-14|Canada|BOLD_AAH3983
Ctenosciara hyalipennis|CNBRB314-14|Canada|BOLD_AAH3983
Ctenosciara hyalipennis|CNBRB293-14|Canada|BOLD_AAH3983
Ctenosciara hyalipennis|CNBRB286-14|Canada|BOLD_AAH3983
Ctenosciara hyalipennis|CNBRB239-14|Canada|BOLD_AAH3983
Ctenosciara hyalipennis|CNBRA166-14|Canada|BOLD_AAH3983
Ctenosciara hyalipennis|CNBRA129-14|Canada|BOLD_AAH3983
Ctenosciara hyalipennis|CNBRA118-14|Canada|BOLD_AAH3983
Ctenosciara hyalipennis|CNBRA080-14|Canada|BOLD_AAH3983
Ctenosciara hyalipennis|CNPEL210-14|Canada|BOLD_AAH3983
Ctenosciara hyalipennis|CNBRB267-14|Canada|BOLD_AAH3983
Ctenosciara hyalipennis|SSBRC2228-14|Canada|BOLD_AAH3983
Ctenosciara hyalipennis|SSBRC1383-14|Canada|BOLD_AAH3983
Ctenosciara hyalipennis|SSBRA854-14|Canada|BOLD_AAH3983
Ctenosciara hyalipennis|SSBRB201-14|Canada|BOLD_AAH3983
Ctenosciara hyalipennis|SSBRB172-14|Canada|BOLD_AAH3983
Ctenosciara hyalipennis|CNPEL1577-14|Canada|BOLD_AAH3983
Ctenosciara hyalipennis|CNPEL105-14|Canada|BOLD_AAH3983
Ctenosciara hyalipennis|CNBRP939-14|Canada|BOLD_AAH3983
Ctenosciara hyalipennis|CNPEQ376-14|Canada|BOLD_AAH3983
Ctenosciara hyalipennis|CNPEL753-14|Canada|BOLD_AAH3983
Ctenosciara hyalipennis|CNBRK399-14|Canada|BOLD_AAH3983
Ctenosciara hyalipennis|CNBRK397-14|Canada|BOLD_AAH3983
Ctenosciara hyalipennis|CNPEB1713-14|Canada|BOLD_AAH3983
Ctenosciara hyalipennis|CNPEB1195-14|Canada|BOLD_AAH3983
Ctenosciara hyalipennis|CNPEB628-14|Canada|BOLD_AAH3983
Ctenosciara hyalipennis|CNPEB457-14|Canada|BOLD_AAH3983
Ctenosciara hyalipennis|CNPEB297-14|Canada|BOLD_AAH3983
Ctenosciara hyalipennis|CNPEB039-14|Canada|BOLD_AAH3983
Ctenosciara hyalipennis|CNBRF635-14|Canada|BOLD_AAH3983
Ctenosciara hyalipennis|HPPPJ614-13|Canada|BOLD_AAH3983
Ctenosciara hyalipennis|HPPPJ602-13|Canada|BOLD_AAH3983
Ctenosciara hyalipennis|HPPPJ390-13|Canada|BOLD_AAH3983
Ctenosciara hyalipennis|HPPPJ364-13|Canada|BOLD_AAH3983
Ctenosciara hyalipennis|HPPPJ351-13|Canada|BOLD_AAH3983

Ctenosciara hyalipennis|HPPPJ390-13|Canada|BOLD_AAH3983
Ctenosciara hyalipennis|HPPPJ364-13|Canada|BOLD_AAH3983
Ctenosciara hyalipennis|HPPPJ351-13|Canada|BOLD_AAH3983
Ctenosciara hyalipennis|HPPPJ189-13|Canada|BOLD_AAH3983
Ctenosciara hyalipennis|HPPPJ003-13|Canada|BOLD_AAH3983
Ctenosciara hyalipennis|HPPPE1198-13|Canada|BOLD_AAH3983
Ctenosciara hyalipennis|HPPPE778-13|Canada|BOLD_AAH3983
Ctenosciara hyalipennis|HPPPE532-13|Canada|BOLD_AAH3983
Ctenosciara hyalipennis|CNBRQ812-14|Canada|BOLD_AAH3983
Ctenosciara hyalipennis|CNBRQ597-14|Canada|BOLD_AAH3983
Ctenosciara hyalipennis|CNBRQ565-14|Canada|BOLD_AAH3983
Ctenosciara hyalipennis|CNBRQ481-14|Canada|BOLD_AAH3983
Ctenosciara hyalipennis|CNBRQ468-14|Canada|BOLD_AAH3983
Ctenosciara hyalipennis|CNBRP472-14|Canada|BOLD_AAH3983
Ctenosciara hyalipennis|CNBRN221-14|Canada|BOLD_AAH3983
Ctenosciara hyalipennis|CNBRN165-14|Canada|BOLD_AAH3983
Ctenosciara hyalipennis|CNBRL590-14|Canada|BOLD_AAH3983
Ctenosciara hyalipennis|CNPEL726-14|Canada|BOLD_AAH3983
Ctenosciara hyalipennis|CNBRK343-14|Canada|BOLD_AAH3983
Ctenosciara hyalipennis|CNBRK339-14|Canada|BOLD_AAH3983
Ctenosciara hyalipennis|CNBRK309-14|Canada|BOLD_AAH3983
Ctenosciara hyalipennis|CNBRK296-14|Canada|BOLD_AAH3983
Ctenosciara hyalipennis|CNPEB396-14|Canada|BOLD_AAH3983
Ctenosciara hyalipennis|CNPEB264-14|Canada|BOLD_AAH3983
Ctenosciara hyalipennis|CNBRB340-14|Canada|BOLD_AAH3983
Ctenosciara hyalipennis|CNBRB186-14|Canada|BOLD_AAH3983
Ctenosciara hyalipennis|CNBRB176-14|Canada|BOLD_AAH3983
Ctenosciara hyalipennis|CNBRB175-14|Canada|BOLD_AAH3983
Ctenosciara hyalipennis|CNBRB153-14|Canada|BOLD_AAH3983
Ctenosciara hyalipennis|CNBRB152-14|Canada|BOLD_AAH3983
Ctenosciara hyalipennis|CNBRB144-14|Canada|BOLD_AAH3983
Ctenosciara hyalipennis|CNBRB122-14|Canada|BOLD_AAH3983
Ctenosciara hyalipennis|CNBRB110-14|Canada|BOLD_AAH3983
Ctenosciara hyalipennis|CNBRA168-14|Canada|BOLD_AAH3983
Ctenosciara hyalipennis|CNBRA158-14|Canada|BOLD_AAH3983
Ctenosciara hyalipennis|CNBRA140-14|Canada|BOLD_AAH3983
Ctenosciara hyalipennis|CNBRA069-14|Canada|BOLD_AAH3983
Ctenosciara hyalipennis|CNBRA043-14|Canada|BOLD_AAH3983
Ctenosciara hyalipennis|CNBRA038-14|Canada|BOLD_AAH3983
Ctenosciara hyalipennis|CNBRA001-14|Canada|BOLD_AAH3983
Ctenosciara hyalipennis|HPPPK339-13|Canada|BOLD_AAH3983
Ctenosciara hyalipennis|HPPPJ610-13|Canada|BOLD_AAH3983
Ctenosciara hyalipennis|HPPPJ383-13|Canada|BOLD_AAH3983
Ctenosciara hyalipennis|HPPPJ292-13|Canada|BOLD_AAH3983
Ctenosciara hyalipennis|HPPPJ202-13|Canada|BOLD_AAH3983
Ctenosciara hyalipennis|GMGRF1244-13|Germany|BOLD_AAH3983
Ctenosciara hyalipennis|HPPPF805-13|Canada|BOLD_AAH3983
Ctenosciara hyalipennis|HPPPF720-13|Canada|BOLD_AAH3983
Ctenosciara hyalipennis|HPPPF166-13|Canada|BOLD_AAH3983
Ctenosciara hyalipennis|HPPPE729-13|Canada|BOLD_AAH3983
Ctenosciara hyalipennis|HPPPE596-13|Canada|BOLD_AAH3983
Ctenosciara hyalipennis|HPPPA470-13|Canada|BOLD_AAH3983
Ctenosciara hyalipennis|HPPPA429-13|Canada|BOLD_AAH3983
Ctenosciara hyalipennis|HPPPA414-13|Canada|BOLD_AAH3983
Ctenosciara hyalipennis|HPPPA409-13|Canada|BOLD_AAH3983
Ctenosciara hyalipennis|HPPPA395-13|Canada|BOLD_AAH3983
Ctenosciara hyalipennis|HPPPA110-13|Canada|BOLD_AAH3983
Ctenosciara hyalipennis|CNPEB1370-14|Canada|BOLD_AAH3983
Ctenosciara hyalipennis|CNBRP714-14|Canada|BOLD_AAH3983
Ctenosciara hyalipennis|CNPEN721-14|Canada|BOLD_AAH3983
Ctenosciara hyalipennis|CNBRL637-14|Canada|BOLD_AAH3983
Ctenosciara hyalipennis|CNPEL747-14|Canada|BOLD_AAH3983
Ctenosciara hyalipennis|CNPEL719-14|Canada|BOLD_AAH3983
Ctenosciara hyalipennis|CNBRK335-14|Canada|BOLD_AAH3983
Ctenosciara hyalipennis|CNBRK331-14|Canada|BOLD_AAH3983
Ctenosciara hyalipennis|CNBRK317-14|Canada|BOLD_AAH3983
Ctenosciara hyalipennis|CNBRK300-14|Canada|BOLD_AAH3983
Ctenosciara hyalipennis|CNPEB1690-14|Canada|BOLD_AAH3983
Ctenosciara hyalipennis|CNPEB1548-14|Canada|BOLD_AAH3983
Ctenosciara hyalipennis|CNPEB126-14|Canada|BOLD_AAH3983
Ctenosciara hyalipennis|CNPEB062-14|Canada|BOLD_AAH3983
Ctenosciara hyalipennis|SSBRB264-14|Canada|BOLD_AAH3983
Ctenosciara hyalipennis|HPPPA327-13|Canada|BOLD_AAH3983
Ctenosciara hyalipennis|HPPPF568-13|Canada|BOLD_AAH3983
Ctenosciara hyalipennis|HPPPF567-13|Canada|BOLD_AAH3983
Ctenosciara hyalipennis|HPPPF478-13|Canada|BOLD_AAH3983
Ctenosciara hyalipennis|HPPPF172-13|Canada|BOLD_AAH3983
Ctenosciara hyalipennis|HPPPA460-13|Canada|BOLD_AAH3983
Ctenosciara hyalipennis|CNBRO423-14|Canada|BOLD_AAH3983
Ctenosciara hyalipennis|CNPEL1662-14|Canada|BOLD_AAH3983
Ctenosciara hyalipennis|CNBRL599-14|Canada|BOLD_AAH3983
Ctenosciara hyalipennis|HPPPK019-13|Canada|BOLD_AAH3983
Ctenosciara hyalipennis|HPPPB251-13|Canada|BOLD_AAH3983
Ctenosciara hyalipennis|HPPPA403-13|Canada|BOLD_AAH3983
Ctenosciara hyalipennis|CNBRQ229-14|Canada|BOLD_AAH3983
Ctenosciara hyalipennis|HPPPA164-13|Canada|BOLD_AAH3983
Ctenosciara hyalipennis|SSBRB444-14|Canada|BOLD_AAH3983
Ctenosciara hyalipennis|CNPEM418-14|Canada|BOLD_AAH3983
Ctenosciara hyalipennis|GMGRG3533-13|Germany|BOLD_AAH3983
Ctenosciara hyalipennis|CNPEC535-14|Canada|BOLD_AAH3983
Ctenosciara hyalipennis|CNPEL547-14|Canada|BOLD_AAH3983
Ctenosciara hyalipennis|CNPEL218-14|Canada|BOLD_AAH3983
Ctenosciara hyalipennis|CNBRK103-14|Canada|BOLD_AAH3983
Ctenosciara hyalipennis|HPPPA424-13|Canada|BOLD_AAH3983
Ctenosciara hyalipennis|CNBRN196-14|Canada|BOLD_AAH3983
Ctenosciara hyalipennis|CNBRM391-14|Canada|BOLD_AAH3983
Ctenosciara hyalipennis|CNPEB1646-14|Canada|BOLD_AAH3983
Ctenosciara hyalipennis|CNPEB912-14|Canada|BOLD_AAH3983
Ctenosciara hyalipennis|CNPEB861-14|Canada|BOLD_AAH3983
Ctenosciara hyalipennis|CNBRB362-14|Canada|BOLD_AAH3983
Ctenosciara hyalipennis|CNBRB304-14|Canada|BOLD_AAH3983
Ctenosciara hyalipennis|HPPPA122-13|Canada|BOLD_AAH3983

[illegible]
Ctenosciara hyalipennis|CNBRB362-14|Canada|BOLD_AAH3983
Ctenosciara hyalipennis|CNBRB304-14|Canada|BOLD_AAH3983
Ctenosciara hyalipennis|HPPPA122-13|Canada|BOLD_AAH3983
Ctenosciara hyalipennis|HPPPA118-13|Canada|BOLD_AAH3983
Ctenosciara hyalipennis|CNPEL505-14|Canada|BOLD_AAH3983
Ctenosciara hyalipennis|CNBRO264-14|Canada|BOLD_AAH3983
Ctenosciara hyalipennis|CNPEU157-14|Canada|BOLD_AAH3983
Ctenosciara hyalipennis|CNPER064-14|Canada|BOLD_AAH3983
Ctenosciara hyalipennis|CNPEB1132-14|Canada|BOLD_AAH3983
Ctenosciara hyalipennis|CNPEB996-14|Canada|BOLD_AAH3983
Ctenosciara hyalipennis|CNPEB942-14|Canada|BOLD_AAH3983
Ctenosciara hyalipennis|CNPEB838-14|Canada|BOLD_AAH3983
Ctenosciara hyalipennis|CNPEB595-14|Canada|BOLD_AAH3983
Ctenosciara hyalipennis|CNBRC107-14|Canada|BOLD_AAH3983
Ctenosciara hyalipennis|CNBRB359-14|Canada|BOLD_AAH3983
Ctenosciara hyalipennis|CNBRB333-14|Canada|BOLD_AAH3983
Ctenosciara hyalipennis|CNBRB257-14|Canada|BOLD_AAH3983
Ctenosciara hyalipennis|CNBRA023-14|Canada|BOLD_AAH3983
Ctenosciara hyalipennis|CNPEC1172-14|Canada|BOLD_AAH3983
Ctenosciara hyalipennis|CNPEB128-14|Canada|BOLD_AAH3983
Ctenosciara hyalipennis|HPPPF764-13|Canada|BOLD_AAH3983
Ctenosciara hyalipennis|CNPEG289-14|Canada|BOLD_AAH3983
Ctenosciara hyalipennis|CNBRB206-14|Canada|BOLD_AAH3983
Ctenosciara hyalipennis|SSBRB2643-14|Canada|BOLD_AAH3983
Ctenosciara hyalipennis|CNBRP550-14|Canada|BOLD_AAH3983
Ctenosciara hyalipennis|CNBRL659-14|Canada|BOLD_AAH3983
Ctenosciara hyalipennis|CNBRL568-14|Canada|BOLD_AAH3983
Ctenosciara hyalipennis|CNBRL559-14|Canada|BOLD_AAH3983
Ctenosciara hyalipennis|CNBRL493-14|Canada|BOLD_AAH3983
Ctenosciara hyalipennis|CNBRL470-14|Canada|BOLD_AAH3983
Ctenosciara hyalipennis|CNPEL527-14|Canada|BOLD_AAH3983
Ctenosciara hyalipennis|CNBRF484-14|Canada|BOLD_AAH3983
Ctenosciara hyalipennis|CNBRG129-14|Canada|BOLD_AAH3983
Ctenosciara hyalipennis|CNBRG020-14|Canada|BOLD_AAH3983
Ctenosciara hyalipennis|CNPEB1247-14|Canada|BOLD_AAH3983
Ctenosciara hyalipennis|CNPEB1126-14|Canada|BOLD_AAH3983
Ctenosciara hyalipennis|CNPEB954-14|Canada|BOLD_AAH3983
Ctenosciara hyalipennis|CNPEB937-14|Canada|BOLD_AAH3983
Ctenosciara hyalipennis|HPPPS059-13|Canada|BOLD_AAH3983
Ctenosciara hyalipennis|HPPPS050-13|Canada|BOLD_AAH3983
Ctenosciara hyalipennis|HPPPS046-13|Canada|BOLD_AAH3983
Ctenosciara hyalipennis|HPPPS045-13|Canada|BOLD_AAH3983
Ctenosciara hyalipennis|HPPPS041-13|Canada|BOLD_AAH3983
Ctenosciara hyalipennis|HPPPR093-13|Canada|BOLD_AAH3983
Ctenosciara hyalipennis|HPPPR086-13|Canada|BOLD_AAH3983
Ctenosciara hyalipennis|HPPPR046-13|Canada|BOLD_AAH3983
Ctenosciara hyalipennis|HPPPR026-13|Canada|BOLD_AAH3983
Ctenosciara hyalipennis|HPPPL322-13|Canada|BOLD_AAH3983
Ctenosciara hyalipennis|GMGRF3838-13|Germany|BOLD_AAH3983
Ctenosciara hyalipennis|HPPPK699-13|Canada|BOLD_AAH3983
Ctenosciara hyalipennis|HPPPK356-13|Canada|BOLD_AAH3983
Ctenosciara hyalipennis|HPPPK335-13|Canada|BOLD_AAH3983
Ctenosciara hyalipennis|HPPPK306-13|Canada|BOLD_AAH3983
Ctenosciara hyalipennis|HPPPK275-13|Canada|BOLD_AAH3983
Ctenosciara hyalipennis|HPPPK270-13|Canada|BOLD_AAH3983
Ctenosciara hyalipennis|HPPPK240-13|Canada|BOLD_AAH3983
Ctenosciara hyalipennis|HPPPK016-13|Canada|BOLD_AAH3983
Ctenosciara hyalipennis|HPPPK012-13|Canada|BOLD_AAH3983
Ctenosciara hyalipennis|HPPPJ967-13|Canada|BOLD_AAH3983
Ctenosciara hyalipennis|HPPPJ675-13|Canada|BOLD_AAH3983
Ctenosciara hyalipennis|HPPPJ663-13|Canada|BOLD_AAH3983
Ctenosciara hyalipennis|HPPPJ629-13|Canada|BOLD_AAH3983
Ctenosciara hyalipennis|HPPPJ626-13|Canada|BOLD_AAH3983
Ctenosciara hyalipennis|HPPPJ621-13|Canada|BOLD_AAH3983
Ctenosciara hyalipennis|HPPPJ616-13|Canada|BOLD_AAH3983
Ctenosciara hyalipennis|HPPPJ599-13|Canada|BOLD_AAH3983
Ctenosciara hyalipennis|HPPPJ598-13|Canada|BOLD_AAH3983
Ctenosciara hyalipennis|HPPPJ590-13|Canada|BOLD_AAH3983
Ctenosciara hyalipennis|HPPPJ388-13|Canada|BOLD_AAH3983
Ctenosciara hyalipennis|HPPPJ367-13|Canada|BOLD_AAH3983
Ctenosciara hyalipennis|HPPPJ230-13|Canada|BOLD_AAH3983
Ctenosciara hyalipennis|HPPPJ150-13|Canada|BOLD_AAH3983
Ctenosciara hyalipennis|HPPPJ144-13|Canada|BOLD_AAH3983
Ctenosciara hyalipennis|HPPPI871-13|Canada|BOLD_AAH3983
Ctenosciara hyalipennis|HPPPI477-13|Canada|BOLD_AAH3983
Ctenosciara hyalipennis|HPPPI225-13|Canada|BOLD_AAH3983
Ctenosciara hyalipennis|HPPPF822-13|Canada|BOLD_AAH3983
Ctenosciara hyalipennis|HPPPF768-13|Canada|BOLD_AAH3983
Ctenosciara hyalipennis|HPPPF731-13|Canada|BOLD_AAH3983
Ctenosciara hyalipennis|HPPPF715-13|Canada|BOLD_AAH3983
Ctenosciara hyalipennis|HPPPF170-13|Canada|BOLD_AAH3983
Ctenosciara hyalipennis|HPPPF143-13|Canada|BOLD_AAH3983
Ctenosciara hyalipennis|HPPPE1246-13|Canada|BOLD_AAH3983
Ctenosciara hyalipennis|HPPPE1245-13|Canada|BOLD_AAH3983
Ctenosciara hyalipennis|HPPPE1225-13|Canada|BOLD_AAH3983
Ctenosciara hyalipennis|HPPPE1175-13|Canada|BOLD_AAH3983
Ctenosciara hyalipennis|HPPPE1172-13|Canada|BOLD_AAH3983
Ctenosciara hyalipennis|HPPPE1167-13|Canada|BOLD_AAH3983
Ctenosciara hyalipennis|HPPPE1165-13|Canada|BOLD_AAH3983
Ctenosciara hyalipennis|HPPPE1146-13|Canada|BOLD_AAH3983
Ctenosciara hyalipennis|HPPPE1142-13|Canada|BOLD_AAH3983
Ctenosciara hyalipennis|HPPPE1098-13|Canada|BOLD_AAH3983
Ctenosciara hyalipennis|HPPPE1087-13|Canada|BOLD_AAH3983
Ctenosciara hyalipennis|HPPPE1082-13|Canada|BOLD_AAH3983
Ctenosciara hyalipennis|HPPPE769-13|Canada|BOLD_AAH3983
Ctenosciara hyalipennis|HPPPE765-13|Canada|BOLD_AAH3983
Ctenosciara hyalipennis|HPPPE749-13|Canada|BOLD_AAH3983
Ctenosciara hyalipennis|HPPPE741-13|Canada|BOLD_AAH3983
Ctenosciara hyalipennis|HPPPE734-13|Canada|BOLD_AAH3983
Ctenosciara hyalipennis|HPPPE731-13|Canada|BOLD_AAH3983
Ctenosciara hyalipennis|HPPPE718-13|Canada|BOLD_AAH3983
[illegible]

Ctenosciara hyalipennis|HPPPE734-13|Canada|BOLD_AAH3983
Ctenosciara hyalipennis|HPPPE731-13|Canada|BOLD_AAH3983
Ctenosciara hyalipennis|HPPPE718-13|Canada|BOLD_AAH3983
Ctenosciara hyalipennis|HPPPE708-13|Canada|BOLD_AAH3983
Ctenosciara hyalipennis|HPPPE693-13|Canada|BOLD_AAH3983
Ctenosciara hyalipennis|HPPPE662-13|Canada|BOLD_AAH3983
Ctenosciara hyalipennis|HPPPE602-13|Canada|BOLD_AAH3983
Ctenosciara hyalipennis|HPPPE585-13|Canada|BOLD_AAH3983
Ctenosciara hyalipennis|HPPPE573-13|Canada|BOLD_AAH3983
Ctenosciara hyalipennis|HPPPE566-13|Canada|BOLD_AAH3983
Ctenosciara hyalipennis|HPPPE523-13|Canada|BOLD_AAH3983
Ctenosciara hyalipennis|HPPPE002-13|Canada|BOLD_AAH3983
Ctenosciara hyalipennis|HPPPD649-13|Canada|BOLD_AAH3983
Ctenosciara hyalipennis|HPPPB537-13|Canada|BOLD_AAH3983
Ctenosciara hyalipennis|HPPPB025-13|Canada|BOLD_AAH3983
Ctenosciara hyalipennis|HPPPA552-13|Canada|BOLD_AAH3983
Ctenosciara hyalipennis|HPPPA435-13|Canada|BOLD_AAH3983
Ctenosciara hyalipennis|HPPPA418-13|Canada|BOLD_AAH3983
Ctenosciara hyalipennis|HPPPA400-13|Canada|BOLD_AAH3983
Ctenosciara hyalipennis|HPPPA393-13|Canada|BOLD_AAH3983
Ctenosciara hyalipennis|HPPPA391-13|Canada|BOLD_AAH3983
Ctenosciara hyalipennis|HPPPA388-13|Canada|BOLD_AAH3983
Ctenosciara hyalipennis|HPPPA190-13|Canada|BOLD_AAH3983
Ctenosciara hyalipennis|HPPPA188-13|Canada|BOLD_AAH3983
Ctenosciara hyalipennis|HPPPA186-13|Canada|BOLD_AAH3983
Ctenosciara hyalipennis|HPPPA174-13|Canada|BOLD_AAH3983
Ctenosciara hyalipennis|HPPPA170-13|Canada|BOLD_AAH3983
Ctenosciara hyalipennis|HPPPA167-13|Canada|BOLD_AAH3983
Ctenosciara hyalipennis|HPPPA163-13|Canada|BOLD_AAH3983
Ctenosciara hyalipennis|HPPPA157-13|Canada|BOLD_AAH3983
Ctenosciara hyalipennis|HPPPA156-13|Canada|BOLD_AAH3983
Ctenosciara hyalipennis|HPPPA152-13|Canada|BOLD_AAH3983
Ctenosciara hyalipennis|HPPPA150-13|Canada|BOLD_AAH3983
Ctenosciara hyalipennis|HPPPA149-13|Canada|BOLD_AAH3983
Ctenosciara hyalipennis|HPPPA140-13|Canada|BOLD_AAH3983
Ctenosciara hyalipennis|HPPPA139-13|Canada|BOLD_AAH3983
Ctenosciara hyalipennis|HPPPA137-13|Canada|BOLD_AAH3983
Ctenosciara hyalipennis|HPPPA132-13|Canada|BOLD_AAH3983
Ctenosciara hyalipennis|HPPPA129-13|Canada|BOLD_AAH3983
Ctenosciara hyalipennis|HPPPA126-13|Canada|BOLD_AAH3983
Ctenosciara hyalipennis|HPPPA117-13|Canada|BOLD_AAH3983
Ctenosciara hyalipennis|HPPPA116-13|Canada|BOLD_AAH3983
Ctenosciara hyalipennis|HPPPA113-13|Canada|BOLD_AAH3983
Ctenosciara hyalipennis|HPPPA112-13|Canada|BOLD_AAH3983
Ctenosciara hyalipennis|HPPPA105-13|Canada|BOLD_AAH3983
Ctenosciara hyalipennis|HPPPA102-13|Canada|BOLD_AAH3983
Ctenosciara hyalipennis|HPPPA101-13|Canada|BOLD_AAH3983
Ctenosciara hyalipennis|HPPPA100-13|Canada|BOLD_AAH3983
Ctenosciara hyalipennis|HPPPA098-13|Canada|BOLD_AAH3983
Ctenosciara hyalipennis|CNBRN145-14|Canada|BOLD_AAH3983
Ctenosciara hyalipennis|CNPEM3034-14|Canada|BOLD_AAH3983
Ctenosciara hyalipennis|CNPEM2089-14|Canada|BOLD_AAH3983
Ctenosciara hyalipennis|CNPEL699-14|Canada|BOLD_AAH3983
Ctenosciara hyalipennis|CNBRK439-14|Canada|BOLD_AAH3983
Ctenosciara hyalipennis|CNBRK363-14|Canada|BOLD_AAH3983
Ctenosciara hyalipennis|CNBRK362-14|Canada|BOLD_AAH3983
Ctenosciara hyalipennis|CNPEC398-14|Canada|BOLD_AAH3983
Ctenosciara hyalipennis|CNPEB1551-14|Canada|BOLD_AAH3983
Ctenosciara hyalipennis|CNPEB755-14|Canada|BOLD_AAH3983
Ctenosciara hyalipennis|CNPEB248-14|Canada|BOLD_AAH3983
Ctenosciara hyalipennis|CNPEB211-14|Canada|BOLD_AAH3983
Ctenosciara hyalipennis|CNPEB200-14|Canada|BOLD_AAH3983
Ctenosciara hyalipennis|CNPEB151-14|Canada|BOLD_AAH3983
Ctenosciara hyalipennis|CNPEB137-14|Canada|BOLD_AAH3983
Ctenosciara hyalipennis|CNBRB349-14|Canada|BOLD_AAH3983
Ctenosciara hyalipennis|CNBRB346-14|Canada|BOLD_AAH3983
Ctenosciara hyalipennis|CNBRB162-14|Canada|BOLD_AAH3983
Ctenosciara hyalipennis|CNBRB146-14|Canada|BOLD_AAH3983
Ctenosciara hyalipennis|CNBRB134-14|Canada|BOLD_AAH3983
Ctenosciara hyalipennis|CNBRB133-14|Canada|BOLD_AAH3983
Ctenosciara hyalipennis|CNBRA156-14|Canada|BOLD_AAH3983
Ctenosciara hyalipennis|CNBRA144-14|Canada|BOLD_AAH3983
Ctenosciara hyalipennis|CNBRA055-14|Canada|BOLD_AAH3983
Ctenosciara hyalipennis|CNBRK266-14|Canada|BOLD_AAH3983
Ctenosciara hyalipennis|CNPEB250-14|Canada|BOLD_AAH3983
Ctenosciara hyalipennis|HPPPK254-13|Canada|BOLD_AAH3983
Ctenosciara hyalipennis|HPPPF519-13|Canada|BOLD_AAH3983
Ctenosciara hyalipennis|HPPPE548-13|Canada|BOLD_AAH3983
Ctenosciara hyalipennis|HPPPF058-13|Canada|BOLD_AAH3983
Ctenosciara hyalipennis|SSBRB3218-14|Canada|BOLD_AAH3983
Ctenosciara hyalipennis|SSBRA397-14|Canada|BOLD_AAH3983
Ctenosciara hyalipennis|CNPEB884-14|Canada|BOLD_AAH3983
Ctenosciara hyalipennis|CNBRB158-14|Canada|BOLD_AAH3983
Ctenosciara hyalipennis|CNBRQ323-14|Canada|BOLD_AAH3983
Ctenosciara hyalipennis|HPPPF477-13|Canada|BOLD_AAH3983
Ctenosciara hyalipennis|HPPPA387-13|Canada|BOLD_AAH3983
Ctenosciara hyalipennis|CNPEQ019-14|Canada|BOLD_AAH3983
Ctenosciara hyalipennis|CNBRK344-14|Canada|BOLD_AAH3983
Ctenosciara hyalipennis|CNPEF724-14|Canada|BOLD_AAH3983
Ctenosciara hyalipennis|CNBRN122-14|Canada|BOLD_AAH3983
Ctenosciara hyalipennis|HPPPK456-13|Canada|BOLD_AAH3983
Ctenosciara hyalipennis|GMGRF1619-13|Germany|BOLD_AAH3983
Ctenosciara hyalipennis|SSBRA1102-14|Canada|BOLD_AAH3983
Ctenosciara hyalipennis|SSPEA329-15|Canada|BOLD_AAH3983
Ctenosciara hyalipennis|GMGMD669-14|Germany|BOLD_AAH3983
Ctenosciara hyalipennis|SSBRB2725-14|Canada|BOLD_AAH3983
Ctenosciara hyalipennis|SSBRB1682-14|Canada|BOLD_AAH3983
Ctenosciara hyalipennis|SSBRA792-14|Canada|BOLD_AAH3983
Ctenosciara hyalipennis|SSBRA733-14|Canada|BOLD_AAH3983
Ctenosciara hyalipennis|SSBRA711-14|Canada|BOLD_AAH3983
Ctenosciara hyalipennis|SSBRA692-14|Canada|BOLD_AAH3983
Ctenosciara hyalipennis|SSBRB400-14|Canada|BOLD_AAH3983

Ctenosciara hyalipennis|SSBRA711-14|Canada|BOLD_AAH3983
Ctenosciara hyalipennis|SSBRA692-14|Canada|BOLD_AAH3983
Ctenosciara hyalipennis|SSBRB400-14|Canada|BOLD_AAH3983
Ctenosciara hyalipennis|SSBRB220-14|Canada|BOLD_AAH3983
Ctenosciara hyalipennis|SSBRB213-14|Canada|BOLD_AAH3983
Ctenosciara hyalipennis|SSBRB147-14|Canada|BOLD_AAH3983
Ctenosciara hyalipennis|SSBRB136-14|Canada|BOLD_AAH3983
Ctenosciara hyalipennis|SSBRB044-14|Canada|BOLD_AAH3983
Ctenosciara hyalipennis|CNPEM770-14|Canada|BOLD_AAH3983
Ctenosciara hyalipennis|CNPEL1889-14|Canada|BOLD_AAH3983
Ctenosciara hyalipennis|CNPEL1597-14|Canada|BOLD_AAH3983
Ctenosciara hyalipennis|CNPEL1584-14|Canada|BOLD_AAH3983
Ctenosciara hyalipennis|CNBRK123-14|Canada|BOLD_AAH3983
Ctenosciara hyalipennis|CNBRK115-14|Canada|BOLD_AAH3983
Ctenosciara hyalipennis|HPPPF847-13|Canada|BOLD_AAH3983
Ctenosciara hyalipennis|CNPEL1365-14|Canada|BOLD_AAH3983
Ctenosciara hyalipennis|SSBRB225-14|Canada|BOLD_AAH3983
Ctenosciara hyalipennis|CNPEL700-14|Canada|BOLD_AAH3983
Ctenosciara hyalipennis|HPPPJ376-13|Canada|BOLD_AAH3983
Ctenosciara hyalipennis|CNPED240-14|Canada|BOLD_AAH3983
Ctenosciara hyalipennis|CNPEL099-14|Canada|BOLD_AAH3983
Ctenosciara hyalipennis|HPPPA047-13|Canada|BOLD_AAH3983
Ctenosciara hyalipennis|HPPPF006-13|Canada|BOLD_AAH3983
Ctenosciara hyalipennis|CNPEM1482-14|Canada|BOLD_AAH3983
Ctenosciara hyalipennis|CNBRB181-14|Canada|BOLD_AAH3983
Ctenosciara hyalipennis|CNPEL216-14|Canada|BOLD_AAH3983
Ctenosciara hyalipennis|SSBRB519-14|Canada|BOLD_AAH3983
Ctenosciara hyalipennis|CNBRB116-14|Canada|BOLD_AAH3983
Ctenosciara hyalipennis|CNPEL189-14|Canada|BOLD_AAH3983
Ctenosciara hyalipennis|CNPEL046-14|Canada|BOLD_AAH3983
Ctenosciara hyalipennis|CNPEM593-14|Canada|BOLD_AAH3983
Ctenosciara hyalipennis|CNPEL1282-14|Canada|BOLD_AAH3983
Ctenosciara hyalipennis|CNPEL950-14|Canada|BOLD_AAH3983
Ctenosciara hyalipennis|CNPEL059-14|Canada|BOLD_AAH3983
Ctenosciara hyalipennis|CNPEL056-14|Canada|BOLD_AAH3983
Ctenosciara hyalipennis|CNBRK126-14|Canada|BOLD_AAH3983
Ctenosciara hyalipennis|HPPPA202-13|Canada|BOLD_AAH3983
Ctenosciara hyalipennis|CNBRQ446-14|Canada|
Ctenosciara hyalipennis|CNBRN242-14|Canada|
Ctenosciara hyalipennis|HPPPE762-13|Canada|BOLD_AAH3983
Ctenosciara hyalipennis|CNBRB199-14|Canada|BOLD_AAH3983
Ctenosciara hyalipennis|SSPEA423-15|Canada|BOLD_AAH3983
Ctenosciara hyalipennis|HPPPF040-13|Canada|BOLD_AAH3983
Ctenosciara hyalipennis|GMGRF862-13|Germany|BOLD_AAH3983
Ctenosciara hyalipennis|HPPPG005-13|Canada|BOLD_AAH3983
Ctenosciara hyalipennis|HPPPM279-13|Canada|BOLD_AAH3983
Ctenosciara hyalipennis|CNBRK166-14|Canada|BOLD_AAH3983
Ctenosciara hyalipennis|SSBRB2728-14|Canada|BOLD_AAH3983
Ctenosciara hyalipennis|CNPEM3017-14|Canada|BOLD_AAH3983
Ctenosciara hyalipennis|CNPEM3016-14|Canada|BOLD_AAH3983
Ctenosciara hyalipennis|GMGRG2294-13|Germany|BOLD_AAH3983
Ctenosciara hyalipennis|CNPEB166-14|Canada|BOLD_AAH3983
Ctenosciara hyalipennis|GMGRF1489-13|Germany|BOLD_AAH3983
Ctenosciara hyalipennis|HPPPE583-13|Canada|BOLD_AAH3983
Ctenosciara hyalipennis|HPPPE780-13|Canada|BOLD_AAH3983
Ctenosciara hyalipennis|CNBRB307-14|Canada|BOLD_AAH3983
Ctenosciara hyalipennis|CNBRB254-14|Canada|BOLD_AAH3983
Ctenosciara hyalipennis|CNBRA164-14|Canada|BOLD_AAH3983
Ctenosciara hyalipennis|HPPPF109-13|Canada|BOLD_AAH3983
Ctenosciara hyalipennis|CNPEL1909-14|Canada|BOLD_AAH3983
Ctenosciara hyalipennis|CNBRN135-14|Canada|BOLD_AAH3983
Ctenosciara hyalipennis|CNPEB1208-14|Canada|BOLD_AAH3983
Ctenosciara hyalipennis|CNBRK113-14|Canada|BOLD_AAH3983
Ctenosciara hyalipennis|CNBRJ122-14|Canada|BOLD_AAH3983
Ctenosciara hyalipennis|CNPEL192-14|Canada|BOLD_AAH3983
Ctenosciara hyalipennis|CNPEL183-14|Canada|BOLD_AAH3983
Ctenosciara hyalipennis|CNPEL170-14|Canada|BOLD_AAH3983
Ctenosciara hyalipennis|CNPEL156-14|Canada|BOLD_AAH3983
Ctenosciara hyalipennis|CNPEL145-14|Canada|BOLD_AAH3983
Ctenosciara hyalipennis|CNPEL045-14|Canada|BOLD_AAH3983
Ctenosciara hyalipennis|CNPEL044-14|Canada|BOLD_AAH3983
Ctenosciara hyalipennis|CNPEE976-14|Canada|BOLD_AAH3983
Ctenosciara hyalipennis|HPPPM394-13|Canada|BOLD_AAH3983
Ctenosciara hyalipennis|HPPPJ596-13|Canada|BOLD_AAH3983
Ctenosciara hyalipennis|HPPPA166-13|Canada|BOLD_AAH3983
Ctenosciara hyalipennis|HPPPA134-13|Canada|BOLD_AAH3983
Ctenosciara hyalipennis|HPPPA123-13|Canada|BOLD_AAH3983
Ctenosciara hyalipennis|CNPEB225-14|Canada|BOLD_AAH3983
Ctenosciara hyalipennis|CNPEB125-14|Canada|BOLD_AAH3983
Ctenosciara hyalipennis|CNPEA751-14|Canada|BOLD_AAH3983
Ctenosciara hyalipennis|HPPPS125-13|Canada|BOLD_AAH3983
Ctenosciara hyalipennis|HPPPA225-13|Canada|BOLD_AAH3983
Ctenosciara hyalipennis|HPPPS133-14|Canada|BOLD_AAH3983
Ctenosciara hyalipennis|CNPEB1188-14|Canada|BOLD_AAH3983
Ctenosciara hyalipennis|HPPPK408-13|Canada|BOLD_AAH3983
Ctenosciara hyalipennis|HPPPF821-13|Canada|BOLD_AAH3983
Ctenosciara hyalipennis|HPPPF095-13|Canada|BOLD_AAH3983
Ctenosciara hyalipennis|CNBRK404-14|Canada|BOLD_AAH3983
Ctenosciara hyalipennis|CNPEB512-14|Canada|BOLD_AAH3983
Ctenosciara hyalipennis|CNBRL541-14|Canada|BOLD_AAH3983
Ctenosciara hyalipennis|HPPPK245-13|Canada|BOLD_AAH3983
Ctenosciara hyalipennis|HPPPA453-13|Canada|BOLD_AAH3983
Ctenosciara hyalipennis|SSBRB052-14|Canada|BOLD_AAH3983
Ctenosciara hyalipennis|HPPPL371-13|Canada|BOLD_AAH3983
Ctenosciara hyalipennis|HPPPF539-13|Canada|BOLD_AAH3983
Ctenosciara hyalipennis|HPPPA185-13|Canada|BOLD_AAH3983
Ctenosciara hyalipennis|HPPPA009-13|Canada|BOLD_AAH3983
Ctenosciara hyalipennis|CNBRB361-14|Canada|BOLD_AAH3983
Ctenosciara hyalipennis|HPPPJ139-13|Canada|BOLD_AAH3983
Ctenosciara hyalipennis|HPPPA270-13|Canada|BOLD_AAH3983
Ctenosciara hyalipennis|HPPPA073-13|Canada|BOLD_AAH3983
Ctenosciara hyalipennis|HPPPA040-13|Canada|BOLD_AAH3983

Ctenosciara hyalipennis|HPPPA270-13|Canada|BOLD_AAH3983
Ctenosciara hyalipennis|HPPPA073-13|Canada|BOLD_AAH3983
Ctenosciara hyalipennis|HPPPA040-13|Canada|BOLD_AAH3983
Ctenosciara hyalipennis|HPPPA032-13|Canada|BOLD_AAH3983
Ctenosciara hyalipennis|HPPPA019-13|Canada|BOLD_AAH3983
Ctenosciara hyalipennis|CNBRL520-14|Canada|BOLD_AAH3983
Ctenosciara hyalipennis|CNBRL463-14|Canada|BOLD_AAH3983
Ctenosciara hyalipennis|CNPEL081-14|Canada|BOLD_AAH3983
Ctenosciara hyalipennis|CNBRB285-14|Canada|BOLD_AAH3983
Ctenosciara hyalipennis|CNBRB245-14|Canada|BOLD_AAH3983
Ctenosciara hyalipennis|HPPPK365-13|Canada|BOLD_AAH3983
Ctenosciara hyalipennis|HPPPJ633-13|Canada|BOLD_AAH3983
Ctenosciara hyalipennis|HPPPJ597-13|Canada|BOLD_AAH3983
Ctenosciara hyalipennis|HPPPE1169-13|Canada|BOLD_AAH3983
Ctenosciara hyalipennis|HPPPE530-13|Canada|BOLD_AAH3983
Ctenosciara hyalipennis|HPPPC458-13|Canada|BOLD_AAH3983
Ctenosciara hyalipennis|HPPPB354-13|Canada|BOLD_AAH3983
Ctenosciara hyalipennis|HPPPA555-13|Canada|BOLD_AAH3983
Ctenosciara hyalipennis|HPPPA176-13|Canada|BOLD_AAH3983
Ctenosciara hyalipennis|HPPPA165-13|Canada|BOLD_AAH3983
Ctenosciara hyalipennis|HPPPA153-13|Canada|BOLD_AAH3983
Ctenosciara hyalipennis|HPPPA147-13|Canada|BOLD_AAH3983
Ctenosciara hyalipennis|HPPPA121-13|Canada|BOLD_AAH3983
Ctenosciara hyalipennis|HPPPA115-13|Canada|BOLD_AAH3983
Ctenosciara hyalipennis|HPPPA114-13|Canada|BOLD_AAH3983
Ctenosciara hyalipennis|HPPPA108-13|Canada|BOLD_AAH3983
Ctenosciara hyalipennis|CNBRL658-14|Canada|BOLD_AAH3983
Ctenosciara hyalipennis|CNPEB689-14|Canada|BOLD_AAH3983
Ctenosciara hyalipennis|CNPEB484-14|Canada|BOLD_AAH3983
Ctenosciara hyalipennis|CNBRK139-14|Canada|BOLD_AAH3983
Ctenosciara hyalipennis|CNBRP1562-14|Canada|BOLD_AAH3983
Ctenosciara hyalipennis|CNPEB493-14|Canada|BOLD_AAH3983
Ctenosciara hyalipennis|HPPPK373-13|Canada|BOLD_AAH3983
Ctenosciara hyalipennis|HPPPK219-13|Canada|BOLD_AAH3983
Ctenosciara hyalipennis|HPPPK198-13|Canada|BOLD_AAH3983
Ctenosciara hyalipennis|HPPPA434-13|Canada|BOLD_AAH3983
Ctenosciara hyalipennis|HPPPA159-13|Canada|BOLD_AAH3983
Ctenosciara hyalipennis|CNPEN267-14|Canada|BOLD_AAH3983
Ctenosciara hyalipennis|HPPPA364-13|Canada|BOLD_AAH3983
Ctenosciara hyalipennis|HPPPA230-13|Canada|BOLD_AAH3983
Ctenosciara hyalipennis|HPPPA148-13|Canada|BOLD_AAH3983
Ctenosciara hyalipennis|HPPPE631-13|Canada|BOLD_AAH3983
Ctenosciara hyalipennis|CNPEQ411-14|Canada|BOLD_AAH3983
Ctenosciara hyalipennis|CNBRA152-14|Canada|BOLD_AAH3983
Ctenosciara hyalipennis|CNBRA002-14|Canada|BOLD_AAH3983
Ctenosciara hyalipennis|HPPPF061-13|Canada|BOLD_AAH3983
Ctenosciara hyalipennis|CNBRB270-14|Canada|BOLD_AAH3983
Ctenosciara hyalipennis|SSBRB2166-14|Canada|BOLD_AAH3983
Ctenosciara hyalipennis|SSBRB482-14|Canada|BOLD_AAH3983
Ctenosciara hyalipennis|SSBRB404-14|Canada|BOLD_AAH3983
Ctenosciara hyalipennis|CNPEM124-14|Canada|BOLD_AAH3983
Ctenosciara hyalipennis|CNPEM042-14|Canada|BOLD_AAH3983
Ctenosciara hyalipennis|CNBRJ129-14|Canada|BOLD_AAH3983
Ctenosciara hyalipennis|GMGRF3843-13|Germany|BOLD_AAH3983
Ctenosciara hyalipennis|SSBRB2700-14|Canada|BOLD_AAH3983
Ctenosciara hyalipennis|CNBRQ619-14|Canada|BOLD_AAH3983
Ctenosciara hyalipennis|CNPEL1854-14|Canada|BOLD_AAH3983
Ctenosciara hyalipennis|CNPEL1232-14|Canada|BOLD_AAH3983
Ctenosciara hyalipennis|CNPEL977-14|Canada|BOLD_AAH3983
Ctenosciara hyalipennis|CNPEL576-14|Canada|BOLD_AAH3983
Ctenosciara hyalipennis|CNPEL384-14|Canada|BOLD_AAH3983
Ctenosciara hyalipennis|CNBRK238-14|Canada|BOLD_AAH3983
Ctenosciara hyalipennis|CNBRK133-14|Canada|BOLD_AAH3983
Ctenosciara hyalipennis|CNPEB1121-14|Canada|BOLD_AAH3983
Ctenosciara hyalipennis|CNBRB291-14|Canada|BOLD_AAH3983
Ctenosciara hyalipennis|CNBRA137-14|Canada|BOLD_AAH3983
Ctenosciara hyalipennis|CNBRA009-14|Canada|BOLD_AAH3983
Ctenosciara hyalipennis|HPPPE1199-13|Canada|BOLD_AAH3983
Ctenosciara hyalipennis|HPPPE622-13|Canada|BOLD_AAH3983
Ctenosciara hyalipennis|CNPEQ542-14|Canada|BOLD_AAH3983
Ctenosciara hyalipennis|CNPEB777-14|Canada|BOLD_AAH3983
Ctenosciara hyalipennis|HPPPK340-13|Canada|BOLD_AAH3983
Ctenosciara hyalipennis|SSBRB1671-14|Canada|BOLD_AAH3983
Ctenosciara hyalipennis|CNBRP1208-14|Canada|BOLD_AAH3983
Ctenosciara hyalipennis|HPPPA302-13|Canada|BOLD_AAH3983
Ctenosciara hyalipennis|GMGRH557-13|Germany|BOLD_AAH3983
Ctenosciara hyalipennis|CNPEM3002-14|Canada|BOLD_AAH3983
Ctenosciara hyalipennis|CNBRP1912-14|Canada|BOLD_AAH3983
Ctenosciara hyalipennis|SSPEA427-15|Canada|BOLD_AAH3983
Ctenosciara hyalipennis|SSPEA302-15|Canada|BOLD_AAH3983
Ctenosciara hyalipennis|SSPEA240-15|Canada|BOLD_AAH3983
Ctenosciara hyalipennis|SSBRB3414-14|Canada|BOLD_AAH3983
Ctenosciara hyalipennis|SSBRB3298-14|Canada|BOLD_AAH3983
Ctenosciara hyalipennis|SSBRB3282-14|Canada|BOLD_AAH3983
Ctenosciara hyalipennis|SSBRB3254-14|Canada|BOLD_AAH3983
Ctenosciara hyalipennis|SSBRB3193-14|Canada|BOLD_AAH3983
Ctenosciara hyalipennis|SSBRB3066-14|Canada|BOLD_AAH3983
Ctenosciara hyalipennis|SSBRB2615-14|Canada|BOLD_AAH3983
Ctenosciara hyalipennis|SSBRB117-14|Canada|BOLD_AAH3983
Ctenosciara hyalipennis|CNBRP1190-14|Canada|BOLD_AAH3983
Ctenosciara hyalipennis|CNPEM1274-14|Canada|BOLD_AAH3983
Ctenosciara hyalipennis|CNBRJ074-14|Canada|BOLD_AAH3983
Ctenosciara hyalipennis|CNBRJ029-14|Canada|BOLD_AAH3983
Ctenosciara hyalipennis|CNPEL1898-14|Canada|BOLD_AAH3983
Ctenosciara hyalipennis|CNPEL1855-14|Canada|BOLD_AAH3983
Ctenosciara hyalipennis|CNPEL1845-14|Canada|BOLD_AAH3983
Ctenosciara hyalipennis|CNPEL1746-14|Canada|BOLD_AAH3983
Ctenosciara hyalipennis|CNPEL1715-14|Canada|BOLD_AAH3983
Ctenosciara hyalipennis|CNPEL1545-14|Canada|BOLD_AAH3983
Ctenosciara hyalipennis|CNPEL1290-14|Canada|BOLD_AAH3983
Ctenosciara hyalipennis|CNPEL1253-14|Canada|BOLD_AAH3983
Ctenosciara hyalipennis|CNPEL1247-14|Canada|BOLD_AAH3983

[illegible]
Ctenosciara hyalipennis|CNPEL1290-14|Canada|BOLD_AAH3983
Ctenosciara hyalipennis|CNPEL1253-14|Canada|BOLD_AAH3983
Ctenosciara hyalipennis|CNPEL1247-14|Canada|BOLD_AAH3983
Ctenosciara hyalipennis|CNPEL1221-14|Canada|BOLD_AAH3983
Ctenosciara hyalipennis|CNPEL1216-14|Canada|BOLD_AAH3983
Ctenosciara hyalipennis|CNPEL1177-14|Canada|BOLD_AAH3983
Ctenosciara hyalipennis|CNPEL1068-14|Canada|BOLD_AAH3983
Ctenosciara hyalipennis|CNPEL1038-14|Canada|BOLD_AAH3983
Ctenosciara hyalipennis|CNPEL1016-14|Canada|BOLD_AAH3983
Ctenosciara hyalipennis|CNPEL926-14|Canada|BOLD_AAH3983
Ctenosciara hyalipennis|CNPEL831-14|Canada|BOLD_AAH3983
Ctenosciara hyalipennis|CNPEL818-14|Canada|BOLD_AAH3983
Ctenosciara hyalipennis|CNPEL806-14|Canada|BOLD_AAH3983
Ctenosciara hyalipennis|CNPEL641-14|Canada|BOLD_AAH3983
Ctenosciara hyalipennis|CNPEL385-14|Canada|BOLD_AAH3983
Ctenosciara hyalipennis|CNPEL276-14|Canada|BOLD_AAH3983
Ctenosciara hyalipennis|CNBRK275-14|Canada|BOLD_AAH3983
Ctenosciara hyalipennis|CNBRK272-14|Canada|BOLD_AAH3983
Ctenosciara hyalipennis|CNBRK241-14|Canada|BOLD_AAH3983
Ctenosciara hyalipennis|CNBRK240-14|Canada|BOLD_AAH3983
Ctenosciara hyalipennis|CNBRK226-14|Canada|BOLD_AAH3983
Ctenosciara hyalipennis|CNBRK221-14|Canada|BOLD_AAH3983
Ctenosciara hyalipennis|CNBRK215-14|Canada|BOLD_AAH3983
Ctenosciara hyalipennis|CNBRK214-14|Canada|BOLD_AAH3983
Ctenosciara hyalipennis|CNBRK212-14|Canada|BOLD_AAH3983
Ctenosciara hyalipennis|CNBRK208-14|Canada|BOLD_AAH3983
Ctenosciara hyalipennis|CNBRK204-14|Canada|BOLD_AAH3983
Ctenosciara hyalipennis|CNBRK202-14|Canada|BOLD_AAH3983
Ctenosciara hyalipennis|CNBRK188-14|Canada|BOLD_AAH3983
Ctenosciara hyalipennis|CNBRK155-14|Canada|BOLD_AAH3983
Ctenosciara hyalipennis|CNBRK144-14|Canada|BOLD_AAH3983
Ctenosciara hyalipennis|CNBRK125-14|Canada|BOLD_AAH3983
Ctenosciara hyalipennis|CNBRK121-14|Canada|BOLD_AAH3983
Ctenosciara hyalipennis|CNBRK119-14|Canada|BOLD_AAH3983
Ctenosciara hyalipennis|CNBRH110-14|Canada|BOLD_AAH3983
Ctenosciara hyalipennis|HPPPS025-13|Canada|BOLD_AAH3983
Ctenosciara hyalipennis|GMGRG3700-13|Germany|BOLD_AAH3983
Ctenosciara hyalipennis|HPPPL029-13|Canada|BOLD_AAH3983
Ctenosciara hyalipennis|HPPPA252-13|Canada|BOLD_AAH3983
Ctenosciara hyalipennis|CNBRQ488-14|Canada|BOLD_AAH3983
Ctenosciara hyalipennis|CNBRG029-14|Canada|BOLD_AAH3983
Ctenosciara hyalipennis|CNBRQ411-14|Canada|BOLD_AAH3983
Ctenosciara hyalipennis|HPPPF383-13|Canada|BOLD_AAH3983
Ctenosciara hyalipennis|HPPPF193-13|Canada|BOLD_AAH3983
Ctenosciara hyalipennis|CNPEA1027-14|Canada|BOLD_AAH3983
Ctenosciara hyalipennis|CNPEA1026-14|Canada|BOLD_AAH3983
Ctenosciara hyalipennis|CNBRP1040-14|Canada|BOLD_AAH3983
Ctenosciara hyalipennis|CNBRN233-14|Canada|BOLD_AAH3983
Ctenosciara hyalipennis|CNBRK437-14|Canada|BOLD_AAH3983
Ctenosciara hyalipennis|CNBRK408-14|Canada|BOLD_AAH3983
Ctenosciara hyalipennis|CNBRK398-14|Canada|BOLD_AAH3983
Ctenosciara hyalipennis|CNBRK390-14|Canada|BOLD_AAH3983
Ctenosciara hyalipennis|CNPEB1745-14|Canada|BOLD_AAH3983
Ctenosciara hyalipennis|CNPEB546-14|Canada|BOLD_AAH3983
Ctenosciara hyalipennis|CNBRA060-14|Canada|BOLD_AAH3983
Ctenosciara hyalipennis|CNPEL139-14|Canada|BOLD_AAH3983
Ctenosciara hyalipennis|HPPPE772-13|Canada|BOLD_AAH3983
Ctenosciara hyalipennis|SSBRA797-14|Canada|BOLD_AAH3983
Ctenosciara hyalipennis|SSBRB3250-14|Canada|BOLD_AAH3983
Ctenosciara hyalipennis|SSBRB1343-14|Canada|BOLD_AAH3983
Ctenosciara hyalipennis|SSBRA836-14|Canada|BOLD_AAH3983
Ctenosciara hyalipennis|SSBRB470-14|Canada|BOLD_AAH3983
Ctenosciara hyalipennis|SSBRB144-14|Canada|BOLD_AAH3983
Ctenosciara hyalipennis|SSBRB135-14|Canada|BOLD_AAH3983
Ctenosciara hyalipennis|CNBRK164-14|Canada|BOLD_AAH3983
Ctenosciara hyalipennis|CNPEQ362-14|Canada|BOLD_AAH3983
Ctenosciara hyalipennis|SSBRC2841-14|Canada|BOLD_AAH3983
Ctenosciara hyalipennis|SSBRB2834-14|Canada|BOLD_AAH3983
Ctenosciara hyalipennis|SSBRB2826-14|Canada|BOLD_AAH3983
Ctenosciara hyalipennis|SSBRB2766-14|Canada|BOLD_AAH3983
Ctenosciara hyalipennis|SSBRB2693-14|Canada|BOLD_AAH3983
Ctenosciara hyalipennis|CNPEL545-14|Canada|BOLD_AAH3983
Ctenosciara hyalipennis|GMGRF3145-13|Germany|BOLD_AAH3983
Ctenosciara hyalipennis|HPPPK664-13|Canada|BOLD_AAH3983
Ctenosciara hyalipennis|HPPPK224-13|Canada|BOLD_AAH3983
Ctenosciara hyalipennis|HPPPJ389-13|Canada|BOLD_AAH3983
Ctenosciara hyalipennis|HPPPJ374-13|Canada|BOLD_AAH3983
Ctenosciara hyalipennis|HPPPJ290-13|Canada|BOLD_AAH3983
Ctenosciara hyalipennis|HPPPF009-13|Canada|BOLD_AAH3983
Ctenosciara hyalipennis|HPPPE1179-13|Canada|BOLD_AAH3983
Ctenosciara hyalipennis|HPPPE1106-13|Canada|BOLD_AAH3983
Ctenosciara hyalipennis|HPPPE1075-13|Canada|BOLD_AAH3983
Ctenosciara hyalipennis|HPPPE738-13|Canada|BOLD_AAH3983
Ctenosciara hyalipennis|HPPPE513-13|Canada|BOLD_AAH3983
Ctenosciara hyalipennis|HPPPA466-13|Canada|BOLD_AAH3983
Ctenosciara hyalipennis|HPPPA402-13|Canada|BOLD_AAH3983
Ctenosciara hyalipennis|HPPPA297-13|Canada|BOLD_AAH3983
Ctenosciara hyalipennis|HPPPA386-13|Canada|BOLD_AAH3983
Ctenosciara hyalipennis|HPPPF253-13|Canada|BOLD_AAH3983
Ctenosciara hyalipennis|SSBRA3004-14|Canada|BOLD_AAH3983
Ctenosciara hyalipennis|SSBRB2177-14|Canada|BOLD_AAH3983
Ctenosciara hyalipennis|SSBRA817-14|Canada|BOLD_AAH3983
Ctenosciara hyalipennis|SSBRA226-14|Canada|BOLD_AAH3983
Ctenosciara hyalipennis|CNPEM2296-14|Canada|BOLD_AAH3983
Ctenosciara hyalipennis|CNBRJ152-14|Canada|BOLD_AAH3983
Ctenosciara hyalipennis|CNPEL1813-14|Canada|BOLD_AAH3983
Ctenosciara hyalipennis|CNPEL1799-14|Canada|BOLD_AAH3983
Ctenosciara hyalipennis|CNPEL1073-14|Canada|BOLD_AAH3983
Ctenosciara hyalipennis|CNPEL337-14|Canada|BOLD_AAH3983
Ctenosciara hyalipennis|GMGRB1676-13|Germany|BOLD_AAH3983
Ctenosciara hyalipennis|CNBRN236-14|Canada|BOLD_AAH3983
[illegible]

Ctenosciara hyalipennis|CNPEL337-14|Canada|BOLD_AAH3983
Ctenosciara hyalipennis|GMGRB1676-13|Germany|BOLD_AAH3983
Ctenosciara hyalipennis|CNBRN236-14|Canada|BOLD_AAH3983
Ctenosciara hyalipennis|CNBRL567-14|Canada|BOLD_AAH3983
Ctenosciara hyalipennis|HPPPF084-13|Canada|BOLD_AAH3983
Ctenosciara hyalipennis|CNPEL1327-14|Canada|BOLD_AAH3983
Ctenosciara hyalipennis|GMGRG1023-13|Germany|BOLD_AAH3983
Ctenosciara hyalipennis|CNBRQ269-14|Canada|BOLD_AAH3983
Ctenosciara hyalipennis|GMGRA596-13|Germany|BOLD_AAH3983
Ctenosciara hyalipennis|CNBRA160-14|Canada|BOLD_AAH3983
Ctenosciara hyalipennis|GMGRF4641-13|Germany|BOLD_AAH3983
Ctenosciara hyalipennis|CNBRQ221-14|Canada|BOLD_AAH3983
Ctenosciara hyalipennis|CNBRP582-14|Canada|BOLD_AAH3983
Ctenosciara hyalipennis|CNBRK440-14|Canada|BOLD_AAH3983
Ctenosciara hyalipennis|CNBRK438-14|Canada|BOLD_AAH3983
Ctenosciara hyalipennis|CNBRK388-14|Canada|BOLD_AAH3983
Ctenosciara hyalipennis|CNBRK368-14|Canada|BOLD_AAH3983
Ctenosciara hyalipennis|CNBRK294-14|Canada|BOLD_AAH3983
Ctenosciara hyalipennis|CNBRF824-14|Canada|BOLD_AAH3983
Ctenosciara hyalipennis|CNBRF819-14|Canada|BOLD_AAH3983
Ctenosciara hyalipennis|CNBRG037-14|Canada|BOLD_AAH3983
Ctenosciara hyalipennis|CNPED432-14|Canada|BOLD_AAH3983
Ctenosciara hyalipennis|CNPEB752-14|Canada|BOLD_AAH3983
Ctenosciara hyalipennis|CNPEB613-14|Canada|BOLD_AAH3983
Ctenosciara hyalipennis|CNPEL985-14|Canada|BOLD_AAH3983
Ctenosciara hyalipennis|CNPEB1730-14|Canada|BOLD_AAH3983
Ctenosciara hyalipennis|CNPEB1172-14|Canada|BOLD_AAH3983
Ctenosciara hyalipennis|CNBRN187-14|Canada|BOLD_AAH3983
Ctenosciara hyalipennis|CNPEL1740-14|Canada|BOLD_AAH3983
Ctenosciara hyalipennis|CNBRN225-14|Canada|BOLD_AAH3983
Ctenosciara hyalipennis|CNBRN219-14|Canada|BOLD_AAH3983
Ctenosciara hyalipennis|CNBRN216-14|Canada|BOLD_AAH3983
Ctenosciara hyalipennis|CNBRN215-14|Canada|BOLD_AAH3983
Ctenosciara hyalipennis|CNBRN168-14|Canada|BOLD_AAH3983
Ctenosciara hyalipennis|CNBRN149-14|Canada|BOLD_AAH3983
Ctenosciara hyalipennis|CNBRN138-14|Canada|BOLD_AAH3983
Ctenosciara hyalipennis|CNPEB1486-14|Canada|BOLD_AAH3983
Ctenosciara hyalipennis|HPPPA173-13|Canada|BOLD_AAH3983
Ctenosciara hyalipennis|CNPEB111-14|Canada|BOLD_AAH3983
Ctenosciara hyalipennis|CNPEB103-14|Canada|BOLD_AAH3983
Ctenosciara hyalipennis|SSBRB273-14|Canada|BOLD_AAH3983
Ctenosciara hyalipennis|CNBRS121-14|Canada|BOLD_AAH3983
Ctenosciara hyalipennis|CNPEL1900-14|Canada|BOLD_AAH3983
Ctenosciara hyalipennis|CNPEB346-14|Canada|BOLD_AAH3983
Ctenosciara hyalipennis|CNPEA588-14|Canada|BOLD_AAH3983
Ctenosciara hyalipennis|CNBRB169-14|Canada|BOLD_AAH3983
Ctenosciara hyalipennis|HPPPE544-13|Canada|BOLD_AAH3983
Ctenosciara hyalipennis|CNPEM224-14|Canada|BOLD_AAH3983
Ctenosciara hyalipennis|SSPEA285-15|Canada|BOLD_AAH3983
Ctenosciara hyalipennis|CNPED270-14|Canada|BOLD_AAH3983
Ctenosciara hyalipennis|CNPER170-14|Canada|BOLD_AAH3983
Ctenosciara hyalipennis|HPPPF115-13|Canada|BOLD_AAH3983
Ctenosciara hyalipennis|HPPPG021-13|Canada|BOLD_AAH3983
Ctenosciara hyalipennis|HPPPS142-14|Canada|BOLD_AAH3983
Ctenosciara hyalipennis|CNBRM457-14|Canada|BOLD_AAH3983
Ctenosciara hyalipennis|HPPPA087-13|Canada|BOLD_AAH3983
Ctenosciara hyalipennis|HPPPB228-13|Canada|BOLD_AAH3983
Ctenosciara hyalipennis|CNBRB129-14|Canada|BOLD_AAH3983
Ctenosciara hyalipennis|CNPEB1303-14|Canada|BOLD_AAH3983
Ctenosciara hyalipennis|GMGRF3472-13|Germany|
Ctenosciara hyalipennis|SSBRB182-14|Canada|BOLD_AAH3983
Ctenosciara hyalipennis|HPPPF189-13|Canada|BOLD_AAH3983
Ctenosciara hyalipennis|CNBRN163-14|Canada|BOLD_AAH3983
Ctenosciara hyalipennis|HPPPA193-13|Canada|BOLD_AAH3983
Ctenosciara hyalipennis|CNPEB393-14|Canada|BOLD_AAH3983
Ctenosciara hyalipennis|HPPPA247-13|Canada|BOLD_AAH3983
Ctenosciara hyalipennis|CNBRP990-14|Canada|BOLD_AAH3983
Ctenosciara hyalipennis|HPPPF228-13|Canada|BOLD_AAH3983
Ctenosciara hyalipennis|HPPPF237-13|Canada|BOLD_AAH3983
Ctenosciara hyalipennis|HPPPF179-13|Canada|BOLD_AAH3983
Ctenosciara hyalipennis|HPPPF217-13|Canada|BOLD_AAH3983
Ctenosciara hyalipennis|HPPPE630-13|Canada|BOLD_AAH3983
Ctenosciara hyalipennis|HPPPF131-13|Canada|BOLD_AAH3983
Ctenosciara hyalipennis|HPPPA467-13|Canada|BOLD_AAH3983
Ctenosciara hyalipennis|HPPPB204-13|Canada|BOLD_AAH3983
Ctenosciara hyalipennis|SSBRB2847-14|Canada|BOLD_AAH3983
Ctenosciara hyalipennis|SSBRB3369-14|Canada|BOLD_AAH3983
Ctenosciara hyalipennis|CNPEL124-14|Canada|BOLD_AAH3983
Ctenosciara hyalipennis|SSBRB2782-14|Canada|BOLD_AAH3983
Ctenosciara hyalipennis|SSPEA315-15|Canada|BOLD_AAH3983
Ctenosciara hyalipennis|SSPEA366-15|Canada|BOLD_AAH3983
Ctenosciara hyalipennis|GMGMM1542-14|Germany|BOLD_AAH3983
Ctenosciara hyalipennis|SSPEA232-15|Canada|BOLD_AAH3983
Ctenosciara hyalipennis|SSBRB3343-14|Canada|BOLD_AAH3983
Ctenosciara hyalipennis|SSBRA2965-14|Canada|BOLD_AAH3983
Ctenosciara hyalipennis|SSBRB3186-14|Canada|BOLD_AAH3983
Ctenosciara hyalipennis|SSBRB3194-14|Canada|BOLD_AAH3983
Ctenosciara hyalipennis|SSBRB1662-14|Canada|BOLD_AAH3983
Ctenosciara hyalipennis|SSBRB2601-14|Canada|BOLD_AAH3983
Ctenosciara hyalipennis|SSBRA963-14|Canada|BOLD_AAH3983
Ctenosciara hyalipennis|SSBRB1503-14|Canada|BOLD_AAH3983
Ctenosciara hyalipennis|SSBRB616-14|Canada|BOLD_AAH3983
Ctenosciara hyalipennis|SSBRA901-14|Canada|BOLD_AAH3983
Ctenosciara hyalipennis|SSBRB269-14|Canada|BOLD_AAH3983
Ctenosciara hyalipennis|SSBRB464-14|Canada|BOLD_AAH3983
Ctenosciara hyalipennis|SSBRB096-14|Canada|BOLD_AAH3983
Ctenosciara hyalipennis|SSBRB230-14|Canada|BOLD_AAH3983
Ctenosciara hyalipennis|SSBRB065-14|Canada|BOLD_AAH3983
Ctenosciara hyalipennis|SSBRB078-14|Canada|BOLD_AAH3983
Ctenosciara hyalipennis|CNPEM3444-14|Canada|BOLD_AAH3983
Ctenosciara hyalipennis|CNBRR162-14|Canada|BOLD_AAH3983
Ctenosciara hyalipennis|CNPEM1939-14|Canada|BOLD_AAH3983

Ctenosciara hyalipennis|CNPEM3444-14|Canada|BOLD_AAH3983
Ctenosciara hyalipennis|CNBRR162-14|Canada|BOLD_AAH3983
Ctenosciara hyalipennis|CNPEM1939-14|Canada|BOLD_AAH3983
Ctenosciara hyalipennis|CNPEM2648-14|Canada|BOLD_AAH3983
Ctenosciara hyalipennis|CNPEM498-14|Canada|BOLD_AAH3983
Ctenosciara hyalipennis|CNPEM1909-14|Canada|BOLD_AAH3983
Ctenosciara hyalipennis|CNBRJ090-14|Canada|BOLD_AAH3983
Ctenosciara hyalipennis|CNPEM497-14|Canada|BOLD_AAH3983
Ctenosciara hyalipennis|CNPEL1851-14|Canada|BOLD_AAH3983
Ctenosciara hyalipennis|CNPEL1912-14|Canada|BOLD_AAH3983
Ctenosciara hyalipennis|CNPEL917-14|Canada|BOLD_AAH3983
Ctenosciara hyalipennis|CNPEL1280-14|Canada|BOLD_AAH3983
Ctenosciara hyalipennis|CNPEL659-14|Canada|BOLD_AAH3983
Ctenosciara hyalipennis|CNPEL849-14|Canada|BOLD_AAH3983
Ctenosciara hyalipennis|CNBRK252-14|Canada|BOLD_AAH3983
Ctenosciara hyalipennis|CNPEL341-14|Canada|BOLD_AAH3983
Ctenosciara hyalipennis|CNBRK185-14|Canada|BOLD_AAH3983
Ctenosciara hyalipennis|CNBRK192-14|Canada|BOLD_AAH3983
Ctenosciara hyalipennis|CNBRK136-14|Canada|BOLD_AAH3983
Ctenosciara hyalipennis|CNBRK165-14|Canada|BOLD_AAH3983
Ctenosciara hyalipennis|CNBRF199-14|Canada|BOLD_AAH3983
Ctenosciara hyalipennis|CNBRK099-14|Canada|BOLD_AAH3983
Ctenosciara hyalipennis|HPPPR066-13|Canada|BOLD_AAH3983
Ctenosciara hyalipennis|HPPPR068-13|Canada|BOLD_AAH3983
Ctenosciara hyalipennis|HPPPA372-13|Canada|BOLD_AAH3983
Ctenosciara hyalipennis|HPPPR050-13|Canada|BOLD_AAH3983
Ctenosciara hyalipennis|HPPPA356-13|Canada|BOLD_AAH3983
Ctenosciara hyalipennis|HPPPA366-13|Canada|BOLD_AAH3983
Ctenosciara hyalipennis|HPPPA305-13|Canada|BOLD_AAH3983
Ctenosciara hyalipennis|HPPPA354-13|Canada|BOLD_AAH3983
Ctenosciara hyalipennis|HPPPA269-13|Canada|BOLD_AAH3983
Ctenosciara hyalipennis|HPPPA291-13|Canada|BOLD_AAH3983
Ctenosciara hyalipennis|HPPPJ275-13|Canada|BOLD_AAH3983
Ctenosciara hyalipennis|HPPPJ363-13|Canada|BOLD_AAH3983
Ctenosciara hyalipennis|HPPPA382-13|Canada|BOLD_AAH3983
Ctenosciara hyalipennis|CNBRQ504-14|Canada|BOLD_AAH3983
Ctenosciara hyalipennis|SSBRB243-14|Canada|BOLD_AAH3983
Ctenosciara hyalipennis|SSBRB251-14|Canada|BOLD_AAH3983
Ctenosciara hyalipennis|HPPPA398-13|Canada|BOLD_AAH3983
Ctenosciara hyalipennis|CNBRP489-14|Canada|BOLD_AAH3983
Ctenosciara hyalipennis|CNBRQ278-14|Canada|BOLD_AAH3983
Ctenosciara hyalipennis|CNBRQ369-14|Canada|BOLD_AAH3983
Ctenosciara hyalipennis|CNPER169-14|Canada|BOLD_AAH3983
Ctenosciara hyalipennis|CNBRQ216-14|Canada|BOLD_AAH3983
Ctenosciara hyalipennis|CNPEQ149-14|Canada|BOLD_AAH3983
Ctenosciara hyalipennis|CNPEQ346-14|Canada|BOLD_AAH3983
Ctenosciara hyalipennis|CNPEB480-14|Canada|BOLD_AAH3983
Ctenosciara hyalipennis|CNPEM346-14|Canada|BOLD_AAH3983
Ctenosciara hyalipennis|HPPPK188-13|Canada|BOLD_AAH3983
Ctenosciara hyalipennis|HPPPK216-13|Canada|BOLD_AAH3983
Ctenosciara hyalipennis|HPPPJ366-13|Canada|BOLD_AAH3983
Ctenosciara hyalipennis|HPPPK186-13|Canada|BOLD_AAH3983
Ctenosciara hyalipennis|HPPPJ239-13|Canada|BOLD_AAH3983
Ctenosciara hyalipennis|HPPPJ252-13|Canada|BOLD_AAH3983
Ctenosciara hyalipennis|HPPPJ112-13|Canada|BOLD_AAH3983
Ctenosciara hyalipennis|HPPPJ190-13|Canada|BOLD_AAH3983
Ctenosciara hyalipennis|GMGRF1645-13|Germany|BOLD_AAH3983
Ctenosciara hyalipennis|HPPPJ007-13|Canada|BOLD_AAH3983
Ctenosciara hyalipennis|HPPPA462-13|Canada|BOLD_AAH3983
Ctenosciara hyalipennis|HPPPE018-13|Canada|BOLD_AAH3983
Ctenosciara hyalipennis|CNBRJ079-14|Canada|BOLD_AAH3983
Ctenosciara hyalipennis|CNBRJ097-14|Canada|BOLD_AAH3983
Ctenosciara hyalipennis|HPPPJ394-13|Canada|BOLD_AAH3983
Ctenosciara hyalipennis|HPPPS044-13|Canada|BOLD_AAH3983
Ctenosciara hyalipennis|GMGRF2214-13|Germany|BOLD_AAH3983
Ctenosciara hyalipennis|HPPPJ236-13|Canada|BOLD_AAH3983
Ctenosciara hyalipennis|HPPPE695-13|Canada|BOLD_AAH3983
Ctenosciara hyalipennis|HPPPE1209-13|Canada|BOLD_AAH3983
Ctenosciara hyalipennis|HPPPA461-13|Canada|BOLD_AAH3983
Ctenosciara hyalipennis|HPPPA464-13|Canada|BOLD_AAH3983
Ctenosciara hyalipennis|HPPPK395-13|Canada|BOLD_AAH3983
Ctenosciara hyalipennis|CNBRB119-14|Canada|BOLD_AAH3983
Ctenosciara hyalipennis|SSBRB2589-14|Canada|BOLD_AAH3983
Ctenosciara hyalipennis|SSBRB2600-14|Canada|BOLD_AAH3983
Ctenosciara hyalipennis|SSBRB066-14|Canada|BOLD_AAH3983
Ctenosciara hyalipennis|SSBRB076-14|Canada|BOLD_AAH3983
Ctenosciara hyalipennis|CNBRJ125-14|Canada|BOLD_AAH3983
Ctenosciara hyalipennis|SSBRB062-14|Canada|BOLD_AAH3983
Ctenosciara hyalipennis|CNPEL1863-14|Canada|BOLD_AAH3983
Ctenosciara hyalipennis|CNBRJ100-14|Canada|BOLD_AAH3983
Ctenosciara hyalipennis|CNPEL1752-14|Canada|BOLD_AAH3983
Ctenosciara hyalipennis|CNPEL1807-14|Canada|BOLD_AAH3983
Ctenosciara hyalipennis|CNPEL1391-14|Canada|BOLD_AAH3983
Ctenosciara hyalipennis|CNPEL1738-14|Canada|BOLD_AAH3983
Ctenosciara hyalipennis|CNBRN183-14|Canada|
Ctenosciara hyalipennis|CNBRN204-14|Canada|
Ctenosciara hyalipennis|CNPEB553-14|Canada|BOLD_AAH3983
Ctenosciara hyalipennis|CNBRN116-14|Canada|BOLD_AAH3983
Ctenosciara hyalipennis|CNBRB114-14|Canada|BOLD_AAH3983
Ctenosciara hyalipennis|CNBRQ408-14|Canada|BOLD_AAH3983
Ctenosciara hyalipennis|CNBRB156-14|Canada|BOLD_AAH3983
Ctenosciara hyalipennis|HPPPS134-14|Canada|BOLD_AAH3983
Ctenosciara hyalipennis|HPPPF162-13|Canada|BOLD_AAH3983
Ctenosciara hyalipennis|HPPPF208-13|Canada|BOLD_AAH3983
Ctenosciara hyalipennis|CNPEB775-14|Canada|BOLD_AAH3983
Ctenosciara hyalipennis|HPPPS150-14|Canada|BOLD_AAH3983
Ctenosciara hyalipennis|CNPEM1204-14|Canada|BOLD_AAH3983
Ctenosciara hyalipennis|CNPEM1937-14|Canada|BOLD_AAH3983
Ctenosciara hyalipennis|CNBRJ110-14|Canada|BOLD_AAH3983
Ctenosciara hyalipennis|CNPEM1103-14|Canada|BOLD_AAH3983
Ctenosciara hyalipennis|CNBRJ069-14|Canada|BOLD_AAH3983
Ctenosciara hyalipennis|CNBRJ075-14|Canada|BOLD_AAH3983

Ctenosciara hyalipennis|CNPEM1103-14|Canada|BOLD_AAH3983
Ctenosciara hyalipennis|CNBRJ069-14|Canada|BOLD_AAH3983
Ctenosciara hyalipennis|CNBRJ075-14|Canada|BOLD_AAH3983
Ctenosciara hyalipennis|CNBRJ042-14|Canada|BOLD_AAH3983
Ctenosciara hyalipennis|CNBRJ047-14|Canada|BOLD_AAH3983
Ctenosciara hyalipennis|CNPEL1858-14|Canada|BOLD_AAH3983
Ctenosciara hyalipennis|CNPEL1879-14|Canada|BOLD_AAH3983
Ctenosciara hyalipennis|CNPEL1558-14|Canada|BOLD_AAH3983
Ctenosciara hyalipennis|CNPEL1618-14|Canada|BOLD_AAH3983
Ctenosciara hyalipennis|CNPEL1377-14|Canada|BOLD_AAH3983
Ctenosciara hyalipennis|CNPEL1549-14|Canada|BOLD_AAH3983
Ctenosciara hyalipennis|CNPEL1316-14|Canada|BOLD_AAH3983
Ctenosciara hyalipennis|CNPEL1334-14|Canada|BOLD_AAH3983
Ctenosciara hyalipennis|CNPEL1041-14|Canada|BOLD_AAH3983
Ctenosciara hyalipennis|CNPEL1160-14|Canada|BOLD_AAH3983
Ctenosciara hyalipennis|CNPEL863-14|Canada|BOLD_AAH3983
Ctenosciara hyalipennis|CNPEL878-14|Canada|BOLD_AAH3983
Ctenosciara hyalipennis|CNPEL810-14|Canada|BOLD_AAH3983
Ctenosciara hyalipennis|CNPEL855-14|Canada|BOLD_AAH3983
Ctenosciara hyalipennis|CNPEL672-14|Canada|BOLD_AAH3983
Ctenosciara hyalipennis|CNPEL687-14|Canada|BOLD_AAH3983
Ctenosciara hyalipennis|CNPEL619-14|Canada|BOLD_AAH3983
Ctenosciara hyalipennis|CNPEL652-14|Canada|BOLD_AAH3983
Ctenosciara hyalipennis|CNPEL607-14|Canada|BOLD_AAH3983
Ctenosciara hyalipennis|CNPEL611-14|Canada|BOLD_AAH3983
Ctenosciara hyalipennis|CNPEL490-14|Canada|BOLD_AAH3983
Ctenosciara hyalipennis|CNPEL596-14|Canada|BOLD_AAH3983
Ctenosciara hyalipennis|CNPEL462-14|Canada|BOLD_AAH3983
Ctenosciara hyalipennis|CNPEL466-14|Canada|BOLD_AAH3983
Ctenosciara hyalipennis|CNPEL412-14|Canada|BOLD_AAH3983
Ctenosciara hyalipennis|CNPEL423-14|Canada|BOLD_AAH3983
Ctenosciara hyalipennis|CNPEL320-14|Canada|BOLD_AAH3983
Ctenosciara hyalipennis|CNPEL375-14|Canada|BOLD_AAH3983
Ctenosciara hyalipennis|CNPEL315-14|Canada|BOLD_AAH3983
Ctenosciara hyalipennis|CNPEL319-14|Canada|BOLD_AAH3983
Ctenosciara hyalipennis|CNPEL286-14|Canada|BOLD_AAH3983
Ctenosciara hyalipennis|CNPEL311-14|Canada|BOLD_AAH3983
Ctenosciara hyalipennis|CNPEL245-14|Canada|BOLD_AAH3983
Ctenosciara hyalipennis|CNPEL250-14|Canada|BOLD_AAH3983
Ctenosciara hyalipennis|CNPEL224-14|Canada|BOLD_AAH3983
Ctenosciara hyalipennis|CNPEL240-14|Canada|BOLD_AAH3983
Ctenosciara hyalipennis|CNBRK268-14|Canada|BOLD_AAH3983
Ctenosciara hyalipennis|CNBRK274-14|Canada|BOLD_AAH3983
Ctenosciara hyalipennis|CNBRK253-14|Canada|BOLD_AAH3983
Ctenosciara hyalipennis|CNBRK267-14|Canada|BOLD_AAH3983
Ctenosciara hyalipennis|CNBRK244-14|Canada|BOLD_AAH3983
Ctenosciara hyalipennis|CNBRK249-14|Canada|BOLD_AAH3983
Ctenosciara hyalipennis|CNBRK235-14|Canada|BOLD_AAH3983
Ctenosciara hyalipennis|CNBRK237-14|Canada|BOLD_AAH3983
Ctenosciara hyalipennis|CNBRK220-14|Canada|BOLD_AAH3983
Ctenosciara hyalipennis|CNBRK224-14|Canada|BOLD_AAH3983
Ctenosciara hyalipennis|CNBRK206-14|Canada|BOLD_AAH3983
Ctenosciara hyalipennis|CNBRK209-14|Canada|BOLD_AAH3983
Ctenosciara hyalipennis|CNBRK200-14|Canada|BOLD_AAH3983
Ctenosciara hyalipennis|CNBRK201-14|Canada|BOLD_AAH3983
Ctenosciara hyalipennis|CNBRK197-14|Canada|BOLD_AAH3983
Ctenosciara hyalipennis|CNBRK198-14|Canada|BOLD_AAH3983
Ctenosciara hyalipennis|CNBRK183-14|Canada|BOLD_AAH3983
Ctenosciara hyalipennis|CNBRK189-14|Canada|BOLD_AAH3983
Ctenosciara hyalipennis|CNBRK168-14|Canada|BOLD_AAH3983
Ctenosciara hyalipennis|CNBRK178-14|Canada|BOLD_AAH3983
Ctenosciara hyalipennis|CNBRK147-14|Canada|BOLD_AAH3983
Ctenosciara hyalipennis|CNBRK149-14|Canada|BOLD_AAH3983
Ctenosciara hyalipennis|CNBRK137-14|Canada|BOLD_AAH3983
Ctenosciara hyalipennis|CNBRK138-14|Canada|BOLD_AAH3983
Ctenosciara hyalipennis|CNBRK118-14|Canada|BOLD_AAH3983
Ctenosciara hyalipennis|CNBRK135-14|Canada|BOLD_AAH3983
Ctenosciara hyalipennis|CNBRK108-14|Canada|BOLD_AAH3983
Ctenosciara hyalipennis|CNBRK117-14|Canada|BOLD_AAH3983
Ctenosciara hyalipennis|CNBRK102-14|Canada|BOLD_AAH3983
Ctenosciara hyalipennis|CNBRK106-14|Canada|BOLD_AAH3983
Ctenosciara hyalipennis|CNBRK092-14|Canada|BOLD_AAH3983
Ctenosciara hyalipennis|CNBRK101-14|Canada|BOLD_AAH3983
Ctenosciara hyalipennis|CNBRI089-14|Canada|BOLD_AAH3983
Ctenosciara hyalipennis|CNBRI107-14|Canada|BOLD_AAH3983
Ctenosciara hyalipennis|CNBRH201-14|Canada|BOLD_AAH3983
Ctenosciara hyalipennis|CNPEI082-14|Canada|BOLD_AAH3983
Ctenosciara hyalipennis|CNBRH112-14|Canada|BOLD_AAH3983
Ctenosciara hyalipennis|CNBRH135-14|Canada|BOLD_AAH3983
Ctenosciara hyalipennis|CNPEG394-14|Canada|BOLD_AAH3983
Ctenosciara hyalipennis|CNBRH108-14|Canada|BOLD_AAH3983
Ctenosciara hyalipennis|CNBRE122-14|Canada|BOLD_AAH3983
Ctenosciara hyalipennis|CNBRE876-14|Canada|BOLD_AAH3983
Ctenosciara hyalipennis|HPPPS020-13|Canada|BOLD_AAH3983
Ctenosciara hyalipennis|HPPPS024-13|Canada|BOLD_AAH3983
Ctenosciara hyalipennis|HPPPS016-13|Canada|BOLD_AAH3983
Ctenosciara hyalipennis|HPPPS018-13|Canada|BOLD_AAH3983
Ctenosciara hyalipennis|HPPPS009-13|Canada|BOLD_AAH3983
Ctenosciara hyalipennis|HPPPS012-13|Canada|BOLD_AAH3983
Ctenosciara hyalipennis|HPPPO138-13|Canada|BOLD_AAH3983
Ctenosciara hyalipennis|HPPPO274-13|Canada|BOLD_AAH3983
Ctenosciara hyalipennis|CNPEB572-14|Canada|BOLD_AAH3983
Ctenosciara hyalipennis|CNPEM3168-14|Canada|BOLD_AAH3983
Ctenosciara hyalipennis|HPPPS126-13|Canada|BOLD_AAH3983
Ctenosciara hyalipennis|CNBRA046-14|Canada|BOLD_AAH3983
Ctenosciara hyalipennis|CNBRB159-14|Canada|BOLD_AAH3983
Ctenosciara hyalipennis|SSBRA073-14|Canada|BOLD_AAH3983
Ctenosciara hyalipennis|CNPEM1974-14|Canada|BOLD_AAH3983
Ctenosciara hyalipennis|SSBRB3227-14|Canada|BOLD_AAH3983
Ctenosciara hyalipennis|CNPEL1101-14|Canada|BOLD_AAH3983
Ctenosciara hyalipennis|CNPEL1409-14|Canada|BOLD_AAH3983
Ctenosciara hyalipennis|CNPEL842-14|Canada|BOLD_AAH3983

Ctenosciara hyalipennis|SSBRB3227-14|Canada|BOLD_AAH3983
Ctenosciara hyalipennis|CNPEL1101-14|Canada|BOLD_AAH3983
Ctenosciara hyalipennis|CNPEL1409-14|Canada|BOLD_AAH3983
Ctenosciara hyalipennis|CNPEL842-14|Canada|BOLD_AAH3983
Ctenosciara hyalipennis|CNPEL947-14|Canada|BOLD_AAH3983
Ctenosciara hyalipennis|HPPPM269-13|Canada|BOLD_AAH3983
Ctenosciara hyalipennis|CNBRB184-14|Canada|BOLD_AAH3983
Ctenosciara hyalipennis|HPPPA440-13|Canada|BOLD_AAH3983
Ctenosciara hyalipennis|CNBRB183-14|Canada|BOLD_AAH3983
Ctenosciara hyalipennis|CNBRR262-14|Canada|BOLD_AAH3983
Ctenosciara hyalipennis|SSBRA037-14|Canada|BOLD_AAH3983
Ctenosciara hyalipennis|CNBRO469-14|Canada|BOLD_AAH3983
Ctenosciara hyalipennis|CNBRR190-14|Canada|BOLD_AAH3983
Ctenosciara hyalipennis|CNPEM2760-14|Canada|BOLD_AAH3983
Ctenosciara hyalipennis|CNPEM2905-14|Canada|BOLD_AAH3983
Ctenosciara hyalipennis|CNPEM2354-14|Canada|BOLD_AAH3983
Ctenosciara hyalipennis|CNPEM2611-14|Canada|BOLD_AAH3983
Ctenosciara hyalipennis|CNPEL1802-14|Canada|BOLD_AAH3983
Ctenosciara hyalipennis|CNPEL1848-14|Canada|BOLD_AAH3983
Ctenosciara hyalipennis|CNPEL1778-14|Canada|BOLD_AAH3983
Ctenosciara hyalipennis|CNPEL1781-14|Canada|BOLD_AAH3983
Ctenosciara hyalipennis|CNPEL1700-14|Canada|BOLD_AAH3983
Ctenosciara hyalipennis|CNPEL1743-14|Canada|BOLD_AAH3983
Ctenosciara hyalipennis|CNPEL1637-14|Canada|BOLD_AAH3983
Ctenosciara hyalipennis|CNPEL1678-14|Canada|BOLD_AAH3983
Ctenosciara hyalipennis|CNPEL1296-14|Canada|BOLD_AAH3983
Ctenosciara hyalipennis|CNPEL1306-14|Canada|BOLD_AAH3983
Ctenosciara hyalipennis|CNPEL1265-14|Canada|BOLD_AAH3983
Ctenosciara hyalipennis|CNPEL1288-14|Canada|BOLD_AAH3983
Ctenosciara hyalipennis|CNPEL1251-14|Canada|BOLD_AAH3983
Ctenosciara hyalipennis|CNPEL1261-14|Canada|BOLD_AAH3983
Ctenosciara hyalipennis|CNPEL1209-14|Canada|BOLD_AAH3983
Ctenosciara hyalipennis|CNPEL1215-14|Canada|BOLD_AAH3983
Ctenosciara hyalipennis|CNPEL1192-14|Canada|BOLD_AAH3983
Ctenosciara hyalipennis|CNPEL1193-14|Canada|BOLD_AAH3983
Ctenosciara hyalipennis|CNPEL1037-14|Canada|BOLD_AAH3983
Ctenosciara hyalipennis|CNPEL1039-14|Canada|BOLD_AAH3983
Ctenosciara hyalipennis|CNPEL997-14|Canada|BOLD_AAH3983
Ctenosciara hyalipennis|CNPEL1011-14|Canada|BOLD_AAH3983
Ctenosciara hyalipennis|CNPEL881-14|Canada|BOLD_AAH3983
Ctenosciara hyalipennis|CNPEL949-14|Canada|BOLD_AAH3983
Ctenosciara hyalipennis|CNBRK231-14|Canada|BOLD_AAH3983
Ctenosciara hyalipennis|CNBRK232-14|Canada|BOLD_AAH3983
Ctenosciara hyalipennis|CNBRK179-14|Canada|BOLD_AAH3983
Ctenosciara hyalipennis|CNBRK180-14|Canada|BOLD_AAH3983
Ctenosciara hyalipennis|CNBRK161-14|Canada|BOLD_AAH3983
Ctenosciara hyalipennis|CNBRK163-14|Canada|BOLD_AAH3983
Ctenosciara hyalipennis|CNBRK151-14|Canada|BOLD_AAH3983
Ctenosciara hyalipennis|CNBRK159-14|Canada|BOLD_AAH3983
Ctenosciara hyalipennis|HPPPQ089-13|Canada|BOLD_AAH3983
Ctenosciara hyalipennis|HPPPR058-13|Canada|BOLD_AAH3983
Ctenosciara hyalipennis|GMGRG1324-13|Germany|BOLD_AAH3983
Ctenosciara hyalipennis|GMGRG2041-13|Germany|BOLD_AAH3983
Ctenosciara hyalipennis|HPPPA374-13|Canada|BOLD_AAH3983
Ctenosciara hyalipennis|HPPPA377-13|Canada|BOLD_AAH3983
Ctenosciara hyalipennis|HPPPA357-13|Canada|BOLD_AAH3983
Ctenosciara hyalipennis|HPPPA370-13|Canada|BOLD_AAH3983
Ctenosciara hyalipennis|HPPPA336-13|Canada|BOLD_AAH3983
Ctenosciara hyalipennis|HPPPA343-13|Canada|BOLD_AAH3983
Ctenosciara hyalipennis|HPPPA310-13|Canada|BOLD_AAH3983
Ctenosciara hyalipennis|HPPPA314-13|Canada|BOLD_AAH3983
Ctenosciara hyalipennis|HPPPA240-13|Canada|BOLD_AAH3983
Ctenosciara hyalipennis|HPPPA251-13|Canada|BOLD_AAH3983
Ctenosciara hyalipennis|HPPPA210-13|Canada|BOLD_AAH3983
Ctenosciara hyalipennis|HPPPA211-13|Canada|BOLD_AAH3983
Ctenosciara hyalipennis|HPPPA203-13|Canada|BOLD_AAH3983
Ctenosciara hyalipennis|HPPPA205-13|Canada|BOLD_AAH3983
Ctenosciara hyalipennis|CNBRN235-14|Canada|BOLD_AAH3983
Ctenosciara hyalipennis|CNBRQ577-14|Canada|BOLD_AAH3983
Ctenosciara hyalipennis|CNBRA053-14|Canada|BOLD_AAH3983
Ctenosciara hyalipennis|CNBRN191-14|Canada|BOLD_AAH3983
Ctenosciara hyalipennis|HPPPK442-13|Canada|BOLD_AAH3983
Ctenosciara hyalipennis|CNBRA151-14|Canada|BOLD_AAH3983
Ctenosciara hyalipennis|HPPPK382-13|Canada|BOLD_AAH3983
Ctenosciara hyalipennis|HPPPK387-13|Canada|BOLD_AAH3983
Ctenosciara hyalipennis|HPPPF506-13|Canada|BOLD_AAH3983
Ctenosciara hyalipennis|HPPPF781-13|Canada|BOLD_AAH3983
Ctenosciara hyalipennis|HPPPF451-13|Canada|BOLD_AAH3983
Ctenosciara hyalipennis|HPPPF502-13|Canada|BOLD_AAH3983
Ctenosciara hyalipennis|HPPPF391-13|Canada|BOLD_AAH3983
Ctenosciara hyalipennis|HPPPF398-13|Canada|BOLD_AAH3983
Ctenosciara hyalipennis|GMGRF255-13|Germany|BOLD_AAH3983
Ctenosciara hyalipennis|CNPEB1103-14|Canada|BOLD_AAH3983
Ctenosciara hyalipennis|HPPPF862-13|Canada|BOLD_AAH3983
Ctenosciara hyalipennis|CNPEB959-14|Canada|BOLD_AAH3983
Ctenosciara hyalipennis|SSPEA248-15|Canada|BOLD_AAH3983
Ctenosciara hyalipennis|SSPEA349-15|Canada|BOLD_AAH3983
Ctenosciara hyalipennis|SSBRB3346-14|Canada|BOLD_AAH3983
Ctenosciara hyalipennis|SSBRC1357-14|Canada|BOLD_AAH3983
Ctenosciara hyalipennis|SSBRB3257-14|Canada|BOLD_AAH3983
Ctenosciara hyalipennis|SSBRB3275-14|Canada|BOLD_AAH3983
Ctenosciara hyalipennis|SSBRB3199-14|Canada|BOLD_AAH3983
Ctenosciara hyalipennis|SSBRB3215-14|Canada|BOLD_AAH3983
Ctenosciara hyalipennis|SSBRB2828-14|Canada|BOLD_AAH3983
Ctenosciara hyalipennis|SSBRB3198-14|Canada|BOLD_AAH3983
Ctenosciara hyalipennis|SSBRB2810-14|Canada|BOLD_AAH3983
Ctenosciara hyalipennis|SSBRB2823-14|Canada|BOLD_AAH3983
Ctenosciara hyalipennis|SSBRB2752-14|Canada|BOLD_AAH3983
Ctenosciara hyalipennis|SSBRB2758-14|Canada|BOLD_AAH3983
Ctenosciara hyalipennis|SSBRB2698-14|Canada|BOLD_AAH3983
Ctenosciara hyalipennis|SSBRB2751-14|Canada|BOLD_AAH3983
Ctenosciara hyalipennis|SSBRB509-14|Canada|BOLD_AAH3983
[illegible]

Ctenosciara hyalipennis|SSBRB2698-14|Canada|BOLD_AAH3983
Ctenosciara hyalipennis|SSBRB2751-14|Canada|BOLD_AAH3983
Ctenosciara hyalipennis|SSBRB509-14|Canada|BOLD_AAH3983
Ctenosciara hyalipennis|SSBRB573-14|Canada|BOLD_AAH3983
Ctenosciara hyalipennis|SSBRB093-14|Canada|BOLD_AAH3983
Ctenosciara hyalipennis|SSBRB113-14|Canada|BOLD_AAH3983
Ctenosciara hyalipennis|CNPEL1257-14|Canada|BOLD_AAH3983
Ctenosciara hyalipennis|CNPEL1350-14|Canada|BOLD_AAH3983
Ctenosciara hyalipennis|CNPEL1079-14|Canada|BOLD_AAH3983
Ctenosciara hyalipennis|CNPEL1082-14|Canada|BOLD_AAH3983
Ctenosciara hyalipennis|CNPEL918-14|Canada|BOLD_AAH3983
Ctenosciara hyalipennis|CNPEL924-14|Canada|BOLD_AAH3983
Ctenosciara hyalipennis|CNPEL828-14|Canada|BOLD_AAH3983
Ctenosciara hyalipennis|CNPEL862-14|Canada|BOLD_AAH3983
Ctenosciara hyalipennis|CNPEL484-14|Canada|BOLD_AAH3983
Ctenosciara hyalipennis|CNPEL493-14|Canada|BOLD_AAH3983
Ctenosciara hyalipennis|CNPEL392-14|Canada|BOLD_AAH3983
Ctenosciara hyalipennis|CNPEL470-14|Canada|BOLD_AAH3983
Ctenosciara hyalipennis|CNPEQ512-14|Canada|BOLD_AAH3983
Ctenosciara hyalipennis|CNBRP1560-14|Canada|BOLD_AAH3983
Ctenosciara hyalipennis|CNPEB406-14|Canada|BOLD_AAH3983
Ctenosciara hyalipennis|CNBRQ326-14|Canada|BOLD_AAH3983
Ctenosciara hyalipennis|HPPPJ330-13|Canada|BOLD_AAH3983
Ctenosciara hyalipennis|HPPPK391-13|Canada|BOLD_AAH3983
Ctenosciara hyalipennis|HPPPJ234-13|Canada|BOLD_AAH3983
Ctenosciara hyalipennis|HPPPJ280-13|Canada|BOLD_AAH3983
Ctenosciara hyalipennis|SSBRB1935-14|Canada|BOLD_AAH3983
Ctenosciara hyalipennis|SSBRB2582-14|Canada|BOLD_AAH3983
Ctenosciara hyalipennis|SSBRA859-14|Canada|BOLD_AAH3983
Ctenosciara hyalipennis|SSBRB1736-14|Canada|BOLD_AAH3983
Ctenosciara hyalipennis|SSBRB121-14|Canada|BOLD_AAH3983
Ctenosciara hyalipennis|SSBRB436-14|Canada|BOLD_AAH3983
Ctenosciara hyalipennis|SSBRB061-14|Canada|BOLD_AAH3983
Ctenosciara hyalipennis|SSBRB081-14|Canada|BOLD_AAH3983
Ctenosciara hyalipennis|SSBRB029-14|Canada|BOLD_AAH3983
Ctenosciara hyalipennis|SSBRB060-14|Canada|BOLD_AAH3983
Ctenosciara hyalipennis|SSBRA052-14|Canada|BOLD_AAH3983
Ctenosciara hyalipennis|SSBRB028-14|Canada|BOLD_AAH3983
Ctenosciara hyalipennis|HPPPA319-13|Canada|BOLD_AAH3983
Ctenosciara hyalipennis|HPPPA331-13|Canada|BOLD_AAH3983
Ctenosciara hyalipennis|HPPPA292-13|Canada|BOLD_AAH3983
Ctenosciara hyalipennis|HPPPA295-13|Canada|BOLD_AAH3983
Ctenosciara hyalipennis|HPPPA284-13|Canada|BOLD_AAH3983
Ctenosciara hyalipennis|HPPPA289-13|Canada|BOLD_AAH3983
Ctenosciara hyalipennis|HPPPA257-13|Canada|BOLD_AAH3983
Ctenosciara hyalipennis|HPPPA263-13|Canada|BOLD_AAH3983
Ctenosciara hyalipennis|HPPPA229-13|Canada|BOLD_AAH3983
Ctenosciara hyalipennis|HPPPA231-13|Canada|BOLD_AAH3983
Ctenosciara hyalipennis|HPPPA197-13|Canada|BOLD_AAH3983
Ctenosciara hyalipennis|HPPPA198-13|Canada|BOLD_AAH3983
Ctenosciara hyalipennis|HPPPJ459-13|Canada|BOLD_AAH3983
Ctenosciara hyalipennis|CNBRK264-14|Canada|BOLD_AAH3983
Ctenosciara hyalipennis|CNBRA041-14|Canada|BOLD_AAH3983
Ctenosciara hyalipennis|CNBRA172-14|Canada|BOLD_AAH3983
Ctenosciara hyalipennis|SSBRA966-14|Canada|BOLD_AAH3983
Ctenosciara hyalipennis|SSBRB1779-14|Canada|BOLD_AAH3983
Ctenosciara hyalipennis|CNBRP246-14|Canada|BOLD_AAH3983
Ctenosciara hyalipennis|CNBRQ531-14|Canada|BOLD_AAH3983
Ctenosciara hyalipennis|CNBRB104-14|Canada|BOLD_AAH3983
Ctenosciara hyalipennis|CNBRN144-14|Canada|BOLD_AAH3983
Ctenosciara hyalipennis|HPPPK194-13|Canada|BOLD_AAH3983
Ctenosciara hyalipennis|CNBRQ553-14|Canada|BOLD_AAH3983
Ctenosciara hyalipennis|SSBRC669-14|Canada|BOLD_AAH3983
Ctenosciara hyalipennis|SSBRC777-14|Canada|BOLD_AAH3983
Ctenosciara hyalipennis|SSBRB4212-14|Canada|BOLD_AAH3983
Ctenosciara hyalipennis|SSBRC659-14|Canada|BOLD_AAH3983
Ctenosciara hyalipennis|SSBRB4002-14|Canada|BOLD_AAH3983
Ctenosciara hyalipennis|SSBRB4025-14|Canada|BOLD_AAH3983
Ctenosciara hyalipennis|SSBRB3574-14|Canada|BOLD_AAH3983
Ctenosciara hyalipennis|SSBRB3976-14|Canada|BOLD_AAH3983
Ctenosciara hyalipennis|SSBRB3339-14|Canada|BOLD_AAH3983
Ctenosciara hyalipennis|SSBRB3359-14|Canada|BOLD_AAH3983
Ctenosciara hyalipennis|SSBRB3333-14|Canada|BOLD_AAH3983
Ctenosciara hyalipennis|SSBRB3337-14|Canada|BOLD_AAH3983
Ctenosciara hyalipennis|SSBRB3046-14|Canada|BOLD_AAH3983
Ctenosciara hyalipennis|SSBRB3064-14|Canada|BOLD_AAH3983
Ctenosciara hyalipennis|SSBRB2814-14|Canada|BOLD_AAH3983
Ctenosciara hyalipennis|SSBRB2831-14|Canada|BOLD_AAH3983
Ctenosciara hyalipennis|SSBRB2649-14|Canada|BOLD_AAH3983
Ctenosciara hyalipennis|SSBRB2761-14|Canada|BOLD_AAH3983
Ctenosciara hyalipennis|SSBRB2612-14|Canada|BOLD_AAH3983
Ctenosciara hyalipennis|SSBRB2627-14|Canada|BOLD_AAH3983
Ctenosciara hyalipennis|CNPEG035-14|Canada|BOLD_AAH3983
Ctenosciara hyalipennis|CNPEL186-14|Canada|BOLD_AAH3983
Ctenosciara hyalipennis|HPPPE582-13|Canada|BOLD_AAH3983
Ctenosciara hyalipennis|HPPPE587-13|Canada|BOLD_AAH3983
Ctenosciara hyalipennis|CNPEL329-14|Canada|BOLD_AAH3983
Ctenosciara hyalipennis|CNPEL367-14|Canada|BOLD_AAH3983
Ctenosciara hyalipennis|CNBRK255-14|Canada|BOLD_AAH3983
Ctenosciara hyalipennis|CNPEL227-14|Canada|BOLD_AAH3983
Ctenosciara hyalipennis|CNBRK225-14|Canada|BOLD_AAH3983
Ctenosciara hyalipennis|CNBRK248-14|Canada|BOLD_AAH3983
Ctenosciara hyalipennis|CNBRK213-14|Canada|BOLD_AAH3983
Ctenosciara hyalipennis|CNBRK216-14|Canada|BOLD_AAH3983
Ctenosciara hyalipennis|CNBRK162-14|Canada|BOLD_AAH3983
Ctenosciara hyalipennis|CNBRK191-14|Canada|BOLD_AAH3983
Ctenosciara hyalipennis|CNBRK131-14|Canada|BOLD_AAH3983
Ctenosciara hyalipennis|CNBRK142-14|Canada|BOLD_AAH3983
Ctenosciara hyalipennis|CNBRK097-14|Canada|BOLD_AAH3983
Ctenosciara hyalipennis|CNBRK112-14|Canada|BOLD_AAH3983
Ctenosciara hyalipennis|CNBRI104-14|Canada|BOLD_AAH3983
Ctenosciara hyalipennis|CNBRK091-14|Canada|BOLD_AAH3983

Ctenosciara hyalipennis|CNBRK112-14|Canada|BOLD_AAH3983
Ctenosciara hyalipennis|CNBRI104-14|Canada|BOLD_AAH3983
Ctenosciara hyalipennis|CNBRK091-14|Canada|BOLD_AAH3983
Ctenosciara hyalipennis|GMGRF3202-13|Germany|BOLD_AAH3983
Ctenosciara hyalipennis|HPPPL501-13|Canada|BOLD_AAH3983
Ctenosciara hyalipennis|HPPPA352-13|Canada|BOLD_AAH3983
Ctenosciara hyalipennis|HPPPA358-13|Canada|BOLD_AAH3983
Ctenosciara hyalipennis|HPPPA344-13|Canada|BOLD_AAH3983
Ctenosciara hyalipennis|HPPPA351-13|Canada|BOLD_AAH3983
Ctenosciara hyalipennis|HPPPA328-13|Canada|BOLD_AAH3983
Ctenosciara hyalipennis|HPPPA329-13|Canada|BOLD_AAH3983
Ctenosciara hyalipennis|HPPPA272-13|Canada|BOLD_AAH3983
Ctenosciara hyalipennis|HPPPA320-13|Canada|BOLD_AAH3983
Ctenosciara hyalipennis|CNBRN237-14|Canada|BOLD_AAH3983
Ctenosciara hyalipennis|CNBRQ546-14|Canada|BOLD_AAH3983
Ctenosciara hyalipennis|CNPEL086-14|Canada|BOLD_AAH3983
Ctenosciara hyalipennis|SSPEA316-15|Canada|BOLD_AAH3983
Ctenosciara hyalipennis|HPPPA136-13|Canada|BOLD_AAH3983
Ctenosciara hyalipennis|GMGRG2136-13|Germany|BOLD_AAH3983
Ctenosciara hyalipennis|SSPEA353-15|Canada|BOLD_AAH3983
Ctenosciara hyalipennis|SSPEA425-15|Canada|BOLD_AAH3983
Ctenosciara hyalipennis|SSPEA254-15|Canada|BOLD_AAH3983
Ctenosciara hyalipennis|SSPEA288-15|Canada|BOLD_AAH3983
Ctenosciara hyalipennis|SSPEA189-15|Canada|BOLD_AAH3983
Ctenosciara hyalipennis|SSPEA241-15|Canada|BOLD_AAH3983
Ctenosciara hyalipennis|GMGMG789-14|Germany|BOLD_AAH3983
Ctenosciara hyalipennis|GMGMG840-14|Germany|BOLD_AAH3983
Ctenosciara hyalipennis|SSBRC1399-14|Canada|BOLD_AAH3983
Ctenosciara hyalipennis|SSBRC3256-14|Canada|BOLD_AAH3983
Ctenosciara hyalipennis|SSBRC1375-14|Canada|BOLD_AAH3983
Ctenosciara hyalipennis|SSBRC1395-14|Canada|BOLD_AAH3983
Ctenosciara hyalipennis|SSBRC1306-14|Canada|BOLD_AAH3983
Ctenosciara hyalipennis|SSBRC1355-14|Canada|BOLD_AAH3983
Ctenosciara hyalipennis|SSBRC937-14|Canada|BOLD_AAH3983
Ctenosciara hyalipennis|SSBRC1066-14|Canada|BOLD_AAH3983
Ctenosciara hyalipennis|SSBRC824-14|Canada|BOLD_AAH3983
Ctenosciara hyalipennis|SSBRC927-14|Canada|BOLD_AAH3983
Ctenosciara hyalipennis|SSBRB3285-14|Canada|BOLD_AAH3983
Ctenosciara hyalipennis|SSBRB3321-14|Canada|BOLD_AAH3983
Ctenosciara hyalipennis|SSBRB3252-14|Canada|BOLD_AAH3983
Ctenosciara hyalipennis|SSBRB3271-14|Canada|BOLD_AAH3983
Ctenosciara hyalipennis|SSBRB3190-14|Canada|BOLD_AAH3983
Ctenosciara hyalipennis|SSBRB3192-14|Canada|BOLD_AAH3983
Ctenosciara hyalipennis|SSBRB3183-14|Canada|BOLD_AAH3983
Ctenosciara hyalipennis|SSBRB3188-14|Canada|BOLD_AAH3983
Ctenosciara hyalipennis|CNBRQ373-14|Canada|BOLD_AAH3983
Ctenosciara hyalipennis|CNBRQ381-14|Canada|BOLD_AAH3983
Ctenosciara hyalipennis|CNBRB143-14|Canada|BOLD_AAH3983
Ctenosciara hyalipennis|CNBRB201-14|Canada|BOLD_AAH3983
Ctenosciara hyalipennis|CNPEB1297-14|Canada|BOLD_AAH3983
Ctenosciara hyalipennis|CNBRK387-14|Canada|BOLD_AAH3983
Ctenosciara hyalipennis|HPPPA274-13|Canada|BOLD_AAH3983
Ctenosciara hyalipennis|CNBRN174-14|Canada|BOLD_AAH3983
Ctenosciara hyalipennis|CNPEL1078-14|Canada|BOLD_AAH3983
Ctenosciara hyalipennis|CNPEL1225-14|Canada|BOLD_AAH3983
Ctenosciara hyalipennis|CNPEL951-14|Canada|BOLD_AAH3983
Ctenosciara hyalipennis|CNPEL994-14|Canada|BOLD_AAH3983
Ctenosciara hyalipennis|CNPEL857-14|Canada|BOLD_AAH3983
Ctenosciara hyalipennis|CNPEL913-14|Canada|BOLD_AAH3983
Ctenosciara hyalipennis|CNPEL182-14|Canada|BOLD_AAH3983
Ctenosciara hyalipennis|CNBRN1224-14|Canada|BOLD_AAH3983
Ctenosciara hyalipennis|SSBRB3200-14|Canada|BOLD_AAH3983
Ctenosciara hyalipennis|SSBRB3247-14|Canada|BOLD_AAH3983
Ctenosciara hyalipennis|GMGRF2632-13|Germany|BOLD_AAH3983
Ctenosciara hyalipennis|CNPEB1724-14|Canada|BOLD_AAH3983
Ctenosciara hyalipennis|HPPPA090-13|Canada|BOLD_AAH3983
Ctenosciara hyalipennis|CNPEL200-14|Canada|BOLD_AAH3983
Ctenosciara hyalipennis|CNBRK210-14|Canada|BOLD_AAH3983
Ctenosciara hyalipennis|CNBRN157-14|Canada|BOLD_AAH3983
Ctenosciara hyalipennis|CNPEC198-14|Canada|BOLD_AAH3983
Ctenosciara hyalipennis|CNBRN181-14|Canada|BOLD_AAH3983
Ctenosciara hyalipennis|HPPPF520-13|Canada|BOLD_AAH3983
Ctenosciara hyalipennis|CNPEB1512-14|Canada|BOLD_AAH3983
Ctenosciara hyalipennis|HPPPA076-13|Canada|BOLD_AAH3983
Ctenosciara hyalipennis|HPPPA085-13|Canada|BOLD_AAH3983
Ctenosciara hyalipennis|CNBRP020-14|Canada|BOLD_AAH3983
Ctenosciara hyalipennis|CNBRQ294-14|Canada|BOLD_AAH3983
Ctenosciara hyalipennis|CNPEQ142-14|Canada|BOLD_AAH3983
Ctenosciara hyalipennis|CNPEQ354-14|Canada|BOLD_AAH3983
Ctenosciara hyalipennis|CNBRM378-14|Canada|BOLD_AAH3983
Ctenosciara hyalipennis|CNBRM484-14|Canada|BOLD_AAH3983
Ctenosciara hyalipennis|CNPEM2146-14|Canada|BOLD_AAH3983
Ctenosciara hyalipennis|CNBRN172-14|Canada|BOLD_AAH3983
Ctenosciara hyalipennis|CNBRK420-14|Canada|BOLD_AAH3983
Ctenosciara hyalipennis|CNBRL501-14|Canada|BOLD_AAH3983
Ctenosciara hyalipennis|CNPEB453-14|Canada|BOLD_AAH3983
Ctenosciara hyalipennis|CNBRK329-14|Canada|BOLD_AAH3983
Ctenosciara hyalipennis|CNPEB1727-14|Canada|BOLD_AAH3983
Ctenosciara hyalipennis|SSBRB2836-14|Canada|BOLD_AAH3983
Ctenosciara hyalipennis|CNBRB208-14|Canada|BOLD_AAH3983
Ctenosciara hyalipennis|CNPEA536-14|Canada|BOLD_AAH3983
Ctenosciara hyalipennis|HPPPE733-13|Canada|BOLD_AAH3983
Ctenosciara hyalipennis|CNBRA173-14|Canada|BOLD_AAH3983
Ctenosciara hyalipennis|HPPPS139-14|Canada|BOLD_AAH3983
Ctenosciara hyalipennis|CNBRQ230-14|Canada|BOLD_AAH3983
Ctenosciara hyalipennis|HPPPJ297-13|Canada|BOLD_AAH3983
Ctenosciara hyalipennis|CNBRB187-14|Canada|BOLD_AAH3983
Ctenosciara hyalipennis|HPPPF493-13|Canada|BOLD_AAH3983
Ctenosciara hyalipennis|HPPPF514-13|Canada|BOLD_AAH3983
Ctenosciara hyalipennis|HPPPF403-13|Canada|BOLD_AAH3983
Ctenosciara hyalipennis|HPPPF415-13|Canada|BOLD_AAH3983
Ctenosciara hyalipennis|HPPPA191-13|Canada|BOLD_AAH3983

Ctenosciara hyalipennis|HPPPF403-13|Canada|BOLD_AAH3983
Ctenosciara hyalipennis|HPPPF415-13|Canada|BOLD_AAH3983
Ctenosciara hyalipennis|HPPPA191-13|Canada|BOLD_AAH3983
Ctenosciara hyalipennis|CNBRS124-14|Canada|BOLD_AAH3983
Ctenosciara hyalipennis|HPPPA333-13|Canada|BOLD_AAH3983
Ctenosciara hyalipennis|HPPPA342-13|Canada|BOLD_AAH3983
Ctenosciara hyalipennis|SSBRB3229-14|Canada|BOLD_AAH3983
Ctenosciara hyalipennis|GMGMC1530-14|Germany|BOLD_AAH3983
Ctenosciara hyalipennis|SSBRB3195-14|Canada|BOLD_AAH3983
Ctenosciara hyalipennis|SSBRB3211-14|Canada|BOLD_AAH3983
Ctenosciara hyalipennis|SSBRB588-14|Canada|BOLD_AAH3983
Ctenosciara hyalipennis|SSBRB2843-14|Canada|BOLD_AAH3983
Ctenosciara hyalipennis|CNPEL1423-14|Canada|BOLD_AAH3983
Ctenosciara hyalipennis|SSBRB218-14|Canada|BOLD_AAH3983
Ctenosciara hyalipennis|CNPEL1342-14|Canada|BOLD_AAH3983
Ctenosciara hyalipennis|CNPEL1346-14|Canada|BOLD_AAH3983
Ctenosciara hyalipennis|CNPEL1297-14|Canada|BOLD_AAH3983
Ctenosciara hyalipennis|CNPEL1332-14|Canada|BOLD_AAH3983
Ctenosciara hyalipennis|CNPEL1238-14|Canada|BOLD_AAH3983
Ctenosciara hyalipennis|CNPEL1250-14|Canada|BOLD_AAH3983
Ctenosciara hyalipennis|CNBRK260-14|Canada|BOLD_AAH3983
Ctenosciara hyalipennis|CNPEL233-14|Canada|BOLD_AAH3983
Ctenosciara hyalipennis|CNBRK243-14|Canada|BOLD_AAH3983
Ctenosciara hyalipennis|CNBRK250-14|Canada|BOLD_AAH3983
Ctenosciara hyalipennis|CNBRK207-14|Canada|BOLD_AAH3983
Ctenosciara hyalipennis|CNBRK230-14|Canada|BOLD_AAH3983
Ctenosciara hyalipennis|CNBRK203-14|Canada|BOLD_AAH3983
Ctenosciara hyalipennis|CNBRK205-14|Canada|BOLD_AAH3983
Ctenosciara hyalipennis|CNBRK174-14|Canada|BOLD_AAH3983
Ctenosciara hyalipennis|CNBRK193-14|Canada|BOLD_AAH3983
Ctenosciara hyalipennis|CNBRK172-14|Canada|BOLD_AAH3983
Ctenosciara hyalipennis|CNBRK173-14|Canada|BOLD_AAH3983
Ctenosciara hyalipennis|CNBRK130-14|Canada|BOLD_AAH3983
Ctenosciara hyalipennis|CNBRK132-14|Canada|BOLD_AAH3983
Ctenosciara hyalipennis|CNBRH179-14|Canada|BOLD_AAH3983
Ctenosciara hyalipennis|CNBRK105-14|Canada|BOLD_AAH3983
Ctenosciara hyalipennis|HPPPK354-13|Canada|BOLD_AAH3983
Ctenosciara hyalipennis|HPPPK652-13|Canada|BOLD_AAH3983
Ctenosciara hyalipennis|HPPPK281-13|Canada|BOLD_AAH3983
Ctenosciara hyalipennis|HPPPK345-13|Canada|BOLD_AAH3983
Ctenosciara hyalipennis|HPPPJ654-13|Canada|BOLD_AAH3983
Ctenosciara hyalipennis|HPPPJ956-13|Canada|BOLD_AAH3983
Ctenosciara hyalipennis|HPPPJ395-13|Canada|BOLD_AAH3983
Ctenosciara hyalipennis|HPPPJ606-13|Canada|BOLD_AAH3983
Ctenosciara hyalipennis|HPPPJ199-13|Canada|BOLD_AAH3983
Ctenosciara hyalipennis|HPPPJ253-13|Canada|BOLD_AAH3983
Ctenosciara hyalipennis|GMGRF1302-13|Germany|BOLD_AAH3983
Ctenosciara hyalipennis|HPPPJ171-13|Canada|BOLD_AAH3983
Ctenosciara hyalipennis|HPPPF051-13|Canada|BOLD_AAH3983
Ctenosciara hyalipennis|HPPPF777-13|Canada|BOLD_AAH3983
Ctenosciara hyalipennis|HPPPE1186-13|Canada|BOLD_AAH3983
Ctenosciara hyalipennis|HPPPF041-13|Canada|BOLD_AAH3983
Ctenosciara hyalipennis|HPPPE1094-13|Canada|BOLD_AAH3983
Ctenosciara hyalipennis|HPPPE1144-13|Canada|BOLD_AAH3983
Ctenosciara hyalipennis|HPPPE776-13|Canada|BOLD_AAH3983
Ctenosciara hyalipennis|HPPPE786-13|Canada|BOLD_AAH3983
Ctenosciara hyalipennis|HPPPE732-13|Canada|BOLD_AAH3983
Ctenosciara hyalipennis|HPPPE760-13|Canada|BOLD_AAH3983
Ctenosciara hyalipennis|CNBRN203-14|Canada|BOLD_AAH3983
Ctenosciara hyalipennis|CNPEN151-14|Canada|BOLD_AAH3983
Ctenosciara hyalipennis|HPPPA041-13|Canada|BOLD_AAH3983
Ctenosciara hyalipennis|HPPPA042-13|Canada|BOLD_AAH3983
Ctenosciara hyalipennis|SSPEA373-15|Canada|BOLD_AAH3983
Ctenosciara hyalipennis|SSPEA419-15|Canada|BOLD_AAH3983
Ctenosciara hyalipennis|SSBRB3598-14|Canada|BOLD_AAH3983
Ctenosciara hyalipennis|SSBRC1148-14|Canada|BOLD_AAH3983
Ctenosciara hyalipennis|SSBRB3327-14|Canada|BOLD_AAH3983
Ctenosciara hyalipennis|SSBRB3366-14|Canada|BOLD_AAH3983
Ctenosciara hyalipennis|SSBRB3231-14|Canada|BOLD_AAH3983
Ctenosciara hyalipennis|SSBRB3246-14|Canada|BOLD_AAH3983
Ctenosciara hyalipennis|SSBRB2771-14|Canada|BOLD_AAH3983
Ctenosciara hyalipennis|SSBRB3208-14|Canada|BOLD_AAH3983
Ctenosciara hyalipennis|CNBRJ035-14|Canada|BOLD_AAH3983
Ctenosciara hyalipennis|CNBRO517-14|Canada|BOLD_AAH3983
Ctenosciara hyalipennis|CNPEL1129-14|Canada|BOLD_AAH3983
Ctenosciara hyalipennis|CNPEL1294-14|Canada|BOLD_AAH3983
Ctenosciara hyalipennis|CNBRK242-14|Canada|BOLD_AAH3983
Ctenosciara hyalipennis|CNPEL1096-14|Canada|BOLD_AAH3983
Ctenosciara hyalipennis|CNBRK187-14|Canada|BOLD_AAH3983
Ctenosciara hyalipennis|CNBRK234-14|Canada|BOLD_AAH3983
Ctenosciara hyalipennis|HPPPL022-13|Canada|BOLD_AAH3983
Ctenosciara hyalipennis|HPPPS032-13|Canada|BOLD_AAH3983
Ctenosciara hyalipennis|HPPPA317-13|Canada|BOLD_AAH3983
Ctenosciara hyalipennis|GMGRF2021-13|Germany|BOLD_AAH3983
Ctenosciara hyalipennis|HPPPL387-13|Canada|BOLD_AAH3983
Ctenosciara hyalipennis|SSBRB613-14|Canada|BOLD_AAH3983
Ctenosciara hyalipennis|CNPEB1369-14|Canada|BOLD_AAH3983
Ctenosciara hyalipennis|CNPEN411-14|Canada|BOLD_AAH3983
Ctenosciara hyalipennis|HPPPA415-13|Canada|BOLD_AAH3983
Ctenosciara hyalipennis|CNPEB552-14|Canada|BOLD_AAH3983
Ctenosciara hyalipennis|HPPPJ373-13|Canada|BOLD_AAH3983
Ctenosciara hyalipennis|HPPPR087-13|Canada|BOLD_AAH3983
Ctenosciara hyalipennis|CNPEB1360-14|Canada|BOLD_AAH3983
Ctenosciara hyalipennis|CNPEB1405-14|Canada|BOLD_AAH3983
Ctenosciara hyalipennis|HPPPS055-13|Canada|BOLD_AAH3983
Ctenosciara hyalipennis|CNBRB209-14|Canada|BOLD_AAH3983
Ctenosciara hyalipennis|HPPPJ315-13|Canada|BOLD_AAH3983
Ctenosciara hyalipennis|HPPPJ371-13|Canada|BOLD_AAH3983
Ctenosciara hyalipennis|HPPPE657-13|Canada|BOLD_AAH3983
Ctenosciara hyalipennis|HPPPE704-13|Canada|BOLD_AAH3983
Ctenosciara hyalipennis|HPPPB249-13|Canada|BOLD_AAH3983
Ctenosciara hyalipennis|HPPPE571-13|Canada|BOLD_AAH3983

Ctenosciara hyalipennis|[illegible]-13|Canada|BOLD_AAH3983
Ctenosciara hyalipennis|HPPPE704-13|Canada|BOLD_AAH3983
Ctenosciara hyalipennis|HPPPB249-13|Canada|BOLD_AAH3983
Ctenosciara hyalipennis|HPPPE571-13|Canada|BOLD_AAH3983
Ctenosciara hyalipennis|HPPPA475-13|Canada|BOLD_AAH3983
Ctenosciara hyalipennis|HPPPB200-13|Canada|BOLD_AAH3983
Ctenosciara hyalipennis|HPPPA390-13|Canada|BOLD_AAH3983
Ctenosciara hyalipennis|HPPPA392-13|Canada|BOLD_AAH3983
Ctenosciara hyalipennis|HPPPF072-13|Canada|BOLD_AAH3983
Ctenosciara hyalipennis|CNBRP1232-14|Canada|BOLD_AAH3983
Ctenosciara hyalipennis|HPPPE660-13|Canada|BOLD_AAH3983
Ctenosciara hyalipennis|CNBRN167-14|Canada|BOLD_AAH3983
Ctenosciara hyalipennis|HPPPA151-13|Canada|BOLD_AAH3983
Ctenosciara hyalipennis|HPPPE1074-13|Canada|BOLD_AAH3983
Ctenosciara hyalipennis|HPPPA107-13|Canada|BOLD_AAH3983
Ctenosciara hyalipennis|HPPPA130-13|Canada|BOLD_AAH3983
Ctenosciara hyalipennis|HPPPA413-13|Canada|BOLD_AAH3983
Ctenosciara hyalipennis|CNPEL586-14|Canada|BOLD_AAH3983
Ctenosciara hyalipennis|CNPEK171-14|Canada|BOLD_AAH3983
Ctenosciara hyalipennis|CNPEL1364-14|Canada|BOLD_AAH3983
Ctenosciara hyalipennis|HPPPA012-13|Canada|BOLD_AAH3983
Ctenosciara hyalipennis|HPPPE779-13|Canada|BOLD_AAH3983
Ctenosciara hyalipennis|CNBRP181-14|Canada|BOLD_AAH3983
Ctenosciara hyalipennis|CNBRP321-14|Canada|BOLD_AAH3983
Ctenosciara hyalipennis|HPPPA428-13|Canada|BOLD_AAH3983
Ctenosciara hyalipennis|HPPPA451-13|Canada|BOLD_AAH3983
Ctenosciara hyalipennis|CNBRA119-14|Canada|BOLD_AAH3983
Ctenosciara hyalipennis|SSBRB168-14|Canada|BOLD_AAH3983
Ctenosciara hyalipennis|HPPPE782-13|Canada|BOLD_AAH3983
Ctenosciara hyalipennis|CNBRN222-14|Canada|BOLD_AAH3983
Ctenosciara hyalipennis|CNBRR300-14|Canada|BOLD_AAH3983
Ctenosciara hyalipennis|SSBRB1600-14|Canada|BOLD_AAH3983
Ctenosciara hyalipennis|HPPPE643-13|Canada|BOLD_AAH3983
Ctenosciara hyalipennis|CNBRI100-14|Canada|BOLD_AAH3983
Ctenosciara hyalipennis|CNPEB1363-14|Canada|BOLD_AAH3983
Ctenosciara hyalipennis|SSPEA217-15|Canada|BOLD_AAH3983
Ctenosciara hyalipennis|CNPEF619-14|Canada|BOLD_AAH3983
Ctenosciara hyalipennis|CNBRK281-14|Canada|BOLD_AAH3983
Ctenosciara hyalipennis|CNBRN146-14|Canada|BOLD_AAH3983
Ctenosciara hyalipennis|CNPEN783-14|Canada|BOLD_AAH3983
Ctenosciara hyalipennis|HPPPS135-14|Canada|BOLD_AAH3983
Ctenosciara hyalipennis|HPPPS138-14|Canada|BOLD_AAH3983
Ctenosciara hyalipennis|HPPPS148-14|Canada|BOLD_AAH3983
Ctenosciara hyalipennis|CNPED804-14|Canada|BOLD_AAH3983
Ctenosciara hyalipennis|CNBRA163-14|Canada|BOLD_AAH3983
Ctenosciara hyalipennis|CNBRB172-14|Canada|BOLD_AAH3983
Ctenosciara hyalipennis|CNBRB179-14|Canada|BOLD_AAH3983
Ctenosciara hyalipennis|CNPEB201-14|Canada|BOLD_AAH3983
Ctenosciara hyalipennis|HPPPF091-13|Canada|BOLD_AAH3983
Ctenosciara hyalipennis|HPPPF779-13|Canada|BOLD_AAH3983
Ctenosciara hyalipennis|HPPPJ664-13|Canada|BOLD_AAH3983
Ctenosciara hyalipennis|HPPPJ665-13|Canada|BOLD_AAH3983
Ctenosciara hyalipennis|HPPPJ260-13|Canada|BOLD_AAH3983
Ctenosciara hyalipennis|HPPPJ263-13|Canada|BOLD_AAH3983
Ctenosciara hyalipennis|HPPPA035-13|Canada|BOLD_AAH3983
Ctenosciara hyalipennis|CNBRQ467-14|Canada|BOLD_AAH3983
Ctenosciara hyalipennis|HPPPE608-13|Canada|BOLD_AAH3983
Ctenosciara hyalipennis|CNBRN120-14|Canada|BOLD_AAH3983
Ctenosciara hyalipennis|CNBRQ206-14|Canada|BOLD_AAH3983
Ctenosciara hyalipennis|CNBRQ391-14|Canada|BOLD_AAH3983
Ctenosciara hyalipennis|HPPPJ210-13|Canada|BOLD_AAH3983
Ctenosciara hyalipennis|HPPPK383-13|Canada|BOLD_AAH3983
Ctenosciara hyalipennis|HPPPK700-13|Canada|BOLD_AAH3983
Ctenosciara hyalipennis|CNBRK447-14|Canada|BOLD_AAH3983
Ctenosciara hyalipennis|HPPPJ379-13|Canada|BOLD_AAH3983
Ctenosciara hyalipennis|CNBRA150-14|Canada|BOLD_AAH3983
Ctenosciara hyalipennis|CNPEL1727-14|Canada|BOLD_AAH3983
Ctenosciara hyalipennis|CNBRR298-14|Canada|BOLD_AAH3983
Ctenosciara hyalipennis|SSBRB434-14|Canada|BOLD_AAH3983
Ctenosciara hyalipennis|SSBRB2696-14|Canada|BOLD_AAH3983
Ctenosciara hyalipennis|SSBRB2842-14|Canada|BOLD_AAH3983
Ctenosciara hyalipennis|SMTPI1679-14|Canada|BOLD_AAH3983
Ctenosciara hyalipennis|CNPEL932-14|Canada|BOLD_AAH3983
Ctenosciara hyalipennis|CNPEL1198-14|Canada|BOLD_AAH3983
Ctenosciara hyalipennis|CNPEL1440-14|Canada|BOLD_AAH3983
Ctenosciara hyalipennis|CNPEM030-14|Canada|BOLD_AAH3983
Ctenosciara hyalipennis|CNPEL376-14|Canada|BOLD_AAH3983
Ctenosciara hyalipennis|CNPEL500-14|Canada|BOLD_AAH3983
Ctenosciara hyalipennis|CNPEL554-14|Canada|BOLD_AAH3983
Ctenosciara hyalipennis|CNPEL565-14|Canada|BOLD_AAH3983
Ctenosciara hyalipennis|HPPPA223-13|Canada|BOLD_AAH3983
Ctenosciara hyalipennis|HPPPA234-13|Canada|BOLD_AAH3983
Ctenosciara hyalipennis|HPPPA288-13|Canada|BOLD_AAH3983
Ctenosciara hyalipennis|HPPPA318-13|Canada|BOLD_AAH3983
Ctenosciara hyalipennis|HPPPF250-13|Canada|BOLD_AAH3983
Ctenosciara hyalipennis|HPPPF276-13|Canada|BOLD_AAH3983
Ctenosciara hyalipennis|CNBRK109-14|Canada|BOLD_AAH3983
Ctenosciara hyalipennis|CNBRN220-14|Canada|BOLD_AAH3983
Ctenosciara hyalipennis|CNPEB385-14|Canada|BOLD_AAH3983
Ctenosciara hyalipennis|SSBRB2783-14|Canada|BOLD_AAH3983
Ctenosciara hyalipennis|SSBRB3332-14|Canada|BOLD_AAH3983
Ctenosciara hyalipennis|SSBRC1350-14|Canada|BOLD_AAH3983
Ctenosciara hyalipennis|HPPPF421-13|Canada|BOLD_AAH3983
Ctenosciara hyalipennis|CNPEB1436-14|Canada|BOLD_AAH3983
Ctenosciara hyalipennis|CNPEI051-14|Canada|BOLD_AAH3983
Ctenosciara hyalipennis|CNBRK156-14|Canada|BOLD_AAH3983
Ctenosciara hyalipennis|CNPEB1443-14|Canada|BOLD_AAH3983
Ctenosciara hyalipennis|CNPEF521-14|Canada|BOLD_AAH3983
Ctenosciara hyalipennis|HPPPE1064-13|Canada|BOLD_AAH3983
Ctenosciara hyalipennis|SSBRB1617-14|Canada|BOLD_AAH3983
Ctenosciara hyalipennis|HPPPF880-13|Canada|BOLD_AAH3983
Ctenosciara hyalipennis|GMGRF2346-13|Germany|BOLD_AAH3983
[illegible]

Ctenosciara hyalipennis|SSBRB1617-14|Canada|BOLD_AAH3983
Ctenosciara hyalipennis|HPPPF880-13|Canada|BOLD_AAH3983
Ctenosciara hyalipennis|GMGRF2346-13|Germany|BOLD_AAH3983
Ctenosciara hyalipennis|HPPPK217-13|Canada|BOLD_AAH3983
Ctenosciara hyalipennis|CNBRN142-14|Canada|BOLD_AAH3983
Ctenosciara hyalipennis|HPPPF491-13|Canada|BOLD_AAH3983
Ctenosciara hyalipennis|HPPPF517-13|Canada|BOLD_AAH3983
Ctenosciara hyalipennis|HPPPF534-13|Canada|BOLD_AAH3983
Ctenosciara hyalipennis|HPPPF536-13|Canada|BOLD_AAH3983
Ctenosciara hyalipennis|HPPPE023-13|Canada|BOLD_AAH3983
Ctenosciara hyalipennis|HPPPF031-13|Canada|BOLD_AAH3983
Ctenosciara hyalipennis|HPPPJ005-13|Canada|BOLD_AAH3983
Ctenosciara hyalipennis|HPPPJ643-13|Canada|BOLD_AAH3983
Ctenosciara hyalipennis|HPPPJ362-13|Canada|BOLD_AAH3983
Ctenosciara hyalipennis|HPPPJ368-13|Canada|BOLD_AAH3983
Ctenosciara hyalipennis|CNPEB834-14|Canada|BOLD_AAH3983
Ctenosciara hyalipennis|CNPEL173-14|Canada|BOLD_AAH3983
Ctenosciara hyalipennis|HPPPA043-13|Canada|BOLD_AAH3983
Ctenosciara hyalipennis|HPPPA050-13|Canada|BOLD_AAH3983
Ctenosciara hyalipennis|HPPPE623-13|Canada|BOLD_AAH3983
Ctenosciara hyalipennis|HPPPF163-13|Canada|BOLD_AAH3983
Ctenosciara hyalipennis|HPPPA322-13|Canada|BOLD_AAH3983
Ctenosciara hyalipennis|HPPPA350-13|Canada|BOLD_AAH3983
Ctenosciara hyalipennis|HPPPA275-13|Canada|BOLD_AAH3983
Ctenosciara hyalipennis|CNPEM1438-14|Canada|BOLD_AAH3983
Ctenosciara hyalipennis|HPPPA249-13|Canada|BOLD_AAH3983
Ctenosciara hyalipennis|HPPPA346-13|Canada|BOLD_AAH3983
Ctenosciara hyalipennis|GMGRG623-13|Germany|BOLD_AAH3983
Ctenosciara hyalipennis|HPPPS136-14|Canada|BOLD_AAH3983
Ctenosciara hyalipennis|HPPPA204-13|Canada|BOLD_AAH3983
Ctenosciara hyalipennis|HPPPA206-13|Canada|BOLD_AAH3983
Ctenosciara hyalipennis|HPPPA207-13|Canada|BOLD_AAH3983
Ctenosciara hyalipennis|HPPPA253-13|Canada|BOLD_AAH3983
Ctenosciara hyalipennis|HPPPF406-13|Canada|BOLD_AAH3983
Ctenosciara hyalipennis|HPPPF798-13|Canada|BOLD_AAH3983
Ctenosciara hyalipennis|GMGRF083-13|Germany|BOLD_AAH3983
Ctenosciara hyalipennis|CNPEA152-14|Canada|BOLD_AAH3983
Ctenosciara hyalipennis|HPPPJ332-13|Canada|BOLD_AAH3983
Ctenosciara hyalipennis|CNBRN232-14|Canada|BOLD_AAH3983
Ctenosciara hyalipennis|CNBRP937-14|Canada|BOLD_AAH3983
Ctenosciara hyalipennis|CNBRQ398-14|Canada|
Ctenosciara hyalipennis|CNBRK169-14|Canada|BOLD_AAH3983
Ctenosciara hyalipennis|CNBRK176-14|Canada|BOLD_AAH3983
Ctenosciara hyalipennis|HPPPA227-13|Canada|BOLD_AAH3983
Ctenosciara hyalipennis|CNBRQ441-14|Canada|BOLD_AAH3983
Ctenosciara hyalipennis|HPPPA442-13|Canada|BOLD_AAH3983
Ctenosciara hyalipennis|HPPPE756-13|Canada|BOLD_AAH3983
Ctenosciara hyalipennis|CNPEM002-14|Canada|BOLD_AAH3983
Ctenosciara hyalipennis|CNPEM950-14|Canada|BOLD_AAH3983
Ctenosciara hyalipennis|CNPEL031-14|Canada|BOLD_AAH3983
Ctenosciara hyalipennis|CNPEL886-14|Canada|BOLD_AAH3983
Ctenosciara hyalipennis|SSBRA840-14|Canada|BOLD_AAH3983
Ctenosciara hyalipennis|SSBRB2195-14|Canada|BOLD_AAH3983
Ctenosciara hyalipennis|HPPPA394-13|Canada|BOLD_AAH3983
Ctenosciara hyalipennis|HPPPA439-13|Canada|BOLD_AAH3983
Ctenosciara hyalipennis|CNBRQ312-14|Canada|BOLD_AAH3983
Ctenosciara hyalipennis|CNBRQ364-14|Canada|BOLD_AAH3983
Ctenosciara hyalipennis|CNBRB200-14|Canada|BOLD_AAH3983
Ctenosciara hyalipennis|CNPEA922-14|Canada|BOLD_AAH3983
Ctenosciara hyalipennis|HPPPA016-13|Canada|BOLD_AAH3983
Ctenosciara hyalipennis|CNBRQ372-14|Canada|BOLD_AAH3983
Ctenosciara hyalipennis|CNPEL1449-14|Canada|BOLD_AAH3983
Ctenosciara hyalipennis|CNBRR119-14|Canada|BOLD_AAH3983
Ctenosciara hyalipennis|CNBRS070-14|Canada|BOLD_AAH3983
Ctenosciara hyalipennis|SSBRA793-14|Canada|BOLD_AAH3983
Ctenosciara hyalipennis|CNBRQ457-14|Canada|BOLD_AAH3983
Ctenosciara hyalipennis|CNBRS084-14|Canada|BOLD_AAH3983
Ctenosciara hyalipennis|HPPPR089-13|Canada|BOLD_AAH3983
Ctenosciara hyalipennis|SSBRB2642-14|Canada|BOLD_AAH3983
Ctenosciara hyalipennis|HPPPA355-13|Canada|BOLD_AAH3983
Ctenosciara hyalipennis|CNBRK127-14|Canada|BOLD_AAH3983
Ctenosciara hyalipennis|CNPEL135-14|Canada|BOLD_AAH3983
Ctenosciara hyalipennis|CNPEL322-14|Canada|BOLD_AAH3983
Ctenosciara hyalipennis|CNBRK199-14|Canada|BOLD_AAH3983
Ctenosciara hyalipennis|CNBRQ473-14|Canada|BOLD_AAH3983
Ctenosciara hyalipennis|GMGRF1183-13|Germany|BOLD_AAH3983
Ctenosciara hyalipennis|SSBRB2735-14|Canada|BOLD_AAH3983
Ctenosciara hyalipennis|CNBRJ147-14|Canada|BOLD_AAH3983
Ctenosciara hyalipennis|CNBRJ184-14|Canada|BOLD_AAH3983
Ctenosciara hyalipennis|CNBRJ192-14|Canada|BOLD_AAH3983
Ctenosciara hyalipennis|CNPEO647-14|Canada|BOLD_AAH3983
Ctenosciara hyalipennis|CNBRR179-14|Canada|BOLD_AAH3983
Ctenosciara hyalipennis|SSBRB228-14|Canada|BOLD_AAH3983
Ctenosciara hyalipennis|SSBRB2586-14|Canada|BOLD_AAH3983
Ctenosciara hyalipennis|SSBRB2750-14|Canada|BOLD_AAH3983
Ctenosciara hyalipennis|CNBRF209-14|Canada|BOLD_AAH3983
Ctenosciara hyalipennis|CNBRI072-14|Canada|BOLD_AAH3983
Ctenosciara hyalipennis|CNBRI102-14|Canada|BOLD_AAH3983
Ctenosciara hyalipennis|CNBRK087-14|Canada|BOLD_AAH3983
Ctenosciara hyalipennis|CNBRK257-14|Canada|BOLD_AAH3983
Ctenosciara hyalipennis|CNBRK261-14|Canada|BOLD_AAH3983
Ctenosciara hyalipennis|CNPEL345-14|Canada|BOLD_AAH3983
Ctenosciara hyalipennis|CNBRJ096-14|Canada|BOLD_AAH3983
Ctenosciara hyalipennis|CNPEL1127-14|Canada|BOLD_AAH3983
Ctenosciara hyalipennis|CNPEL1396-14|Canada|BOLD_AAH3983
Ctenosciara hyalipennis|CNPEL1566-14|Canada|BOLD_AAH3983
Ctenosciara hyalipennis|CNPEL1887-14|Canada|BOLD_AAH3983
Ctenosciara hyalipennis|SSBRB183-14|Canada|BOLD_AAH3983
Ctenosciara hyalipennis|SSBRB442-14|Canada|BOLD_AAH3983
Ctenosciara hyalipennis|SSBRA764-14|Canada|BOLD_AAH3983
Ctenosciara hyalipennis|SSBRB2704-14|Canada|BOLD_AAH3983
Ctenosciara hyalipennis|CNBRB138-14|Canada|BOLD_AAH3983

Ctenosciara hyalipennis|SSBRA764-14|Canada|BOLD_AAH3983
Ctenosciara hyalipennis|SSBRB2704-14|Canada|BOLD_AAH3983
Ctenosciara hyalipennis|CNBRB138-14|Canada|BOLD_AAH3983
Ctenosciara hyalipennis|CNBRB337-14|Canada|BOLD_AAH3983
Ctenosciara hyalipennis|CNPEF175-14|Canada|BOLD_AAH3983
Ctenosciara hyalipennis|CNBRK090-14|Canada|BOLD_AAH3983
Ctenosciara hyalipennis|CNPEM747-14|Canada|BOLD_AAH3983
Ctenosciara hyalipennis|CNBRQ841-14|Canada|BOLD_AAH3983
Ctenosciara hyalipennis|CNBRR121-14|Canada|BOLD_AAH3983
Ctenosciara hyalipennis|CNBRS110-14|Canada|BOLD_AAH3983
Ctenosciara hyalipennis|CNBRK140-14|Canada|BOLD_AAH3983
Ctenosciara hyalipennis|CNBRK158-14|Canada|BOLD_AAH3983
Ctenosciara hyalipennis|CNBRK211-14|Canada|BOLD_AAH3983
Ctenosciara hyalipennis|CNPEL174-14|Canada|BOLD_AAH3983
Ctenosciara hyalipennis|CNPEL215-14|Canada|BOLD_AAH3983
Ctenosciara hyalipennis|CNPEL419-14|Canada|BOLD_AAH3983
Ctenosciara hyalipennis|CNPEL440-14|Canada|BOLD_AAH3983
Ctenosciara hyalipennis|CNPEL1006-14|Canada|BOLD_AAH3983
Ctenosciara hyalipennis|HPPPA264-13|Canada|BOLD_AAH3983
Ctenosciara hyalipennis|HPPPA271-13|Canada|BOLD_AAH3983
Ctenosciara hyalipennis|HPPPA303-13|Canada|BOLD_AAH3983
Ctenosciara hyalipennis|HPPPA338-13|Canada|BOLD_AAH3983
Ctenosciara hyalipennis|HPPPA371-13|Canada|BOLD_AAH3983
Ctenosciara hyalipennis|HPPPE1141-13|Canada|BOLD_AAH3983
Ctenosciara hyalipennis|HPPPI766-13|Canada|BOLD_AAH3983
Ctenosciara hyalipennis|GMGRG1470-13|Germany|BOLD_AAH3983
Ctenosciara hyalipennis|HPPPA417-13|Canada|BOLD_AAH3983
Ctenosciara hyalipennis|CNPEB1431-14|Canada|BOLD_AAH3983
Ctenosciara hyalipennis|SSBRC1221-14|Canada|BOLD_AAH3983
Ctenosciara hyalipennis|SSBRC1313-14|Canada|BOLD_AAH3983
Ctenosciara hyalipennis|HPPPK440-13|Canada|BOLD_AAH3983
Ctenosciara hyalipennis|HPPPK688-13|Canada|BOLD_AAH3983
Ctenosciara hyalipennis|GMGRG342-13|Germany|BOLD_AAH3983
Ctenosciara hyalipennis|GMGRG588-13|Germany|BOLD_AAH3983
Ctenosciara hyalipennis|CNBRA130-14|Canada|BOLD_AAH3983
Ctenosciara hyalipennis|CNBRA133-14|Canada|BOLD_AAH3983
Ctenosciara hyalipennis|CNBRB174-14|Canada|BOLD_AAH3983
Ctenosciara hyalipennis|CNPEB195-14|Canada|BOLD_AAH3983
Ctenosciara hyalipennis|SSBRB2817-14|Canada|BOLD_AAH3983
Ctenosciara hyalipennis|SSBRB3262-14|Canada|BOLD_AAH3983
Ctenosciara hyalipennis|SSBRB3299-14|Canada|BOLD_AAH3983
Ctenosciara hyalipennis|SSBRB3326-14|Canada|BOLD_AAH3983
Ctenosciara hyalipennis|CNBRK100-14|Canada|BOLD_AAH3983
Ctenosciara hyalipennis|CNBRK120-14|Canada|BOLD_AAH3983
Ctenosciara hyalipennis|CNBRK124-14|Canada|BOLD_AAH3983
Ctenosciara hyalipennis|CNBRK128-14|Canada|BOLD_AAH3983
Ctenosciara hyalipennis|HPPPK392-13|Canada|BOLD_AAH3983
Ctenosciara hyalipennis|HPPPK405-13|Canada|BOLD_AAH3983
Ctenosciara hyalipennis|HPPPK422-13|Canada|BOLD_AAH3983
Ctenosciara hyalipennis|HPPPK436-13|Canada|BOLD_AAH3983
Ctenosciara hyalipennis|HPPPK263-13|Canada|BOLD_AAH3983
Ctenosciara hyalipennis|HPPPK295-13|Canada|BOLD_AAH3983
Ctenosciara hyalipennis|HPPPK314-13|Canada|BOLD_AAH3983
Ctenosciara hyalipennis|HPPPK344-13|Canada|BOLD_AAH3983
Ctenosciara hyalipennis|HPPPK201-13|Canada|BOLD_AAH3983
Ctenosciara hyalipennis|HPPPK202-13|Canada|BOLD_AAH3983
Ctenosciara hyalipennis|HPPPK222-13|Canada|BOLD_AAH3983
Ctenosciara hyalipennis|HPPPK261-13|Canada|BOLD_AAH3983
Ctenosciara hyalipennis|HPPPJ195-13|Canada|BOLD_AAH3983
Ctenosciara hyalipennis|CNPEA987-14|Canada|BOLD_AAH3983
Ctenosciara hyalipennis|CNPEC1055-14|Canada|BOLD_AAH3983
Ctenosciara hyalipennis|CNBRG007-14|Canada|BOLD_AAH3983
Ctenosciara hyalipennis|HPPPB285-13|Canada|BOLD_AAH3983
Ctenosciara hyalipennis|HPPPE636-13|Canada|BOLD_AAH3983
Ctenosciara hyalipennis|HPPPE1180-13|Canada|BOLD_AAH3983
Ctenosciara hyalipennis|HPPPI588-13|Canada|BOLD_AAH3983
Ctenosciara hyalipennis|HPPPJ959-13|Canada|BOLD_AAH3983
Ctenosciara hyalipennis|HPPPK185-13|Canada|BOLD_AAH3983
Ctenosciara hyalipennis|HPPPK193-13|Canada|BOLD_AAH3983
Ctenosciara hyalipennis|HPPPK195-13|Canada|BOLD_AAH3983
Ctenosciara hyalipennis|HPPPJ607-13|Canada|BOLD_AAH3983
Ctenosciara hyalipennis|HPPPJ612-13|Canada|BOLD_AAH3983
Ctenosciara hyalipennis|HPPPJ618-13|Canada|BOLD_AAH3983
Ctenosciara hyalipennis|HPPPJ651-13|Canada|BOLD_AAH3983
Ctenosciara hyalipennis|HPPPJ340-13|Canada|BOLD_AAH3983
Ctenosciara hyalipennis|HPPPJ347-13|Canada|BOLD_AAH3983
Ctenosciara hyalipennis|HPPPJ349-13|Canada|BOLD_AAH3983
Ctenosciara hyalipennis|HPPPJ357-13|Canada|BOLD_AAH3983
Ctenosciara hyalipennis|HPPPJ256-13|Canada|BOLD_AAH3983
Ctenosciara hyalipennis|HPPPJ268-13|Canada|BOLD_AAH3983
Ctenosciara hyalipennis|HPPPJ301-13|Canada|BOLD_AAH3983
Ctenosciara hyalipennis|HPPPJ319-13|Canada|BOLD_AAH3983
Ctenosciara hyalipennis|HPPPJ238-13|Canada|BOLD_AAH3983
Ctenosciara hyalipennis|HPPPJ245-13|Canada|BOLD_AAH3983
Ctenosciara hyalipennis|HPPPJ248-13|Canada|BOLD_AAH3983
Ctenosciara hyalipennis|HPPPJ251-13|Canada|BOLD_AAH3983
Ctenosciara hyalipennis|HPPPJ148-13|Canada|BOLD_AAH3983
Ctenosciara hyalipennis|HPPPJ175-13|Canada|BOLD_AAH3983
Ctenosciara hyalipennis|HPPPJ235-13|Canada|BOLD_AAH3983
Ctenosciara hyalipennis|HPPPJ237-13|Canada|BOLD_AAH3983
Ctenosciara hyalipennis|HPPPF122-13|Canada|BOLD_AAH3983
Ctenosciara hyalipennis|HPPPF140-13|Canada|BOLD_AAH3983
Ctenosciara hyalipennis|HPPPF231-13|Canada|BOLD_AAH3983
Ctenosciara hyalipennis|HPPPF384-13|Canada|BOLD_AAH3983
Ctenosciara hyalipennis|HPPPF832-13|Canada|BOLD_AAH3983
Ctenosciara hyalipennis|HPPPF848-13|Canada|BOLD_AAH3983
Ctenosciara hyalipennis|HPPPJ117-13|Canada|BOLD_AAH3983
Ctenosciara hyalipennis|HPPPJ125-13|Canada|BOLD_AAH3983
Ctenosciara hyalipennis|HPPPJ656-13|Canada|BOLD_AAH3983
Ctenosciara hyalipennis|HPPPJ955-13|Canada|BOLD_AAH3983
Ctenosciara hyalipennis|CNBRK277-14|Canada|BOLD_AAH3983
Ctenosciara hyalipennis|CNBRK285-14|Canada|BOLD_AAH3983

Ctenosciara hyalipennis|HPPPJ955-13|Canada|BOLD_AAH3983
Ctenosciara hyalipennis|CNBRK277-14|Canada|BOLD_AAH3983
Ctenosciara hyalipennis|CNBRK285-14|Canada|BOLD_AAH3983
Ctenosciara hyalipennis|HPPPE685-13|Canada|BOLD_AAH3983
Ctenosciara hyalipennis|HPPPF047-13|Canada|BOLD_AAH3983
Ctenosciara hyalipennis|CNBRP473-14|Canada|BOLD_AAH3983
Ctenosciara hyalipennis|CNBRP1014-14|Canada|BOLD_AAH3983
Ctenosciara hyalipennis|HPPPK378-13|Canada|BOLD_AAH3983
Ctenosciara hyalipennis|HPPPK380-13|Canada|BOLD_AAH3983
Ctenosciara hyalipennis|GMGRA1345-13|Germany|BOLD_AAH3983
Ctenosciara hyalipennis|CNBRL467-14|Canada|BOLD_AAH3983
Ctenosciara hyalipennis|CNPEQ322-14|Canada|BOLD_AAH3983
Ctenosciara hyalipennis|CNPEQ567-14|Canada|BOLD_AAH3983
Ctenosciara hyalipennis|CNPER068-14|Canada|BOLD_AAH3983
Ctenosciara hyalipennis|CNBRP435-14|Canada|BOLD_AAH3983
Ctenosciara hyalipennis|CNPEL762-14|Canada|BOLD_AAH3983
Ctenosciara hyalipennis|CNBRL482-14|Canada|BOLD_AAH3983
Ctenosciara hyalipennis|CNBRL625-14|Canada|BOLD_AAH3983
Ctenosciara hyalipennis|CNPEQ157-14|Canada|BOLD_AAH3983
Ctenosciara hyalipennis|CNBRK310-14|Canada|BOLD_AAH3983
Ctenosciara hyalipennis|CNBRK341-14|Canada|BOLD_AAH3983
Ctenosciara hyalipennis|CNBRK364-14|Canada|BOLD_AAH3983
Ctenosciara hyalipennis|CNPEL738-14|Canada|BOLD_AAH3983
Ctenosciara hyalipennis|CNPED399-14|Canada|BOLD_AAH3983
Ctenosciara hyalipennis|CNPED846-14|Canada|BOLD_AAH3983
Ctenosciara hyalipennis|CNBRG107-14|Canada|BOLD_AAH3983
Ctenosciara hyalipennis|CNBRG136-14|Canada|BOLD_AAH3983
Ctenosciara hyalipennis|CNPEB214-14|Canada|BOLD_AAH3983
Ctenosciara hyalipennis|CNPEB262-14|Canada|BOLD_AAH3983
Ctenosciara hyalipennis|CNPEB463-14|Canada|BOLD_AAH3983
Ctenosciara hyalipennis|CNPEB623-14|Canada|BOLD_AAH3983
Ctenosciara hyalipennis|CNBRB302-14|Canada|BOLD_AAH3983
Ctenosciara hyalipennis|CNBRC148-14|Canada|BOLD_AAH3983
Ctenosciara hyalipennis|CNBRC152-14|Canada|BOLD_AAH3983
Ctenosciara hyalipennis|CNPEA102-14|Canada|BOLD_AAH3983
Ctenosciara hyalipennis|CNBRB198-14|Canada|BOLD_AAH3983
Ctenosciara hyalipennis|CNBRB220-14|Canada|BOLD_AAH3983
Ctenosciara hyalipennis|CNBRB227-14|Canada|BOLD_AAH3983
Ctenosciara hyalipennis|CNBRB261-14|Canada|BOLD_AAH3983
Ctenosciara hyalipennis|CNBRA161-14|Canada|BOLD_AAH3983
Ctenosciara hyalipennis|CNBRB127-14|Canada|BOLD_AAH3983
Ctenosciara hyalipennis|CNBRB141-14|Canada|BOLD_AAH3983
Ctenosciara hyalipennis|CNBRB166-14|Canada|BOLD_AAH3983
Ctenosciara hyalipennis|HPPPF005-13|Canada|BOLD_AAH3983
Ctenosciara hyalipennis|HPPPF211-13|Canada|BOLD_AAH3983
Ctenosciara hyalipennis|CNPEA566-14|Canada|BOLD_AAH3983
Ctenosciara hyalipennis|CNPEA648-14|Canada|BOLD_AAH3983
Ctenosciara hyalipennis|GMGRB609-13|Germany|BOLD_AAH3983
Ctenosciara hyalipennis|HPPPA021-13|Canada|BOLD_AAH3983
Ctenosciara hyalipennis|HPPPA037-13|Canada|BOLD_AAH3983
Ctenosciara hyalipennis|HPPPA080-13|Canada|BOLD_AAH3983
Ctenosciara hyalipennis|HPPPA062-13|Canada|BOLD_AAH3983
Ctenosciara hyalipennis|HPPPA064-13|Canada|BOLD_AAH3983
Ctenosciara hyalipennis|HPPPA069-13|Canada|BOLD_AAH3983
Ctenosciara hyalipennis|HPPPA075-13|Canada|BOLD_AAH3983
Ctenosciara hyalipennis|HPPPA053-13|Canada|BOLD_AAH3983
Ctenosciara hyalipennis|HPPPA054-13|Canada|BOLD_AAH3983
Ctenosciara hyalipennis|HPPPA055-13|Canada|BOLD_AAH3983
Ctenosciara hyalipennis|HPPPA058-13|Canada|BOLD_AAH3983
Ctenosciara hyalipennis|GMGRA474-13|Germany|BOLD_AAH3983
Ctenosciara hyalipennis|GMGRA737-13|Germany|BOLD_AAH3983
Ctenosciara hyalipennis|HPPPA187-13|Canada|BOLD_AAH3983
Ctenosciara hyalipennis|CNPEL1366-14|Canada|BOLD_AAH3983
Ctenosciara hyalipennis|CNPEQ166-14|Canada|BOLD_AAH3983
Ctenosciara hyalipennis|CNBRQ168-14|Canada|BOLD_AAH3983
Ctenosciara hyalipennis|SSBRB3248-14|Canada|BOLD_AAH3983
Ctenosciara hyalipennis|SSBRB3274-14|Canada|BOLD_AAH3983
Ctenosciara hyalipennis|HPPPA082-13|Canada|BOLD_AAH3983
Ctenosciara hyalipennis|HPPPM306-13|Canada|BOLD_AAH3983
Ctenosciara hyalipennis|HPPPA025-13|Canada|BOLD_AAH3983
Ctenosciara hyalipennis|HPPPA038-13|Canada|BOLD_AAH3983
Ctenosciara hyalipennis|HPPPJ327-13|Canada|BOLD_AAH3983
Ctenosciara hyalipennis|HPPPJ331-13|Canada|BOLD_AAH3983
Ctenosciara hyalipennis|HPPPA326-13|Canada|BOLD_AAH3983
Ctenosciara hyalipennis|HPPPA337-13|Canada|BOLD_AAH3983
Ctenosciara hyalipennis|HPPPA235-13|Canada|BOLD_AAH3983
Ctenosciara hyalipennis|CNPEL1718-14|Canada|BOLD_AAH3983
Ctenosciara hyalipennis|GMGRB190-13|Germany|BOLD_AAH3983
Ctenosciara hyalipennis|HPPPA002-13|Canada|BOLD_AAH3983
Ctenosciara hyalipennis|GMGRA229-13|Germany|BOLD_AAH3983
Ctenosciara hyalipennis|GMGRA649-13|Germany|BOLD_AAH3983
Ctenosciara hyalipennis|HPPPA071-13|Canada|BOLD_AAH3983
Ctenosciara hyalipennis|SSBRB3219-14|Canada|BOLD_AAH3983
Ctenosciara hyalipennis|HPPPA266-13|Canada|BOLD_AAH3983
Ctenosciara hyalipennis|HPPPE633-13|Canada|BOLD_AAH3983
Ctenosciara hyalipennis|HPPPA030-13|Canada|BOLD_AAH3983
Ctenosciara hyalipennis|HPPPA084-13|Canada|BOLD_AAH3983
Ctenosciara hyalipennis|GMGRB475-13|Germany|BOLD_AAH3983
Ctenosciara hyalipennis|CNBRK316-14|Canada|BOLD_AAH3983
Ctenosciara hyalipennis|CNBRP590-14|Canada|BOLD_AAH3983
Ctenosciara hyalipennis|CNBRS118-14|Canada|BOLD_AAH3983
Ctenosciara hyalipennis|HPPPA312-13|Canada|BOLD_AAH3983
Ctenosciara hyalipennis|CNBRK322-14|Canada|BOLD_AAH3983
Ctenosciara hyalipennis|HPPPA277-13|Canada|BOLD_AAH3983
Ctenosciara hyalipennis|HPPPA279-13|Canada|BOLD_AAH3983
Ctenosciara hyalipennis|HPPPA060-13|Canada|BOLD_AAH3983
Ctenosciara hyalipennis|HPPPA250-13|Canada|BOLD_AAH3983
Ctenosciara hyalipennis|HPPPM283-13|Canada|BOLD_AAH3983
Ctenosciara hyalipennis|CNBRL472-14|Canada|BOLD_AAH3983
Ctenosciara hyalipennis|CNBRK351-14|Canada|BOLD_AAH3983
Ctenosciara hyalipennis|CNBRN201-14|Canada|BOLD_AAH3983
Ctenosciara hyalipennis|CNBRK337-14|Canada|BOLD_AAH3983

Ctenosciara hyalipennis|[illegible]|Canada|BOLD_AAH3983
Ctenosciara hyalipennis|CNBRK351-14|Canada|BOLD_AAH3983
Ctenosciara hyalipennis|CNBRN201-14|Canada|BOLD_AAH3983
Ctenosciara hyalipennis|CNBRK337-14|Canada|BOLD_AAH3983
Ctenosciara hyalipennis|CNBRK349-14|Canada|BOLD_AAH3983
Ctenosciara hyalipennis|CNBRK321-14|Canada|BOLD_AAH3983
Ctenosciara hyalipennis|CNBRK326-14|Canada|BOLD_AAH3983
Ctenosciara hyalipennis|CNBRK288-14|Canada|BOLD_AAH3983
Ctenosciara hyalipennis|CNBRK306-14|Canada|BOLD_AAH3983
Ctenosciara hyalipennis|HPPPA380-13|Canada|BOLD_AAH3983
Ctenosciara hyalipennis|HPPPS019-13|Canada|BOLD_AAH3983
Ctenosciara hyalipennis|CNBRN227-14|Canada|BOLD_AAH3983
Ctenosciara hyalipennis|CNBRN234-14|Canada|BOLD_AAH3983
Ctenosciara hyalipennis|HPPPA293-13|Canada|BOLD_AAH3983
Ctenosciara hyalipennis|HPPPA334-13|Canada|BOLD_AAH3983
Ctenosciara hyalipennis|HPPPA079-13|Canada|BOLD_AAH3983
Ctenosciara hyalipennis|HPPPA241-13|Canada|BOLD_AAH3983
Ctenosciara hyalipennis|HPPPA004-13|Canada|BOLD_AAH3983
Ctenosciara hyalipennis|HPPPA070-13|Canada|BOLD_AAH3983
Ctenosciara hyalipennis|CNBRN212-14|Canada|BOLD_AAH3983
Ctenosciara hyalipennis|CNBRN226-14|Canada|BOLD_AAH3983
Ctenosciara hyalipennis|HPPPA347-13|Canada|BOLD_AAH3983
Ctenosciara hyalipennis|CNBRN192-14|Canada|BOLD_AAH3983
Ctenosciara hyalipennis|HPPPA068-13|Canada|BOLD_AAH3983
Ctenosciara hyalipennis|HPPPA072-13|Canada|BOLD_AAH3983
Ctenosciara hyalipennis|HPPPA036-13|Canada|BOLD_AAH3983
Ctenosciara hyalipennis|HPPPA046-13|Canada|BOLD_AAH3983
Ctenosciara hyalipennis|HPPPA022-13|Canada|BOLD_AAH3983
Ctenosciara hyalipennis|HPPPA026-13|Canada|BOLD_AAH3983
Ctenosciara hyalipennis|HPPPA063-13|Canada|BOLD_AAH3983
Ctenosciara hyalipennis|HPPPA261-13|Canada|BOLD_AAH3983
Ctenosciara hyalipennis|HPPPA078-13|Canada|BOLD_AAH3983
Ctenosciara hyalipennis|HPPPA093-13|Canada|BOLD_AAH3983
Ctenosciara hyalipennis|HPPPA045-13|Canada|BOLD_AAH3983
Ctenosciara hyalipennis|HPPPA049-13|Canada|BOLD_AAH3983
Ctenosciara hyalipennis|CNBRK445-14|Canada|BOLD_AAH3983
Ctenosciara hyalipennis|CNPEN170-14|Canada|BOLD_AAH3983
Ctenosciara hyalipennis|CNBRB121-14|Canada|BOLD_AAH3983
Ctenosciara hyalipennis|CNBRK325-14|Canada|BOLD_AAH3983
Ctenosciara hyalipennis|HPPPA161-13|Canada|BOLD_AAH3983
Ctenosciara hyalipennis|HPPPF385-13|Canada|BOLD_AAH3983
Ctenosciara hyalipennis|HPPPJ002-13|Canada|BOLD_AAH3983
Ctenosciara hyalipennis|HPPPJ155-13|Canada|BOLD_AAH3983
Ctenosciara hyalipennis|HPPPJ194-13|Canada|BOLD_AAH3983
Ctenosciara hyalipennis|HPPPK197-13|Canada|BOLD_AAH3983
Ctenosciara hyalipennis|HPPPS049-13|Canada|BOLD_AAH3983
Ctenosciara hyalipennis|CNPEA1025-14|Canada|BOLD_AAH3983
Ctenosciara hyalipennis|CNPEB119-14|Canada|BOLD_AAH3983
Ctenosciara hyalipennis|CNPEB288-14|Canada|BOLD_AAH3983
Ctenosciara hyalipennis|CNPEB315-14|Canada|BOLD_AAH3983
Ctenosciara hyalipennis|CNPEQ469-14|Canada|BOLD_AAH3983
Ctenosciara hyalipennis|CNBRP344-14|Canada|BOLD_AAH3983
Ctenosciara hyalipennis|HPPPA089-13|Canada|BOLD_AAH3983
Ctenosciara hyalipennis|HPPPA404-13|Canada|BOLD_AAH3983
Ctenosciara hyalipennis|CNBRB334-14|Canada|BOLD_AAH3983
Ctenosciara hyalipennis|GMGRB570-13|Germany|BOLD_AAH3983
Ctenosciara hyalipennis|GMGRA054-13|Germany|BOLD_AAH3983
Ctenosciara hyalipennis|HPPPA255-13|Canada|BOLD_AAH3983
Ctenosciara hyalipennis|HPPPA067-13|Canada|BOLD_AAH3983
Ctenosciara hyalipennis|HPPPA066-13|Canada|BOLD_AAH3983
Ctenosciara hyalipennis|HPPPA065-13|Canada|BOLD_AAH3983
Ctenosciara hyalipennis|GBMWN1426-15|Germany|BOLD_AAH3983
Ctenosciara hyalipennis|HPPPA244-13|Canada|BOLD_AAH3983
Ctenosciara hyalipennis|HPPPA224-13|Canada|BOLD_AAH3983
Ctenosciara hyalipennis|HPPPA214-13|Canada|BOLD_AAH3983
Ctenosciara hyalipennis|HPPPA212-13|Canada|BOLD_AAH3983
Ctenosciara hyalipennis|HPPPA194-13|Canada|BOLD_AAH3983
Ctenosciara hyalipennis|HPPPA219-13|Canada|BOLD_AAH3983
Ctenosciara hyalipennis|HPPPF706-13|Canada|BOLD_AAH3983
Ctenosciara hyalipennis|HPPPS127-13|Canada|BOLD_AAH3983
Ctenosciara hyalipennis|SSBRB474-14|Canada|BOLD_AAH3983
Ctenosciara hyalipennis|GBMWN576-15|Germany|BOLD_AAH3983
Ctenosciara hyalipennis|GBMWN549-15|Germany|BOLD_AAH3983
Ctenosciara hyalipennis|CNBRB354-14|Canada|BOLD_AAH3983
Ctenosciara hyalipennis|CNBRB295-14|Canada|BOLD_AAH3983
Ctenosciara hyalipennis|CNBRB278-14|Canada|BOLD_AAH3983
Ctenosciara hyalipennis|CNBRB244-14|Canada|BOLD_AAH3983
Ctenosciara hyalipennis|CNBRA065-14|Canada|BOLD_AAH3983
Ctenosciara hyalipennis|HPPPE519-13|Canada|BOLD_AAH3983
Ctenosciara hyalipennis|CNPEL519-14|Canada|BOLD_AAH3983
Ctenosciara hyalipennis|GBMWN464-15|Germany|BOLD_AAH3983
Ctenosciara hyalipennis|GBMWN391-15|Germany|BOLD_AAH3983
Ctenosciara hyalipennis|GBMWN388-15|Germany|BOLD_AAH3983
Ctenosciara hyalipennis|HPPPA024-13|Canada|BOLD_AAH3983
Ctenosciara hyalipennis|HPPPA282-13|Canada|BOLD_AAH3983
Ctenosciara hyalipennis|CNPEG267-14|Canada|BOLD_AAH3983
Ctenosciara hyalipennis|CNPEQ140-14|Canada|BOLD_AAH3983
Ctenosciara hyalipennis|HPPPA294-13|Canada|BOLD_AAH3983
Ctenosciara hyalipennis|CNBRR158-14|Canada|BOLD_AAH3983
Ctenosciara hyalipennis|HPPPA465-13|Canada|BOLD_AAH3983
Ctenosciara hyalipennis|SSBRB270-14|Canada|BOLD_AAH3983
Ctenosciara hyalipennis|HPPPA083-13|Canada|BOLD_AAH3983
Ctenosciara hyalipennis|CNBRG096-14|Canada|BOLD_AAH3983
Ctenosciara hyalipennis|SSBRB570-14|Canada|BOLD_AAH3983
Ctenosciara hyalipennis|SSBRC1147-14|Canada|BOLD_AAH3983
Ctenosciara hyalipennis|GBMWN384-15|Germany|BOLD_AAH3983
Ctenosciara hyalipennis|GBMWN356-15|Germany|BOLD_AAH3983
Ctenosciara hyalipennis|GBMWN346-15|Germany|BOLD_AAH3983
Ctenosciara hyalipennis|GBMWN339-15|Germany|BOLD_AAH3983
Ctenosciara hyalipennis|GBMWN329-15|Germany|BOLD_AAH3983
Ctenosciara hyalipennis|CNPEB932-14|Canada|BOLD_AAH3983
Ctenosciara hyalipennis|SSFDC3434-14|Canada|BOLD_AAH3983
[illegible]

Ctenosciara hyalipennis|GBMWN329-15|Germany|BOLD_AAH3983
Ctenosciara hyalipennis|CNPEB932-14|Canada|BOLD_AAH3983
Ctenosciara hyalipennis|SSFDC3434-14|Canada|BOLD_AAH3983
Ctenosciara hyalipennis|SSFDC4270-14|Canada|BOLD_AAH3983
Ctenosciara hyalipennis|GMGRB295-13|Germany|BOLD_AAH3983
Ctenosciara hyalipennis|CNKJB061-14|Canada|BOLD_AAH3983
Ctenosciara hyalipennis|GMGRB280-13|Germany|BOLD_AAH3983
Ctenosciara hyalipennis|GMGRA258-13|Germany|BOLD_AAH3983
Ctenosciara hyalipennis|SSFDC4274-14|Canada|BOLD_AAH3983
Ctenosciara hyalipennis|SSFDC4038-14|Canada|BOLD_AAH3983
Ctenosciara hyalipennis|SSFDC4110-14|Canada|BOLD_AAH3983
Ctenosciara hyalipennis|CNKJB515-14|Canada|BOLD_AAH3983
Ctenosciara hyalipennis|SSFDC4015-14|Canada|BOLD_AAH3983
Ctenosciara hyalipennis|GMGRA1186-13|Germany|BOLD_AAH3983
Ctenosciara hyalipennis|GMGRA294-13|Germany|BOLD_AAH3983
Ctenosciara hyalipennis|CNKJB353-14|Canada|BOLD_AAH3983
Ctenosciara hyalipennis|SSFDC4561-14|Canada|BOLD_AAH3983
Ctenosciara hyalipennis|GMGRA395-13|Germany|BOLD_AAH3983
Ctenosciara hyalipennis|CNKJA271-14|Canada|BOLD_AAH3983
Ctenosciara hyalipennis|CNKJB454-14|Canada|BOLD_AAH3983
Ctenosciara hyalipennis|CNPEM3070-14|Canada|BOLD_AAH3983
Ctenosciara hyalipennis|CNKJA185-14|Canada|BOLD_AAH3983
Ctenosciara hyalipennis|GMGRB681-13|Germany|BOLD_AAH3983
Ctenosciara hyalipennis|SSFDC4276-14|Canada|BOLD_AAH3983
Ctenosciara hyalipennis|GMGRA673-13|Germany|BOLD_AAH3983
Ctenosciara hyalipennis|GMGRA1277-13|Germany|BOLD_AAH3983
Ctenosciara hyalipennis|GMGRA829-13|Germany|BOLD_AAH3983
Ctenosciara hyalipennis|GMGRB520-13|Germany|BOLD_AAH3983
Ctenosciara hyalipennis|GMGRA1327-13|Germany|BOLD_AAH3983
Ctenosciara hyalipennis|GMGRA659-13|Germany|BOLD_AAH3983
Ctenosciara hyalipennis|GMGRA644-13|Germany|BOLD_AAH3983
Ctenosciara hyalipennis|GMGRA228-13|Germany|BOLD_AAH3983
Ctenosciara hyalipennis|GMGRA091-13|Germany|BOLD_AAH3983
Ctenosciara hyalipennis|SSFDC4224-14|Canada|BOLD_AAH3983
Ctenosciara hyalipennis|SSFDC3950-14|Canada|BOLD_AAH3983
Ctenosciara hyalipennis|SSFDC3024-14|Canada|BOLD_AAH3983
Ctenosciara hyalipennis|SSFDC4242-14|Canada|BOLD_AAH3983
Ctenosciara hyalipennis|SSFDC3474-14|Canada|BOLD_AAH3983
Ctenosciara hyalipennis|SSFDC3505-14|Canada|BOLD_AAH3983
Ctenosciara hyalipennis|SSFDC4665-14|Canada|BOLD_AAH3983
Ctenosciara hyalipennis|CNPEC1621-14|Canada|BOLD_AAH3983
Ctenosciara hyalipennis|CNBRL558-14|Canada|BOLD_AAH3983
Ctenosciara hyalipennis|CNPEB1577-14|Canada|BOLD_AAH3983
Ctenosciara hyalipennis|SSFDC4144-14|Canada|BOLD_AAH3983
Ctenosciara hyalipennis|SSFDC4250-14|Canada|BOLD_AAH3983
Ctenosciara hyalipennis|CNPEB1740-14|Canada|BOLD_AAH3983
Ctenosciara hyalipennis|CNKJA168-14|Canada|BOLD_AAH3983
Ctenosciara hyalipennis|CNPEN271-14|Canada|BOLD_AAH3983
Ctenosciara hyalipennis|CNPEB1149-14|Canada|BOLD_AAH3983
Ctenosciara hyalipennis|CNPEG089-14|Canada|BOLD_AAH3983
Ctenosciara hyalipennis|CNPEB960-14|Canada|BOLD_AAH3983
Ctenosciara hyalipennis|CNKJB030-14|Canada|BOLD_AAH3983
Ctenosciara hyalipennis|SSFDC589-14|Canada|BOLD_AAH3983
Ctenosciara hyalipennis|CNRVG1770-15|Canada|BOLD_AAH3983
Ctenosciara hyalipennis|CNGAI201-15|Canada|BOLD_AAH3983
Ctenosciara hyalipennis|CNPEQ036-14|Canada|BOLD_AAH3983
Ctenosciara hyalipennis|CNPEL757-14|Canada|BOLD_AAH3983
Ctenosciara hyalipennis|SSFDB1270-14|Canada|BOLD_AAH3983
Ctenosciara hyalipennis|CNPEB1671-14|Canada|BOLD_AAH3983
Ctenosciara hyalipennis|CNPEB1679-14|Canada|BOLD_AAH3983
Ctenosciara hyalipennis|CNPEB939-14|Canada|BOLD_AAH3983
Ctenosciara hyalipennis|CNPEB1117-14|Canada|BOLD_AAH3983
Ctenosciara hyalipennis|CNPEB190-14|Canada|BOLD_AAH3983
Ctenosciara hyalipennis|CNPEB275-14|Canada|BOLD_AAH3983
Ctenosciara hyalipennis|CNBRB303-14|Canada|BOLD_AAH3983
Ctenosciara hyalipennis|CNPEB076-14|Canada|BOLD_AAH3983
Ctenosciara hyalipennis|CNFDC195-14|Canada|BOLD_AAH3983
Ctenosciara hyalipennis|CNFDI092-14|Canada|BOLD_AAH3983
Ctenosciara hyalipennis|GMGRG331-13|Germany|BOLD_AAH3983
Ctenosciara hyalipennis|CNFDL459-14|Canada|BOLD_AAH3983
Ctenosciara hyalipennis|CNPEB704-14|Canada|BOLD_AAH3983
Ctenosciara hyalipennis|CNPEB756-14|Canada|BOLD_AAH3983
Ctenosciara hyalipennis|CNPEB637-14|Canada|BOLD_AAH3983
Ctenosciara hyalipennis|CNPEB639-14|Canada|BOLD_AAH3983
Ctenosciara hyalipennis|CNPEB296-14|Canada|BOLD_AAH3983
Ctenosciara hyalipennis|CNPEB308-14|Canada|BOLD_AAH3983
Ctenosciara hyalipennis|CNPEA670-14|Canada|BOLD_AAH3983
Ctenosciara hyalipennis|CNPEB258-14|Canada|BOLD_AAH3983
Ctenosciara hyalipennis|CNPEA080-14|Canada|BOLD_AAH3983
Ctenosciara hyalipennis|CNPEA127-14|Canada|BOLD_AAH3983
Ctenosciara hyalipennis|CNPEB1546-14|Canada|BOLD_AAH3983
Ctenosciara hyalipennis|CNPEB1566-14|Canada|BOLD_AAH3983
Ctenosciara hyalipennis|CNPEB943-14|Canada|BOLD_AAH3983
Ctenosciara hyalipennis|CNPEB1021-14|Canada|BOLD_AAH3983
Ctenosciara hyalipennis|CNPEB782-14|Canada|BOLD_AAH3983
Ctenosciara hyalipennis|CNPEB886-14|Canada|BOLD_AAH3983
Ctenosciara hyalipennis|CNPEB1576-14|Canada|BOLD_AAH3983
Ctenosciara hyalipennis|CNPEB1582-14|Canada|BOLD_AAH3983
Ctenosciara hyalipennis|CNPEB1592-14|Canada|BOLD_AAH3983
Ctenosciara hyalipennis|CNPED779-14|Canada|BOLD_AAH3983
Ctenosciara hyalipennis|CNBRK406-14|Canada|BOLD_AAH3983
Ctenosciara hyalipennis|CNPEQ343-14|Canada|BOLD_AAH3983
Ctenosciara hyalipennis|CNPEQ393-14|Canada|BOLD_AAH3983
Ctenosciara hyalipennis|SSFDB193-14|Canada|BOLD_AAH3983
Ctenosciara hyalipennis|SSFDB1299-14|Canada|BOLD_AAH3983
Ctenosciara hyalipennis|SSFDB1334-14|Canada|BOLD_AAH3983
Ctenosciara hyalipennis|SSFDB1345-14|Canada|BOLD_AAH3983
Ctenosciara hyalipennis|SSFDB1377-14|Canada|BOLD_AAH3983
Ctenosciara hyalipennis|SSFDC3241-14|Canada|BOLD_AAH3983
Ctenosciara hyalipennis|SSFDC3335-14|Canada|BOLD_AAH3983
Ctenosciara hyalipennis|SSFDB1316-14|Canada|BOLD_AAH3983
Ctenosciara hyalipennis|SSFDC3382-14|Canada|BOLD_AAH3983

Ctenosciara hyalipennis|SSFDC3335-14|Canada|BOLD_AAH3983
Ctenosciara hyalipennis|SSFDB1316-14|Canada|BOLD_AAH3983
Ctenosciara hyalipennis|SSFDC3382-14|Canada|BOLD_AAH3983
Ctenosciara hyalipennis|CNPEB059-14|Canada|BOLD_AAH3983
Ctenosciara hyalipennis|SSFDB1304-14|Canada|BOLD_AAH3983
Ctenosciara hyalipennis|CNPEN331-14|Canada|BOLD_AAH3983
Ctenosciara hyalipennis|CNBRL527-14|Canada|BOLD_AAH3983
Ctenosciara hyalipennis|SSFDC3977-14|Canada|BOLD_AAH3983
Ctenosciara hyalipennis|GMGRF1813-13|Germany|BOLD_AAH3983
Ctenosciara hyalipennis|CNPEG395-14|Canada|BOLD_AAH3983
Ctenosciara hyalipennis|CNPEL1511-14|Canada|BOLD_AAH3983
Ctenosciara hyalipennis|SSFDC4818-14|Canada|BOLD_AAH3983
Ctenosciara hyalipennis|SSFDB3759-14|Canada|BOLD_AAH3983
Ctenosciara hyalipennis|SSFDC4678-14|Canada|BOLD_AAH3983
Ctenosciara hyalipennis|SSFDC4700-14|Canada|BOLD_AAH3983
Ctenosciara hyalipennis|SSFDC4577-14|Canada|BOLD_AAH3983
Ctenosciara hyalipennis|SSFDC4653-14|Canada|BOLD_AAH3983
Ctenosciara hyalipennis|SSFDC4365-14|Canada|BOLD_AAH3983
Ctenosciara hyalipennis|SSFDC4370-14|Canada|BOLD_AAH3983
Ctenosciara hyalipennis|SSFDC4327-14|Canada|BOLD_AAH3983
Ctenosciara hyalipennis|SSFDC4329-14|Canada|BOLD_AAH3983
Ctenosciara hyalipennis|CNPEB1098-14|Canada|BOLD_AAH3983
Ctenosciara hyalipennis|SSFDC4295-14|Canada|BOLD_AAH3983
Ctenosciara hyalipennis|CNPEB904-14|Canada|BOLD_AAH3983
Ctenosciara hyalipennis|CNPEB365-14|Canada|BOLD_AAH3983
Ctenosciara hyalipennis|CNPEB814-14|Canada|BOLD_AAH3983
Ctenosciara hyalipennis|CNPEB286-14|Canada|BOLD_AAH3983
Ctenosciara hyalipennis|SSFDC4740-14|Canada|BOLD_AAH3983
Ctenosciara hyalipennis|SSFDC1496-14|Canada|BOLD_AAH3983
Ctenosciara hyalipennis|SSFDC4751-14|Canada|BOLD_AAH3983
Ctenosciara hyalipennis|SSFDC4764-14|Canada|BOLD_AAH3983
Ctenosciara hyalipennis|SSFDC4709-14|Canada|BOLD_AAH3983
Ctenosciara hyalipennis|SSFDC4737-14|Canada|BOLD_AAH3983
Ctenosciara hyalipennis|SSFDC1893-14|Canada|BOLD_AAH3983
Ctenosciara hyalipennis|SSFDC4698-14|Canada|BOLD_AAH3983
Ctenosciara hyalipennis|CNPEL1654-14|Canada|BOLD_AAH3983
Ctenosciara hyalipennis|SSFDC1492-14|Canada|BOLD_AAH3983
Ctenosciara hyalipennis|CNPEL1619-14|Canada|BOLD_AAH3983
Ctenosciara hyalipennis|CNPEL1635-14|Canada|BOLD_AAH3983
Ctenosciara hyalipennis|CNPEL814-14|Canada|BOLD_AAH3983
Ctenosciara hyalipennis|CNPEL988-14|Canada|BOLD_AAH3983
Ctenosciara hyalipennis|CNPEL1077-14|Canada|BOLD_AAH3983
Ctenosciara hyalipennis|CNPEL1119-14|Canada|BOLD_AAH3983
Ctenosciara hyalipennis|GMGRG875-13|Germany|BOLD_AAH3983
Ctenosciara hyalipennis|CNGUA217-14|Canada|BOLD_AAH3983
Ctenosciara hyalipennis|SSFDB687-14|Canada|BOLD_AAH3983
Ctenosciara hyalipennis|SSFDB762-14|Canada|BOLD_AAH3983
Ctenosciara hyalipennis|SSFDB628-14|Canada|BOLD_AAH3983
Ctenosciara hyalipennis|SSFDB656-14|Canada|BOLD_AAH3983
Ctenosciara hyalipennis|CNPEL1745-14|Canada|BOLD_AAH3983
Ctenosciara hyalipennis|CNPEL1770-14|Canada|BOLD_AAH3983
Ctenosciara hyalipennis|CNPEL1888-14|Canada|BOLD_AAH3983
Ctenosciara hyalipennis|CNPEM1115-14|Canada|BOLD_AAH3983
Ctenosciara hyalipennis|CNPEM1856-14|Canada|BOLD_AAH3983
Ctenosciara hyalipennis|CNKJN376-14|Canada|BOLD_AAH3983
Ctenosciara hyalipennis|CNPEL1697-14|Canada|BOLD_AAH3983
Ctenosciara hyalipennis|CNPEL1709-14|Canada|BOLD_AAH3983
Ctenosciara hyalipennis|CNPEL1668-14|Canada|BOLD_AAH3983
Ctenosciara hyalipennis|CNPEL1686-14|Canada|BOLD_AAH3983
Ctenosciara hyalipennis|CNPEL647-14|Canada|BOLD_AAH3983
Ctenosciara hyalipennis|CNPEL1027-14|Canada|BOLD_AAH3983
Ctenosciara hyalipennis|SSFDB097-14|Canada|BOLD_AAH3983
Ctenosciara hyalipennis|SSFDB341-14|Canada|BOLD_AAH3983
Ctenosciara hyalipennis|SSFDB395-14|Canada|BOLD_AAH3983
Ctenosciara hyalipennis|SSFDB486-14|Canada|BOLD_AAH3983
Ctenosciara hyalipennis|SSFDB1070-14|Canada|BOLD_AAH3983
Ctenosciara hyalipennis|SSFDC634-14|Canada|BOLD_AAH3983
Ctenosciara hyalipennis|SSFDC765-14|Canada|BOLD_AAH3983
Ctenosciara hyalipennis|SSFDC798-14|Canada|BOLD_AAH3983
Ctenosciara hyalipennis|SSFDC813-14|Canada|BOLD_AAH3983
Ctenosciara hyalipennis|SSFDC1245-14|Canada|BOLD_AAH3983
Ctenosciara hyalipennis|SSFDC1371-14|Canada|BOLD_AAH3983
Ctenosciara hyalipennis|SSFDC1544-14|Canada|BOLD_AAH3983
Ctenosciara hyalipennis|SSFDC1754-14|Canada|BOLD_AAH3983
Ctenosciara hyalipennis|SSFDC1840-14|Canada|BOLD_AAH3983
Ctenosciara hyalipennis|SSFDC1853-14|Canada|BOLD_AAH3983
Ctenosciara hyalipennis|SSFDC1867-14|Canada|BOLD_AAH3983
Ctenosciara hyalipennis|SSFDC1970-14|Canada|BOLD_AAH3983
Ctenosciara hyalipennis|SSFDC2007-14|Canada|BOLD_AAH3983
Ctenosciara hyalipennis|SSFDC2024-14|Canada|BOLD_AAH3983
Ctenosciara hyalipennis|SSFDC2085-14|Canada|BOLD_AAH3983
Ctenosciara hyalipennis|SSFDB2595-14|Canada|BOLD_AAH3983
Ctenosciara hyalipennis|SSFDB2638-14|Canada|BOLD_AAH3983
Ctenosciara hyalipennis|SSFDC2718-14|Canada|BOLD_AAH3983
Ctenosciara hyalipennis|SSFDC3003-14|Canada|BOLD_AAH3983
Ctenosciara hyalipennis|SSFDC3527-14|Canada|BOLD_AAH3983
Ctenosciara hyalipennis|SSFDC3581-14|Canada|BOLD_AAH3983
Ctenosciara hyalipennis|SSFDC3613-14|Canada|BOLD_AAH3983
Ctenosciara hyalipennis|SSFDC3646-14|Canada|BOLD_AAH3983
Ctenosciara hyalipennis|SSFDC3940-14|Canada|BOLD_AAH3983
Ctenosciara hyalipennis|SSFDC3988-14|Canada|BOLD_AAH3983
Ctenosciara hyalipennis|SSFDC4292-14|Canada|BOLD_AAH3983
Ctenosciara hyalipennis|SSFDC4389-14|Canada|BOLD_AAH3983
Ctenosciara hyalipennis|SSFDC4422-14|Canada|BOLD_AAH3983
Ctenosciara hyalipennis|SSFDC4564-14|Canada|BOLD_AAH3983
Ctenosciara hyalipennis|SSFDC4566-14|Canada|BOLD_AAH3983
Ctenosciara hyalipennis|SSFDC4571-14|Canada|BOLD_AAH3983
Ctenosciara hyalipennis|SSFDC4574-14|Canada|BOLD_AAH3983
Ctenosciara hyalipennis|SSFDC4586-14|Canada|BOLD_AAH3983
Ctenosciara hyalipennis|SSFDC4592-14|Canada|BOLD_AAH3983
Ctenosciara hyalipennis|SSFDC4614-14|Canada|BOLD_AAH3983
Ctenosciara hyalipennis|SSFDC4626-14|Canada|BOLD_AAH3983

Ctenosciara hyalipennis|SSFDC4592-14|Canada|BOLD_AAH3983
Ctenosciara hyalipennis|SSFDC4614-14|Canada|BOLD_AAH3983
Ctenosciara hyalipennis|SSFDC4626-14|Canada|BOLD_AAH3983
Ctenosciara hyalipennis|SSFDC4627-14|Canada|BOLD_AAH3983
Ctenosciara hyalipennis|SSFDC4628-14|Canada|BOLD_AAH3983
Ctenosciara hyalipennis|SSFDC4667-14|Canada|BOLD_AAH3983
Ctenosciara hyalipennis|SSFDC4800-14|Canada|BOLD_AAH3983
Ctenosciara hyalipennis|SMTPJ5714-14|Canada|BOLD_AAH3983
Ctenosciara hyalipennis|CNRVF171-15|Canada|BOLD_AAH3983
Ctenosciara hyalipennis|CNRVG787-15|Canada|BOLD_AAH3983
Ctenosciara hyalipennis|CNPEL553-14|Canada|BOLD_AAH3983
Ctenosciara hyalipennis|CNPEL601-14|Canada|BOLD_AAH3983
Ctenosciara hyalipennis|CNPEL063-14|Canada|BOLD_AAH3983
Ctenosciara hyalipennis|CNPEL249-14|Canada|BOLD_AAH3983
Ctenosciara hyalipennis|CNPEL1575-14|Canada|BOLD_AAH3983
Ctenosciara hyalipennis|CNPEL1578-14|Canada|BOLD_AAH3983
Ctenosciara hyalipennis|CNPEL1091-14|Canada|BOLD_AAH3983
Ctenosciara hyalipennis|CNPEL1235-14|Canada|BOLD_AAH3983
Ctenosciara hyalipennis|CNPEL1032-14|Canada|BOLD_AAH3983
Ctenosciara hyalipennis|CNPEL1076-14|Canada|BOLD_AAH3983
Ctenosciara hyalipennis|SSFDC4756-14|Canada|BOLD_AAH3983
Ctenosciara hyalipennis|SSFDC4765-14|Canada|BOLD_AAH3983
Ctenosciara hyalipennis|GMGRG1940-13|Germany|BOLD_AAH3983
Ctenosciara hyalipennis|CNFDB143-14|Canada|BOLD_AAH3983
Ctenosciara hyalipennis|SSFDC4754-14|Canada|BOLD_AAH3983
Ctenosciara hyalipennis|SSFDC4755-14|Canada|BOLD_AAH3983
Ctenosciara hyalipennis|GMGRG1248-13|Germany|BOLD_AAH3983
Ctenosciara hyalipennis|GMGRG1605-13|Germany|BOLD_AAH3983
Ctenosciara hyalipennis|CNFDB186-14|Canada|BOLD_AAH3983
Ctenosciara hyalipennis|GMGRG735-13|Germany|BOLD_AAH3983
Ctenosciara hyalipennis|GMGRG1623-13|Germany|BOLD_AAH3983
Ctenosciara hyalipennis|SSFDC4037-14|Canada|BOLD_AAH3983
Ctenosciara hyalipennis|SSFDC5933-14|Canada|BOLD_AAH3983
Ctenosciara hyalipennis|SSFDC4769-14|Canada|BOLD_AAH3983
Ctenosciara hyalipennis|SSFDC4054-14|Canada|BOLD_AAH3983
Ctenosciara hyalipennis|SSFDC4810-14|Canada|BOLD_AAH3983
Ctenosciara hyalipennis|SSFDC806-14|Canada|BOLD_AAH3983
Ctenosciara hyalipennis|CNPEL1712-14|Canada|BOLD_AAH3983
Ctenosciara hyalipennis|CNPEL1408-14|Canada|BOLD_AAH3983
Ctenosciara hyalipennis|CNPEL1169-14|Canada|BOLD_AAH3983
Ctenosciara hyalipennis|CNPEL394-14|Canada|BOLD_AAH3983
Ctenosciara hyalipennis|CNKJB320-14|Canada|BOLD_AAH3983
Ctenosciara hyalipennis|SSFDB432-14|Canada|BOLD_AAH3983
Ctenosciara hyalipennis|CNPEB1124-14|Canada|BOLD_AAH3983
Ctenosciara hyalipennis|BBDEE274-10|Canada|BOLD_AAH3983
Ctenosciara hyalipennis|BBDED382-10|Canada|BOLD_AAH3983
Ctenosciara hyalipennis|BBDED381-10|Canada|BOLD_AAH3983
Ctenosciara hyalipennis|GMGRF826-13|Germany|BOLD_AAH3983
Ctenosciara hyalipennis|CNKJJ078-14|Canada|BOLD_AAH3983
Ctenosciara hyalipennis|CNPEN138-14|Canada|BOLD_AAH3983
Ctenosciara hyalipennis|CNPEB1066-14|Canada|BOLD_AAH3983
Ctenosciara hyalipennis|CNPEL767-14|Canada|BOLD_AAH3983
Ctenosciara hyalipennis|CNPEB723-14|Canada|BOLD_AAH3983
Ctenosciara hyalipennis|CNPEB981-14|Canada|BOLD_AAH3983
Ctenosciara hyalipennis|CNPEB638-14|Canada|BOLD_AAH3983
Ctenosciara hyalipennis|CNPEB675-14|Canada|BOLD_AAH3983
Ctenosciara hyalipennis|CNPEB085-14|Canada|BOLD_AAH3983
Ctenosciara hyalipennis|CNPEB587-14|Canada|BOLD_AAH3983
Ctenosciara hyalipennis|GMGRG377-13|Germany|BOLD_AAH3983
Ctenosciara hyalipennis|GMGRG155-13|Germany|BOLD_AAH3983
Ctenosciara hyalipennis|CNPED406-14|Canada|BOLD_AAH3983
Ctenosciara hyalipennis|GMGRF1768-13|Germany|BOLD_AAH3983
Ctenosciara hyalipennis|CNKJA098-14|Canada|BOLD_AAH3983
Ctenosciara hyalipennis|CNPEB507-14|Canada|BOLD_AAH3983
Ctenosciara hyalipennis|SSFDC4731-14|Canada|BOLD_AAH3983
Ctenosciara hyalipennis|SSFDC4344-14|Canada|BOLD_AAH3983
Ctenosciara hyalipennis|SSFDC4342-14|Canada|BOLD_AAH3983
Ctenosciara hyalipennis|SSFDC4152-14|Canada|BOLD_AAH3983
Ctenosciara hyalipennis|CNPEL887-14|Canada|BOLD_AAH3983
Ctenosciara hyalipennis|CNPEL940-14|Canada|BOLD_AAH3983
Ctenosciara hyalipennis|CNPEL477-14|Canada|BOLD_AAH3983
Ctenosciara hyalipennis|CNPEL499-14|Canada|BOLD_AAH3983
Ctenosciara hyalipennis|CNRVE2561-15|Canada|BOLD_AAH3983
Ctenosciara hyalipennis|SSPEA293-15|Canada|BOLD_AAH3983
Ctenosciara hyalipennis|CNPEL520-14|Canada|BOLD_AAH3983
Ctenosciara hyalipennis|CNPEL575-14|Canada|BOLD_AAH3983
Ctenosciara hyalipennis|SSFDC626-14|Canada|BOLD_AAH3983
Ctenosciara hyalipennis|SSFDC3564-14|Canada|BOLD_AAH3983
Ctenosciara hyalipennis|GMGRG2032-13|Germany|BOLD_AAH3983
Ctenosciara hyalipennis|CNPEL263-14|Canada|BOLD_AAH3983
Ctenosciara hyalipennis|GMGRF3016-13|Germany|BOLD_AAH3983
Ctenosciara hyalipennis|CNPEB1516-14|Canada|BOLD_AAH3983
Ctenosciara hyalipennis|CNPEL1304-14|Canada|BOLD_AAH3983
Ctenosciara hyalipennis|CNPEM524-14|Canada|BOLD_AAH3983
Ctenosciara hyalipennis|SSPEA314-15|Canada|BOLD_AAH3983
Ctenosciara hyalipennis|CNPEB428-14|Canada|BOLD_AAH3983
Ctenosciara hyalipennis|CNFDC138-14|Canada|BOLD_AAH3983
Ctenosciara hyalipennis|CNPEB1732-14|Canada|BOLD_AAH3983
Ctenosciara hyalipennis|CNPEM3062-14|Canada|BOLD_AAH3983
Ctenosciara hyalipennis|CNPEB1255-14|Canada|BOLD_AAH3983
Ctenosciara hyalipennis|CNPEB1726-14|Canada|BOLD_AAH3983
Ctenosciara hyalipennis|CNPEB1521-14|Canada|BOLD_AAH3983
Ctenosciara hyalipennis|CNPEB599-14|Canada|BOLD_AAH3983
Ctenosciara hyalipennis|CNPEB556-14|Canada|BOLD_AAH3983
Ctenosciara hyalipennis|CNPEB522-14|Canada|BOLD_AAH3983
Ctenosciara hyalipennis|CNPEB424-14|Canada|BOLD_AAH3983
Ctenosciara hyalipennis|CNPEB415-14|Canada|BOLD_AAH3983
Ctenosciara hyalipennis|GMGRG130-13|Germany|BOLD_AAH3983
Ctenosciara hyalipennis|CNKJA114-14|Canada|BOLD_AAH3983
Ctenosciara hyalipennis|CNKJB227-14|Canada|BOLD_AAH3983
Ctenosciara hyalipennis|GMGRG565-13|Germany|BOLD_AAH3983
Ctenosciara hyalipennis|GMGRG318-13|Germany|BOLD_AAH3983

Ctenosciara hyalipennis|CNKJA114-14|Canada|BOLD_AAH3983
Ctenosciara hyalipennis|CNKJB227-14|Canada|BOLD_AAH3983
Ctenosciara hyalipennis|GMGRG565-13|Germany|BOLD_AAH3983
Ctenosciara hyalipennis|GMGRG318-13|Germany|BOLD_AAH3983
Ctenosciara hyalipennis|GMGRG146-13|Germany|BOLD_AAH3983
Ctenosciara hyalipennis|CNPEB1503-14|Canada|BOLD_AAH3983
Ctenosciara hyalipennis|SSFDC2651-14|Canada|BOLD_AAH3983
Ctenosciara hyalipennis|CNFDL467-14|Canada|BOLD_AAH3983
Ctenosciara hyalipennis|CNPEB1501-14|Canada|BOLD_AAH3983
Ctenosciara hyalipennis|CNFDL508-14|Canada|BOLD_AAH3983
Ctenosciara hyalipennis|CNFDL502-14|Canada|BOLD_AAH3983
Ctenosciara hyalipennis|SSFDC4434-14|Canada|BOLD_AAH3983
Ctenosciara hyalipennis|SSFDC3989-14|Canada|BOLD_AAH3983
Ctenosciara hyalipennis|SSFDC3945-14|Canada|BOLD_AAH3983
Ctenosciara hyalipennis|SSFDC1614-14|Canada|BOLD_AAH3983
Ctenosciara hyalipennis|SSFDB1321-14|Canada|BOLD_AAH3983
Ctenosciara hyalipennis|SSFDC2607-14|Canada|BOLD_AAH3983
Ctenosciara hyalipennis|CNPEL725-14|Canada|BOLD_AAH3983
Ctenosciara hyalipennis|CNPEB271-14|Canada|BOLD_AAH3983
Ctenosciara hyalipennis|CNPEU149-14|Canada|BOLD_AAH3983
Ctenosciara hyalipennis|CNPEQ318-14|Canada|BOLD_AAH3983
Ctenosciara hyalipennis|GMGRG601-13|Germany|BOLD_AAH3983
Ctenosciara hyalipennis|CNPEB899-14|Canada|BOLD_AAH3983
Ctenosciara hyalipennis|SSFDC4527-14|Canada|BOLD_AAH3983
Ctenosciara hyalipennis|SSFDC4661-14|Canada|BOLD_AAH3983
Ctenosciara hyalipennis|CNPEL1728-14|Canada|BOLD_AAH3983
Ctenosciara hyalipennis|SSFDC3987-14|Canada|BOLD_AAH3983
Ctenosciara hyalipennis|CNPEL666-14|Canada|BOLD_AAH3983
Ctenosciara hyalipennis|CNPEL1309-14|Canada|BOLD_AAH3983
Ctenosciara hyalipennis|SSFDB1893-14|Canada|BOLD_AAH3983
Ctenosciara hyalipennis|SSFDC1011-14|Canada|BOLD_AAH3983
Ctenosciara hyalipennis|CNRVF438-15|Canada|BOLD_AAH3983
Ctenosciara hyalipennis|CNPEL094-14|Canada|BOLD_AAH3983
Ctenosciara hyalipennis|SSFDB1730-14|Canada|BOLD_AAH3983
Ctenosciara hyalipennis|CNKJF1001-14|Canada|BOLD_AAH3983
Ctenosciara hyalipennis|CNKJF910-14|Canada|BOLD_AAH3983
Ctenosciara hyalipennis|CNPEM1990-14|Canada|BOLD_AAH3983
Ctenosciara hyalipennis|CNPEL1426-14|Canada|BOLD_AAH3983
Ctenosciara hyalipennis|CNPEL228-14|Canada|BOLD_AAH3983
Ctenosciara hyalipennis|SSFDC4526-14|Canada|BOLD_AAH3983
Ctenosciara hyalipennis|SSFDC4427-14|Canada|BOLD_AAH3983
Ctenosciara hyalipennis|CNPEL1144-14|Canada|BOLD_AAH3983
Ctenosciara hyalipennis|CNPEG391-14|Canada|BOLD_AAH3983
Ctenosciara hyalipennis|CNPEL1587-14|Canada|BOLD_AAH3983
Ctenosciara hyalipennis|SSFDC4249-14|Canada|BOLD_AAH3983
Ctenosciara hyalipennis|CNTIF1892-15|Canada|BOLD_AAH3983
Ctenosciara hyalipennis|SSPEA378-15|Canada|BOLD_AAH3983
Ctenosciara hyalipennis|SSFDC4706-14|Canada|BOLD_AAH3983
Ctenosciara hyalipennis|SSFDC4644-14|Canada|BOLD_AAH3983
Ctenosciara hyalipennis|SSFDC4386-14|Canada|BOLD_AAH3983
Ctenosciara hyalipennis|CNKJA027-14|Canada|BOLD_AAH3983
Ctenosciara hyalipennis|CNPEM726-14|Canada|BOLD_AAH3983
Ctenosciara hyalipennis|CNPEL1896-14|Canada|BOLD_AAH3983
Ctenosciara hyalipennis|CNPEL815-14|Canada|BOLD_AAH3983
Ctenosciara hyalipennis|CNPEB906-14|Canada|BOLD_AAH3983
Ctenosciara hyalipennis|CNPEL1823-14|Canada|BOLD_AAH3983
Ctenosciara hyalipennis|CNPEL1252-14|Canada|BOLD_AAH3983
Ctenosciara hyalipennis|SSFDC1033-14|Canada|BOLD_AAH3983
Ctenosciara hyalipennis|SSFDC5922-14|Canada|BOLD_AAH3983
Ctenosciara hyalipennis|SSFDC5894-14|Canada|BOLD_AAH3983
Ctenosciara hyalipennis|SSFDC4210-14|Canada|BOLD_AAH3983
Ctenosciara hyalipennis|SSFDC4180-14|Canada|BOLD_AAH3983
Ctenosciara hyalipennis|SSFDC4094-14|Canada|BOLD_AAH3983
Ctenosciara hyalipennis|SSFDC4047-14|Canada|BOLD_AAH3983
Ctenosciara hyalipennis|SSFDC4020-14|Canada|BOLD_AAH3983
Ctenosciara hyalipennis|SSFDC4001-14|Canada|BOLD_AAH3983
Ctenosciara hyalipennis|CNFDB074-14|Canada|BOLD_AAH3983
Ctenosciara hyalipennis|CNPEB1063-14|Canada|BOLD_AAH3983
Ctenosciara hyalipennis|CNPEB655-14|Canada|BOLD_AAH3983
Ctenosciara hyalipennis|SSFDC5797-14|Canada|BOLD_AAH3983
Ctenosciara hyalipennis|SSFDC5775-14|Canada|BOLD_AAH3983
Ctenosciara hyalipennis|SSFDC5759-14|Canada|BOLD_AAH3983
Ctenosciara hyalipennis|SSFDC5473-14|Canada|BOLD_AAH3983
Ctenosciara hyalipennis|SSFDC4476-14|Canada|BOLD_AAH3983
Ctenosciara hyalipennis|SSFDC2877-14|Canada|BOLD_AAH3983
Ctenosciara hyalipennis|SSFDB2674-14|Canada|BOLD_AAH3983
Ctenosciara hyalipennis|SSBRB3953-14|Canada|BOLD_AAH3983
Ctenosciara hyalipennis|CNKJN1031-14|Canada|BOLD_AAH3983
Ctenosciara hyalipennis|CNKJB319-14|Canada|BOLD_AAH3983
Ctenosciara hyalipennis|CNKJB282-14|Canada|BOLD_AAH3983
Ctenosciara hyalipennis|CNPEM2960-14|Canada|BOLD_AAH3983
Ctenosciara hyalipennis|CNPEM2299-14|Canada|BOLD_AAH3983
Ctenosciara hyalipennis|CNPEM681-14|Canada|BOLD_AAH3983
Ctenosciara hyalipennis|CNPEL1692-14|Canada|BOLD_AAH3983
Ctenosciara hyalipennis|CNPEL1689-14|Canada|BOLD_AAH3983
Ctenosciara hyalipennis|CNPEL559-14|Canada|BOLD_AAH3983
Ctenosciara hyalipennis|GMGRG2364-13|Germany|BOLD_AAH3983
Ctenosciara hyalipennis|GMGRG1628-13|Germany|BOLD_AAH3983
Ctenosciara hyalipennis|GMGRG1282-13|Germany|BOLD_AAH3983
Ctenosciara hyalipennis|SSFDC4311-14|Canada|BOLD_AAH3983
Ctenosciara hyalipennis|CNPEB1163-14|Canada|BOLD_AAH3983
Ctenosciara hyalipennis|CNPEB390-14|Canada|BOLD_AAH3983
Ctenosciara hyalipennis|SSFDC5734-14|Canada|BOLD_AAH3983
Ctenosciara hyalipennis|SSPEA375-15|Canada|BOLD_AAH3983
Ctenosciara hyalipennis|SSFDC2924-14|Canada|BOLD_AAH3983
Ctenosciara hyalipennis|SSFDC649-14|Canada|BOLD_AAH3983
Ctenosciara hyalipennis|SSFDA715-14|Canada|BOLD_AAH3983
Ctenosciara hyalipennis|CNKJJ028-14|Canada|BOLD_AAH3983
Ctenosciara hyalipennis|CNPEL1816-14|Canada|BOLD_AAH3983
Ctenosciara hyalipennis|GMGRG3469-13|Germany|BOLD_AAH3983
Ctenosciara hyalipennis|CNPEB259-14|Canada|BOLD_AAH3983
Ctenosciara hyalipennis|CNPEB247-14|Canada|BOLD_AAH3983
[illegible]

Ctenosciara hyalipennis|GMGRG3469-13|Germany|BOLD_AAH3983
Ctenosciara hyalipennis|CNPEB259-14|Canada|BOLD_AAH3983
Ctenosciara hyalipennis|CNPEB247-14|Canada|BOLD_AAH3983
Ctenosciara hyalipennis|SSFDC2644-14|Canada|BOLD_AAH3983
Ctenosciara hyalipennis|CNPEB1725-14|Canada|BOLD_AAH3983
Ctenosciara hyalipennis|CNKJB210-14|Canada|BOLD_AAH3983
Ctenosciara hyalipennis|GMGRF1349-13|Germany|BOLD_AAH3983
Ctenosciara hyalipennis|GMGRF1218-13|Germany|BOLD_AAH3983
Ctenosciara hyalipennis|CNPES161-14|Canada|BOLD_AAH3983
Ctenosciara hyalipennis|CNFDD064-14|Canada|BOLD_AAH3983
Ctenosciara hyalipennis|CNFDC133-14|Canada|BOLD_AAH3983
Ctenosciara hyalipennis|CNPEB1333-14|Canada|BOLD_AAH3983
Ctenosciara hyalipennis|CNPEB1276-14|Canada|BOLD_AAH3983
Ctenosciara hyalipennis|CNPEB1239-14|Canada|BOLD_AAH3983
Ctenosciara hyalipennis|CNPEB607-14|Canada|BOLD_AAH3983
Ctenosciara hyalipennis|CNPEB402-14|Canada|BOLD_AAH3983
Ctenosciara hyalipennis|CNPEL781-14|Canada|BOLD_AAH3983
Ctenosciara hyalipennis|CNPEB1203-14|Canada|BOLD_AAH3983
Ctenosciara hyalipennis|SSPEA880-15|Canada|BOLD_AAH3983
Ctenosciara hyalipennis|SSPEA304-15|Canada|BOLD_AAH3983
Ctenosciara hyalipennis|SSFDC5560-14|Canada|BOLD_AAH3983
Ctenosciara hyalipennis|SSFDC4559-14|Canada|BOLD_AAH3983
Ctenosciara hyalipennis|SSFDC898-14|Canada|BOLD_AAH3983
Ctenosciara hyalipennis|CNKJK980-14|Canada|BOLD_AAH3983
Ctenosciara hyalipennis|CNKJB327-14|Canada|BOLD_AAH3983
Ctenosciara hyalipennis|CNPEM1858-14|Canada|BOLD_AAH3983
Ctenosciara hyalipennis|CNPEL968-14|Canada|BOLD_AAH3983
Ctenosciara hyalipennis|CNPEB1174-14|Canada|BOLD_AAH3983
Ctenosciara hyalipennis|GMGRG885-13|Germany|BOLD_AAH3983
Ctenosciara hyalipennis|CNKJA056-14|Canada|BOLD_AAH3983
Ctenosciara hyalipennis|SSPEA258-15|Canada|BOLD_AAH3983
Ctenosciara hyalipennis|CNGUB234-14|Canada|BOLD_AAH3983
Ctenosciara hyalipennis|SSFDC5864-14|Canada|BOLD_AAH3983
Ctenosciara hyalipennis|SSFDC2934-14|Canada|BOLD_AAH3983
Ctenosciara hyalipennis|SSFDC2930-14|Canada|BOLD_AAH3983
Ctenosciara hyalipennis|SSFDC2926-14|Canada|BOLD_AAH3983
Ctenosciara hyalipennis|SSFDC835-14|Canada|BOLD_AAH3983
Ctenosciara hyalipennis|SSFDB632-14|Canada|BOLD_AAH3983
Ctenosciara hyalipennis|CNKJN1098-14|Canada|BOLD_AAH3983
Ctenosciara hyalipennis|CNKJK387-14|Canada|BOLD_AAH3983
Ctenosciara hyalipennis|GMGRG1075-13|Germany|BOLD_AAH3983
Ctenosciara hyalipennis|CNPER204-14|Canada|BOLD_AAH3983
Ctenosciara hyalipennis|SSFDC4002-14|Canada|BOLD_AAH3983
Ctenosciara hyalipennis|SSFDC4012-14|Canada|BOLD_AAH3983
Ctenosciara hyalipennis|CNPEM387-14|Canada|BOLD_AAH3983
Ctenosciara hyalipennis|SSFDC3978-14|Canada|BOLD_AAH3983
Ctenosciara hyalipennis|SSFDC4022-14|Canada|BOLD_AAH3983
Ctenosciara hyalipennis|CNKJB181-14|Canada|BOLD_AAH3983
Ctenosciara hyalipennis|SSFDC3363-14|Canada|BOLD_AAH3983
Ctenosciara hyalipennis|CNKJP806-14|Canada|BOLD_AAH3983
Ctenosciara hyalipennis|CNKJA164-14|Canada|BOLD_AAH3983
Ctenosciara hyalipennis|CNPEB1465-14|Canada|BOLD_AAH3983
Ctenosciara hyalipennis|CNFDL371-14|Canada|BOLD_AAH3983
Ctenosciara hyalipennis|SSFDC4259-14|Canada|BOLD_AAH3983
Ctenosciara hyalipennis|SSFDC4254-14|Canada|BOLD_AAH3983
Ctenosciara hyalipennis|SSFDC4127-14|Canada|BOLD_AAH3983
Ctenosciara hyalipennis|SSFDC4121-14|Canada|BOLD_AAH3983
Ctenosciara hyalipennis|SSFDC4018-14|Canada|BOLD_AAH3983
Ctenosciara hyalipennis|CNPEM1339-14|Canada|BOLD_AAH3983
Ctenosciara hyalipennis|CNPEL1381-14|Canada|BOLD_AAH3983
Ctenosciara hyalipennis|CNPEL089-14|Canada|BOLD_AAH3983
Ctenosciara hyalipennis|GMGRG3002-13|Germany|BOLD_AAH3983
Ctenosciara hyalipennis|GMGRG2308-13|Germany|BOLD_AAH3983
Ctenosciara hyalipennis|GMGRG1379-13|Germany|BOLD_AAH3983
Ctenosciara hyalipennis|CNPEB1233-14|Canada|BOLD_AAH3983
Ctenosciara hyalipennis|SSFDC4223-14|Canada|BOLD_AAH3983
Ctenosciara hyalipennis|SSFDC4506-14|Canada|BOLD_AAH3983
Ctenosciara hyalipennis|GMGRF467-13|Germany|BOLD_AAH3983
Ctenosciara hyalipennis|SSFDC4719-14|Canada|BOLD_AAH3983
Ctenosciara hyalipennis|SSFDC719-14|Canada|BOLD_AAH3983
Ctenosciara hyalipennis|CNPEM2791-14|Canada|BOLD_AAH3983
Ctenosciara hyalipennis|CNPEL1763-14|Canada|BOLD_AAH3983
Ctenosciara hyalipennis|CNPEL1610-14|Canada|BOLD_AAH3983
Ctenosciara hyalipennis|CNPEL637-14|Canada|BOLD_AAH3983
Ctenosciara hyalipennis|CNPEL317-14|Canada|BOLD_AAH3983
Ctenosciara hyalipennis|GMGRI591-13|Germany|BOLD_AAH3983
Ctenosciara hyalipennis|GMGRG4616-13|Germany|BOLD_AAH3983
Ctenosciara hyalipennis|SSFDB1286-14|Canada|BOLD_AAH3983
Ctenosciara hyalipennis|GMGRF1491-13|Germany|BOLD_AAH3983
Ctenosciara hyalipennis|SSFDC4705-14|Canada|BOLD_AAH3983
Ctenosciara hyalipennis|SSFDC2972-14|Canada|BOLD_AAH3983
Ctenosciara hyalipennis|CNKJK953-14|Canada|BOLD_AAH3983
Ctenosciara hyalipennis|CNKJF301-14|Canada|BOLD_AAH3983
Ctenosciara hyalipennis|CNPEL1839-14|Canada|BOLD_AAH3983
Ctenosciara hyalipennis|SSFDC4061-14|Canada|BOLD_AAH3983
Ctenosciara hyalipennis|SSFDC4027-14|Canada|BOLD_AAH3983
Ctenosciara hyalipennis|CNKJA005-14|Canada|BOLD_AAH3983
Ctenosciara hyalipennis|SSFDC4221-14|Canada|BOLD_AAH3983
Ctenosciara hyalipennis|GMGRG2264-13|Germany|BOLD_AAH3983
Ctenosciara hyalipennis|SSFDC4475-14|Canada|BOLD_AAH3983
Ctenosciara hyalipennis|SSFDC4284-14|Canada|BOLD_AAH3983
Ctenosciara hyalipennis|CNPEB1345-14|Canada|BOLD_AAH3983
Ctenosciara hyalipennis|SSFDC4535-14|Canada|BOLD_AAH3983
Ctenosciara hyalipennis|SSFDC3389-14|Canada|BOLD_AAH3983
Ctenosciara hyalipennis|SSFDC3196-14|Canada|BOLD_AAH3983
Ctenosciara hyalipennis|CNPEO183-14|Canada|BOLD_AAH3983
Ctenosciara hyalipennis|CNKJJ055-14|Canada|BOLD_AAH3983
Ctenosciara hyalipennis|SSFDC4009-14|Canada|BOLD_AAH3983
Ctenosciara hyalipennis|SSFDC4456-14|Canada|BOLD_AAH3983
Ctenosciara hyalipennis|SSFDC4387-14|Canada|BOLD_AAH3983
Ctenosciara hyalipennis|CNPEB360-14|Canada|BOLD_AAH3983
Ctenosciara hyalipennis|SSFDC4426-14|Canada|BOLD_AAH3983

Ctenosciara hyalipennis|SSFDC4387-14|Canada|BOLD_AAH3983
Ctenosciara hyalipennis|CNPEB360-14|Canada|BOLD_AAH3983
Ctenosciara hyalipennis|SSFDC4426-14|Canada|BOLD_AAH3983
Ctenosciara hyalipennis|CNPEL888-14|Canada|BOLD_AAH3983
Ctenosciara hyalipennis|SSFDC1617-14|Canada|BOLD_AAH3983
Ctenosciara hyalipennis|SSFDC570-14|Canada|BOLD_AAH3983
Ctenosciara hyalipennis|CNPEL976-14|Canada|BOLD_AAH3983
Ctenosciara hyalipennis|SSFDC4536-14|Canada|BOLD_AAH3983
Ctenosciara hyalipennis|SSFDC4528-14|Canada|BOLD_AAH3983
Ctenosciara hyalipennis|SSFDC4288-14|Canada|BOLD_AAH3983
Ctenosciara hyalipennis|SSFDC4445-14|Canada|BOLD_AAH3983
Ctenosciara hyalipennis|CNPEB561-14|Canada|BOLD_AAH3983
Ctenosciara hyalipennis|CNPEB350-14|Canada|BOLD_AAH3983
Ctenosciara hyalipennis|GMGRF4907-13|Germany|BOLD_AAH3983
Ctenosciara hyalipennis|CNPER130-14|Canada|BOLD_AAH3983
Ctenosciara hyalipennis|GMGRF4892-13|Germany|BOLD_AAH3983
Ctenosciara hyalipennis|CNPEL1753-14|Canada|BOLD_AAH3983
Ctenosciara hyalipennis|CNPEL103-14|Canada|BOLD_AAH3983
Ctenosciara hyalipennis|CNKJA266-14|Canada|BOLD_AAH3983
Ctenosciara hyalipennis|SSFDC4089-14|Canada|BOLD_AAH3983
Ctenosciara hyalipennis|CNPEH063-14|Canada|BOLD_AAH3983
Ctenosciara hyalipennis|CNPEB1379-14|Canada|BOLD_AAH3983
Ctenosciara hyalipennis|CNPEB547-14|Canada|BOLD_AAH3983
Ctenosciara hyalipennis|SSFDC4534-14|Canada|BOLD_AAH3983
Ctenosciara hyalipennis|GMGRF205-13|Germany|BOLD_AAH3983
Ctenosciara hyalipennis|GMGRF369-13|Germany|BOLD_AAH3983
Ctenosciara hyalipennis|CNKJA049-14|Canada|BOLD_AAH3983
Ctenosciara hyalipennis|SSFDC4575-14|Canada|BOLD_AAH3983
Ctenosciara hyalipennis|SSFDB3421-14|Canada|BOLD_AAH3983
Ctenosciara hyalipennis|SSFDC4233-14|Canada|BOLD_AAH3983
Ctenosciara hyalipennis|SSFDC4066-14|Canada|BOLD_AAH3983
Ctenosciara hyalipennis|GMGRF194-13|Germany|BOLD_AAH3983
Ctenosciara hyalipennis|SSFDC2490-14|Canada|BOLD_AAH3983
Ctenosciara hyalipennis|GMGRG2316-13|Germany|BOLD_AAH3983
Ctenosciara hyalipennis|GMGRG4589-13|Germany|BOLD_AAH3983
Ctenosciara hyalipennis|SSFDC4502-14|Canada|BOLD_AAH3983
Ctenosciara hyalipennis|SSFDC4188-14|Canada|BOLD_AAH3983
Ctenosciara hyalipennis|SSFDC2994-14|Canada|BOLD_AAH3983
Ctenosciara hyalipennis|GMGRF1257-13|Germany|BOLD_AAH3983
Ctenosciara hyalipennis|SSFDC2819-14|Canada|BOLD_AAH3983
Ctenosciara hyalipennis|CNPEN139-14|Canada|BOLD_AAH3983
Ctenosciara hyalipennis|SSFDC4484-14|Canada|BOLD_AAH3983
Ctenosciara hyalipennis|CNPER156-14|Canada|BOLD_AAH3983
Ctenosciara hyalipennis|CNFDL525-14|Canada|BOLD_AAH3983
Ctenosciara hyalipennis|CNKJL403-14|Canada|BOLD_AAH3983
Ctenosciara hyalipennis|CNPEB1445-14|Canada|BOLD_AAH3983
Ctenosciara hyalipennis|CNKJJ058-14|Canada|BOLD_AAH3983
Ctenosciara hyalipennis|SSFDC2850-14|Canada|BOLD_AAH3983
Ctenosciara hyalipennis|CNPEB1515-14|Canada|BOLD_AAH3983
Ctenosciara hyalipennis|CNPEM3201-14|Canada|
Ctenosciara hyalipennis|SSFDC1059-14|Canada|BOLD_AAH3983
Ctenosciara hyalipennis|SSFDC5255-14|Canada|BOLD_AAH3983
Ctenosciara hyalipennis|SSPEA216-15|Canada|BOLD_AAH3983
Ctenosciara hyalipennis|SSPEA280-15|Canada|BOLD_AAH3983
Ctenosciara hyalipennis|SSPEA1043-15|Canada|BOLD_AAH3983
Ctenosciara hyalipennis|SCINO531-15|Norway|BOLD_AAH3983
Ctenosciara hyalipennis|SSFDC4388-14|Canada|BOLD_AAH3983
Ctenosciara hyalipennis|SSFDC4390-14|Canada|BOLD_AAH3983
Ctenosciara hyalipennis|SSFDC4410-14|Canada|BOLD_AAH3983
Ctenosciara hyalipennis|SSFDC4446-14|Canada|BOLD_AAH3983
Ctenosciara hyalipennis|SSFDC3002-14|Canada|BOLD_AAH3983
Ctenosciara hyalipennis|SSFDC4279-14|Canada|BOLD_AAH3983
Ctenosciara hyalipennis|SSFDC4332-14|Canada|BOLD_AAH3983
Ctenosciara hyalipennis|SSFDC4377-14|Canada|BOLD_AAH3983
Ctenosciara hyalipennis|SSFDC4457-14|Canada|BOLD_AAH3983
Ctenosciara hyalipennis|SSFDC4480-14|Canada|BOLD_AAH3983
Ctenosciara hyalipennis|SSFDC4507-14|Canada|BOLD_AAH3983
Ctenosciara hyalipennis|SSFDC4567-14|Canada|BOLD_AAH3983
Ctenosciara hyalipennis|SSFDC5828-14|Canada|BOLD_AAH3983
Ctenosciara hyalipennis|SSPEA371-15|Canada|BOLD_AAH3983
Ctenosciara hyalipennis|SSFDC4635-14|Canada|BOLD_AAH3983
Ctenosciara hyalipennis|SSFDC4711-14|Canada|BOLD_AAH3983
Ctenosciara hyalipennis|SSPEA428-15|Canada|BOLD_AAH3983
Ctenosciara hyalipennis|CNTIE321-15|Canada|BOLD_AAH3983
Ctenosciara hyalipennis|GBMWN368-15|Germany|BOLD_AAH3983
Ctenosciara hyalipennis|GBMWN1479-15|Germany|BOLD_AAH3983
Ctenosciara hyalipennis|GBMWN1569-15|Germany|BOLD_AAH3983
Ctenosciara exigua|HRCTE002-15|Finland|
Ctenosciara exigua|HRCTE003-15|Finland|
Ctenosciara hyalipennis|HPPPA007-13|Canada|BOLD_AAH3983
Ctenosciara hyalipennis|HPPPA052-13|Canada|BOLD_AAH3983
Ctenosciara hyalipennis|GMGRA1238-13|Germany|BOLD_AAH3983
Ctenosciara hyalipennis|GMGRA451-13|Germany|BOLD_AAH3983
Ctenosciara hyalipennis|HPPPM273-13|Canada|BOLD_AAH3983
Ctenosciara hyalipennis|HPPPA353-13|Canada|BOLD_AAH3983
Ctenosciara hyalipennis|CNBRK273-14|Canada|BOLD_AAH3983
Ctenosciara hyalipennis|CNPEL1470-14|Canada|BOLD_AAH3983
Ctenosciara hyalipennis|CNPEL1533-14|Canada|BOLD_AAH3983
Ctenosciara hyalipennis|HPPPA091-13|Canada|BOLD_AAH3983
Ctenosciara hyalipennis|CNPEL1754-14|Canada|BOLD_AAH3983
Ctenosciara hyalipennis|HPPPF117-13|Canada|BOLD_AAH3983
Ctenosciara hyalipennis|HPPPF419-13|Canada|BOLD_AAH3983
Ctenosciara hyalipennis|HPPPF408-13|Canada|BOLD_AAH3983
Ctenosciara hyalipennis|CNBRQ261-14|Canada|BOLD_AAH3983
Ctenosciara hyalipennis|CNPEL1596-14|Canada|BOLD_AAH3983
Ctenosciara hyalipennis|CNPEL1624-14|Canada|BOLD_AAH3983
Ctenosciara hyalipennis|CNBRQ300-14|Canada|BOLD_AAH3983
Ctenosciara hyalipennis|CNBRQ569-14|Canada|BOLD_AAH3983
Ctenosciara hyalipennis|GMGRF2120-13|Germany|BOLD_AAH3983
Ctenosciara hyalipennis|SSBRA343-14|Canada|BOLD_AAH3983
